# One-step synthesis of benzo[*b*]thiophenes by aryne reaction with alkynyl sulfides†

**DOI:** 10.1039/d0sc04450d

**Published:** 2020-09-01

**Authors:** Tsubasa Matsuzawa, Takamitsu Hosoya, Suguru Yoshida

**Affiliations:** Laboratory of Chemical Bioscience, Institute of Biomaterials and Bioengineering, Tokyo Medical and Dental University (TMDU) 2-3-10 Kanda-Surugadai Chiyoda-ku Tokyo 101-0062 Japan s-yoshida.cb@tmd.ac.jp

## Abstract

An aryne reaction with alkynyl sulfides affording benzo[*b*]thiophenes is disclosed. A wide range of 3-substituted benzothiophenes were synthesized from easily available *o*-silylaryl triflates and alkynyl sulfides in a one-step intermolecular manner. The synthesis of diverse multisubstituted benzothiophene derivatives involving a pentacyclic compound was achieved by virtue of the good functional group tolerance and versatile C2 functionalizations.

## Abstract 2

An aryne reaction with alkynyl sulfides affords benzo[*b*]thiophenes in a one-step simple procedure.

## Graphical abstract

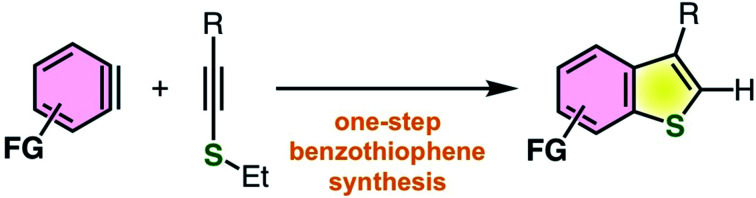

## Introduction

**Fig. 1:**
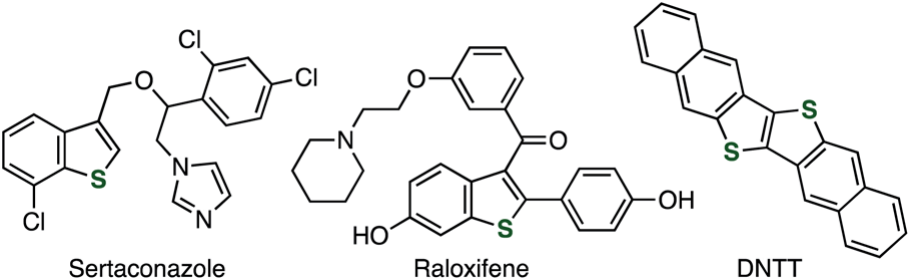
Various benzothiophene derivatives.

Benzo[*b*]thiophenes are a promising class of organosulfur compounds.^1–3^ In particular, multisubstituted benzothiophenes such as sertaconazole, raloxifene, and DNTT have served in a broad range of research fields including pharmaceutical sciences and materials chemistry (Fig. 1). In spite of their significance, although a number of benzothiophene syntheses such as transition-metal catalyzed reactions have been developed, the synthesis of multisubstituted benzothiophenes remains still difficult in terms of the applicable functional groups and substitution patterns due to the limited methods constructing the benzothiophene skeleton and introducing substituents.^4,5^ We herein present a novel approach to form benzothiophene scaffold from easily available alkynyl sulfides and aryne precursors.

**Fig. 2:**
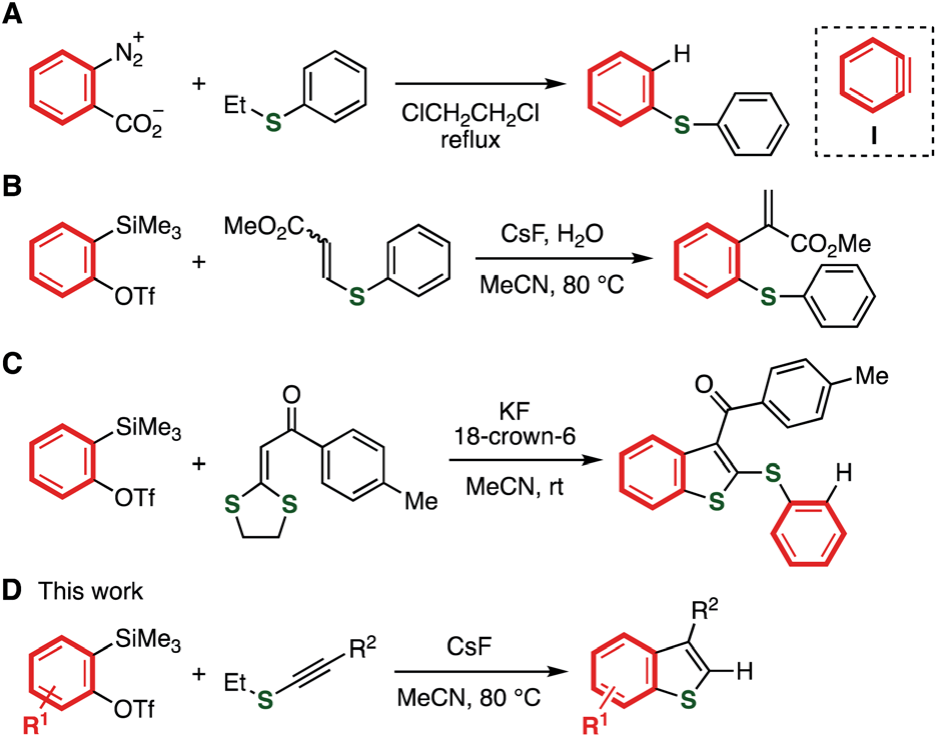
Aryne reactions with organosulfur compounds. (A) Nakayama's study. (B) Studer's work. (C) Singh's work. (D) This work.

Reactions of aryne intermediates with a variety of sulfides are attractive methods for preparing a wide range of organosulfur compounds (Fig. 2).^6–9^ In the 1980s, a pioneering study on the reaction between sulfides and benzyne intermediate (**I**) generated from benzenediazonium-2-carboxylate was reported by Nakayama and coworkers (Fig. 2A).^9*a*,9*b*^ Recently, an elegant difunctionalization of aryne intermediates was achieved by Studer and coworkers, in which C–S and C–C formations and C–S cleavage simultaneously took place (Fig. 2B).^9*h*^ Benzothiophene synthesis from *o*-silylaryl triflates and acyl-substituted ketene dithioacetals was developed by Singh and coworkers in 2016 through the formation of benzothiophene skeleton *via* aryne intermediates, and further addition with aryne intermediates (Fig. 2C).^9*o*^ On the basis of our recent studies of synthetic aryne chemistry,^10^ we envisioned that benzothiophenes can be synthesized from aryne precursors and alkynyl sulfides,^11,12^ starting from the nucleophilic attack of the sulfur or carbon of alkynyl sulfides to electrophilic aryne intermediates followed by ring-closure (Fig. 2D).

## Results and discussion

**Fig. 3:**
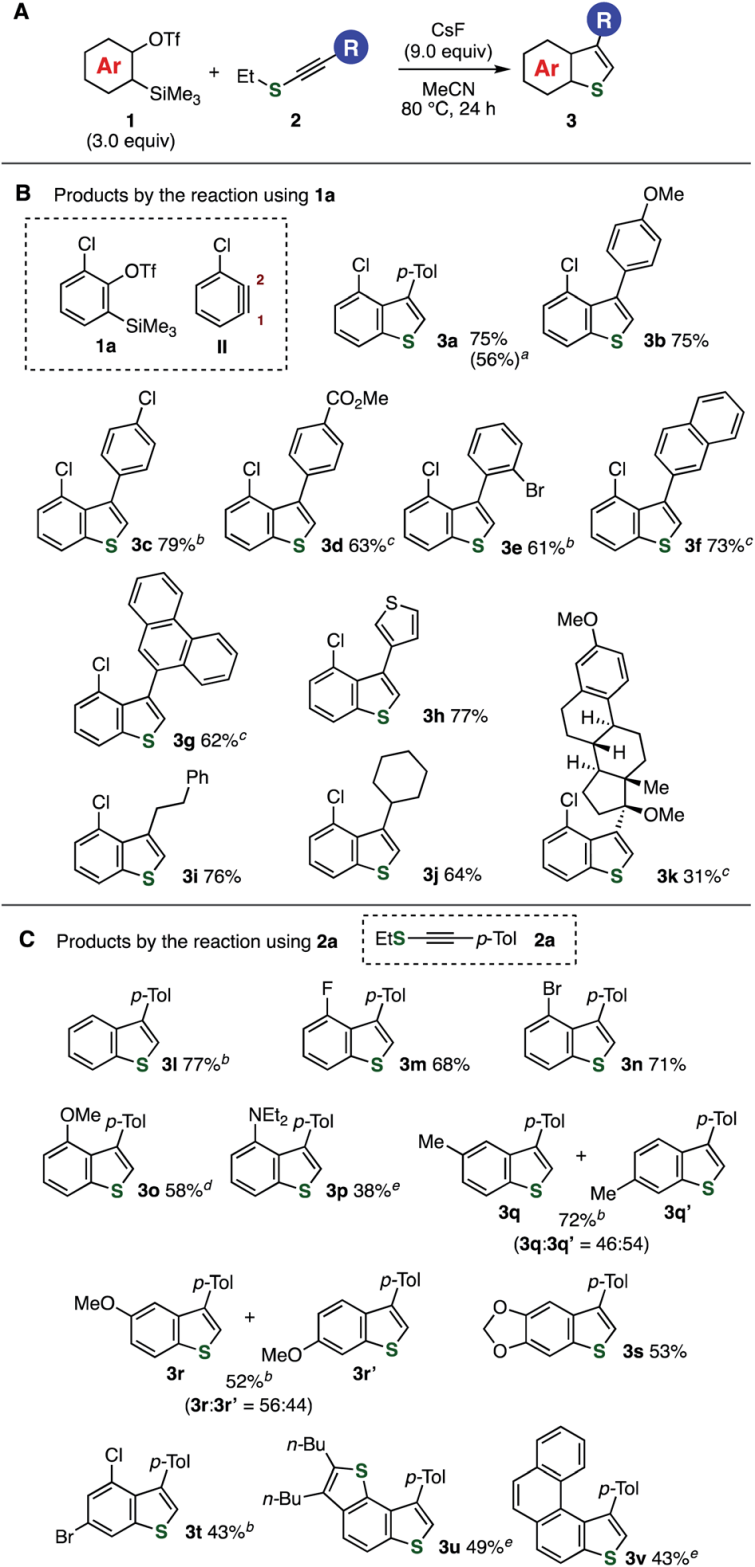
Synthesis of benzothiophenes **3** from *o*-silylaryl triflates **1** and alkynyl sulfides **2**. See, the ESI† for the structures of **1** and **2**. (A) General scheme. (B) Reactions of various alkynyl sulfides **2** with **1a**. (C) Reactions of various *o*-silylaryl triflates **1** with **2a**. ^*a*^ Isolated yield using 2.0 mmol of **2a** in parentheses. ^*b*^ The reactions were performed in 1,4-dioxane at 110 °C. ^*c*^ The reactions were performed using 5.0 equiv. of **1** and 15 equiv. of CsF. ^*d*^ The reaction was performed at rt. ^*e*^ The reactions were performed using 5.0 equiv. of **1** and 15 equiv. of CsF in 1,4-dioxane at 110 °C.

First, a reaction between 2-chloro-6-(trimethylsilyl)phenyl triflate (**1a**) and ethyl *p*-tolylethynyl sulfide (**2a**) was examined (Fig. 3A and B). As a result, we found that treatment of a mixture between aryne precursor **1a** and alkynyl sulfide **2a** with cesium fluoride in hot acetonitrile provided 3-(4-tolyl)-4-chlorobenzo[*b*]thiophene (**3a**) in high yield. The construction of benzothiophene scaffold was accomplished by C–S bond formation selectively at C1 of 3-chlorobenzyne (**II**), C–C bond formation, protonation, and deethylation, where the regioisomer was not detected. When the reaction was conducted on a larger scale using 2 mmol of alkynyl sulfide **2a**, the yield of benzothiophene **3a** was slightly decreased. Increasing concentration from 0.05 M to 0.2 or 0.5 M slightly reduced the yield of **3a** (64% or 52%, respectively).^13^ Benzothiophene **3a** was also obtained in moderate to good yields even when the amount of aryne precursor **1a** was decreased from 3.0 equiv. to 2.0, 1.5, and 1.2 equiv.^13^ These results show good practicality of the benzothiophene synthesis from *o*-silylaryl triflates **1** and alkynyl sulfides **2**. The reaction of aryne precursor **1a** with methyl, isopropyl, or benzyl *p*-tolylethynyl sulfides instead of **2a** also afforded benzothiophene **3a**, although **3a** was not detected in the case of *p*-tolyl *p*-tolylethynyl sulfide.^13^

A broad range of 3-aryl- and 3-alkyl-substituted benzo[*b*]thiophenes were prepared from aryne precursor **1a** and various alkynyl sulfides **2** (Fig. 3A and B). For example, electron-donating methoxy- and electron-withdrawing chloro- and methoxycarbonyl-substituted arylethynyl ethyl sulfides smoothly reacted with aryne intermediates to afford benzo[*b*]thiophenes **3b–d** without damaging these functional groups. Bulky 2-bromophenylethynyl ethyl sulfide also participated in the reaction providing **3e** in good yield. Furthermore, benzothiophenes **3f** and **3g** having π-extended aromatics and **3h** possessing heteroaromatic thiophene ring were synthesized efficiently from the corresponding alkynyl sulfides. The reaction of primary and secondary alkylethynyl ethyl sulfides with aryne intermediates also proceeded under the same conditions to afford **3i** and **3j** in high yields. Moreover, we succeeded in the synthesis of benzothiophene **3k** from alkynyl sulfide **2k** prepared from an ethynylestradiol derivative. Since a wide variety of alkynyl sulfides were easily available from the corresponding terminal alkynes and thiosulfonates catalyzed by copper as we recently reported,^12*e*^ this method enables the synthesis of diverse 3-substituted benzothiophenes.

Diverse aryne precursors were applicable to the one-step benzothiophene synthesis enabling to prepare a variety of benzothiophenes **3l–v** (Fig. 3A and C). Not only simple benzyne but also 3-fluoro-, 3-bromo, 3-methoxy, and 3-aminobenzyne intermediates reacted with alkynyl sulfides **2a** to furnish **3l–p** in moderate to good yields leaving these functional groups untouched, in which regioisomers were not detected. Especially, selective C–S bond formation proceeded in the reaction of aryne intermediates bearing functional groups at 3-position, showing that the benzothiophene formation triggered by the nucleophilic attack of the sulfur atom onto C1 of the 3-substituted aryne intermediates due to the inductive effect of fluorine, bromine, oxygen, and nitrogen.^14^ Reactions of 4-methoxy- and 4-methylbenzyne with alkynyl sulfide **2a** furnished *ca.* 1 : 1 mixtures of regioisomers of benzothiophenes **3q** and **3r** in moderate to high yields. Trisubstituted benzothiophenes **3s** and **3t** were also synthesized from the corresponding *o*-silylaryl triflates. It is worthy to note that the synthesis of π-conjugated benzothiophenes **3u** and **3v** was accomplished from the corresponding benzothiophene- and phenanthrene-type *o*-silylaryl triflates.^10*c*^ The broad scope of the synthesizable benzothiophenes clearly demonstrated a benefit of this method by virtue of the recent remarkable advancement of the accessibility of *o*-silylaryl triflates and predictable reactivity of aryne intermediates by the aryne distortion model.^14^

**Fig. 4:**
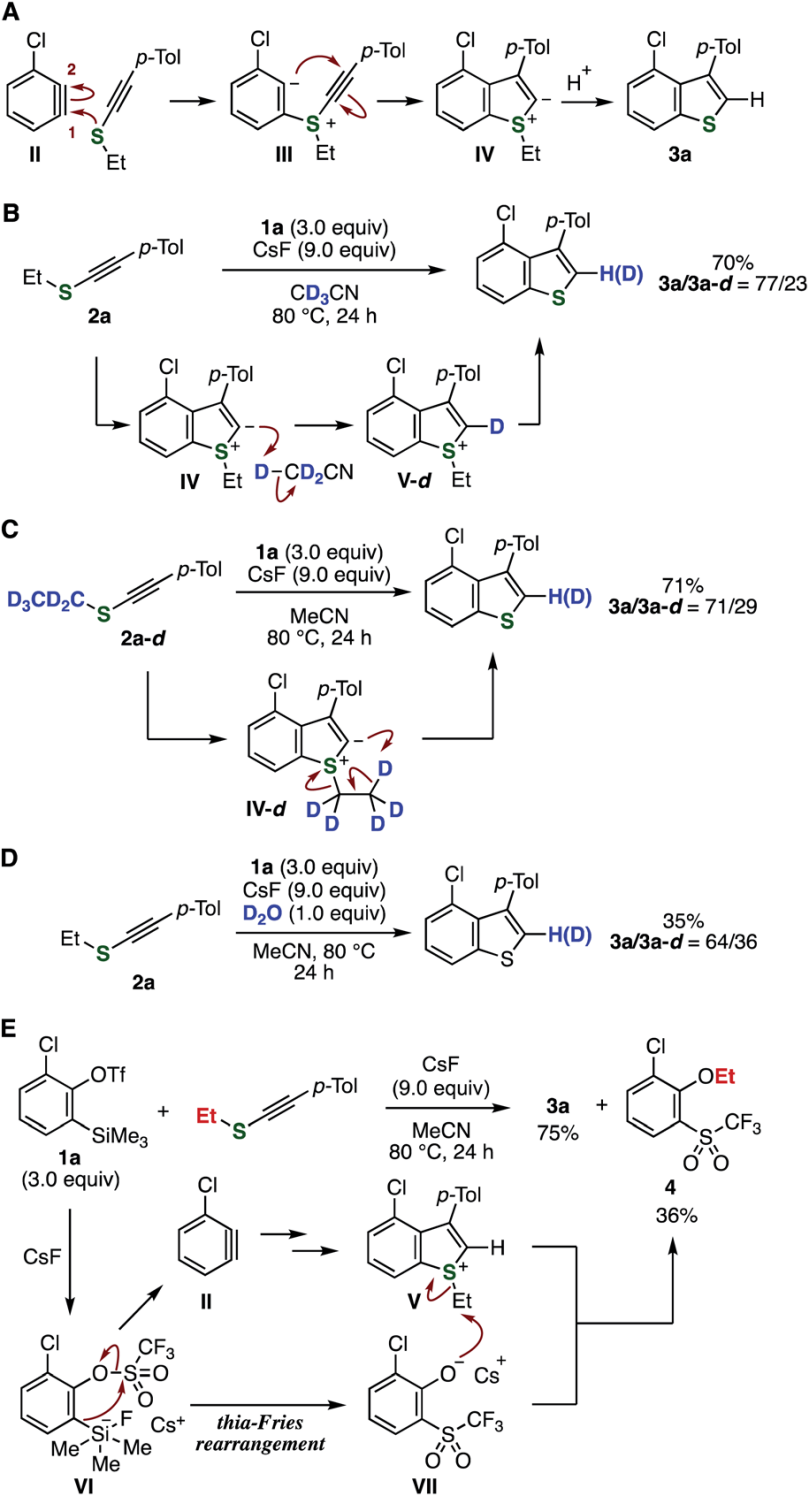
Mechanistic studies. (A) Plausible reaction pathway. (B) Deuteration experiment using CD_3_CN. (C) Deuteration experiment using **2a-d**. (D) Deuteration experiment in the presence of D_2_O. (E) Mechanistic insights from side-product **4**.

A plausible reaction mechanism is shown in Fig. 4A. First, the nucleophilic addition of the sulfur atom of alkynyl sulfides onto arynes and following cyclization to the alkyne carbon construct the benzothiophene skeleton. Then, protonation of the resulting zwitterionic intermediate **IV** leads to benzothiophene **3a**. To examine the proton source, we then performed control experiments using deuterated compounds (Fig. 4B–D). Treatment of *o*-silylaryl triflate **1a** and alkynyl sulfide **2a** dissolved in CD_3_CN with cesium fluoride provided benzothiophene **3a** with partial incorporation of deuterium through sulfonium intermediate **V-d** (Fig. 4B).^9*b*,15^ The reaction using deuterium-labeled ethyl *p*-tolylethynyl sulfide **2a-d** in acetonitrile also resulted in partial deuterium incorporation, suggesting intramolecular deuteration of zwitterionic intermediate **IV-d** with liberating ethylene (Fig. 4C).^9*m*^ An alternative proton source would be water in the reagents used, since deuterium was incorporated when the reaction was performed in the presence of deuterium oxide (Fig. 4D). Moreover, 2-chloro-6-(trifluoromethanesulfonyl)phenyl ethyl ether (**4**) was detected as a side-product in the synthesis of **3a**, clearly showing that 2-chloro-6-(trifluoromethanesulfonyl)phenolate intermediate **VII** was generated from **1a***via* the thia-Fries rearrangement of silicate intermediate **VI**,^16^ and cesium phenolate **VII** was involved in the deethylation process of sulfonium intermediate **V** (Fig. 4E). Thus, possible protonation and deethylation mechanisms from zwitterionic intermediate **IV** were supported by these results.

**Fig. 5:**
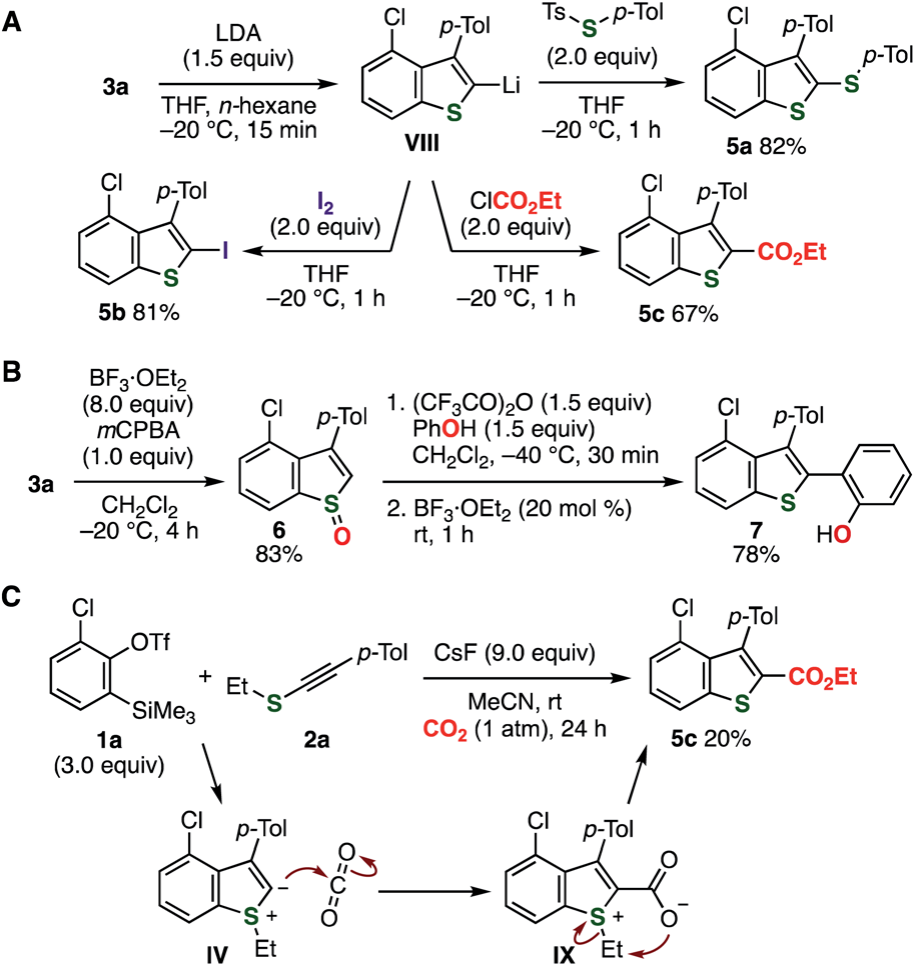
C2-functionalizations of benzothiophene **3a**. (A) Transformations through C2-deprotonation. (B) Arylation *via* Pummerer-type activation. (C) Direct ester formation.

Various C2-functionalizations of benzothiophene **3a** allowed for the preparation of a wide range of 2,3,4-trisubstituted benzothiophenes (Fig. 5).^17^ For example, selective deprotonation of **3a** with lithium diisopropylamide (LDA) proceeded efficiently (Fig. 5A). Sulfanylation, iodination, and ethoxycarbonylation of the resulting 2-benzothiophenyllithium **VIII** provided benzothiophenes **5a–c** in good yields. Furthermore, *S*-oxidation followed by the Pummerer-type C2-arylation with phenol through [3,3]-sigmatropic rearrangement selectively afforded benzothiophene **7** (Fig. 5B).^17*h*^ Additionally, treatment of *o*-silylaryl triflate **1a** and alkynyl sulfide **2a** with cesium fluoride under carbon dioxide furnished benzothiophene **5c** having an ester moiety albeit in low yield, where C–C bond formation of zwitterionic intermediate **IV** and subsequent migration of the ethyl group occurred (Fig. 5C).

**Fig. 6:**
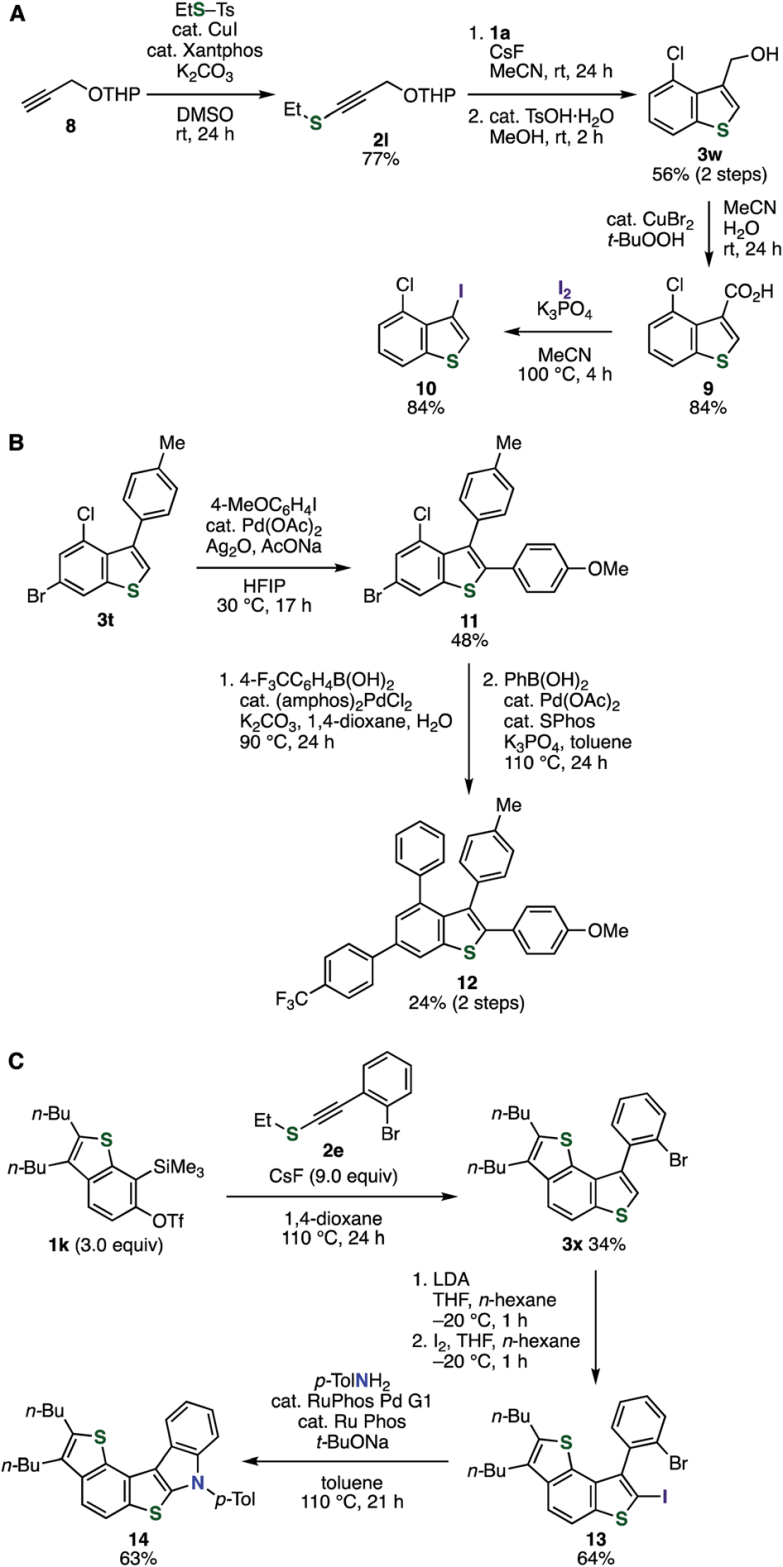
Applications of the benzothiophene synthesis. (A) Benzothiophene synthesis through C3-functionalizations. (B) Synthesis of tetraarylbenzothiophene **12**. (C) Synthesis of pentacyclic compound **14**.

We succeeded in the preparation of C3-functionalized benzothiophene **10** from alkyne **8** and aryne precursor **1a** through C–C cleavage of carboxylic acid **9** by virtue of the good accessibility of alkynyl sulfides and broad substrate scope of the aryne reaction (Fig. 6A). Indeed, latently transformable alkynyl sulfide **2l** was synthesized from terminal alkyne **8** and *S*-ethyl *p*-toluenethiosulfonate catalyzed by CuI/xantphos under mild conditions.^12*e*^ Following aryne reaction between **2l** and *o*-silylaryl triflate **1a** and subsequent removal of the tetrahydropyranyl (THP) group successfully afforded benzothiophene **3w** having chloro and hydroxymethyl groups. Then, carboxylic acid **9** was prepared by oxidation with *tert*-butyl hydroperoxide catalyzed by copper(ii) bromide.^18^ Considering that recent remarkable achievements for transformations of the carboxy group into a range of functional groups such as halogens, phosphorus moieties, and aryl groups through C–C cleavage,^19^ diverse C3-functionalized benzothiophenes will be synthesized from benzothiophene **9**. For example, decarboxylative iodination of **9** took place smoothly to provide 4-chloro-3-iodobenzo[*b*]thiophene (**10**) in high yield.^19*b*^ Thus, a wide variety of benzothiophenes can be synthesized through the aryne reaction between alkynyl sulfide **2l** and *o*-silylaryl triflates and decarboxylative transformations.

We achieved the synthesis of benzothiophene **12** bearing four different aryl groups by virtue of the halogen-tolerated benzothiophene synthesis and the versatility of C2-position (Fig. 6B). Indeed, direct C–H arylation of benzothiophene **3t** with 4-iodoanisole proceeded smoothly to provide **11** in moderate yield keeping bromo and chloro groups intact.^17*i*^ Then, a sequential Suzuki–Miyaura cross-coupling of **11** with 4-(trifluoromethyl)phenyboronic acid and phenylboronic acid at the bromo and chloro group, respectively, successfully furnished 2,3,4,6-tetraarylbenzothiophene **12**. This modular synthetic route would enable the preparation of diverse multi-arylated benzothiophenes using various alkynyl sulfides, aryl iodides, and arylboronic acids.^20^

The good accessibility of *o*-silylaryl triflates and alkynyl sulfides realized the synthesis of polycyclic aromatic compound **14** (Fig. 6C). Firstly, the treatment of 6,7-thienobenzyne precursor **1k** and alkynyl sulfide **2e** with cesium fluoride afforded dithienobenzene **3x** in moderate yield. Then, C2-iodination was realized by deprotonation with LDA followed by the addition of iodine. Finally, palladium-catalyzed amination at C2 of benzothiophene with *p*-toluidine and subsequent cyclization proceeded efficiently to afford pentahelicene analog **14**. This result clearly demonstrated an advantage of the benzothiophene synthesis by the aryne reaction with alkynyl sulfides enabling to prepare π-extended benzothiophenes having functional groups such as halogens. The benzothiophene synthesis will serve in the synthesis of various polyaromatic analogs containing benzothiophene skeleton.^21^

## Conclusions

In summary, we have developed a facile one-step synthetic method of benzothiophenes from *o*-silylaryl triflates and alkynyl sulfides. The wide scope of the benzothiophene synthesis and the versatile C2-functionalizations enabled the synthesis of a variety of multisubstituted benzothiophenes, which is difficult by the conventional methods. Further studies to clarify the reaction mechanism and to expand synthesizable multisubstituted benzothiophenes involving three-component couplings, and applications to synthesize analogs of bioactive compounds are currently underway.

## Conflicts of interest

There are no conflicts to declare.

## Supplementary Material

SC-011-D0SC04450D-s001
