# Supplementary material for: One-step synthesis of benzo[b]thiophenes by aryne reaction with alkynyl sulfides
Source: Chem Sci. 2020 Sep 1;11(35):9691–6. doi: 10.1039/d0sc04450d (PMC8162113; doi:10.1039/d0sc04450d)

Electronic Supplementary Material (ESI) for Chemical Science.
This journal is © The Royal Society of Chemistry 2020

Supporting Information

One-step synthesis of benzo[*b*]thiophenes by aryne reaction with alkynyl sulfides

Tsubasa Matsuzawa, Takamitsu Hosoya, and Suguru Yoshida*

*Laboratory of Chemical Bioscience, Institute of Biomaterials and Bioengineering,
Tokyo Medical and Dental University (TMDU),
2-3-10 Kanda-Surugadai, Chiyoda-ku, Tokyo 101-0062, Japan*

Contents

General Information S1
Structures of Aryne precursors 1 and Alkynyl sulfides 2 S2
Optimization of Reaction Conditions S3
Experimental Procedures S7
Deuteration Experiments S15
Characterization Data of New Compounds S17
References for Supporting Information S31
^{1}H and ^{13}C NMR Spectra of Compounds S32

General Information

All reactions were performed with dry glassware under atmosphere of argon, unless otherwise noted. Analytical thin-layer chromatography (TLC) was performed on precoated (0.25 mm) silica-gel plates (Merck Chemicals, Silica Gel 60 F_{254}, Cat. No. 1.05715). Column chromatography was conducted using silica-gel (Kanto Chemical Co., Inc., Silica Gel 60, spherical, particle size 40–50 μm, Cat. No. 37562-85). Preparative thin-layer chromatography (PTLC) was performed on silica-gel (Wako Pure Chemical Industries Ltd., Wakogel® B-5F, Cat. No. 230-00043). Recycling preparative HPLC was conducted using a YMC-GPC T2000 (600 mm × 20 φ) column (YMC Co., Ltd.) with a recycling preparative HPLC system (SHIMADZU, eluent: $CHCl_3$). Melting points (Mp) were measured on an OptiMelt MPA100 (Stanford Research Systems), and are uncorrected. ^{1}H NMR spectra were obtained with a Bruker AVANCE 500 spectrometer at 500 MHz, or a Bruker AVANCE 400 spectrometer at 400 MHz. ^{13}C NMR spectra were obtained with a Bruker AVANCE 500 spectrometer at 126 MHz, or a Bruker AVANCE 400 spectrometer at 101 MHz. ^{19}F NMR spectra were obtained with a Bruker AVANCE 400 spectrometer at 376 MHz. All NMR measurements were carried out at 25 ºC. $CDCl_3$ (Kanto Chemical Co. Inc., Cat. No. 07663-23) and DMSO-d_6 (Kanto Chemical Co. Inc., Cat. No. 11560-43) were used as a solvent for obtaining NMR spectra. Chemical shifts (δ) are given in parts per million (ppm) downfield from the solvent peak (δ 7.26 for ^{1}H NMR and δ 77.2 for ^{13}C NMR in $CDCl_3$, and δ 2.50 for ^{1}H NMR in DMSO-d_6) as an internal reference or α,α,α-trifluorotoluene (δ −63.0 ppm for ^{19}F NMR in $CDCl_3$) as an external standard with coupling constants (*J*) in hertz (Hz). The abbreviations s, d, t, q, sept, and m, signify singlet, doublet, triplet, quartet, septet, and multiplet, respectively. IR spectra were measured by diffuse reflectance method on a Shimadzu IRPrestige-21 spectrometer attached with DRS-8000A with the absorption band given in cm^{-1}. High-resolution mass spectra (HRMS) were measured on a Bruker micrOTOF mass spectrometer under positive electrospray ionization (ESI^{+}) conditions.

S-Methyl 4-toluenethiosulfonate,[S1] *S*-ethyl 4-toluenethiosulfonate,[S2] *S*-isopropyl 4-toluenethiosulfonate,[S1] *S*-benzyl 4-toluenethiosulfonate,[S3] *S*-(4-tolyl) 4-toluenethiosulfonate,[S1] 3,17-di-*O*-methylethinylestradiol,[S4] 2-fluoro-6-(trimethylsilyl)phenyl triflate (**1c**),[S5] 3-(diethylamino)-2-(trimethylsilyl)phenyl triflate (**1f**),[S6] 4-bromo-2-chloro-6-(trimethylsilyl)phenyl triflate (**1h**),[S7] and 2,3-dibutyl-6-triflyloxy-7-(trimethylsilyl)benzo[*b*]thiophene (**1i**)[S8] were prepared according to the reported methods. *n*-BuLi (1.65 M, in *n*-hexane), and LDA (1.0 M, in THF) were used after titrimetric determination of the concentration by the 1,10-phenanthroline method.[S9] All other chemical reagents used were commercial grade and used as received.

Structures of Aryne precursors 1 and Alkynyl sulfides 2

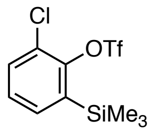
1a

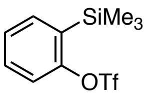
1b

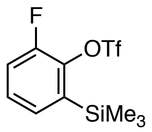
1c

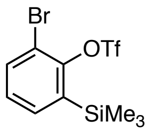
1d

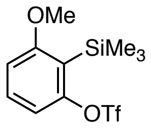
1e

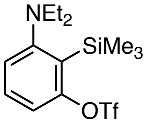
1f

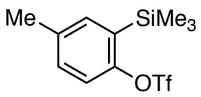
1g

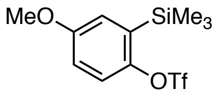
1h

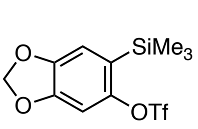
1i

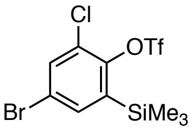
1j

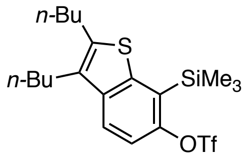
1k

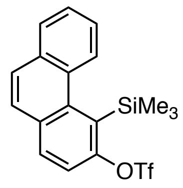
1l

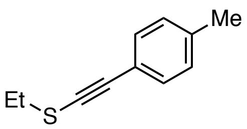
2a

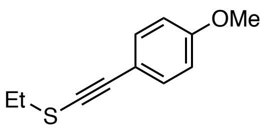
2b

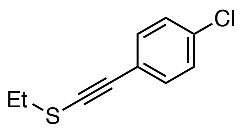
2c

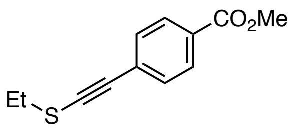
2d

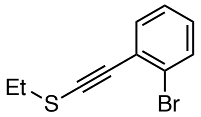
2e

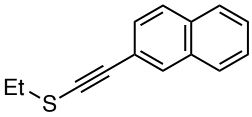
2f

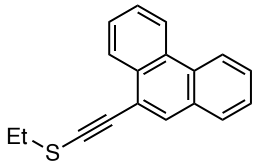
2g

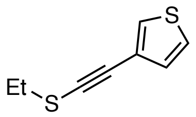
2h

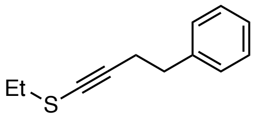
2i

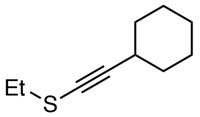
2j

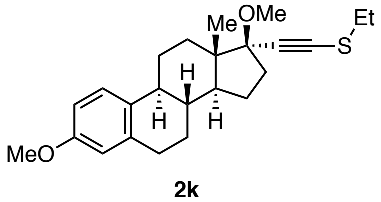
2k

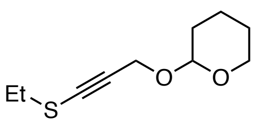
2l

Optimization of Reaction Conditions

Table S1. Reactions of aryne precursor 1a with various alkynyl sulfides

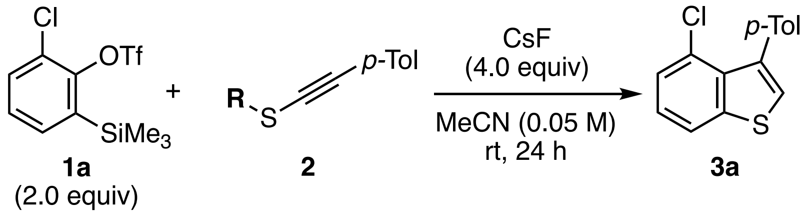

entry	**R**	**3a** : yield[a]
1	Me	65%
2	Et	70%
3	*i*-Pr	48%
4	Bn	33%
5	*p*-Tol	0%

[a] Yields based on ^{1}H NMR analyses.

Table S2. Optimization of activator

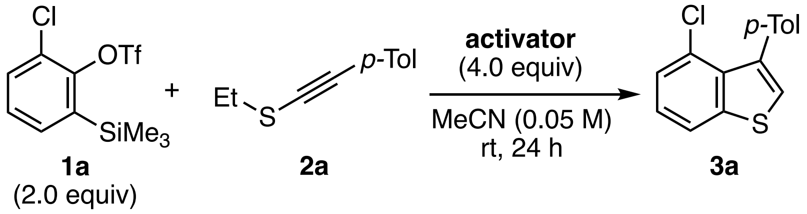

entry	**activator**	**3a** : yield[a]
1	CsF	70%
2	KF, 18-crown-6	61%
3	n-Bu_4NF	0%
4	n-$Bu_4NF\cdot 3H_2O$	18%
5	n-$Bu_4N[Ph_3SiF_2]$	28%
6	Cs_2CO_3, 18-crown-6	32%
7	K_2CO_3, 18-crown-6	38%

[a] Yields based on ^{1}H NMR analyses.

Table S3. Optimization of solvent

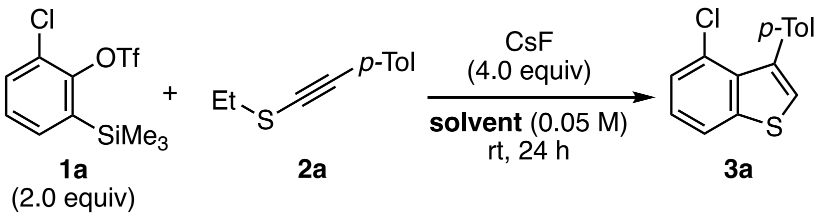

entry	**solvent**	**3a** : yield[a]
1	MeCN	70%
2	THF	40%
3	DME	57%
4	acetone	38%
5	$MeNO_2$	31%
6	PhCN	32%

[a] Yields based on ^{1}H NMR analyses.

Table S4. Optimization of concentration

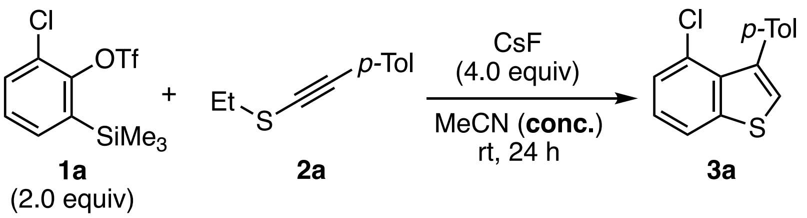

entry	**conc.**	**3a** : yield[a]
1	0.05 M	70%
2	0.025 M	68%
3	0.1 M	64%
4	0.2 M	64%
5	0.5 M	52%

[a] Yields based on ^{1}H NMR analyses.

Table S5. Optimization of temperature

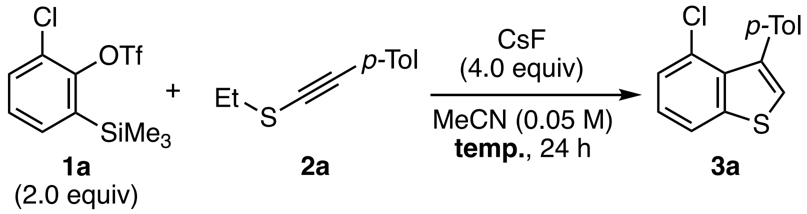

entry	**temp.**	**3a** : yield[a]
1	rt	70%
2	80 °C	72%
3	100 °C	62%

[a] Yields based on ^{1}H NMR analyses.

Table S6. Optimization of the amount of aryne precursor 1a and cesium fluoride

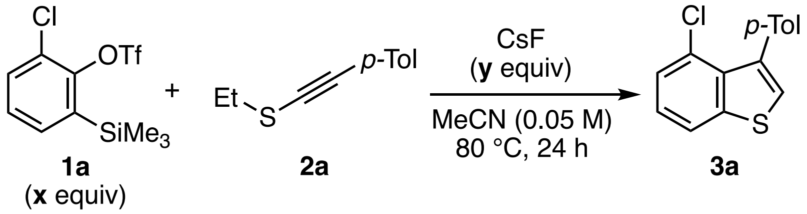

entry	**x**	**y**	**3a** : yield[a]
1	2.0	4.0	72%
2	1.5	3.0	59%
3	1.2	2.4	45%
4	3.0	6.0	77%
5	3.0	9.0	79%(75%)[b]

[a] Yields based on ^{1}H NMR analyses, unless otherwise noted.
[b] Isolated yield.

Table S7. Optimization of quencher

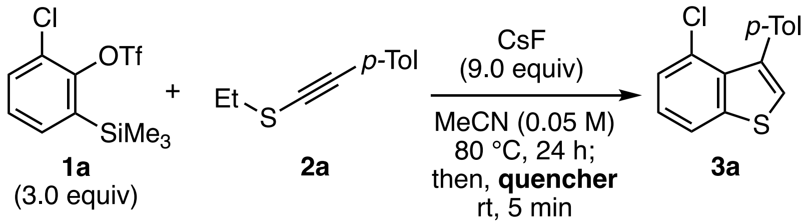

1a (3.0 equiv) **2a** **3a**

entry	**quencher**	**3a** : yield[a]
1	H_2O	79%
2	Et_3N	80%
3	none	80%

[a] Yields based on ^{1}H NMR analyses.

Experimental Procedures

A typical procedure for the preparation of alkynyl sulfides[S10]

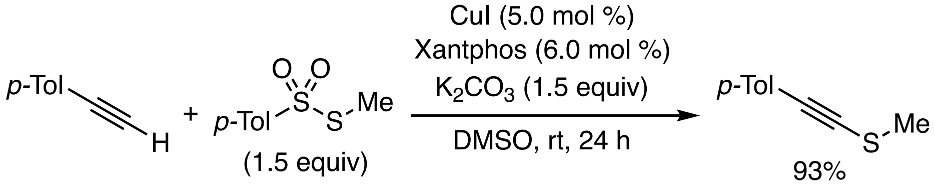

A mixture of 4-ethynyltoluene (581 mg, 5.00 mmol), *S*-methyl 4-toluenethiosulfonate (1.52 g, 7.50 mmol, 1.5 equiv), CuI (47.6 mg, 0.250 mmol, 5.0 mol %), Xantphos (173.6 mg, 0.300 mmol, 6.0 mol %), and K_2CO_3 (1.04 g, 7.50 mmol, 1.5 equiv) suspended in DMSO (30 mL) was stirred at room temperature. After stirring for 24 h at the same temperature, to the mixture was added an aqueous saturated potassium carbonate solution (30 mL). The mixture was extracted with EtOAc (30 mL × 3), and the combined organic extract was washed with aqueous saturated potassium carbonate solution (20 mL × 3) and brine (20 mL), dried (Na_2SO_4), and after filtration, the filtrate was concentrated under reduced pressure. The residue was purified by flash column chromatography (silica-gel 20 g, *n*-hexane) to give methyl (4-tolyl)ethynyl sulfide (752 mg, 4.64 mmol, 93%) as a colorless oil.

According to the procedure for preparing methyl (4-tolyl)ethynyl sulfide, ethyl (4-tolyl)ethynyl sulfide (**2a**), isopropyl (4-tolyl)ethynyl sulfide, ethyl (4-methoxyphenyl)ethynyl sulfide (**2b**), (4-chlorophenyl)ethynyl ethyl sulfide (**2c**), ethyl (4-methoxycarbonylphenyl)ethynyl sulfide (**2d**), (2-bromophenyl)ethynyl ethyl sulfide (**2e**), ethyl (2-naphthyl)ethynyl sulfide (**2f**), ethyl (9-phenanthrenyl)ethynyl sulfide (**2g**), ethyl (3-thienyl)ethynyl sulfide (**2h**), and 2-((3-(ethylthio)prop-2-yn-1-yl)oxy)tetrahydro-2*H*-pyran (**2l**) were prepared from the corresponding thiosulfonates and terminal alkynes.

Preparation of benzyl (4-tolyl)ethynyl sulfide

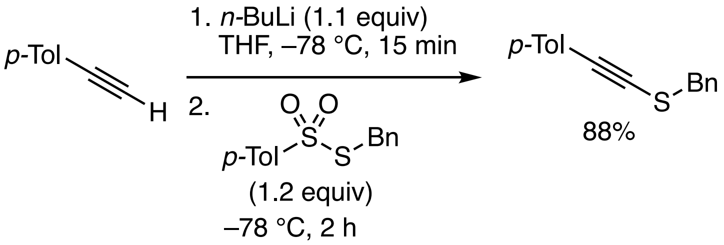

To a solution of 4-ethynyltoluene (232 mg, 2.00 mmol) dissolved in THF (5.0 mL) was slowly added *n*-BuLi (1.65 M, hexane solution, 1.33 mL, 2.20 mmol, 1.1 equiv) at –78 ºC. After stirring for 15 minutes at the same temperature, to the mixture was slowly added a solution of *S*-benzyl 4-toluenethiosulfonate (668 mg, 2.40 mmol, 1.2 equiv) dissolved in THF (5.0 mL) at –78 °C. After stirring for 2 h at the same temperature, the mixture was allowed to warm to room temperature, and to this was added an aqueous saturated ammonium chloride solution (10 mL). The mixture was extracted with EtOAc (20 mL × 3), and the combined organic extract was washed with brine (10 mL), dried (Na_2SO_4), and after filtration, the filtrate was concentrated under reduced pressure. The residue was purified by flash column chromatography (silica-gel 10 g, *n*-hexane/CH_2Cl_2 = 10/1) to give benzyl (4-tolyl)ethynyl sulfide (418 mg, 1.75 mmol, 87.7%) as a pale red oil.

According to the procedure for preparing benzyl (4-tolyl)ethynyl sulfide, 4-tolyl (4-tolyl)ethynyl sulfide, ethyl (2-phenethyl)ethynyl sulfide (**2i**), cyclohexylethynyl ethyl sulfide (**2j**), and 3,17-di-*O*-methyl-17-(ethylthioethynyl)estradiol (**2k**) were prepared from the corresponding thiosulfonates and terminal alkynes.

A typical procedure for the synthesis of benzothiophenes by reaction of arynes with alkynyl sulfides

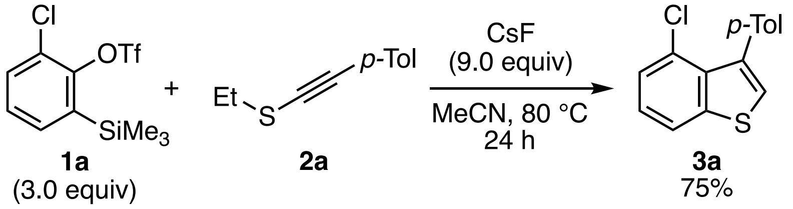

To a mixture of 2-chloro-6-(trimethylsilyl)phenyl triflate (**1a**) (99.8 mg, 0.300 mmol, 3.0 equiv) and ethyl (4-tolyl)ethynyl sulfide (**2a**) (19.2 mg, 0.100 mmol) dissolved in MeCN (2.0 mL) were added cesium fluoride (137 mg, 0.900 mmol, 9.0 equiv) at room temperature. After stirring for 24 h at 80 °C, the mixture was cooled to room temperature, and to this was added water (3 mL). The mixture was extracted with EtOAc (10 mL × 3), and the combined organic extract was washed with brine (5 mL), dried (Na_2SO_4), and after filtration, the filtrate was concentrated under reduced pressure. The residue was purified by preparative TLC (*n*-hexane) to give 4-chloro-3-(4-tolyl)benzo[*b*]thiophene (**3a**) (20.6 mg, 74.9 µmol, 75%) as a colorless oil.

According to the procedure for preparing 4-chloro-3-(4-tolyl)benzo[*b*]thiophene (**3a**), 4-chloro-3-(4-methoxyphenyl)benzo[*b*]thiophene (**3b**), 4-chloro-3-(4-chlorophenyl)benzo[*b*]thiophene (**3c**), 4-chloro-3-(4-methoxycarbonylphenyl)benzo[*b*]thiophene (**3d**), 3-(2-bromophenyl)-4-chlorobenzo[*b*]thiophene (**3e**), 4-chloro-3-(2-naphthyl)benzo[*b*]thiophene (**3f**), 4-chloro-3-(9-phenanthrenyl)benzo[*b*]thiophene (**3g**), 4-chloro-3-(3-thienyl)benzo[*b*]thiophene (**3h**), 4-chloro-3-(2-phenylethyl)benzo[*b*]thiophene (**3i**), 4-chloro-3-cyclohexylbenzo[*b*]thiophene (**3j**), 4-chloro-3-(3,17-di-*O*-methylestradiol-17-yl)benzo[*b*]thiophene (**3k**), 3-(4-tolyl)benzo[*b*]thiophene (**3l**), 4-fluoro-3-(4-tolyl)benzo[*b*]thiophene (**3m**), 4-bromo-3-(4-tolyl)benzo[*b*]thiophene (**3n**), 4-methoxy-3-(4-tolyl)benzo[*b*]thiophene (**3o**), 4-diethylamino-3-(4-tolyl)benzo[*b*]thiophene (**3p**), 5-methyl-3-(4-tolyl)benzo[*b*]thiophene (**3q**), 6-methyl-3-(4-tolyl)benzo[*b*]thiophene (**3q'**), 5-methoxy-3-(4-tolyl)benzo[*b*]thiophene (**3r**), 6-methoxy-3-(4-tolyl)benzo[*b*]thiophene (**3r'**), 5,6-(methylenedioxy)-3-(4-tolyl)benzo[*b*]thiophene (**3s**), 6-bromo-4-chloro-3-(4-tolyl)benzo[*b*]thiophene (**3t**), 2,3-dibutyl-8-(4-tolyl)benzo[1,2-*b*:3,4-*b'*]dithiophene (**3u**), 1-(4-tolyl)phenanthro[3,4-*b*]thiophene (**3v**), and 8-(2-bromophenyl)-2,3-(dibutyl)benzo[1,2-*b*:3,4-*b'*]dithiophene (**3x**) were prepared from the corresponding aryne precursors and alkynyl sulfides.

*Procedure for the synthesis of benzothiophene **3a** using 2.0 mmol of alkynyl sulfide **2a***

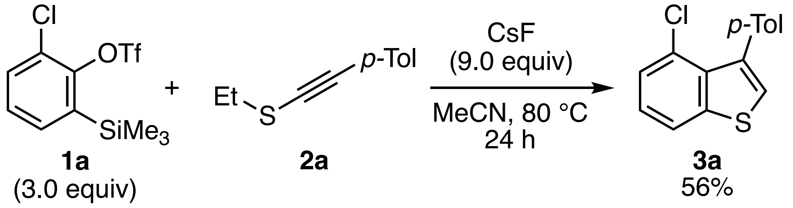

To a mixture of 2-chloro-6-(trimethylsilyl)phenyl triflate (**1a**) (2.00 g, 6.00 mmol, 3.0 equiv) and ethyl (4-tolyl)ethynyl sulfide (**2a**) (353 mg, 2.00 mmol) dissolved in MeCN (20 mL) were added cesium fluoride (2.73 g, 18.0 mmol, 9.0 equiv) at room temperature. After stirring for 24 h at 80 °C, the mixture was cooled to room temperature, and to this was added water (30 mL). The mixture was extracted with EtOAc (30 mL × 3), and the combined organic extract was washed with brine (10 mL), dried (Na_2SO_4), and after filtration, the filtrate was concentrated under reduced pressure. The residue was purified by by flash column chromatography (silica-gel 26 g, *n*-hexane) to give 4-chloro-3-(4-tolyl)benzo[*b*]thiophene (**3a**) (292 mg, 1.13 mmol, 56%) as a colorless oil.

*A typical procedure for the functionalization of benzothiophene **3a** via deprotonation at the C2 position*

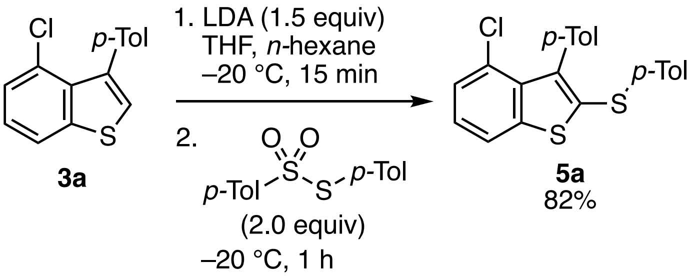

To a solution of 4-chloro-3-(4-tolyl)benzo[*b*]thiophene (**3a**) (25.4 mg, 98.2 μmol) dissolved in THF (500 μL) was slowly added LDA (1.00 M, THF/*n*-hexane solution, 150 μL, 0.150 mmol, 1.5 equiv) at –20 °C. After stirring for 15 min at the same temperature, to the mixture was slowly added a solution of *S*-(4-tolyl) 4-toluenethiosulfonate (55.7 mg, 0.200 mmol, 2.0 equiv) dissolved in THF (500 μL) at –20 °C. After stirring for 1 h at the same temperature, the mixture was allowed to warm to room temperature, and to this was added an aqueous saturated ammonium chloride solution (5 mL). The mixture was extracted with EtOAc (10 mL × 3), and the combined organic extract was washed with brine (5 mL), dried (Na_2SO_4), and after filtration, the filtrate was concentrated under reduced pressure. The residue was purified by preparative TLC (*n*-hexane/CH_2Cl_2 = 5/1) to give 4-chloro-3-(4-tolyl)-2-(4-tolylthio)benzo[*b*]thiophene (**5a**) (30.8 mg, 80.9 μmol, 82%) as a colorless oil.

Similarly, 4-chloro-2-iodo-3-(4-tolyl)benzo[*b*]thiophene (**5b**) and 4-chloro-2-ethoxycarbonyl-3-(4-tolyl)benzo[*b*]thiophene (**5c**) were prepared.

*Procedure for the synthesis of 4-chloro-3-(4-tolyl)benzo[b]thiophene S-oxide (**6**)*[S11]

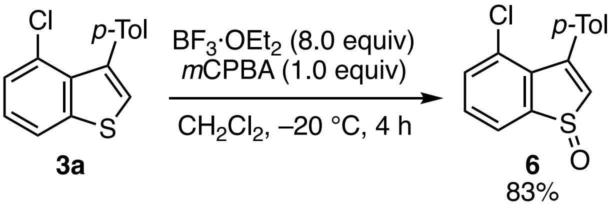

To a solution of 4-chloro-3-(4-tolyl)benzo[*b*]thiophene (**3a**) (38.8 mg, 0.150 mmol) dissolved in CH_2Cl_2 (1.0 mL) was added $BF_3·OEt_2$ (151 μL, 1.20 mmol, 8.0 equiv) at –20 °C. To the mixture was slowly added *m*CPBA (ca. 65%, 39.8 mg, ca. 0.15 mmol, ca. 1.0 equiv) over 1.5 h at the same temperature. After stirring for 4 h at the same temperature, to this were added an aqueous saturated solution of sodium bicarbonate (5 mL) and an aqueous saturated solution of sodium thiosulfate (5 mL). The mixture was extracted with CH_2Cl_2 (10 mL × 3), and the combined organic extract was washed with brine (5 mL), dried (Na_2SO_4), and after filtration, the filtrate was concentrated under reduced pressure. The residue was purified by preparative TLC (*n*-hexane/EtOAc = 1/1) to give 4-chloro-3-(4-tolyl)benzo[*b*]thiophene *S*-oxide (**6**) (34.1 mg, 0.124 mmol, 83%) as a colorless solid.

*Procedure for the synthesis of benzothiophene 7 by C2 C–H arylation of benzothiophene S-oxide **6**[S11]*

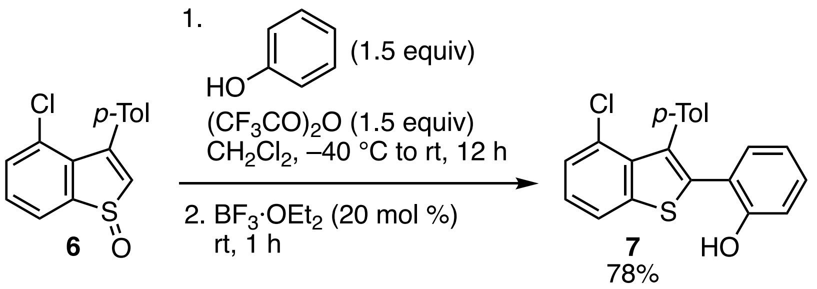

To a mixture of 4-chloro-3-(4-tolyl)benzo[*b*]thiophene *S*-oxide (**6**) (10.7 mg, 38.9 µmol) and phenol (5.50 mg, 58.4 µmol, 1.5 equiv) dissolved in CH_2Cl_2 (500 µL) was added trifluoroacetic anhydride (8.23 µL, 58.4 µmol, 1.5 equiv) at –40 °C. After 15 min at the same temperature, the mixture was warmed to room temperature. After stirring for 12 h at room temperature, to this were added $BF_3·OEt_2$ (0.98 µL, 7.8 µmol, 20 mol %) at room temperature. After stirring for 1 h at the same temperature, to the mixture was added water (3 mL). The mixture was extracted with CH_2Cl_2 (10 mL × 3), and the combined organic extract was washed with brine (5 mL), dried (Na_2SO_4), and after filtration, the filtrate was concentrated under reduced pressure. The residue was purified by preparative TLC (*n*-hexane/EtOAc = 9/1) to give 4-chloro-2-(2-hydroxyphenyl)-3-(4-tolyl)benzo[*b*]thiophene (**7**) (11 mg, 30 µmol, 78%) as a pale yellow oil.

*Procedure for the synthesis of benzothiophene **5c** by the reaction of aryne precursor **1a** with alkynyl sulfide **2a** under CO_2 atmosphere*

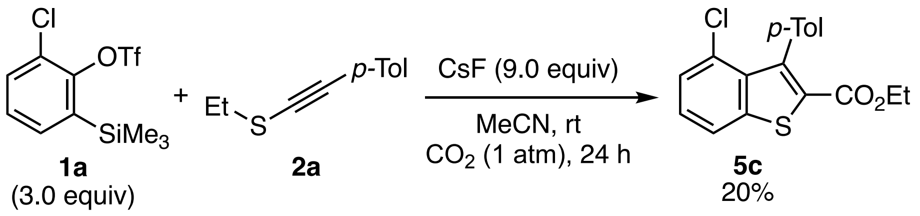

To a mixture of 2-chloro-6-(trimethylsilyl)phenyl triflate (**1a**) (49.9 mg, 0.150 mmol, 3.0 equiv) and ethyl (4-tolyl)ethynyl sulfide (**2a**) (8.81 mg, 50.0 µmol) dissolved in MeCN (1.0 mL) was added cesium fluoride (68.4 mg, 0.450 mmol, 9.0 equiv) at room temperature. The reaction mixture was evacuated and backfilled with CO_2 (1 atm) for three times. After stirring for 24 h at the same temperature, to the mixture was added water (3 mL). The mixture was extracted with EtOAc (10 mL × 3), and the combined organic extract was washed with brine (5 mL), dried (Na_2SO_4), and after filtration, the filtrate was concentrated under reduced pressure. The residue was purified by preparative TLC (*n*-hexane/CH_2Cl_2 = 3/1) to give 4-chloro-2-ethoxycarbonyl-3-(4-tolyl)benzo[*b*]thiophene (**5c**) (3.30 mg, 9.97 µmol, 20%) as a colorless solid.

*Procedure for the synthesis of 4-chloro-3-(hydroxymethyl)benzo[b]thiophene (**3w**)*

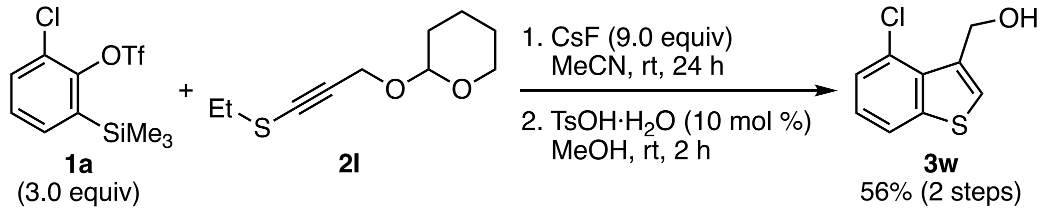

To a mixture of 2-chloro-6-(trimethylsilyl)phenyl triflate (**1a**) (2.00 g, 6.00 mmol, 3.0 equiv) and 2-((3-(ethylthio)prop-2-yn-1-yl)oxy)tetrahydro-2*H*-pyran (**2l**) (401 mg, 2.00 mmol) dissolved in MeCN (40 mL) were added cesium fluoride (2.73 g, 18.0 mmol, 9.0 equiv) at room temperature. After stirring for 24 h at room temperature, to the mixture was added an aqueous saturated solution of sodium bicarbonate (30 mL). The mixture was extracted with EtOAc (30 mL × 3), and the combined organic extract was washed with brine (10 mL), dried (Na_2SO_4), and after filtration, the filtrate was concentrated under reduced pressure. All the resulting mixture was used for the following procedure.

To a solution of the residue dissolved in MeOH (20 mL) was added *p*-toluenesulfonic acid monohydrate (38.0 mg, 0.200 mmol, 10 mol %) at room temperature. After stirring for 2 h at room temperature, to the mixture was added an aqueous saturated solution of sodium bicarbonate (30 mL). The mixture was extracted with CH_2Cl_2 (30 mL × 3), and the combined organic extract was washed with brine (10 mL), dried (Na_2SO_4), and after filtration, the filtrate was concentrated under reduced pressure. The residue was purified by flash column chromatography (silica-gel 16 g, *n*-hexane/EtOAc = 4/1) to give 4-chloro-3-(hydroxymethyl)benzo[*b*]thiophene (**3w**) (223 mg, 1.12 mmol, 56%) as a colorless solid.

*Procedure for the synthesis of 4-chlorobenzo[b]thiophene-3-carboxylic acid (**9**)*[S12]

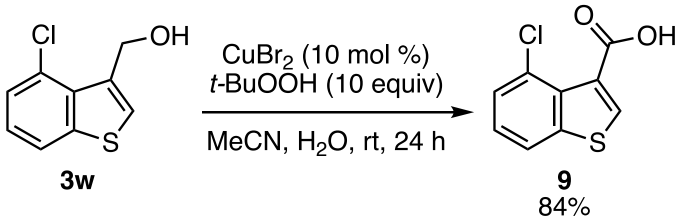

To a mixture of 4-chloro-3-(hydroxymethyl)benzo[*b*]thiophene (**3w**) (99.3 mg, 0.500 mmol) and $CuBr_2$ (11.2 mg, 50.0 µmol, 10 mol %) dissolved in MeCN (3.0 mL) was slowly added *t*-BuOOH (70% water solution, 640 µL, 5.00 mmol, 10 equiv) at room temperature. After stirring for 24 h at room temperature, to the mixture was added an aqueous saturated solution of sodium bicarbonate (10 mL). The mixture was extracted with EtOAc (10 mL), and the aqueous layer was acidified using 1 M HCl. The aqueous layer was extracted with EtOAc (20 mL × 3), and the combined organic extract was washed with brine (10 mL), dried (Na_2SO_4), and after filtration, the filtrate was concentrated under reduced pressure. The residue was purified by flash column chromatography (silica-gel 1.8 g, EtOAc) to give 4-chlorobenzo[*b*]thiophene-3-carboxylic acid (**9**) (88.9 mg, 0.418 mmol, 84%) as a colorless solid.

*Procedure for the synthesis of 4-chloro-3-iodobenzo[b]thiophene (**10**)*[S13]

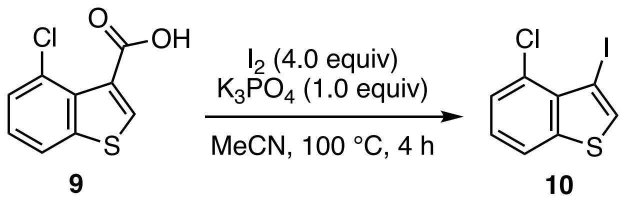

9 **10** 84%

To a mixture of 4-chlorobenzo[*b*]thiophene-3-carboxylic acid (**9**) (21.3 mg, 0.100 mmol) and I_2 (102 mg, 0.400 mmol, 4.0 equiv) dissolved in MeCN (500 µL) was added tripotassium phosphate (21.2 mg, 0.100 mmol, 1.0 equiv) at room temperature. After stirring for 4 h at 100 °C, the mixture was cooled to room temperature, and to this was added an aqueous saturated solution of sodium thiosulfate (5 mL). The mixture was extracted with CH_2Cl_2 (10 mL × 3), and the combined organic extract was washed with brine (5 mL), dried (Na_2SO_4), and after filtration, the filtrate was concentrated under reduced pressure. The residue was purified by preparative TLC (*n*-hexane) to give 4-chloro-3-iodobenzo[*b*]thiophene (**10**) (24.8 mg, 84.2 µmol, 84%) as a colorless solid.

*Palladium-catalyzed direct C2 C–H arylation for the synthesis of benzo[b]thiophene **11***[S14]

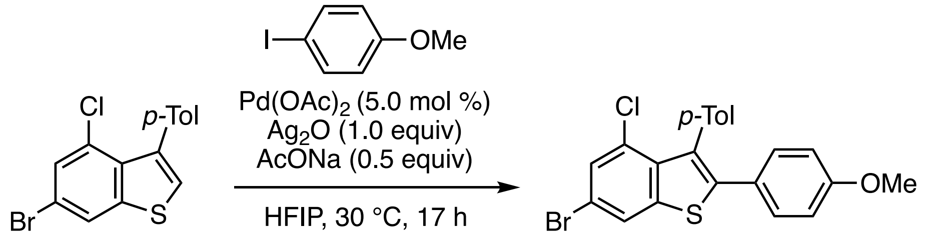

3t (1.5 equiv) **11** 48%

To a mixture of 6-bromo-4-chloro-3-(4-tolyl)benzo[*b*]thiophene (**3t**) (68.0 mg, 0.180 mmol, 1.5 equiv) and 4-iodoanisole (28.0 mg, 0.120 mmol) dissolved in 1,1,1,3,3,3-hexafluoro-2-propanol (HFIP) (3.0 mL) were added $Pd(OAc)_2$ (1.35 mg, 6.00 µmol, 5.0 mol %), Ag_2O (27.8 mg, 0.120 mmol, 1.0 equiv), and AcONa (4.92 mg, 60.0 µmol, 0.50 equiv) at 30 ºC. After stirring for 17 h at the same temperature, the resultant mixture was diluted with EtOAc (5 mL) and filtered through a plug of silica. The silica plug was flushed with EtOAc (30 mL), and the filtrate was concentrated under reduced pressure. The residue was purified by preparative TLC (*n*-hexane/CH_2Cl_2 = 9/1) to give 6-bromo-4-chloro-2-(4-methoxyphenyl)-3-(4-tolyl)benzo[*b*]thiophene (**11**) (25.6 mg, 57.7 µmol, 48%) as a colorless oil.

Consecutive Suzuki–Miyaura cross-coupling for the synthesis of benzo[b]thiophene **12**

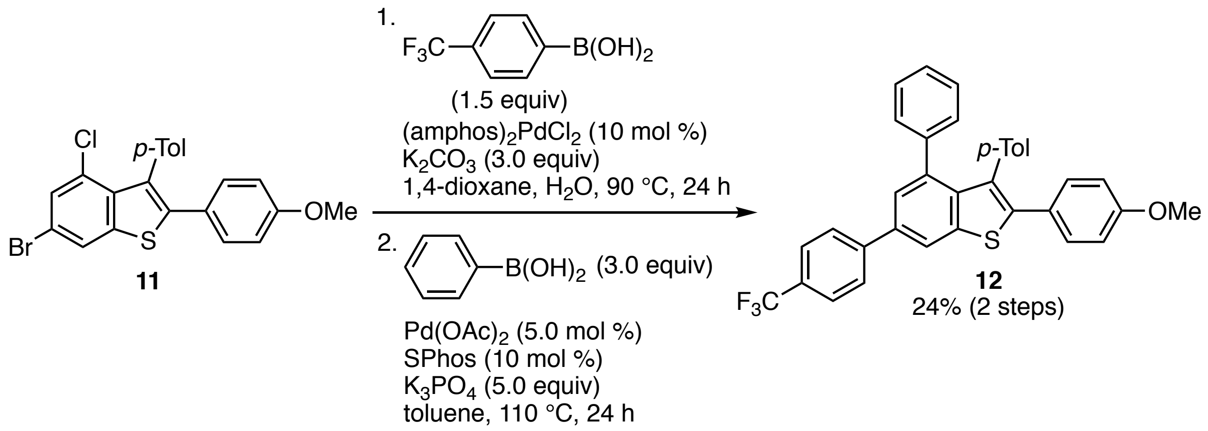

To a mixture of 6-bromo-4-chloro-2-(4-methoxyphenyl)-3-(4-tolyl)benzo[*b*]thiophene (**11**) (17.8 mg, 40.0 μmol) and 4-(trifluoromethyl)phenylboronic acid (11.4 mg, 60.0 μmol, 1.5 equiv) dissolved in 1,4-dioxane (720 μL) and water (80 μL) were added $(amphos)_2PdCl_2$ (2.83 mg, 4.00 μmol, 10 mol %) and K_2CO_3 (16.6 mg, 0.120 mmol, 3.0 equiv) at room temperature. After stirring for 24 h at 90 °C, the mixture was cooled to room temperature, and to this was added water (5 mL). The mixture was extracted with EtOAc (10 mL × 3), and the combined organic extract was washed with brine (5 mL), dried (Na_2SO_4), and after filtration, the filtrate was concentrated under reduced pressure. All the resulting mixture was used for the following procedure.

To a mixture of the residue prepared above and phenylboronic acid (14.6 mg, 0.120 mmol, 3.0 equiv) dissolved in toluene (800 μL) were added $Pd(OAc)_2$ (0.45 mg, 2.0 μmol, 5.0 mol %), SPhos (1.64 mg, 4.0 μmol, 10 mol %), and K_3PO_4 (42.5 mg, 0.200 mmol, 5.0 equiv) at room temperature. After stirring for 24 h at 110 °C, the mixture was cooled to room temperature, and to this was added water (5 mL). The mixture was extracted with EtOAc (10 mL × 3), and the combined organic extract was washed with brine (5 mL), dried (Na_2SO_4), and after filtration, the filtrate was concentrated under reduced pressure. The residue was purified by preparative TLC (*n*-hexane/CH_2Cl_2 = 3/1) to give 2-(4-methoxyphenyl)-4-phenyl-3-(4-tolyl)-6-(4-(trifluoromethyl)phenyl)benzo[*b*]thiophene (**12**) (5.3 mg, 9.6 μmol, 24%) as a colorless solid.

Procedure for the iodination of benzothiophene ***3x*** *via deprotolithilation at the C2 position*

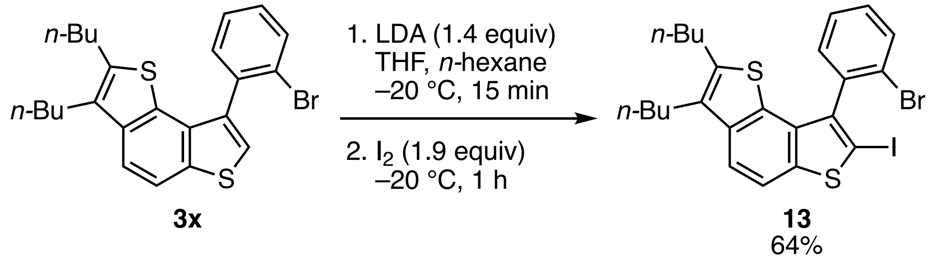

To a solution of 8-(2-bromophenyl)-2,3-(dibutyl)benzo[1,2-*b*:3,4-*b′*]dithiophene (**3x**) (24.2 mg, 52.8 μmol) dissolved in THF (500 μL) was slowly added LDA (1.00 M, THF/*n*-hexane solution, 75.0 μL, 75.0 μmol, 1.4 equiv) at –20 °C. After stirring for 15 min at the same temperature, to the mixture was added iodine (25.4 mg, 0.100 mmol, 1.9 equiv) at –20 °C. After stirring for 1 h at the same temperature, the mixture was allowed to warm to room temperature, and to this were added an aqueous saturated solution of sodium bicarbonate (5 mL) and an aqueous saturated solution of sodium thiosulfate (5 mL). The mixture was extracted with EtOAc (10 mL × 3), and the combined organic extract was washed with brine (5 mL), dried (Na_2SO_4), and after filtration, the filtrate was concentrated under reduced pressure. The residue was purified by preparative TLC (*n*-hexane/CH_2Cl_2 = 5/1) to give 8-(2-bromophenyl)-2,3-dibutyl-7-iodobenzo[1,2-*b*:3,4-*b′*]dithiophene (**13**) (19.8 mg, 33.9 μmol, 64%) as a pale yellow oil.

*Procedure for the synthesis of benzothiophene **14** by palladium-catalyzed N-arylation*

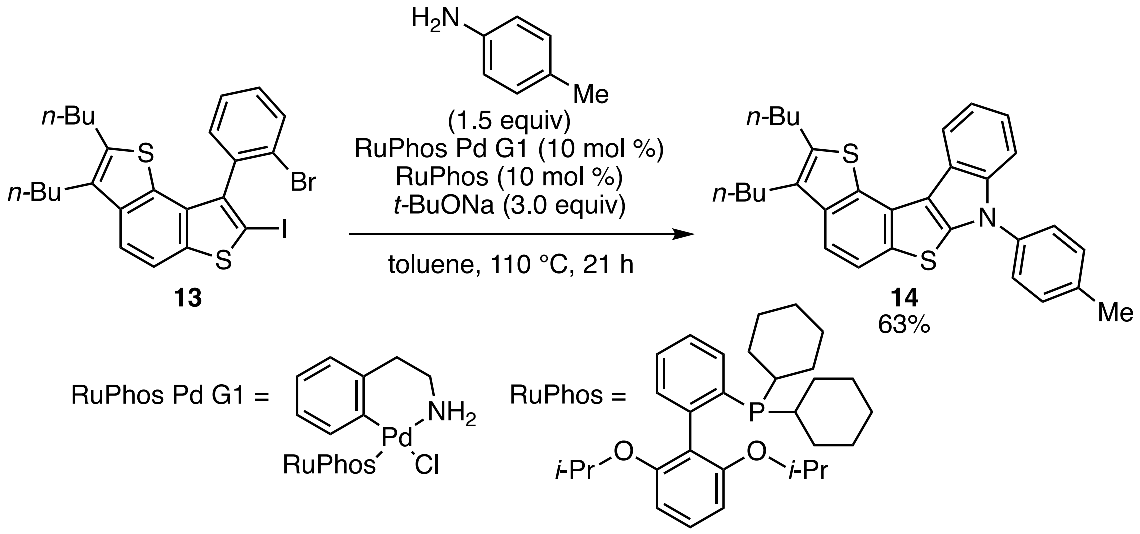

To a mixture of 8-(2-bromophenyl)-2,3-dibutyl-7-iodobenzo[1,2-*b*:3,4-*b'*]dithiophene (**13**) (33.7 mg, 57.8 μmol) and *p*-toluidine (9.29 mg, 86.7 μmol, 1.5 equiv) dissolved in toluene (1.0 mL) were added RuPhos Pd G1 (4.20 mg, 5.78 μmol, 10 mol %), RuPhos (2.70 mg, 5.78 μmol, 10 mol %), and *t*-BuONa (16.6 mg, 0.173 mmol, 3.0 equiv) at room temperature. After stirring for 21 h at 110 °C, the mixture was cooled to room temperature, and to this was added water (5 mL). The mixture was extracted with EtOAc (10 mL × 3), and the combined organic extract was washed with brine (5 mL), dried (Na_2SO_4), and after filtration, the filtrate was concentrated under reduced pressure. The residue was purified by preparative TLC (*n*-hexane/CH_2Cl_2 = 6/1) to give 2,3-dibutyl-7-(4-tolyl)-7*H*-thieno[3",2":5',6']benzo[1',2':4,5]thieno[2,3-*b*]indole (**14**) (17.5 mg, 36.3 μmol, 63%) as a colorless solid.

Deuteration Experiments

Deuteration experiment using CD_3CN

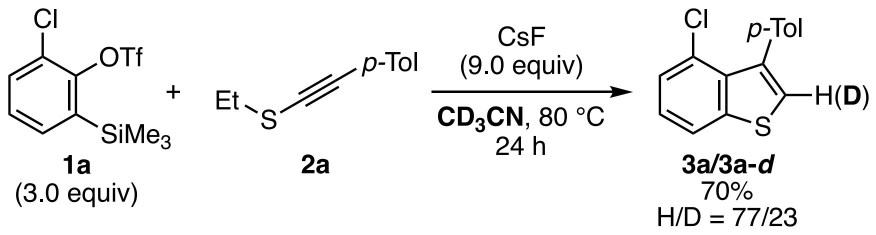

To a mixture of 2-chloro-6-(trimethylsilyl)phenyl triflate (**1a**) (49.9 mg, 0.150 mmol, 3.0 equiv) and ethyl (4-tolyl)ethynyl sulfide (**2a**) (8.81 mg, 50.0 µmol) dissolved in CD_3CN (1.0 mL) was added cesium fluoride (68.4 mg, 0.450 mmol, 9.0 equiv) at room temperature. After stirring for 24 h at 80 °C, the mixture was cooled to room temperature, and to this was added water (3 mL). The mixture was extracted with EtOAc (10 mL × 3), and the combined organic extract was washed with brine (5 mL), dried (Na_2SO_4), and after filtration, the filtrate was concentrated under reduced pressure. The residue was purified by preparative TLC (*n*-hexane) to give mixture of **3a** and ***d*-3a** (9.0 mg, 35 µmol, 70%, 23 atom %D) (determined by comparing the relative values of integration for the peaks observed at 7.70 ppm (1H for **3a**) in DMSO-d_6) was obtained as a colorless oil.

*Deuteration experiment using deuterated ethyl-substituted alkynyl sulfide **2a-d***

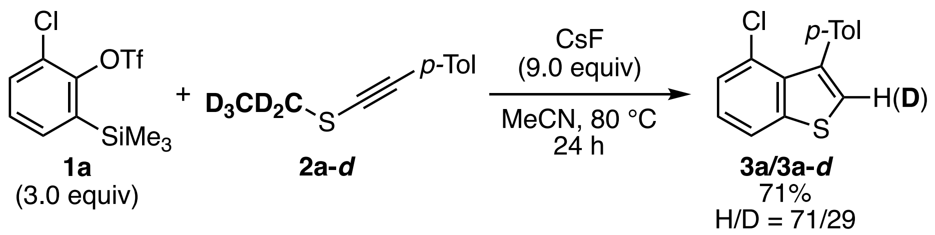

To a mixture of 2-chloro-6-(trimethylsilyl)phenyl triflate (**1a**) (49.9 mg, 0.150 mmol, 3.0 equiv) and pentadeuterioethyl (4-tolyl)ethynyl sulfide (**2a-*d***) (11.1 mg, 50.0 µmol) dissolved in MeCN (1.0 mL) was added cesium fluoride (68.4 mg, 0.450 mmol, 9.0 equiv) at room temperature. After stirring for 24 h at 80 °C, the mixture was cooled to room temperature, and to this was added water (3 mL). The mixture was extracted with EtOAc (10 mL × 3), and the combined organic extract was washed with brine (5 mL), dried (Na_2SO_4), and after filtration, the filtrate was concentrated under reduced pressure. The residue was purified by preparative TLC (*n*-hexane) to give mixture of **3a** and **3a-*d*** (9.20 mg, 35.5 µmol, 71%, 29 atom %D) (determined by comparing the relative values of integration for the peaks observed at 7.70 ppm (1H for **3a**) in DMSO-d_6) was obtained as a colorless oil.

Benzothiophene synthesis in the presence of D_2O

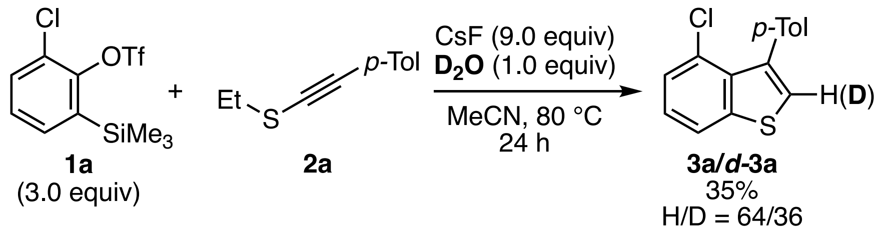

To a mixture of 2-chloro-6-(trimethylsilyl)phenyl triflate (**1a**) (49.9 mg, 0.150 mmol, 3.0 equiv) and ethyl (4-tolyl)ethynyl sulfide (**2a**) (8.81 mg, 50.0 µmol) dissolved in MeCN (1.0 mL) was added cesium fluoride (68.4 mg, 0.450 mmol, 9.0 equiv) and deuterium oxide (0.91 µL, 50 µmol, 1.0 equiv) at room temperature. After stirring for 24 h at 80 °C, the mixture was cooled to room temperature, and to this was added water (3 mL). The mixture was extracted with EtOAc (10 mL × 3), and the combined organic extract was washed with brine (5 mL), dried (Na_2SO_4), and after filtration, the filtrate was concentrated under reduced pressure. The residue was purified by preparative TLC (*n*-hexane) to give mixture of **3a** and ***d*-3a** (4.5 mg, 17 µmol, 35%, 36 atom %D) (determined by comparing the relative values of integration for the peaks observed at 7.70 ppm (1H for **3a**) in DMSO-*d*6) was obtained as a colorless oil.

Benzothiophene synthesis using D_2O in work-up

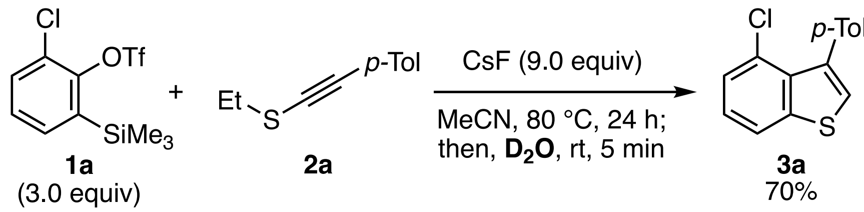

To a mixture of 2-chloro-6-(trimethylsilyl)phenyl triflate (**1a**) (49.9 mg, 0.150 mmol, 3.0 equiv) and ethyl (4-tolyl)ethynyl sulfide (**2a**) (8.81 mg, 50.0 µmol) dissolved in MeCN (1.0 mL) was added cesium fluoride (68.4 mg, 0.450 mmol, 9.0 equiv) at room temperature. After stirring for 24 h at 80 °C, the mixture was cooled to room temperature, and to this was added deuterium oxide (2 mL). After stirring for 5 min at the same temperature, the mixture was extracted with EtOAc (10 mL × 3), and the combined organic extract was washed with brine (5 mL), dried (Na_2SO_4), and after filtration, the filtrate was concentrated under reduced pressure. The residue was purified by preparative TLC (*n*-hexane) to give 4-chloro-3-(4-tolyl)benzo[*b*]thiophene (**3a**) (9.1 mg, 35 µmol, 70%) as a colorless oil, in which no incorporation of deuterium was observed.

Characterization Data of New Compounds

Methyl (4-tolyl)ethynyl sulfide,[S15] ethyl (4-tolyl)ethynyl sulfide (**2a**),[S15] 4-tolyl (4-tolyl)ethynyl sulfide,[S16] ethyl (4-methoxyphenyl)ethynyl sulfide (**2b**),[S17] (4-chlorophenyl)ethynyl ethyl sulfide (**2c**),[S18] ethyl (2-naphthyl)ethynyl sulfide (**2f**),[S19] 3-(4-tolyl)benzo[*b*]thiophene (**3l**),[S20] 5-methyl-3-(4-tolyl)benzo[*b*]thiophene (**3q**),[S21] 6-methyl-3-(4-tolyl)benzo[*b*]thiophene (**3q'**),[S22] 5-methoxy-3-(4-tolyl)benzo[*b*]thiophene (**3r**),[S23] and 6-methoxy-3-(4-tolyl)benzo[*b*]thiophene (**3r'**)[S24] were identical in spectra data with those reported in the literature.

Isopropyl (4-tolyl)ethynyl sulfide

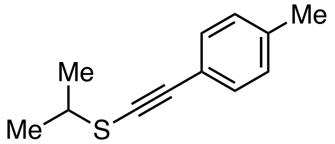

Colorless oil; TLC R_f 0.32 (*n*-hexane); ^{1}H NMR ($CDCl_3$, 500 MHz) δ 1.42 (d, 6H, J = 6.7 Hz), 2.34 (s, 3H), 3.24 (sept, 1H, J = 6.7 H), 7.08–7.12 (AA'BB', 2H), 7.30–7.34 (AA'BB', 2H); ^{13}C NMR ($CDCl_3$, 126 MHz) δ 21.4 (1C), 22.9 (2C), 39.8 (1C), 77.5 (1C), 94.8 (1C), 120.5 (1C), 129.0 (2C), 131.4 (2C), 138.1 (1C); IR (KBr, cm^{-1}) 814, 1051, 1236, 1248, 1450, 1506, 2864, 2922, 2963; HRMS (ESI$^+$) *m/z* 191.0890 ([M+H]$^+$, $C_{12}H_{15}S^+$ requires 191.0889).

Benzyl (4-tolyl)ethynyl sulfide

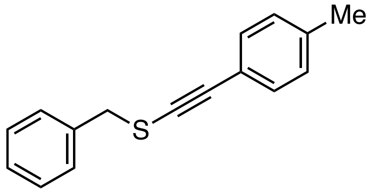

Pale red oil; TLC R_f 0.14 (*n*-hexane); ^{1}H NMR ($CDCl_3$, 500 MHz) δ 2.33 (s, 3H), 4.00 (s, 2H), 7.06–7.11 (AA'BB', 2H), 7.22–7.26 (AA'BB', 2H), 7.27–7.40 (m, 5H); ^{13}C NMR ($CDCl_3$, 126 MHz) δ 21.5 (1C), 40.5 (1C), 78.1 (1C), 94.6 (1C), 120.2 (1C), 127.7 (1C), 128.5 (2C), 129.0 (2C), 129.1 (2C), 131.4 (2C), 136.6 (1C), 138.3 (1C); IR (KBr, cm^{-1}) 530, 696, 764, 816, 1454, 1495, 1506, 3028; HRMS (ESI$^+$) *m/z* 239.0887 ([M+H]$^+$, $C_{16}H_{15}S^+$ requires 239.0889).

Ethyl (4-methoxycarbonylphenyl)ethynyl sulfide (**2d**)

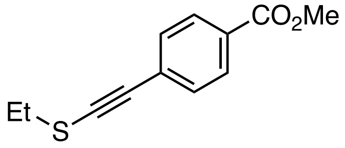

Pale yellow solid; Mp 37–39 ºC; TLC R_f 0.30 (*n*-hexane/CH_2Cl_2 = 1/1); ^{1}H NMR ($CDCl_3$, 500 MHz) δ 1.47 (t, 3H, J = 7.3 Hz), 2.85 (q, 2H, J = 7.3 Hz), 3.91 (s, 3H), 7.41–7.47 (AA'BB', 2H), 7.94–7.99 (AA'BB', 2H); ^{13}C NMR ($CDCl_3$, 126 MHz) δ 14.7 (1C), 30.0 (1C), 52.2 (1C), 83.4 (1C), 93.2 (1C), 128.2 (1C), 128.9 (1C), 129.4 (2C), 130.8 (2C), 166.5 (1C); IR (KBr, cm^{-1}) 768, 1107, 1175, 1275, 1308, 1435, 1603, 1722, 2162, 2949; HRMS (ESI$^+$) *m/z* 243.0449 ([M+Na]$^+$, $C_{12}H_{12}NaO_2S^+$ requires 243.0450).

(2-Bromophenyl)ethynyl ethyl sulfide (**2e**)

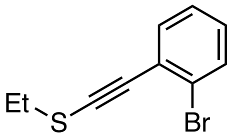

Colorless oil; TLC R_f 0.22 (*n*-hexane); ^{1}H NMR ($CDCl_3$, 500 MHz) δ 1.51 (t, 3H, J = 7.3 Hz), 2.86 (q, 2H, J = 7.3 Hz), 7.12 (ddd, 1H, J = 7.7, 7.7, 1.7 Hz), 7.23 (ddd, 1H, J = 7.7, 7.7, 1.2 Hz), 7.41 (dd, 1H, J = 7.7, 1.7 Hz), 7.55 (dd, 1H, J = 7.7, 1.2 Hz); ^{13}C NMR ($CDCl_3$, 126 MHz) δ 14.9 (1C), 30.1 (1C), 84.8 (1C), 92.2 (1C), 124.8 (1C), 125.6 (1C), 126.9 (1C), 128.8 (1C), 132.3 (1C), 132.6 (1C); IR (KBr, cm^{-1}) 679, 750, 1024, 1045, 1258, 1431, 1464, 2170, 2926, 2965; HRMS (ESI$^+$) *m/z* 262.9496 ([M+Na]$^+$, $C_{10}H_9{}^{79}BrNaS^+$ requires 262.9501).

Ethyl (9-phenanthrenyl)ethynyl sulfide (**2g**)

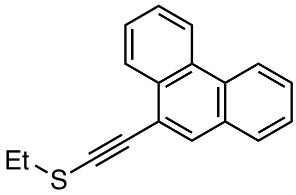

Yellow solid; Mp 56–57 ºC; TLC R_f 0.63 (*n*-hexane/CH_2Cl_2 = 1/1); ^{1}H NMR ($CDCl_3$, 500 MHz) δ 1.55 (t, 3H, *J* = 7.3 Hz), 2.93 (q, 2H, *J* = 7.3 Hz), 7.57 (ddd, 1H, *J* = 7.9, 7.9, 1.0 Hz), 7.60–7.71 (m, 3H), 7.82 (d, 1H, *J* = 8.0 Hz), 7.95 (s, 1H), 8.37–8.43 (m, 1H), 8.62 (d, 1H, *J* = 8.0 Hz), 8.64–8.69 (m, 1H); ^{13}C NMR ($CDCl_3$, 126 MHz) δ 14.9 (1C), 30.2 (1C), 83.8 (1C), 91.8 (1C), 119.9 (1C), 122.6 (1C), 122.7 (1C), 126.85 (1C), 126.91 (1C), 127.0 (1C+1C, two signals overlapped), 127.3 (1C), 128.4 (1C), 130.0 (1C), 130.1 (1C), 131.16 (1C), 131.20 (1C), 131.5 (1C); IR (KBr, cm^{-1}) 723, 748, 764, 891, 1256, 1377, 1450, 1491, 2924; HRMS (ESI$^+$) *m*/*z* 285.0700 ([M+Na]$^+$, $C_{18}H_{14}NaS^+$ requires 285.0708).

Ethyl (3-thienyl)ethynyl sulfide (**2h**)

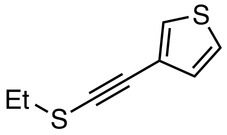

Colorless oil; TLC R_f 0.19 (*n*-hexane); ^{1}H NMR ($CDCl_3$, 500 MHz) δ 1.44 (t, 3H, *J* = 7.3 Hz), 2.80 (q, 2H, *J* = 7.3 Hz), 7.10 (dd, 1H, *J* = 5.0, 1.2 Hz), 7.24 (dd, 1H, *J* = 5.0, 3.0 Hz), 7.43 (dd, 1H, *J* = 3.0, 1.2 Hz); ^{13}C NMR ($CDCl_3$, 126 MHz) δ 14.7 (1C), 29.9 (1C), 78.5 (1C), 88.2 (1C), 122.5 (1C), 125.1 (1C), 128.9 (1C), 130.0 (1C); IR (KBr, cm^{-1}) 625, 779, 837, 872, 957, 1260, 1354, 1447, 2924, 2965; HRMS (ESI$^+$) *m*/*z* 169.0144 ([M+H]$^+$, $C_8H_9S_2^+$ requires 169.0140).

Ethyl (2-phenethyl)ethynyl sulfide (**2i**)

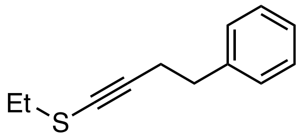

Pale yellow oil; TLC R_f 0.22 (*n*-hexane); ^{1}H NMR ($CDCl_3$, 500 MHz) δ 1.32 (t, 3H, *J* = 7.3 Hz), 2.60 (t, 2H, *J* = 7.5 Hz), 2.65 (q, 2H, *J* = 7.3 Hz), 2.84 (t, 2H, *J* = 7.3 Hz), 7.18–7.24 (m, 3H), 7.26–7.32 (AA’BB’C, 2H); ^{13}C NMR ($CDCl_3$, 126 MHz) δ 14.6 (1C), 22.3 (1C), 29.5 (1C), 35.2 (1C), 69.0 (1C), 93.9 (1C), 126.3 (1C), 128.3 (2C), 128.4 (2C), 140.6 (1C); IR (KBr, cm^{-1}) 698, 748, 1260, 1452, 1495, 2926, 2965, 3026; HRMS (ESI$^+$) *m*/*z* 213.0708 ([M+Na]$^+$, $C_{12}H_{14}NaS^+$ requires 213.0708).

Cyclohexylethynyl ethyl sulfide (**2j**)

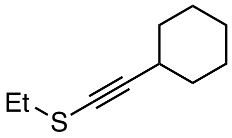

Colorless oil; TLC R_f 0.30 (*n*-hexane); ^{1}H NMR ($CDCl_3$, 500 MHz) δ 1.25–1.35 (m, 3H), 1.38 (t, 3H, *J* = 7.3 Hz), 1.40–1.54 (m, 3H), 1.65–1.74 (m, 2H), 1.75–1.84 (m, 2H), 2.44–2.53 (m, 1H), 2.68 (q, 2H, *J* = 7.3 Hz); ^{13}C NMR ($CDCl_3$, 126 MHz) δ 14.5 (1C), 24.8 (1C), 25.8 (2C), 29.6 (2C), 30.4 (1C), 32.7 (1C), 67.8 (1C), 98.9 (1C); IR (KBr, cm^{-1}) 970, 1032, 1055, 1072, 1258, 1447, 2853, 2928; HRMS (ESI$^+$) *m*/*z* 169.1040 ([M+H]$^+$, $C_{10}H_{17}S^+$ requires 169.1045).

3,17-Di-*O*-methyl-17-(ethylthioethynyl)estradiol (**2k**)

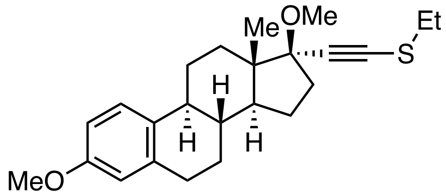

Colorless oil; TLC R_f 0.25 (*n*-hexane/CH_2Cl_2 = 1/1); ^{1}H NMR ($CDCl_3$, 500 MHz) δ 0.87 (s, 3H), 1.31–1.53 (m, 7H), 1.72–2.04 (m, 6H), 2.16–2.27 (m, 2H), 2.28–2.36 (m, 1H), 2.69–2.79 (m, 2H), 2.80–2.94 (m, 2H), 3.40 (s, 3H), 3.77 (s, 3H), 6.62 (d, 1H, *J* = 2.7 Hz), 6.71 (dd, 1H, *J* = 8.6, 2.7 Hz), 7.20 (d, 1H, *J* = 8.6 Hz); ^{13}C NMR ($CDCl_3$, 126 MHz) δ 12.9 (1C), 14.9 (1C), 22.8 (1C), 26.6 (1C), 27.3 (1C), 29.8 (1C), 29.9 (1C), 34.4 (1C), 36.8 (1C), 39.2 (1C), 43.5 (1C), 47.9 (1C), 49.7 (1C), 53.3 (1C), 55.2 (1C), 77.2 (1C), 86.7 (1C), 95.2 (1C), 111.5 (1C), 113.8 (1C), 126.3 (1C), 132.6 (1C), 137.9 (1C), 157.4 (1C); IR (KBr, cm^{-1}) 1047, 1082, 1098, 1238, 1256, 1452, 1499, 2870, 2930; HRMS (ESI^+) *m/z* 407.2014 ($[M+Na]^+$, $C_{24}H_{32}NaO_2S^+$ requires 407.2015).

2-((3-(Ethylthio)prop-2-yn-1-yl)oxy)tetrahydro-2*H*-pyran (**2l**)

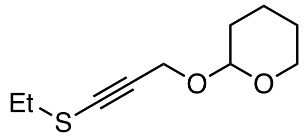

Colorless oil; TLC R_f 0.34 (*n*-hexane/EtOAc = 10/1); ^{1}H NMR ($CDCl_3$, 500 MHz) δ 1.39 (t, 3H, *J* = 7.3 Hz), 1.50–1.66 (m, 4H), 1.69–1.88 (m, 2H), 2.73 (q, 2H, *J* = 7.3 Hz), 3.49–3.56 (m, 1H), 3.80–3.88 (m, 1H), 4.31–4.44 (m, 2H), 4.80–4.85 (m, 1H); ^{13}C NMR ($CDCl_3$, 126 MHz) δ 14.6 (1C), 19.0 (1C), 25.3 (1C), 29.5 (1C), 30.2 (1C), 55.0 (1C), 62.0 (1C), 76.3 (1C), 90.6 (1C), 96.5 (1C); IR (KBr, cm^{-1}) 903, 1015, 1038, 1061, 1076, 1119, 2928, 2941; HRMS (ESI^+) *m/z* 223.0764 ($[M+Na]^+$, $C_{10}H_{16}NaO_2S^+$ requires 223.0763).

4-Chloro-3-(4-tolyl)benzo[*b*]thiophene (**3a**)

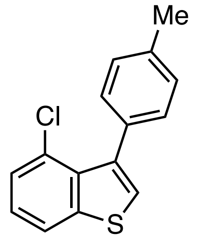

Colorless oil; TLC R_f 0.33 (*n*-hexane); ^{1}H NMR (DMSO-d_6, 500 MHz) δ 2.37 (s, 3H), 7.19–7.24 (AA'BB', 2H), 7.27–7.30 (AA'BB', 2H), 7.36–7.44 (m, 2H), 7.70 (s, 1H), 8.07 (dd, 1H, *J* = 7.6, 1.3 Hz); ^{13}C NMR ($CDCl_3$, 126 MHz) δ 21.3 (1C), 121.6 (1C), 124.8 (1C), 126.2 (1C), 126.3 (1C), 128.0 (2C), 129.4 (1C), 130.1 (2C), 134.28 (1C), 134.33 (1C), 137.1 (1C), 138.4 (1C), 142.4 (1C); IR (KBr, cm^{-1}) 745, 770, 814, 829, 1092, 1198, 1395, 1443, 1526; HRMS (ESI^+) *m/z* 281.0166 ($[M+Na]^+$, $C_{15}H_{11}{}^{35}ClNaS^+$ requires 281.0162).

The regiochemistry of **3a** was determined by the HMBC experiment.

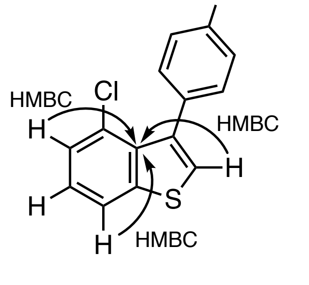

4-Chloro-3-(4-methoxyphenyl)benzo[*b*]thiophene (**3b**)

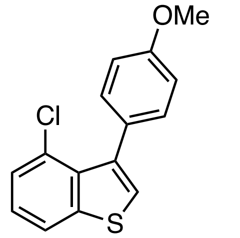

Pale yellow solid; Mp 94–96 ºC; TLC R_f 0.55 (*n*-hexane/CH_2Cl_2 = 1/1); ^{1}H NMR ($CDCl_3$, 500 MHz) δ 3.87 (s, 3H), 6.91–6.96 (AA′BB′, 2H), 7.24–7.30 (m, 2H), 7.31–7.36 (m, 3H), 7.80 (dd, 1H, J = 8.0, 1.0 Hz); ^{13}C NMR ($CDCl_3$, 126 MHz) δ 55.3 (1C), 112.8 (2C), 121.6 (1C), 124.8 (1C), 126.2 (1C), 126.3 (1C), 129.5 (1C), 129.6 (1C), 131.4 (2C), 134.4 (1C), 138.1 (1C), 142.4 (1C), 159.1 (1C); IR (KBr, cm^{-1}) 746, 772, 833, 1034, 1175, 1246, 1287, 1491, 1526; HRMS (ESI$^+$) *m/z* 297.0111 ([M+Na]$^+$, $C_{15}H_{11}{}^{35}ClNaOS^+$ requires 297.0111).

4-Chloro-3-(4-chlorophenyl)benzo[*b*]thiophene (**3c**)

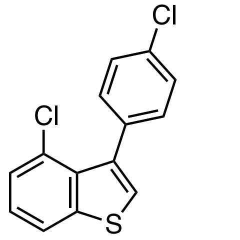

Colorless oil; TLC R_f 0.32 (*n*-hexane); ^{1}H NMR ($CDCl_3$, 500 MHz) δ 7.29 (dd, 1H, J = 7.9, 7.9 Hz), 7.32 (s, 1H), 7.33–7.39 (m, 5H), 7.82 (dd, 1H, J = 7.9, 1.1 Hz); ^{13}C NMR ($CDCl_3$, 126 MHz) δ 121.7 (1C), 125.1 (1C), 126.4 (1C), 126.7 (1C), 127.6 (2C), 129.3 (1C), 131.6 (2C), 133.5 (1C), 134.1(1C), 135.7 (1C), 137.1 (1C), 142.4 (1C); IR (KBr, cm^{-1}) 743, 772, 833, 1016, 1092, 1198, 1327, 1395, 1479, 1516; HRMS (ESI$^+$) *m/z* 278.9799 ([M+H]$^+$, $C_{14}H_9{}^{35}Cl_2S^+$ requires 278.9797).

4-Chloro-3-(4-methoxycarbonylphenyl)benzo[*b*]thiophene (**3d**)

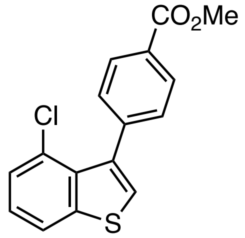

Colorless solid; Mp 109–111 ºC; TLC R_f 0.26 (*n*-hexane/CH_2Cl_2 = 1/1); ^{1}H NMR ($CDCl_3$, 500 MHz) δ 3.96 (s, 3H), 7.30 (dd, 1H, J = 7.8, 7.8 Hz), 7.34–7.37 (m, 2H), 7.48–7.52 (AA'BB', 2H), 7.83 (dd, 1H, J = 7.8, 1.1 Hz), 8.05–8.09 (AA'BB', 2H); ^{13}C NMR ($CDCl_3$, 126 MHz) δ 52.1 (1C), 121.7 (1C), 125.1 (1C), 126.4 (1C), 126.9 (1C), 128.6 (2C), 129.1 (1C), 129.3 (1C), 130.3 (2C), 134.0 (1C), 137.3 (1C), 142.1 (1C), 142.4 (1C), 167.0 (1C); IR (KBr, cm^{-1}) 704, 766, 1101, 1113, 1177, 1198, 1275, 1308, 1435, 1717; HRMS (ESI$^+$) *m/z* 325.0055 ([M+Na]$^+$, $C_{16}H_{11}{}^{35}ClNaO_2S^+$ requires 325.0060).

3-(2-Bromophenyl)-4-chlorobenzo[*b*]thiophene (**3e**)

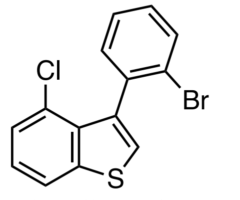

Pale yellow oil; TLC R_f 0.21 (*n*-hexane); ^{1}H NMR ($CDCl_3$, 500 MHz) δ 7.24–7.30 (m, 2H), 7.31–7.38 (m, 4H), 7.64 (d, 1H, J = 8.0 Hz), 7.81 (dd, 1H, J = 7.9, 1.0 Hz); ^{13}C NMR ($CDCl_3$, 126 MHz) δ 121.6 (1C), 125.1 (1C), 125.7 (1C), 126.1 (1C), 126.6 (1C), 126.7 (1C), 129.4 (1C+1C, two signals overlapped), 131.86 (1C), 131.92 (1C), 134.4 (1C), 136.8 (1C), 138.5 (1C), 141.9 (1C); IR (KBr, cm^{-1}) 743, 756, 772, 826, 1092, 1200, 1396, 1460; HRMS (ESI$^+$) *m/z* 344.9118 ([M+Na]$^+$, $C_{14}H_8{}^{79}Br^{35}ClNaS^+$ requires 344.9111).

4-Chloro-3-(2-naphthyl)benzo[*b*]thiophene (**3f**)

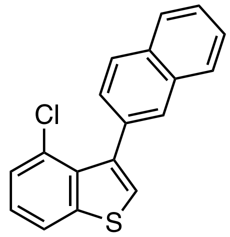

Pale yellow oil; TLC R_f 0.16 (*n*-hexane); ^{1}H NMR ($CDCl_3$, 500 MHz) δ 7.29 (dd, 1H, *J* = 7.7, 7.7 Hz), 7.35 (dd, 1H, *J* = 7.7, 1.0 Hz), 7.40 (s, 1H), 7.49–7.54 (m, 2H), 7.56 (dd, 1H, *J* = 8.3, 1.7 Hz), 7.82–7.92 (m, 5H); ^{13}C NMR ($CDCl_3$, 126 MHz) δ 121.7 (1C), 125.0 (1C), 126.0 (1C), 126.2 (1C), 126.3 (1C), 126.5 (1C), 126.8 (1C), 127.7 (1C), 128.0 (1C), 128.7 (1C), 128.9 (1C), 129.5 (1C), 132.7 (1C), 132.8 (1C), 134.5 (1C), 134.9 (1C), 138.4 (1C), 142.5 (1C); IR (KBr, cm^{-1}) 743, 766, 773, 818, 827, 853, 1090, 1200, 1443; HRMS (ESI$^+$) *m*/*z* 317.0159 ([M+Na]$^+$, $C_{18}H_{11}{}^{35}ClNaS^+$ requires 317.0162).

4-Chloro-3-(9-phenanthrenyl)benzo[*b*]thiophene (**3g**)

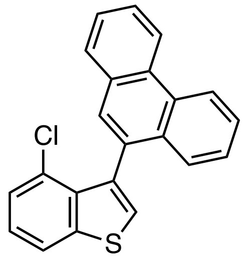

Pale yellow oil; TLC R_f 0.70 (*n*-hexane/CH_2Cl_2 = 1/1); ^{1}H NMR ($CDCl_3$, 500 MHz) δ 7.23–7.34 (m, 2H), 7.42–7.52 (m, 3H), 7.60–7.66 (m, 2H), 7.69 (ddd, 1H, *J* = 7.0, 7.0, 1.4 Hz), 7.76 (s, 1H), 7.86–7.92 (m, 2H), 8.72–8.78 (m, 2H); ^{13}C NMR ($CDCl_3$, 126 MHz) δ 121.6 (1C), 122.60 (1C), 122.64 (1C), 125.1 (1C), 126.1 (1C), 126.4 (1C), 126.6 (1C), 126.8 (1C+1C, two signals overlapped), 126.9 (1C), 127.2 (1C), 128.5 (1C), 128.7 (1C), 129.6 (1C), 129.8 (1C), 130.4 (1C), 131.3 (1C), 133.3 (1C), 134.0 (1C), 135.7 (1C), 136.2 (1C), 142.2 (1C); IR (KBr, cm^{-1}) 725, 746, 827, 1200, 1215, 1395, 1441, 1450; HRMS (ESI$^+$) *m*/*z* 367.0316 ([M+Na]$^+$, $C_{22}H_{13}{}^{35}ClNaS^+$ requires 367.0319).

4-Chloro-3-(3-thienyl)benzo[*b*]thiophene (**3h**)

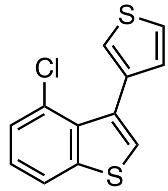

Pale yellow oil; TLC R_f 0.25 (*n*-hexane); ^{1}H NMR ($CDCl_3$, 500 MHz) δ 7.18 (dd, 1H, *J* = 4.8, 1.0 Hz), 7.24–7.29 (m, 2H), 7.30–7.37 (m, 3H), 7.79 (d, 1H, *J* = 8.0 Hz); ^{13}C NMR ($CDCl_3$, 126 MHz) δ 121.7 (1C), 123.8 (1C), 124.1 (1C), 124.9 (1C), 126.3 (1C), 126.8 (1C), 129.4 (1C), 130.6 (1C), 133.0 (1C), 134.5 (1C), 137.0 (1C), 142.2 (1C); IR (KBr, cm^{-1}) 652, 743, 768, 827, 1092, 1200, 1310, 1396, 1445; HRMS (ESI$^+$) *m*/*z* 250.9757 ([M+H]$^+$, $C_{12}H_8{}^{35}ClS_2{}^+$ requires 250.9750).

4-Chloro-3-(2-phenylethyl)benzo[*b*]thiophene (**3i**)

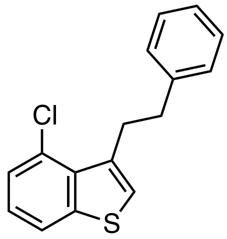

Colorless oil; TLC R_f 0.27 (*n*-hexane); ^{1}H NMR ($CDCl_3$, 500 MHz) δ 3.02–3.08 (m, 2H), 3.43–3.49 (m, 2H), 7.08 (s, 1H), 7.19–7.26 (m, 4H), 7.28–7.33 (AA'BB'C, 2H), 7.37 (dd, 1H, *J* = 7.8, 0.9 Hz), 7.74 (dd, 1H, *J* = 7.8, 0.9 Hz); ^{13}C NMR ($CDCl_3$, 126 MHz) δ 33.3 (1C), 37.3 (1C), 121.8 (1C), 123.9 (1C), 124.6 (1C), 126.0 (1C), 126.1 (1C), 128.4 (2C), 128.5 (2C), 129.0 (1C), 134.8 (1C), 137.1 (1C), 141.6 (1C), 143.2 (1C); IR (KBr, cm^{-1}) 698, 741, 764, 820, 1200, 1398, 1445, 1495, 1547; HRMS (ESI$^+$) *m*/*z* 295.0312 ([M+Na]$^+$, $C_{16}H_{13}{}^{35}ClNaS^+$ requires 295.0319).

4-Chloro-3-cyclohexylbenzo[*b*]thiophene (**3j**)

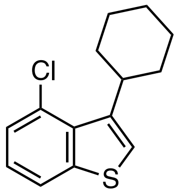

Colorless oil; TLC R_f 0.51 (*n*-hexane); ^{1}H NMR ($CDCl_3$, 500 MHz) δ 1.23–1.43 (m, 3H), 1.44–1.55 (m, 2H), 1.75–1.91 (m, 3H), 2.12–2.21 (m, 2H), 3.56–3.64 (m, 1H), 7.13–7.22 (m, 2H), 7.34 (dd, 1H, *J* = 7.8, 1.0 Hz), 7.73 (dd, 1H, *J* = 7.8, 1.0 Hz); ^{13}C NMR ($CDCl_3$, 126 MHz) δ 26.4 (1C), 26.9 (2C), 34.9 (2C), 38.8 (1C), 120.9 (1C), 121.8 (1C), 124.2 (1C), 126.3 (1C), 128.9 (1C), 134.6 (1C), 143.2 (1C), 144.1 (1C); IR (KBr, cm^{-1}) 743, 758, 770, 822, 1200, 1443, 2849, 2926; HRMS (ESI$^+$) *m*/*z* 251.0660 ([M+H]$^+$, $C_{14}H_{16}{}^{35}ClS^+$ requires 251.0656).

4-Chloro-3-(3,17-di-*O*-methylestradiol-17-yl)benzo[*b*]thiophene (**3k**)

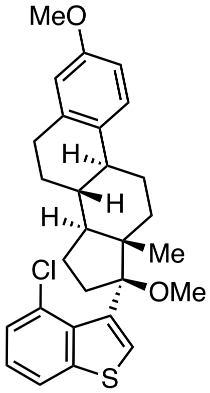

Colorless solid; Mp 198–200 ºC; TLC R_f 0.29 (*n*-hexane/CH_2Cl_2 = 1/1); ^{1}H NMR ($CDCl_3$, 500 MHz) δ 0.69 (ddd, 1H, *J* = 13.0, 13.0, 4.2 Hz), 1.12 (s, 3H), 1.22–1.34 (m, 1H), 1.36–1.53 (m, 3H), 1.56–1.67 (m, 1H), 1.79–1.86 (m, 1H), 1.86–1.95 (m, 2H), 1.98–2.06 (m, 1H), 2.31–2.39 (m, 1H), 2.42 (ddd, 1H, *J* = 12.6, 3.5, 3.5 Hz), 2.53 (ddd, 1H, *J* = 14.6, 9.8, 4.8 Hz), 2.76–2.91 (m, 2H), 3.08 (s, 3H), 3.74 (s, 3H), 6.59 (d, 1H, *J* = 2.7 Hz), 6.63 (dd, 1H, *J* = 8.6, 2.7 Hz), 7.03 (d, 1H, *J* = 8.6 Hz), 7.22–7.27 (m, 2H), 7.53 (dd, 1H, *J* = 7.6, 1.2 Hz), 7.78 (dd, 1H, *J* = 7.6, 1.2 Hz); ^{13}C NMR ($CDCl_3$, 126 MHz) δ 14.7 (1C), 23.1 (1C), 26.4 (1C), 27.4 (1C), 29.9 (1C), 33.1 (1C), 38.1 (1C), 39.3 (1C), 43.2 (1C), 48.2 (1C), 49.3 (1C), 52.3 (1C), 55.1 (1C), 92.1 (1C), 111.3 (1C), 113.7 (1C), 121.8 (1C), 124.2 (1C), 126.2 (1C), 127.3 (1C), 128.9 (1C), 129.4 (1C), 132.7 (1C), 136.5 (1C), 137.8 (1C), 139.2 (1C), 143.3 (1C), 157.3 (1C); IR (KBr, cm^{-1}) 756, 773, 1090, 1099, 1236, 1254, 1431, 1499, 2928; HRMS (ESI$^+$) *m*/*z* 489.1635 ([M+Na]$^+$, $C_{28}H_{31}{}^{35}ClNaO_2S^+$ requires 489.1625).

4-Fluoro-3-(4-tolyl)benzo[*b*]thiophene (**3m**)

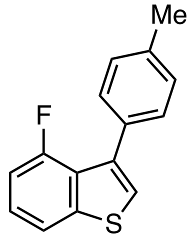

Colorless oil; TLC R_f 0.21 (*n*-hexane); ^{1}H NMR ($CDCl_3$, 500 MHz) δ 2.41 (s, 3H), 7.01 (ddd, 1H, *J* = 11.7, 8.0, 0.7 Hz), 7.20–7.25 (m, 3H), 7.30 (ddd, 1H, *J* = 8.0, 8.0, 4.6 Hz), 7.40–7.44 (m, 2H), 7.65 (dd, 1H, *J* = 8.0, 0.7 Hz); ^{13}C NMR ($CDCl_3$, 126 MHz) δ 21.2 (1C), 110.3 (d, 1C, $^2J_{C\text{–}F}$ = 21.0 Hz), 118.8 (d, 1C, $^4J_{C\text{–}F}$ = 4.0 Hz), 124.3 (1C), 125.2 (d, 1C, $^3J_{C\text{–}F}$ = 7.9 Hz), 126.6 (d, 1C, $^2J_{C\text{–}F}$ = 15.3 Hz), 128.6 (2C), 129.1 (2C), 133.5 (1C), 136.5 (d, 1C, $^4J_{C\text{–}F}$ = 3.7 Hz), 137.2 (1C), 143.3 (d, 1C, $^3J_{C\text{–}F}$ = 6.3 Hz), 158.5 (d, 1C, $^1J_{C\text{–}F}$ = 253.8 Hz); ^{19}F NMR ($CDCl_3$, 376 MHz) δ –113.7 to –113.3 (m); IR (KBr, cm^{-1}) 741, 772, 818, 922, 1240, 1335, 1462, 1528, 1557; HRMS (ESI$^+$) *m*/*z* 265.0453 ([M+Na]$^+$, $C_{15}H_{11}FNaS^+$ requires 265.0458).

The regiochemistry of **3m** was determined by the HMBC experiment.

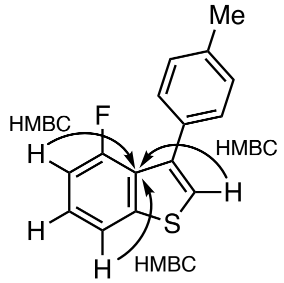

4-Bromo-3-(4-tolyl)benzo[*b*]thiophene (**3n**)

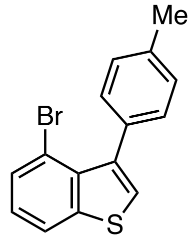

Colorless oil; TLC R_f 0.25 (*n*-hexane); ^{1}H NMR ($CDCl_3$, 500 MHz) δ 2.42 (s, 3H), 7.14–7.22 (m, 3H), 7.25–7.30 (AA'BB', 2H), 7.31 (s, 1H), 7.55 (dd, 1H, *J* = 7.8, 0.8 Hz), 7.85 (dd, 1H, *J* = 7.8, 0.8 Hz); ^{13}C NMR ($CDCl_3$, 126 MHz) δ 21.3 (1C), 117.4 (1C), 122.2 (1C), 125.0 (1C), 126.6 (1C), 128.0 (2C), 130.0 (1C), 130.5 (2C), 134.2 (1C), 135.5 (1C), 137.3 (1C), 139.1 (1C), 142.2 (1C); IR (KBr, cm^{-1}) 745, 768, 818, 1072, 1196, 1327, 1393, 1439, 1526; HRMS (ESI$^+$) *m*/*z* 324.9654 ([M+Na]$^+$, $C_{15}H_{11}{}^{79}BrNaS^+$ requires 324.9657).

The regiochemistry of **3n** was determined by the HMBC experiment.

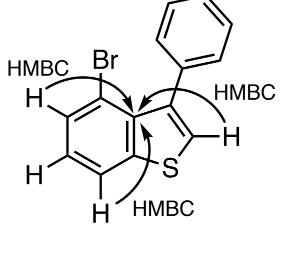

4-Methoxy-3-(4-tolyl)benzo[*b*]thiophene (**3o**)

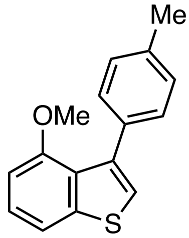

Colorless oil; TLC R_f 0.61 (n-hexane/CH_2Cl_2 = 1/1); ^{1}H NMR ($CDCl_3$, 500 MHz) δ 2.41 (s, 3H), 3.70 (s, 3H), 6.76 (d, 1H, J = 7.9 Hz), 7.13 (s, 1H), 7.16–7.21 (AA'BB', 2H), 7.30 (dd, 1H, J = 7.9, 7.9 Hz), 7.36–7.40 (AA'BB', 2H), 7.48 (d, 1H, J = 7.9 Hz); ^{13}C NMR ($CDCl_3$, 126 MHz) δ 21.2 (1C), 55.2 (1C), 105.2 (1C), 115.4 (1C), 123.2 (1C), 125.3 (1C), 127.6 (1C), 127.8 (2C), 129.5 (2C), 135.1 (1C), 136.3 (1C), 138.3 (1C), 142.6 (1C), 156.3 (1C); IR (KBr, cm^{-1}) 743, 772, 816, 1045, 1261, 1335, 1464, 1526, 1558; HRMS (ESI$^+$) m/z 277.0648 ([M+Na]$^+$, $C_{16}H_{14}NaOS^+$ requires 277.0658).

The regiochemistry of **3o** was determined by the HMBC experiment.

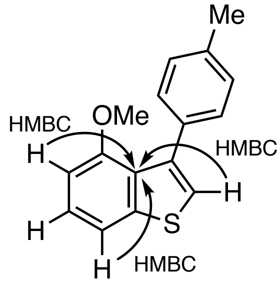

4-Diethylamino-3-(4-tolyl)benzo[*b*]thiophene (**3p**)

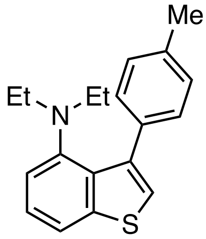

Pale yellow oil; TLC R_f 0.45 (n-hexane/CH_2Cl_2 = 1/1); ^{1}H NMR ($CDCl_3$, 500 MHz) δ 1.10 (t, 6H, J = 7.1 Hz), 2.42 (s, 3H), 3.30 (q, 4H, J = 7.1 Hz), 7.02 (d, 1H, J = 7.8 Hz), 7.26–7.30 (AA'BB', 2H), 7.31–7.36 (m, 2H), 7.46–7.50 (AA'BB', 2H), 7.58 (d, 1H, J = 7.8 Hz); ^{13}C NMR ($CDCl_3$, 126 MHz) δ 12.6 (2C), 21.2 (1C), 46.7 (2C), 115.9 (1C), 117.7 (1C), 122.8 (1C), 125.0 (1C), 128.6 (2C), 129.3 (2C), 133.5 (1C), 137.1 (1C), 137.6 (1C), 138.6 (1C), 139.3 (1C), 145.9 (1C); IR (KBr, cm^{-1}) 729, 793, 820, 1252, 1468, 1497, 1562, 2928, 2970; HRMS (ESI$^+$) m/z 296.1475 ([M+H]$^+$, $C_{19}H_{22}NS^+$ requires 296.1467).

The regiochemistry of **3p** was determined by the HMBC experiment.

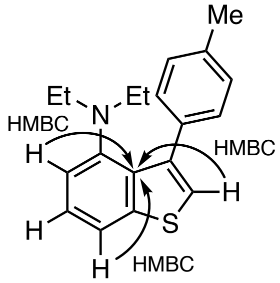

5,6-(Methylenedioxy)-3-(4-tolyl)benzo[*b*]thiophene (**3s**)

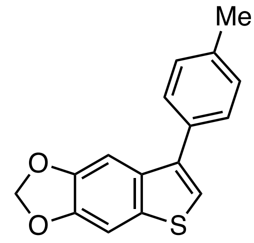

Yellow oil; TLC R_f 0.56 (*n*-hexane/CH_2Cl_2 = 1/1); ^{1}H NMR ($CDCl_3$, 500 MHz) δ 2.42 (s, 3H), 6.01 (s, 2H), 7.20 (s, 1H), 7.25–7.30 (m, 4H), 7.41–7.44 (AA′BB′, 2H); ^{13}C NMR ($CDCl_3$, 126 MHz) δ 21.2 (1C), 101.4 (1C), 101.82 (1C), 101.85 (1C), 121.1 (1C), 128.4 (2C), 129.4 (2C), 132.8 (1C), 133.2 (1C), 134.2 (1C), 137.3 (1C), 137.8 (1C), 146.7 (1C), 146.9 (1C); IR (KBr, cm^{-1}) 764, 820, 945, 1040, 1105, 1246, 1439, 1462, 1499; HRMS (ESI$^+$) *m*/*z* 269.0629 ([M+H]$^+$, $C_{16}H_{13}O_2S^+$ requires 269.0631).

6-Bromo-4-chloro-3-(4-tolyl)benzo[*b*]thiophene (**3t**)

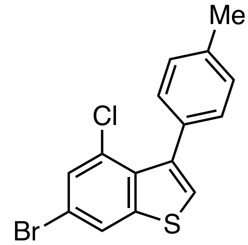

Colorless oil; TLC R_f 0.32 (*n*-hexane); ^{1}H NMR ($CDCl_3$, 500 MHz) δ 2.42 (s, 3H), 7.18–7.22 (AA'BB', 2H), 7.24–7.29 (m, 3H), 7.47 (d, 1H, J = 1.5 Hz), 7.93 (d, 1H, J = 1.5 Hz); ^{13}C NMR ($CDCl_3$, 126 MHz) δ 21.3 (1C), 117.5 (1C), 124.1 (1C), 126.5 (1C), 128.1 (2C), 129.0 (1C), 130.0 (1C), 130.1 (2C), 133.4 (1C), 133.6 (1C), 137.4 (1C), 138.3 (1C), 143.5 (1C); IR (KBr, cm^{-1}) 785, 799, 812, 843, 1194, 1325, 1360, 1422, 1520, 1570; HRMS (ESI$^+$) *m*/*z* 358.9266 ([M+Na]$^+$, $C_{15}H_{10}{}^{79}Br^{35}ClNaS^+$ requires 358.9267).

The regiochemistry of **3t** was determined by the HMBC experiment.

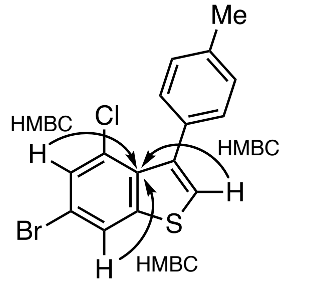

2,3-Dibutyl-8-(4-tolyl)benzo[1,2-*b*:3,4-*b′*]dithiophene (**3u**)

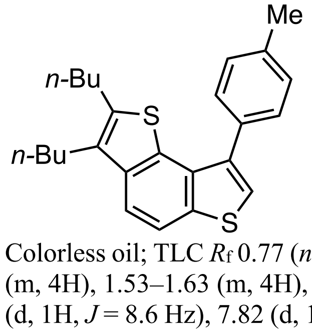

Colorless oil; TLC R_f 0.77 (*n*-hexane/CH_2Cl_2 = 1/1); ^{1}H NMR ($CDCl_3$, 500 MHz) δ 0.88–1.00 (m, 6H), 1.32–1.47 (m, 4H), 1.53–1.63 (m, 4H), 2.49 (s, 3H), 2.73–2.84 (m, 4H), 7.29–7.34 (m, 3H), 7.41–7.46 (AA'BB', 2H), 7.62 (d, 1H, J = 8.6 Hz), 7.82 (d, 1H, J = 8.6 Hz); ^{13}C NMR ($CDCl_3$, 126 MHz) δ 13.8 (1C), 14.0 (1C), 21.4 (1C), 22.5 (1C), 22.8 (1C), 26.4 (1C), 27.9 (1C), 32.4 (1C), 34.0 (1C), 118.4 (1C), 118.8 (1C), 124.2 (1C), 128.9 (2C), 129.9 (2C), 131.2 (1C), 132.5 (1C), 132.6 (1C), 133.3 (1C), 136.0 (1C), 137.8 (1C), 138.0 (1C), 138.2 (1C), 138.5 (1C); IR (KBr, cm^{-1}) 772, 814, 849, 1213, 1404, 1464, 2857, 2928, 2955; HRMS (ESI$^+$) *m*/*z* 415.1530 ([M+Na]$^+$, $C_{25}H_{28}NaS_2^+$ requires 415.1525).

The regiochemistry of **3u** was determined by the HMBC experiment.

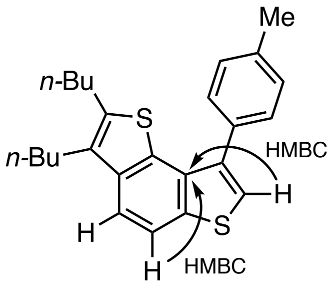

1-(4-Tolyl)phenanthro[3,4-*b*]thiophene (**3v**)

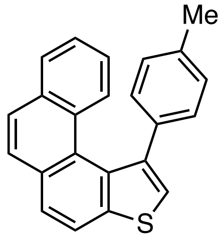

Colorless solid; Mp 174–176 ºC; TLC R_f 0.14 (*n*-hexane); ^{1}H NMR ($CDCl_3$, 500 MHz) δ 2.47 (s, 3H), 7.33–7.38 (AA'BB', 2H), 7.53–7.58 (AA'BB', 2H), 7.62 (s, 1H), 7.71 (ddd, 1H, *J* = 7.8, 7.8, 1.1 Hz), 7.84–7.90 (m, 2H), 7.91–7.95 (m, 2H), 8.05 (dd, 1H, *J* = 7.8, 1.1 Hz), 8.12 (d, 1H, *J* = 8.5 Hz), 9.29 (d, 1H, *J* = 8.5 Hz); ^{13}C NMR ($CDCl_3$, 126 MHz) δ 21.3 (1C), 122.2 (1C), 123.0 (1C), 126.0 (1C), 126.2 (1C), 126.3 (1C), 126.4 (1C), 126.88 (1C), 126.89 (1C), 127.7 (1C), 129.06 (1C), 129.11 (1C), 129.5 (2C), 129.6 (2C), 130.8 (1C), 132.9 (1C), 133.2 (1C), 135.8 (1C), 137.5 (1C), 137.7 (1C), 138.6 (1C); IR (KBr, cm^{-1}) 745, 773, 820, 835, 856, 1217, 1450, 2920; HRMS (ESI$^+$) *m*/*z* 347.0873 ([M+Na]$^+$, $C_{23}H_{16}NaS^+$ requires 347.0865).

The regiochemistry of **3v** was determined by the HMBC experiment.

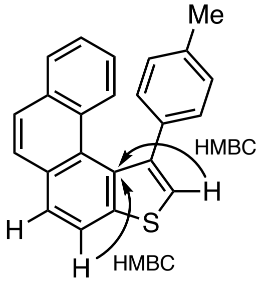

4-Chloro-3-(hydroxymethyl)benzo[*b*]thiophene (**3w**)

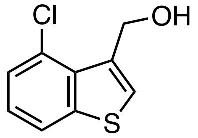

Colorless solid; Mp 116–118 ºC; TLC R_f 0.29 (*n*-hexane/EtOAc = 4/1); ^{1}H NMR ($CDCl_3$, 500 MHz) δ 2.23 (s, 1H), 5.14 (s, 2H), 7.26 (dd, 1H, *J* = 7.8, 7.8 Hz), 7.38 (dd, 1H, *J* = 7.8, 0.9 Hz), 7.52 (s, 1H), 7.76 (dd, 1H, *J* = 7.8, 0.9 Hz); ^{13}C NMR ($CDCl_3$, 126 MHz) δ 61.1 (1C), 121.9 (1C), 124.9 (1C), 125.8 (1C), 126.0 (1C), 128.2 (1C), 134.2 (1C), 136.7 (1C), 143.5 (1C); IR (KBr, cm^{-1}) 758, 822, 1061, 1078, 1200, 1404, 1452, 3240, 3258; HRMS (ESI$^+$) *m*/*z* 198.9978 ([M+H]$^+$, $C_9H_8{}^{35}ClOS^+$ requires 198.9979).

8-(2-Bromophenyl)-2,3-(dibutyl)benzo[1,2-*b*:3,4-*b'*]dithiophene (**3x**)

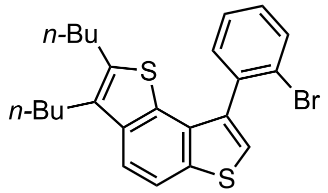

Yellow oil; TLC R_f 0.75 (*n*-hexane/CH_2Cl_2 = 1/1); ^{1}H NMR ($CDCl_3$, 500 MHz) δ 0.87–0.98 (m, 6H), 1.29–1.46 (m, 4H), 1.49–1.62 (m, 4H), 2.71–2.81 (m, 4H), 7.34–7.45 (m, 4H), 7.62 (d, 1H, *J* = 8.6 Hz), 7.75 (d, 1H, *J* = 7.8 Hz), 7.82 (d, 1H, *J* = 8.6 Hz); ^{13}C NMR ($CDCl_3$, 126 MHz) δ 13.8 (1C), 14.0 (1C), 22.5 (1C), 22.9 (1C), 26.4 (1C), 28.0 (1C), 32.4 (1C), 33.9 (1C), 118.6 (1C), 118.7 (1C), 124.8 (1C), 125.4 (1C), 127.2 (1C), 130.0 (1C), 131.3 (1C), 132.4 (1C+1C, two signals overlapped), 132.6 (1C), 132.7 (1C), 135.5 (1C), 136.0 (1C), 137.2 (1C), 138.0 (1C), 138.9 (1C); IR (KBr, cm^{-1}) 752, 800, 1302, 1408, 1431, 1462, 2857, 2930, 2955; HRMS (ESI$^+$) *m*/*z* 479.0459 ([M+Na]$^+$, $C_{24}H_{25}{}^{79}BrNaS_2^+$ requires 479.0473).

3-Chloro-2-ethoxy-1-((trifluoromethyl)sulfonyl)benzene (**4**)

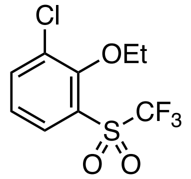

Yellow oil; TLC R_f 0.33 (*n*-hexane/EtOAc = 10/1); ^{1}H NMR ($CDCl_3$, 500 MHz) δ 1.50 (t, 3H, *J* = 7.0 Hz), 4.30 (q, 2H, *J* = 7.0 Hz), 7.31 (dd, 1H, *J* = 8.1, 8.1 Hz), 7.80 (dd, 1H, *J* = 8.1, 1.5 Hz), 7.94 (dd, 1H, *J* = 8.1, 1.5 Hz); ^{13}C NMR ($CDCl_3$, 126 MHz) δ 14.9 (1C), 71.9 (1C), 119.8 (q, 1C, $^1J_{C–F}$ = 326.6 Hz), 124.9 (1C), 128.5 (1C), 130.7 (1C), 131.3 (1C), 138.9 (1C), 156.0 (1C); ^{19}F NMR ($CDCl_3$, 376 MHz) δ –76.0 (s); IR (KBr, cm^{-1}) 600, 637, 1016, 1082, 1130, 1207, 1254, 1368, 1389, 1450; HRMS (ESI$^+$) *m*/*z* 288.9901 ([M+H]$^+$, $C_9H_9{}^{35}ClF_3O_3S^+$ requires 288.9908).

4-Chloro-3-(4-tolyl)-2-(4-tolylthio)benzo[*b*]thiophene (**5a**)

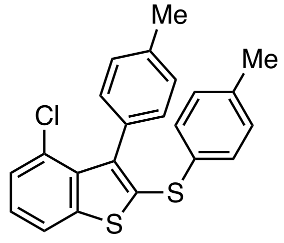

Colorless oil; TLC R_f 0.13 (*n*-hexane); ^{1}H NMR ($CDCl_3$, 400 MHz) δ 2.32 (s, 3H), 2.42 (s, 3H), 7.08–7.12 (AA'BB', 2H), 7.17 (dd, 1H, *J* = 7.8, 7.8 Hz), 7.21–7.23 (m, 4H), 7.24–7.28 (m, 3H), 7.59 (dd, 1H, *J* = 7.8, 1.0 Hz); ^{13}C NMR ($CDCl_3$, 101 MHz) δ 21.1 (1C), 21.5 (1C), 120.6 (1C), 124.7 (1C), 126.5 (1C), 128.4 (2C), 128.8 (1C), 130.0 (2C), 130.5 (2C), 131.5 (1C), 131.6 (2C), 132.9 (1C), 135.5 (1C), 137.0 (1C), 137.5 (1C), 138.1 (1C), 138.7 (1C), 142.2 (1C); IR (KBr, cm^{-1}) 762, 806, 845, 1105, 1206, 1395, 1443, 1489; HRMS (ESI$^+$) *m*/*z* 403.0345 ([M+Na]$^+$, $C_{22}H_{17}{}^{35}ClNaS_2^+$ requires 403.0352).

4-Chloro-2-iodo-3-(4-tolyl)benzo[*b*]thiophene (**5b**)

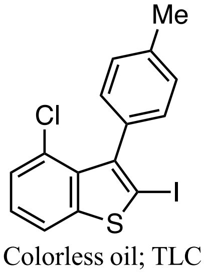

Colorless oil; TLC R_f 0.29 (*n*-hexane); ^{1}H NMR ($CDCl_3$, 500 MHz) δ 2.44 (s, 3H), 7.13–7.17 (AA'BB', 2H), 7.20 (dd, 1H, *J* = 7.8, 7.8 Hz), 7.23–7.27 (m, 3H), 7.68 (dd, 1H, *J* = 7.8, 1.1 Hz); ^{13}C NMR ($CDCl_3$, 126 MHz) δ 21.6 (1C), 85.6 (1C), 120.4 (1C), 125.0 (1C), 126.5 (1C), 128.6 (2C), 128.7 (1C), 130.5 (2C), 134.4 (1C), 135.1 (1C), 137.9 (1C), 143.2 (1C), 145.8 (1C); IR (KBr, cm^{-1}) 640, 743, 770, 839, 980, 1103, 1202, 1310, 1391, 1441; HRMS (ESI$^+$) *m*/*z* 406.9134 ([M+Na]$^+$, $C_{15}H_{10}{}^{35}ClINaS^+$ requires 406.9129).

4-Chloro-2-ethoxycarbonyl-3-(4-tolyl)benzo[*b*]thiophene (**5c**)

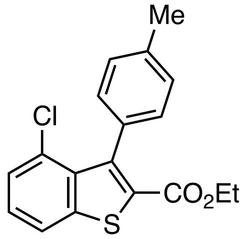

Colorless solid; Mp 84–86 ºC; TLC R_f 0.46 (*n*-hexane/CH_2Cl_2 = 1/1); ^{1}H NMR ($CDCl_3$, 400 MHz) δ 1.15 (t, 3H, *J* = 7.1 Hz), 2.43 (s, 3H), 4.17 (q, 2H, *J* = 7.1 Hz), 7.16–7.24 (m, 4H), 7.29–7.37 (m, 2H), 7.78 (dd, 1H, *J* = 7.4, 1.6 Hz); ^{13}C NMR ($CDCl_3$, 101 MHz) δ 13.8 (1C), 21.4 (1C), 61.2 (1C), 121.3 (1C), 127.0 (1C), 127.1 (1C), 128.0 (2C), 129.4 (2C), 130.7 (1C), 131.4 (1C), 133.1 (1C), 135.3 (1C), 137.3 (1C), 142.2 (1C), 143.6 (1C), 162.1 (1C); IR (KBr, cm^{-1}) 746, 775, 1076, 1198, 1240, 1263, 1339, 1697, 1728; HRMS (ESI$^+$) *m*/*z* 353.0380 ([M+Na]$^+$, $C_{18}H_{15}{}^{35}ClNaO_2S^+$ requires 353.0373).

4-Chloro-3-(4-tolyl)benzo[*b*]thiophene *S*-oxide (**6**)

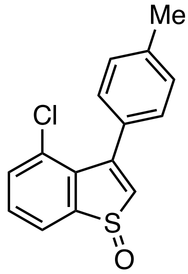

Colorless solid; Mp 117–119 ºC; TLC R_f 0.27 (*n*-hexane/EtOAc = 1/1); ^{1}H NMR ($CDCl_3$, 500 MHz) δ 2.42 (s, 3H), 6.89 (s, 1H), 7.20–7.25 (AA'BB', 2H), 7.26–7.31 (AA'BB', 2H), 7.39–7.47 (m, 2H), 7.88 (dd, 1H, *J* = 6.7, 1.7 Hz); ^{13}C NMR ($CDCl_3$, 126 MHz) δ 21.4 (1C), 125.1 (1C), 128.3 (2C), 128.7 (2C), 129.9 (1C), 130.7 (1C), 131.2 (1C), 133.5 (1C), 134.3 (1C), 135.5 (1C), 139.1 (1C), 148.9 (1C), 149.0 (1C); IR (KBr, cm^{-1}) 592, 748, 772, 779, 827, 1043, 1084, 1142, 1441, 1504; HRMS (ESI$^+$) *m*/*z* 297.0109 ([M+Na]$^+$, $C_{15}H_{11}{}^{35}ClNaOS^+$ requires 297.0111).

4-Chloro-2-(2-hydroxyphenyl)-3-(4-tolyl)benzo[*b*]thiophene (**7**)

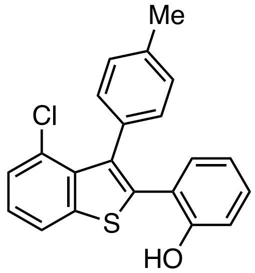

Pale yellow oil; TLC R_f 0.31 (*n*-hexane/CH_2Cl_2 = 1/1); ^{1}H NMR ($CDCl_3$, 500 MHz) δ 2.32 (s, 3H), 5.06 (s, 1H), 6.80 (d, 1H, *J* = 7.8 Hz), 6.85 (ddd, 1H, *J* = 7.6, 7.6, 0.9 Hz), 7.04–7.09 (AA'BB', 2H), 7.12–7.15 (AA'BB', 2H), 7.16–7.22 (m, 2H), 7.29 (dd, 1H, *J* = 7.8, 7.8 Hz), 7.36 (dd, 1H, *J* = 7.6, 0.9 Hz), 7.80 (dd, 1H, *J* = 7.6, 0.9 Hz); ^{13}C NMR ($CDCl_3$, 126 MHz) δ 21.3 (1C), 115.9 (1C), 119.8 (1C), 120.3 (1C), 121.0 (1C), 125.1 (1C), 126.9 (1C), 128.3 (2C), 129.6 (1C), 130.4 (1C), 130.5 (2C), 132.0 (1C), 132.1 (1C), 135.3 (1C), 136.3 (1C), 136.6 (1C), 137.4 (1C), 141.8 (1C), 153.1 (1C); IR (KBr, cm^{-1}) 824, 1105, 1150, 1179, 1194, 1229, 1288, 3528; HRMS (ESI$^+$) *m*/*z* 373.0422 ([M+Na]$^+$, $C_{21}H_{15}{}^{35}ClNaOS^+$ requires 373.0424).

4-Chlorobenzo[*b*]thiophene-3-carboxylic acid (**9**)

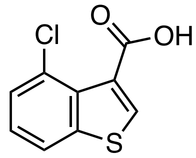

Colorless solid; Mp 185–187 ºC; TLC R_f 0.37 (EtOAc); ^{1}H NMR ($CDCl_3$, 500 MHz) δ 7.36 (dd, 1H, J = 7.8, 7.8 Hz), 7.50 (d, 1H, J = 7.8 Hz), 7.80 (d, 1H, J = 7.8 Hz), 8.18 (s, 1H); ^{13}C NMR ($CDCl_3$, 126 MHz) δ 121.4 (1C), 125.9 (1C), 127.3 (1C), 128.4 (1C), 129.2 (1C), 133.2 (1C), 134.8 (1C), 142.1 (1C), 168.2 (1C); IR (KBr, cm^{-1}) 750, 1204, 1248, 1281, 1456, 1674, 1709, 3105; HRMS (ESI$^+$) *m/z* 234.9597 ([M+Na]$^+$, $C_9H_5{}^{35}ClNaO_2S^+$ requires 234.9591).

4-Chloro-3-iodobenzo[*b*]thiophene (**10**)

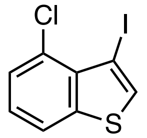

Colorless solid; Mp 99–100 ºC; TLC R_f 0.47 (*n*-hexane); ^{1}H NMR ($CDCl_3$, 500 MHz) δ 7.26 (dd, 1H, J = 7.8, 7.8 Hz), 7.39 (dd, 1H, J = 7.8, 0.9 Hz), 7.72 (s, 1H), 7.81 (dd, 1H, J = 7.8, 0.9 Hz); ^{13}C NMR ($CDCl_3$, 126 MHz) δ 71.5 (1C), 121.7 (1C), 125.4 (1C), 127.0 (1C), 129.5 (1C), 132.8 (1C), 133.3 (1C), 140.5 (1C); IR (KBr, cm^{-1}) 727, 764, 835, 1094, 1198, 1273, 1391, 1435; HRMS (ESI$^+$) *m/z* 316.8659 ([M+Na]$^+$, $C_8H_4{}^{35}ClINaS^+$ requires 316.8659).

6-Bromo-4-chloro-2-(4-methoxyphenyl)-3-(4-tolyl)benzo[*b*]thiophene (**11**)

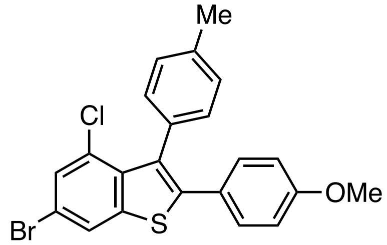

Colorless oil; TLC R_f 0.62 (*n*-hexane/CH_2Cl_2 = 1/1); ^{1}H NMR ($CDCl_3$, 500 MHz) δ 2.38 (s, 3H), 3.75 (s, 3H), 6.71–6.75 (AA'BB', 2H), 7.10–7.18 (m, 6H), 7.42 (d, 1H, J = 1.8 Hz), 7.87 (d, 1H, J = 1.8 Hz); ^{13}C NMR ($CDCl_3$, 126 MHz) δ 21.4 (1C), 55.2 (1C), 113.7 (2C), 116.9 (1C), 123.2 (1C), 125.9 (1C), 128.5 (2C), 129.3 (1C), 129.9 (1C), 130.8 (2C), 131.2 (2C), 132.4 (1C), 133.0 (1C), 135.3 (1C), 137.2 (1C), 141.4 (1C), 141.8 (1C), 159.3 (1C); IR (KBr, cm^{-1}) 829, 1034, 1179, 1252, 1292, 1423, 1487, 1514, 1607; HRMS (ESI$^+$) *m/z* 464.9676 ([M+Na]$^+$, $C_{22}H_{16}{}^{79}Br^{35}ClNaOS^+$ requires 464.9686).

2-(4-Methoxyphenyl)-4-phenyl-3-(4-tolyl)-6-(4-(trifluoromethyl)phenyl)benzo[*b*]thiophene (**12**)

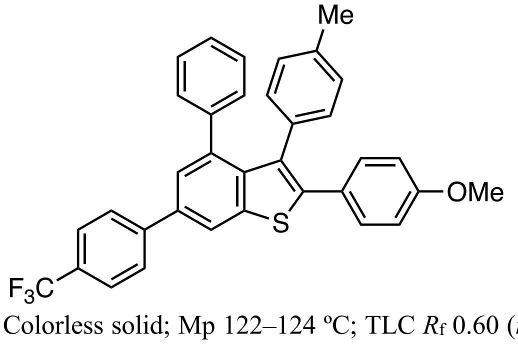

Colorless solid; Mp 122–124 ºC; TLC R_f 0.60 (*n*-hexane/CH_2Cl_2 = 1/1); ^{1}H NMR ($CDCl_3$, 500 MHz) δ 2.19 (s, 3H), 3.76 (s, 3H), 6.60–6.68 (m, 4H), 6.72–6.77 (AA'BB', 2H), 6.88–6.95 (m, 4H), 6.99–7.04 (AA'BB'C, 1H), 7.11–7.17 (AA'BB', 2H), 7.48 (d, 1H, J = 1.7 Hz), 7.68–7.74 (AA'BB', 2H), 7.79–7.85 (AA'BB', 2H), 8.12 (d, 1H, J = 1.7 Hz); ^{13}C NMR ($CDCl_3$, 126 MHz) δ 21.1 (1C), 55.2 (1C), 113.6 (2C), 119.6 (1C), 124.3 (q, 1C, $^1J_{C–F}$ = 272.0 Hz), 125.6 (1C), 125.8 (q, 2C, $^3J_{C–F}$ = 3.6 Hz), 126.8 (1C), 126.9 (2C), 127.1 (1C), 127.5 (2C), 128.1 (2C), 129.2 (q, 1C, $^2J_{C–F}$ = 31.4 Hz), 129.4 (2C), 130.4 (2C), 131.0 (2C), 133.1 (1C), 133.4 (1C), 134.8 (1C), 135.6 (1C), 137.1 (1C), 139.7 (1C), 140.0 (1C), 140.7 (1C), 141.0 (1C), 144.2 (1C), 159.1 (1C); ^{19}F NMR ($CDCl_3$, 376 MHz) δ –62.6 (s); IR (KBr, cm^{-1}) 835, 1072, 1113, 1125, 1165, 1179, 1252, 1325; HRMS (ESI$^+$) *m*/*z* 573.1466 ([M+Na]$^+$, $C_{35}H_{25}F_3NaOS^+$ requires 573.1470).

8-(2-Bromophenyl)-2,3-dibutyl-7-iodobenzo[1,2-*b*:3,4-*b'*]dithiophene (**13**)

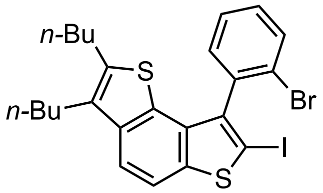

Pale yellow oil; TLC R_f 0.75 (*n*-hexane/CH_2Cl_2 = 1/1); ^{1}H NMR ($CDCl_3$, 400 MHz) δ 0.86–0.97 (m, 6H), 1.28–1.46 (m, 4H), 1.47–1.61 (m, 4H), 2.68–2.79 (m, 4H), 7.33 (dd, 1H, J = 7.6, 1.5 Hz), 7.41–7.52 (m, 2H), 7.56 (d, 1H, J = 8.6 Hz), 7.71 (d, 1H, J = 8.6 Hz), 7.79 (dd, 1H, J = 7.6, 1.5 Hz); ^{13}C NMR ($CDCl_3$, 101 MHz) δ 13.8 (1C), 14.0 (1C), 22.5 (1C), 22.9 (1C), 26.4 (1C), 27.9 (1C), 32.4 (1C), 33.8 (1C), 81.9 (1C), 117.5 (1C), 118.6 (1C), 125.4 (1C), 127.7 (1C), 130.6 (1C), 131.3 (1C), 131.9 (1C), 132.3 (1C), 132.5 (1C), 133.0 (1C), 137.6 (1C), 138.2 (1C), 139.2 (1C), 139.3 (1C), 142.0 (1C); IR (KBr, cm^{-1}) 725, 756, 799, 1406, 1458, 2857, 2928, 2953; HRMS (ESI$^+$) *m*/*z* 604.9442 ([M+Na]$^+$, $C_{24}H_{24}{}^{79}BrINaS_2^+$ requires 604.9440).

2,3-Dibutyl-7-(4-tolyl)-7*H*-thieno[3",2":5',6']benzo[1',2':4,5]thieno[2,3-*b*]indole (**14**)

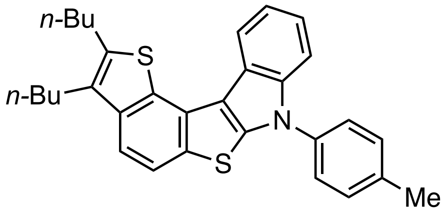

Colorless solid; Mp 137–139 ºC; TLC R_f 0.75 (*n*-hexane/CH_2Cl_2 = 1/1); ^{1}H NMR ($CDCl_3$, 500 MHz) δ 0.95–1.05 (m, 6H), 1.42–1.55 (m, 4H), 1.65 (tt, 2H, J = 7.7, 7.7 Hz), 1.83 (tt, 2H, J = 7.7, 7.7 Hz), 2.47 (s, 3H), 2.86 (t, 2H, J = 7.7 Hz), 2.99 (t, 2H, J = 7.7 Hz), 7.30 (dd, 1H, J = 8.0, 8.0 Hz), 7.35–7.42 (m, 3H), 7.52–7.61 (m, 4H), 7.70 (d, 1H, J = 8.5 Hz), 8.78 (d, 1H, J = 8.0 Hz); ^{13}C NMR ($CDCl_3$, 126 MHz) δ 14.0 (1C), 14.1 (1C), 21.2 (1C), 22.6 (1C), 22.9 (1C), 26.6 (1C), 28.3 (1C), 32.4 (1C), 34.0 (1C), 110.4 (1C), 116.4 (1C), 118.1 (1C), 119.4 (1C), 120.6 (1C), 120.7 (1C), 122.2 (1C), 122.9 (1C), 124.3 (2C), 127.2 (1C), 130.61 (2C), 130.65 (1C), 132.18 (1C), 132.23 (1C), 135.8 (1C), 137.5 (1C), 138.35 (1C), 138.41 (1C), 141.4 (1C), 143.4 (1C); IR (KBr, cm^{-1}) 739, 756, 1404, 1450, 1481, 1514, 2857, 2928, 2953; HRMS (ESI$^+$) *m*/*z* 504.1800 ([M+Na]$^+$, $C_{31}H_{31}NNaS_2^+$ requires 504.1790).

References for Supporting Information

S1 S. Yoshida, Y. Sugimura, Y. Hazama, Y. Nishiyama, T. Yano, S. Shimizu and T. Hosoya, *Chem. Commun.*, 2015, **51**, 16613.

S2 V. Girijavallabhan, C. Alvarez and F. G. Njoroge, *J. Org. Chem.*, 2011, **76**, 6442.

S3 Y. Zhang, P. Ji, W. Hu, Y. Wei, H. Huang and W. Wang, *Chem. Eur. J.*, 2019, **25**, 8225.

S4 A. Dermenci, R. E. Whittaker and G. Dong, *Org. Lett*., 2013, **15**, 2242.

S5 J. M. Medina, J. L. Mackey, N. K. Garg and K. N. Houk, *J. Am. Chem. Soc.*, 2014, **136**, 15798.

S6 S. Yoshida, Y. Nakamura, K. Uchida, Y. Hazama and T. Hosoya, *Org. Lett.*, 2016, **18**, 6212.

S7 S. Yoshida, Y. Hazama, K. Kanemoto, Y. Nakamura and T. Hosoya, *Chem. Lett.*, 2019, **48**, 742.

S8 S. Yoshida, T. Kuribara, T. Morita, T. Matsuzawa, K. Morimoto, T. Kobayashi and T. Hosoya, *RSC Adv.*, 2018, **8**, 21754.

S9 D. E. Bergbreiter and E. Pendergrass, *J. Org. Chem*., 1981, **46**, 219.

S10 K. Kanemoto, S. Yoshida and T. Hosoya, *Org. Lett.*, 2019, **21**, 3172.

S11 Z. He, H. J. Shrives, J. A. Fernández-Salas, A. Abengózar, J. Neufeld, K. Yang, A. P. Pulis and D. J. Procter, *Angew. Chem., Int. Ed.*, 2018, **57**, 5759.

S12 R. Das and D. Chakraborty, *Appl. Organometal. Chem.*, 2011, **25**, 437.

S13 G. J. P. Perry, J. M. Quibell, A. Panigrahi, and I. Larrosa, *J. Am. Chem. Soc.*, 2017, **139**, 11527.

S14 C. Colletto, A. Panigrahi, J. Fernández-Casado and I. Larrosa, *J. Am. Chem. Soc.*, 2018, **140**, 9638.

S15 C. J. Narangoda, T. R. Lex, M. A. Moore, C. D. McMillen, A. Kitaygorodskiy, J. E. Jackson and D. C. Whitehead, *Org. Lett*., 2018, **20**, 8009.

S16 Z. Ni, S. Wang, H. Mao and Y. Pan, *Tetrahedron Lett.*, 2012, **53**, 3907.

S17 R. J. Reddy, M. P. Ball-Jones and P. W. Davies, *Angew. Chem., Int. Ed.*, 2017, **56**, 13310.

S18 X.-Q. Zhu, Q. Sun, Z.-X. Zhang, B. Zhou, P.-X. Xie, W.-B. Shen, X. Lu, J.-M. Zhou and L.-W. Ye, *Chem. Commun.*, 2018, **54**, 7435.

S19 Q. Su, Z.-J. Zhao, F. Xu, P.-C. Lou, K. Zhang, D.-X. Xie, L. Shi, Q.-Y. Cai, Z.-H. Peng and D.-L. An, *Eur. J. Org. Chem.*, **2013**, 1551.

S20 C. Colletto, S. Islam, F. Juliá-Hernández and I. Larrosa, *J. Am. Chem. Soc.*, 2016, **138**, 1677.

S21 A. R. Katritzky, K. Kirichenko, Y. Ji and I. Prakash, *Chem. Heterocycl. Compd.*, 2002, **38**, 156.

S22 S. Sakai, K. Sato and K. Yoshida, *Tetrahedron Lett.*, 2020, **61**, 151476.

S23 K. Kobayashi, M. Horiuchi, S. Fukamachi and H. Konishi, *Tetrahedron Lett.*, 2009, **65**, 2430.

S24 K. Kobayashi, D. Nakai, S. Fukamachi and H. Konishi, *Heterocycles*, 2010, **81**, 1703.

^{1}H and ^{13}C NMR Spectra of Compounds

^{1}H NMR (500 MHz) and ^{13}C NMR (126 MHz) spectra of isopropyl (4-tolyl)ethynyl sulfide ($CDCl_3$)

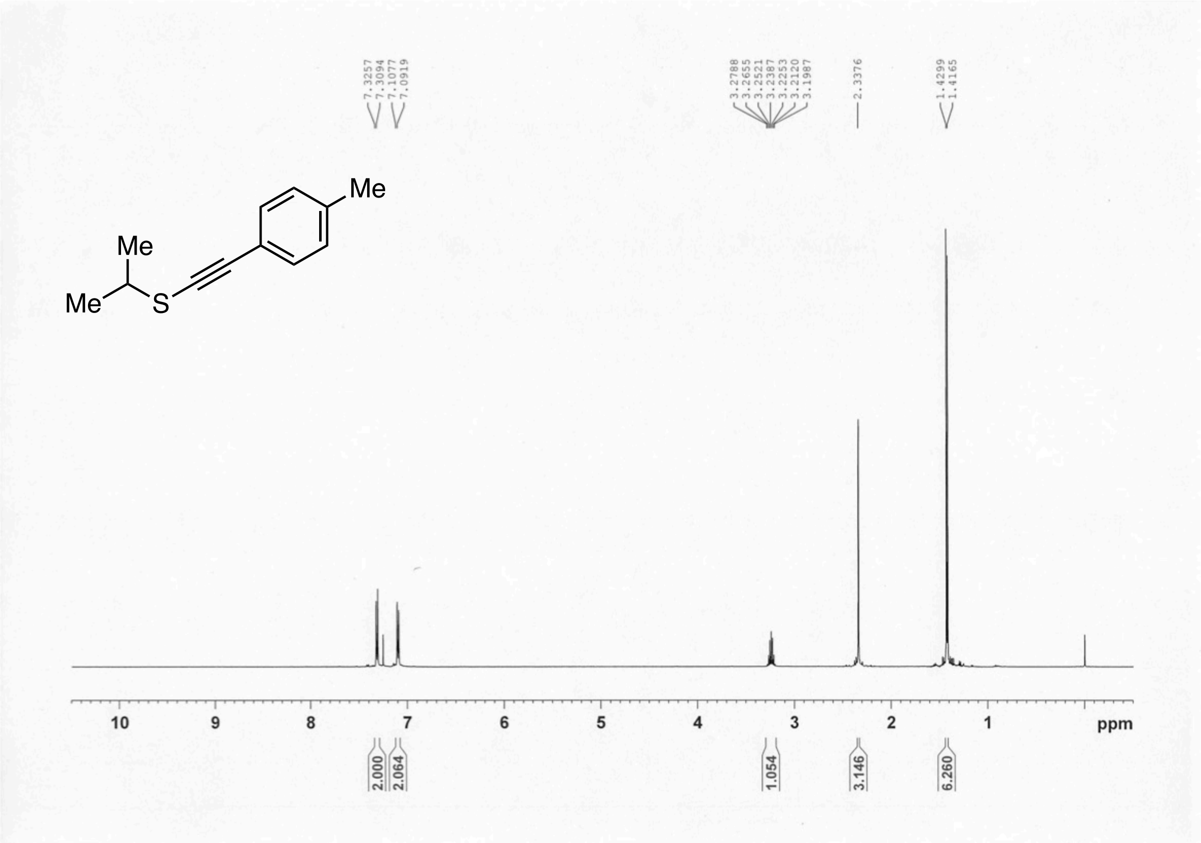

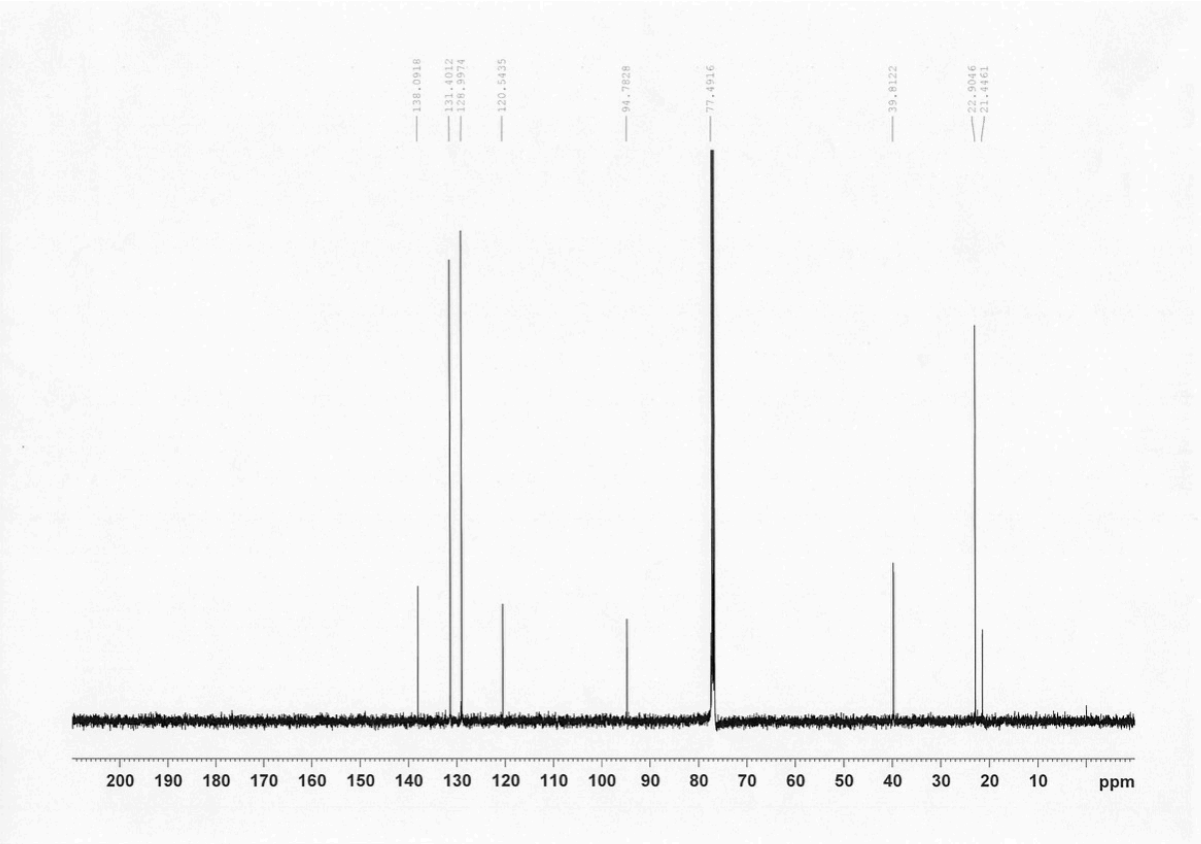

^{1}H NMR (500 MHz) and ^{13}C NMR (126 MHz) spectra of benzyl (4-tolyl)ethynyl sulfide ($CDCl_3$)

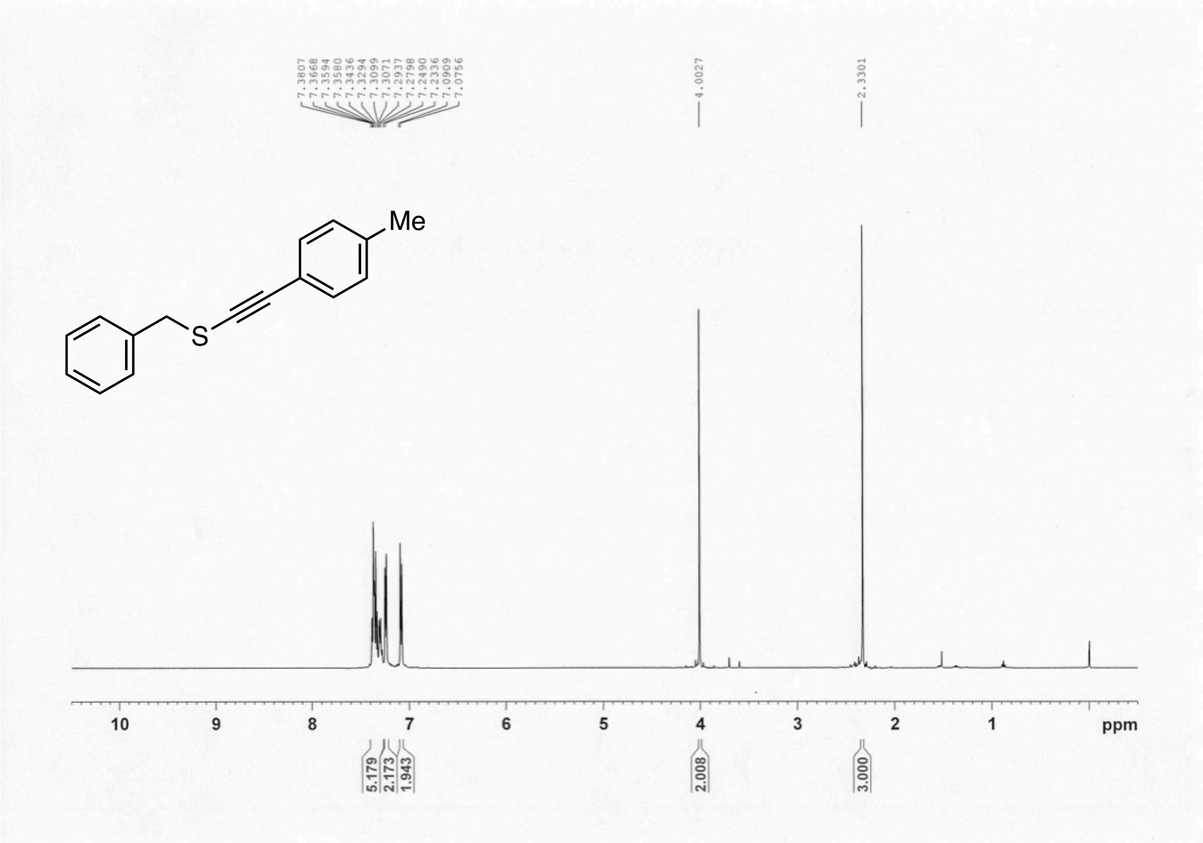

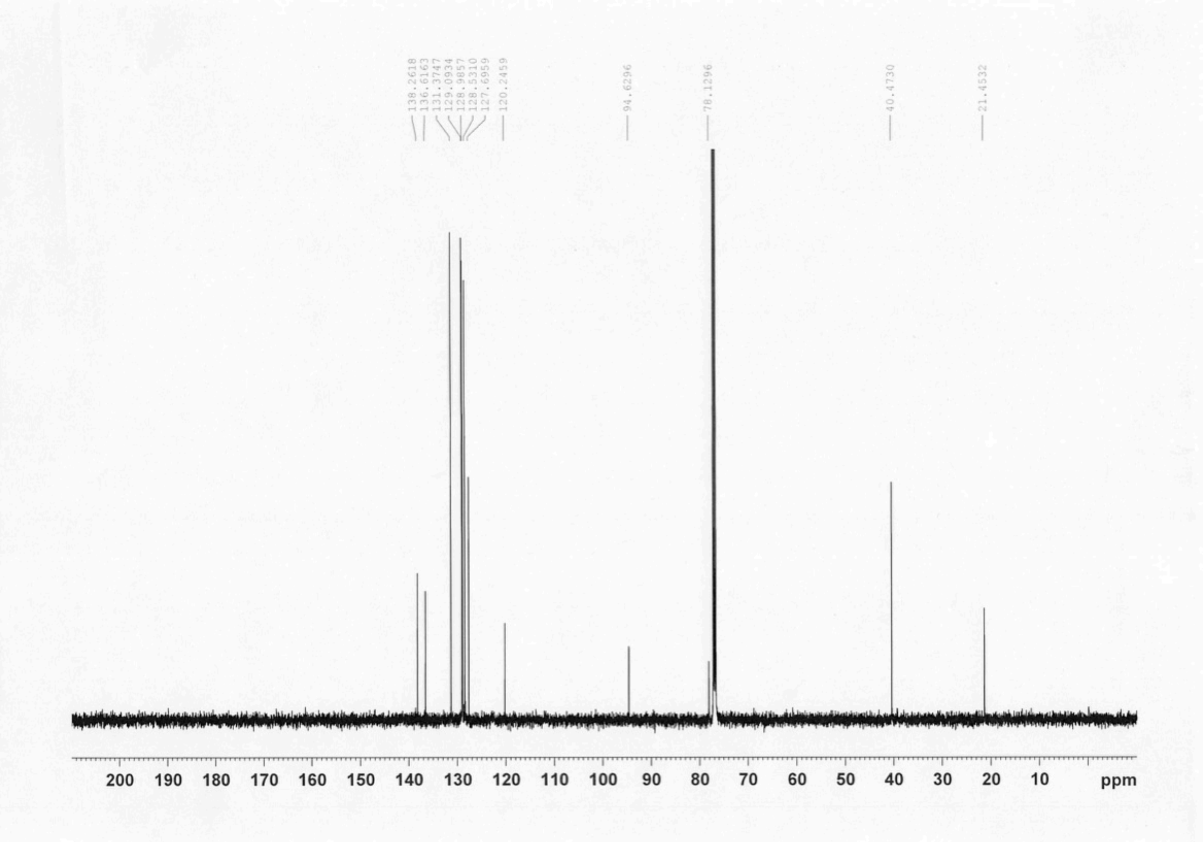

^{1}H NMR (500 MHz) and ^{13}C NMR (126 MHz) spectra of ethyl (4-methoxycarbonylphenyl)ethynyl sulfide (**2d**) ($CDCl_3$)

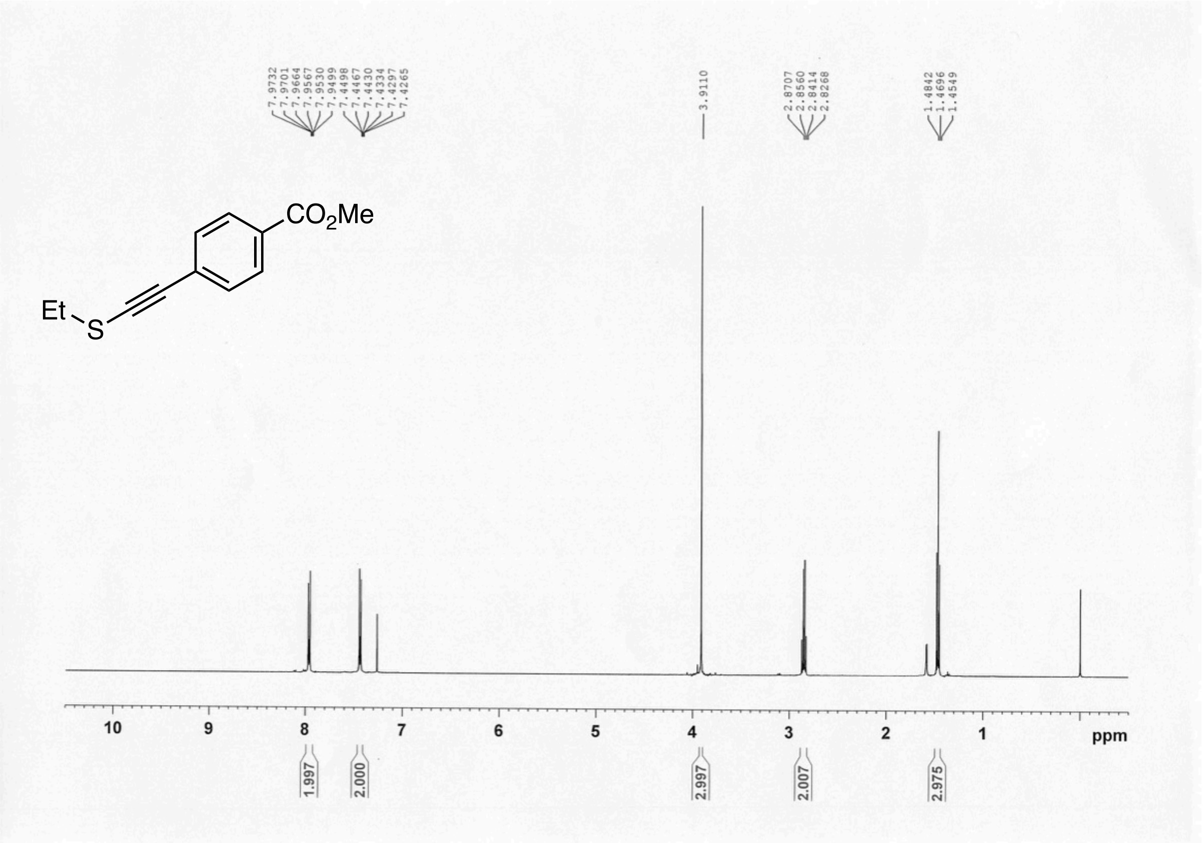

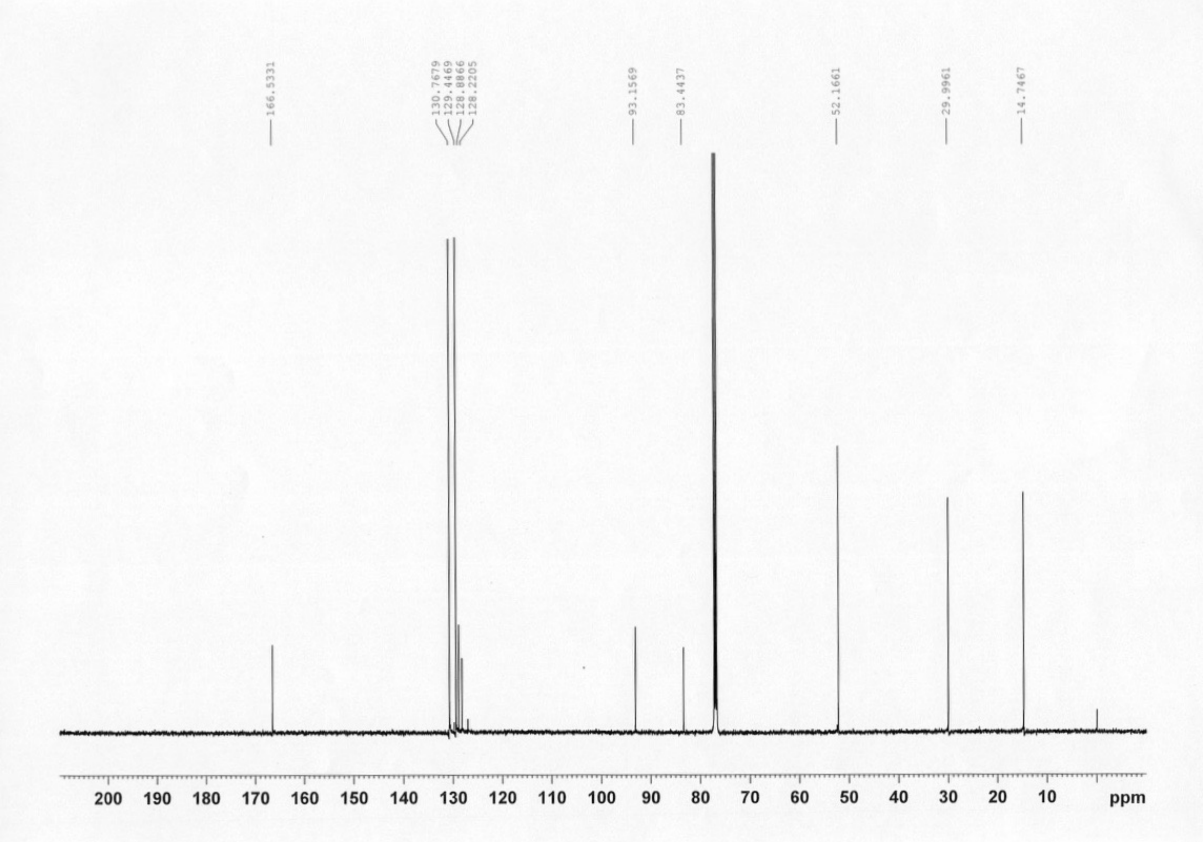

^{1}H NMR (500 MHz) and ^{13}C NMR (126 MHz) spectra of (2-bromophenyl)ethynyl ethyl sulfide (**2e**) ($CDCl_3$)

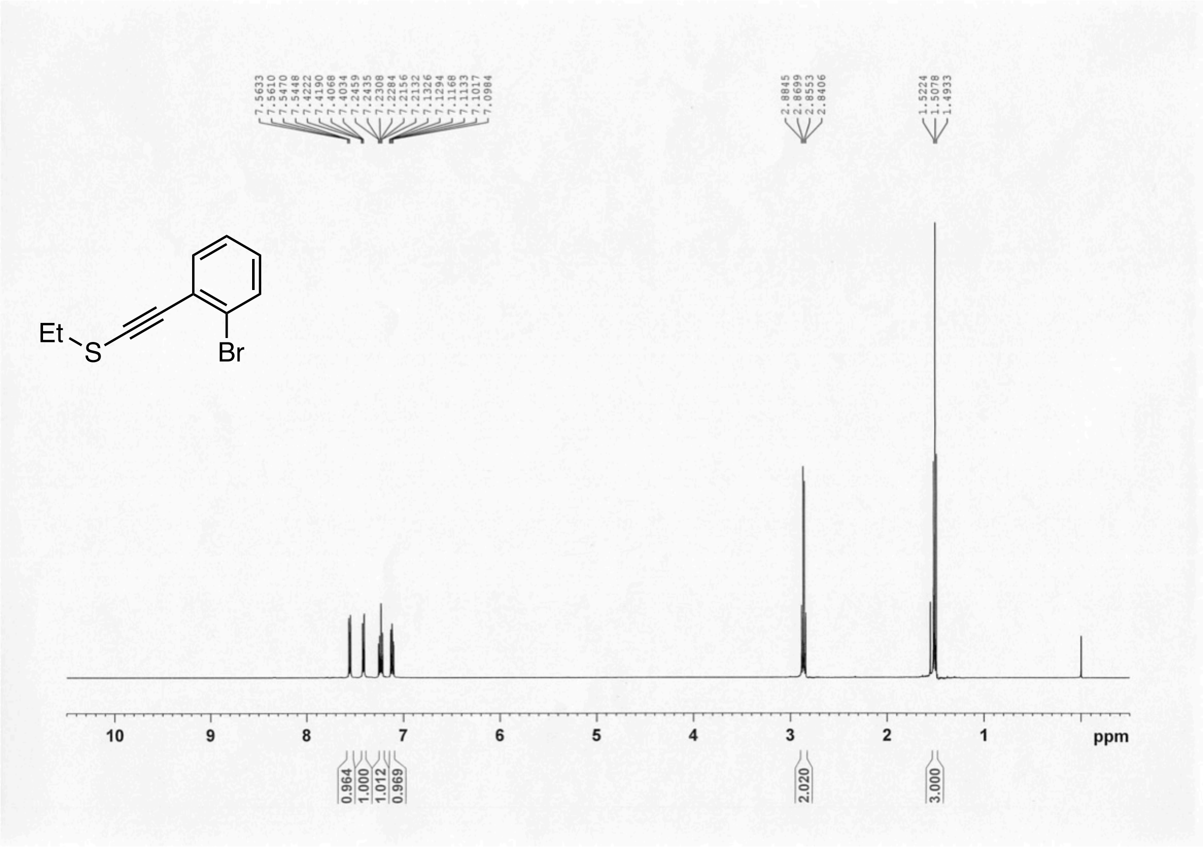

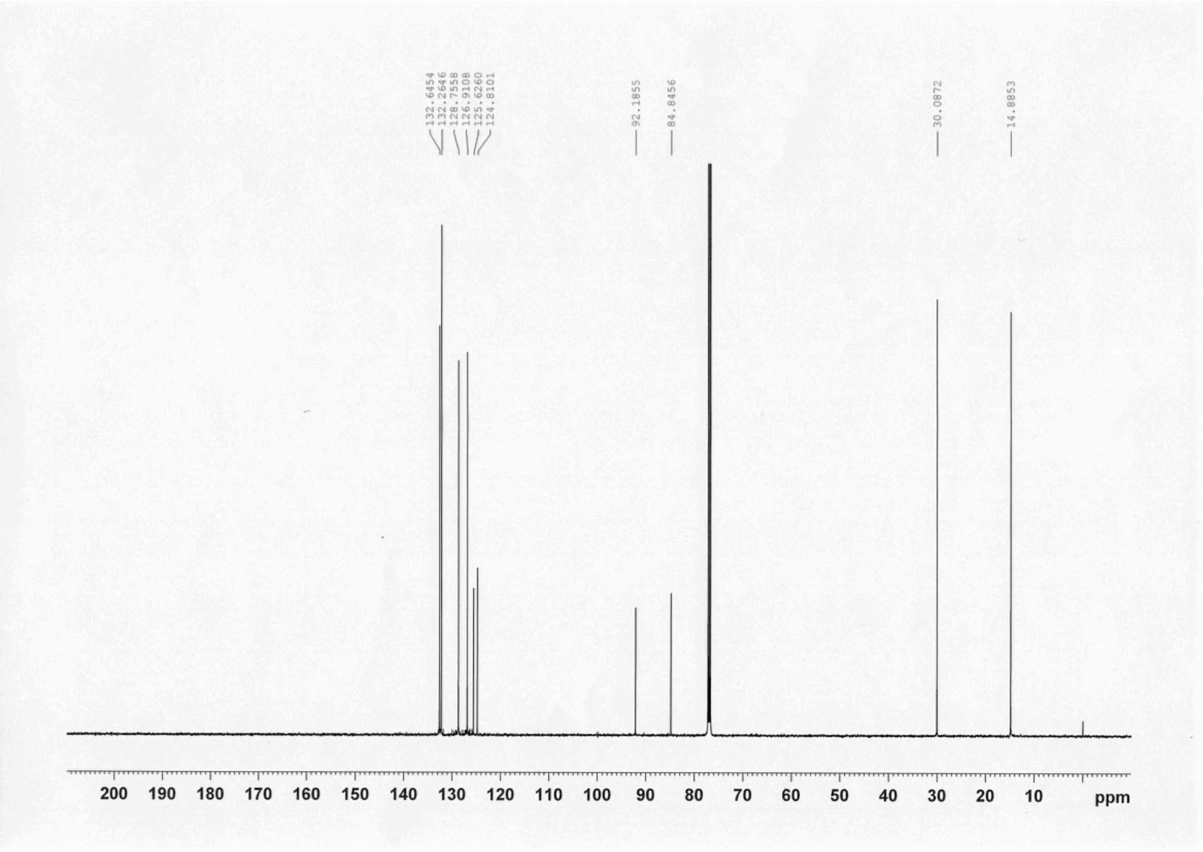

^{1}H NMR (500 MHz) and ^{13}C NMR (126 MHz) spectra of ethyl (9-phenanthrenyl)ethynyl sulfide (**2g**) ($CDCl_3$)

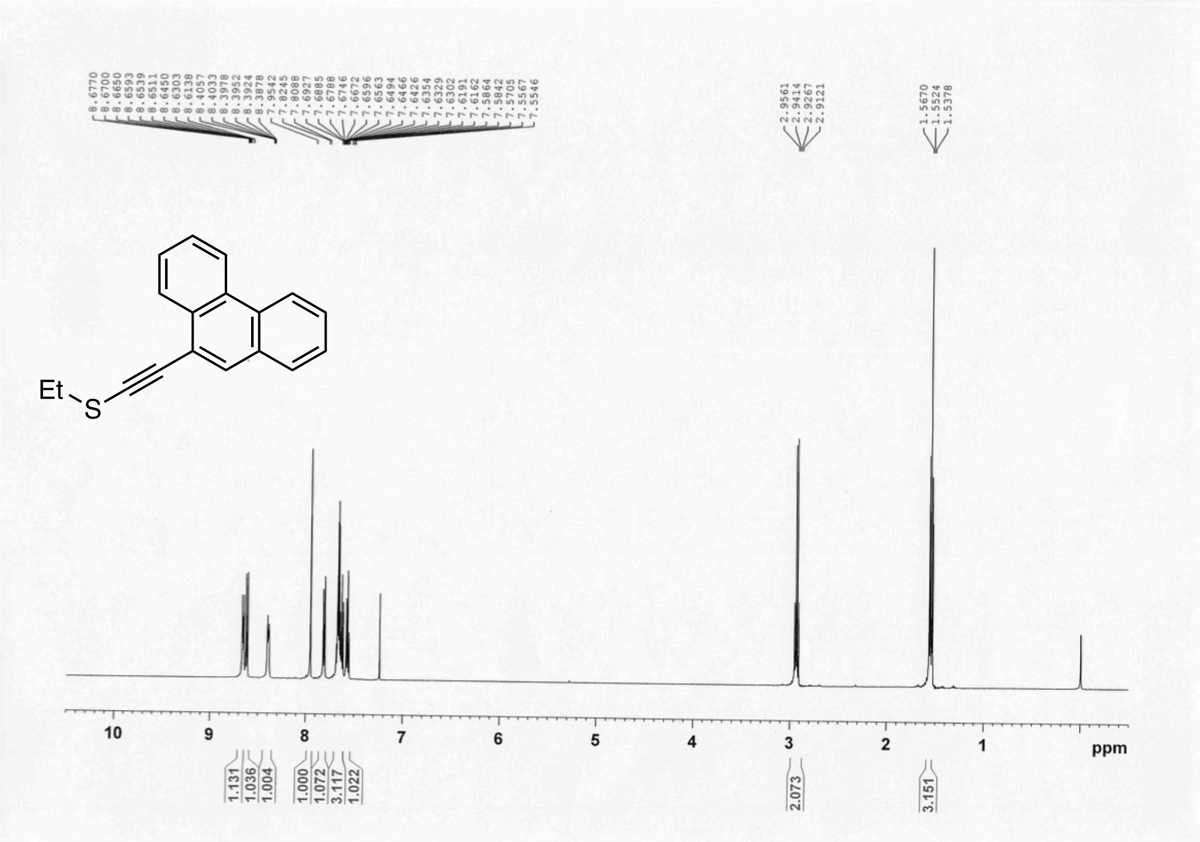

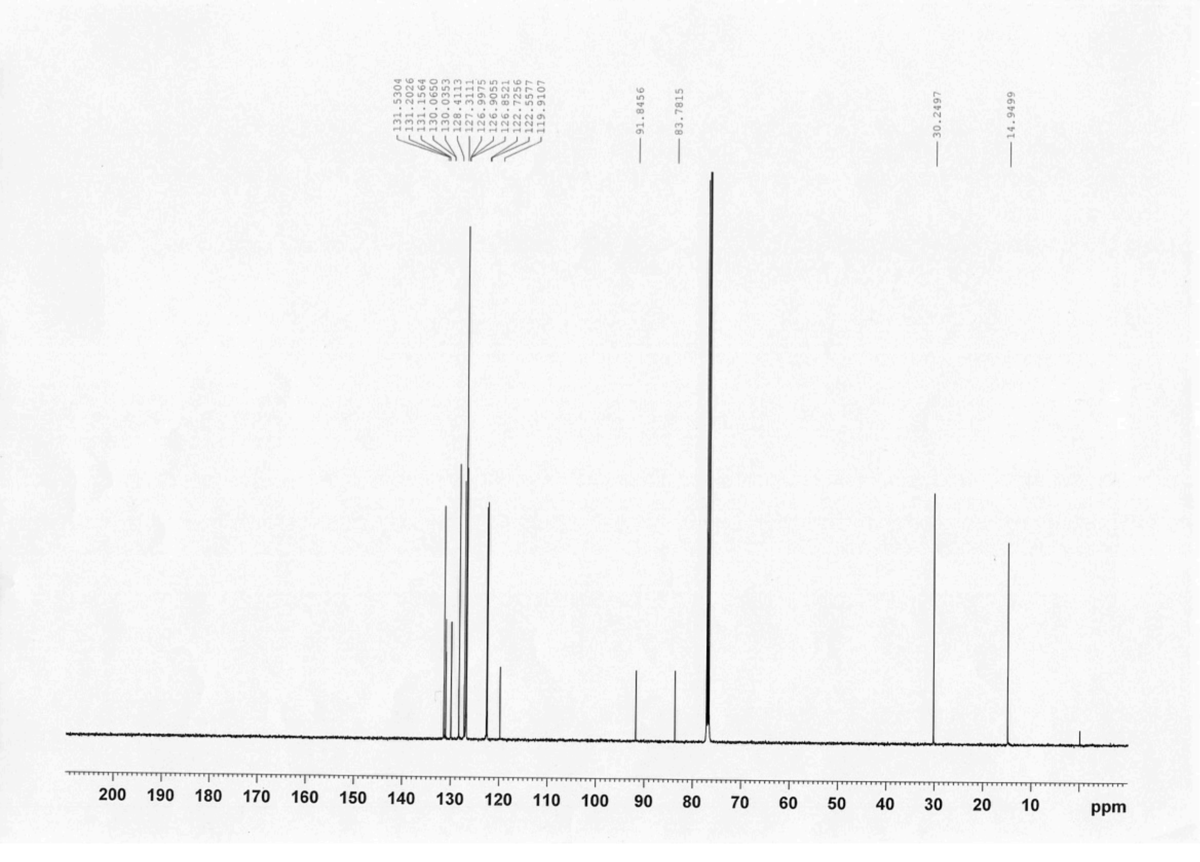

^{1}H NMR (500 MHz) and ^{13}C NMR (126 MHz) spectra of ethyl (3-thienyl)ethynyl sulfide (**2h**) ($CDCl_3$)

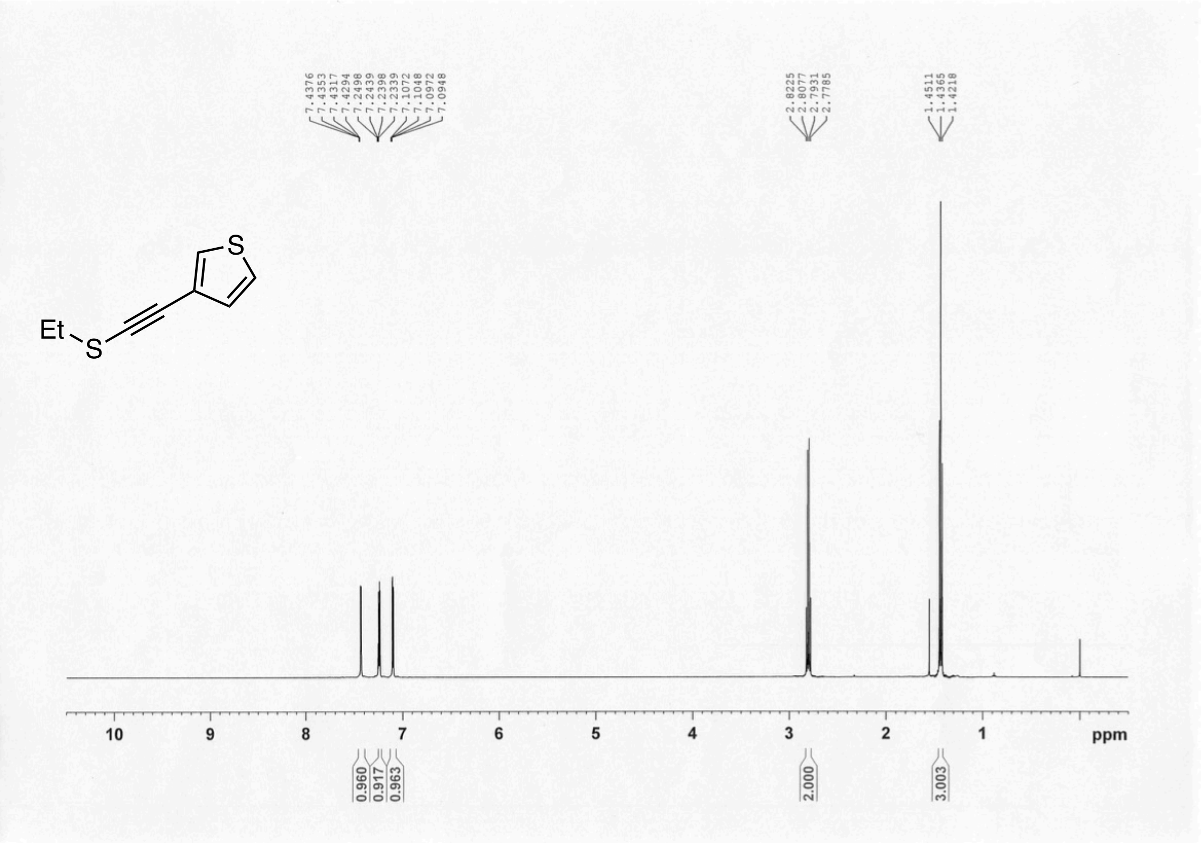

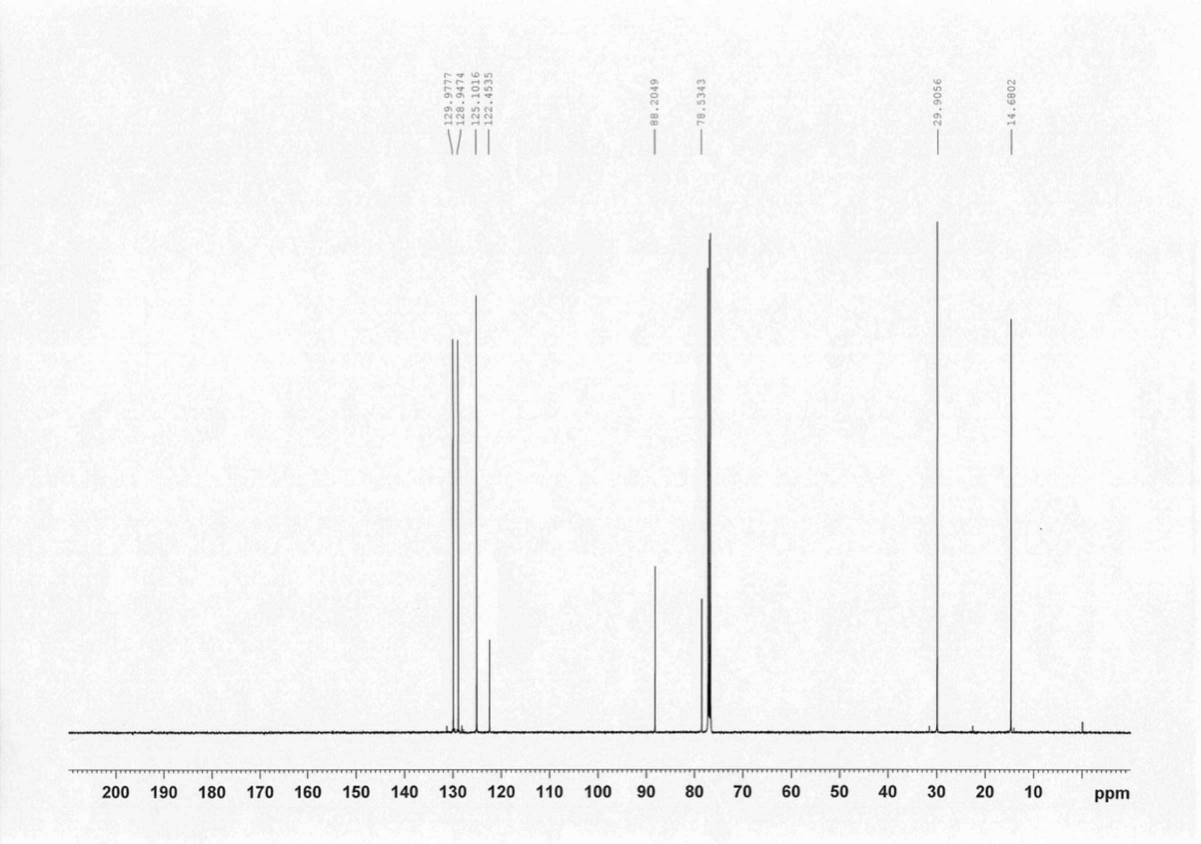

^{1}H NMR (500 MHz) and ^{13}C NMR (126 MHz) spectra of ethyl (2-phenethyl)ethynyl sulfide (**2i**) ($CDCl_3$)

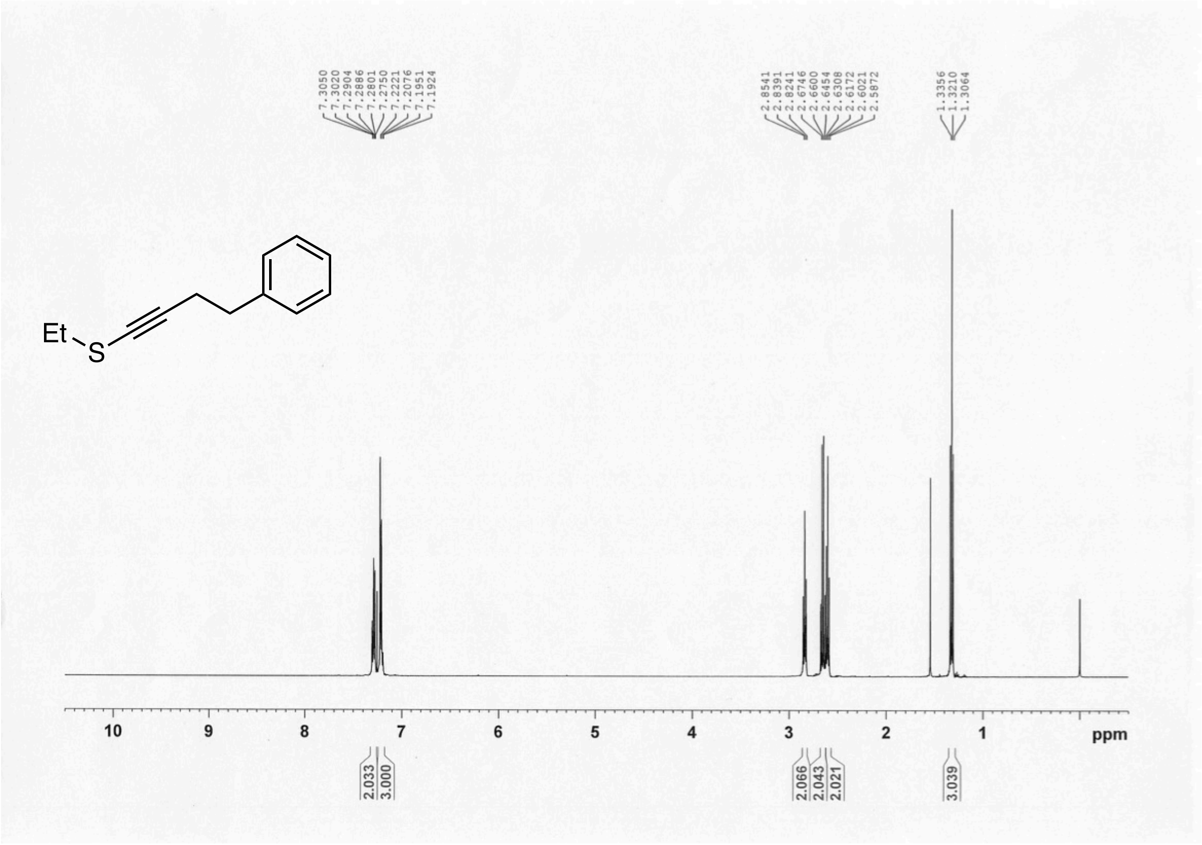

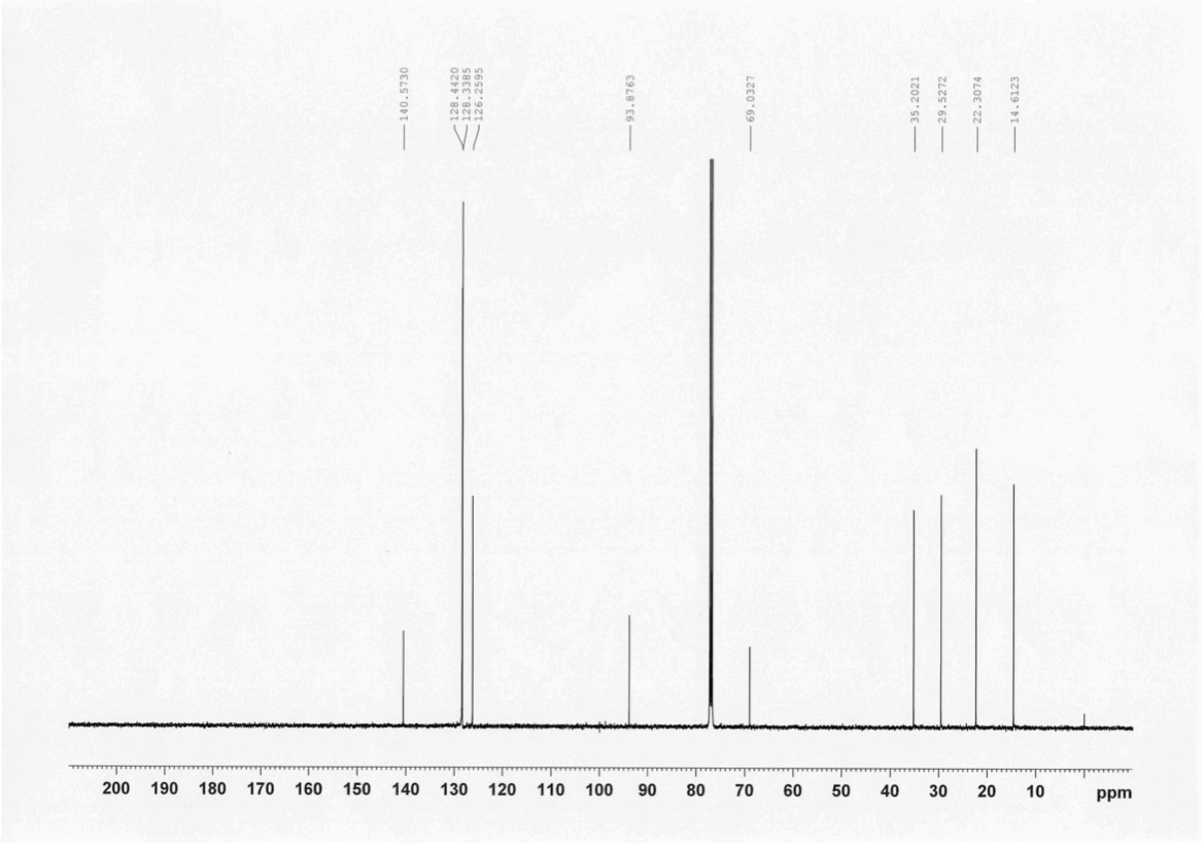

^{1}H NMR (500 MHz) and ^{13}C NMR (126 MHz) spectra of cyclohexylethynyl ethyl sulfide (**2j**) ($CDCl_3$)

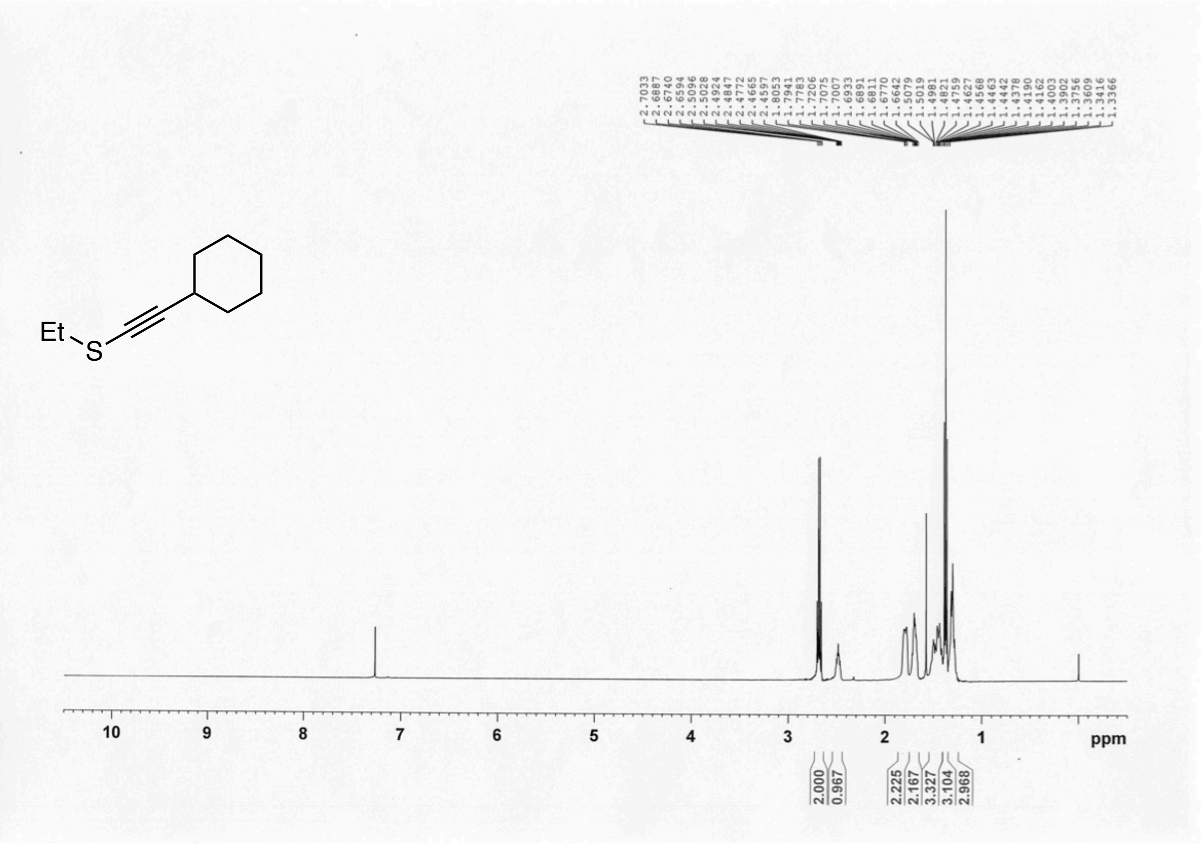

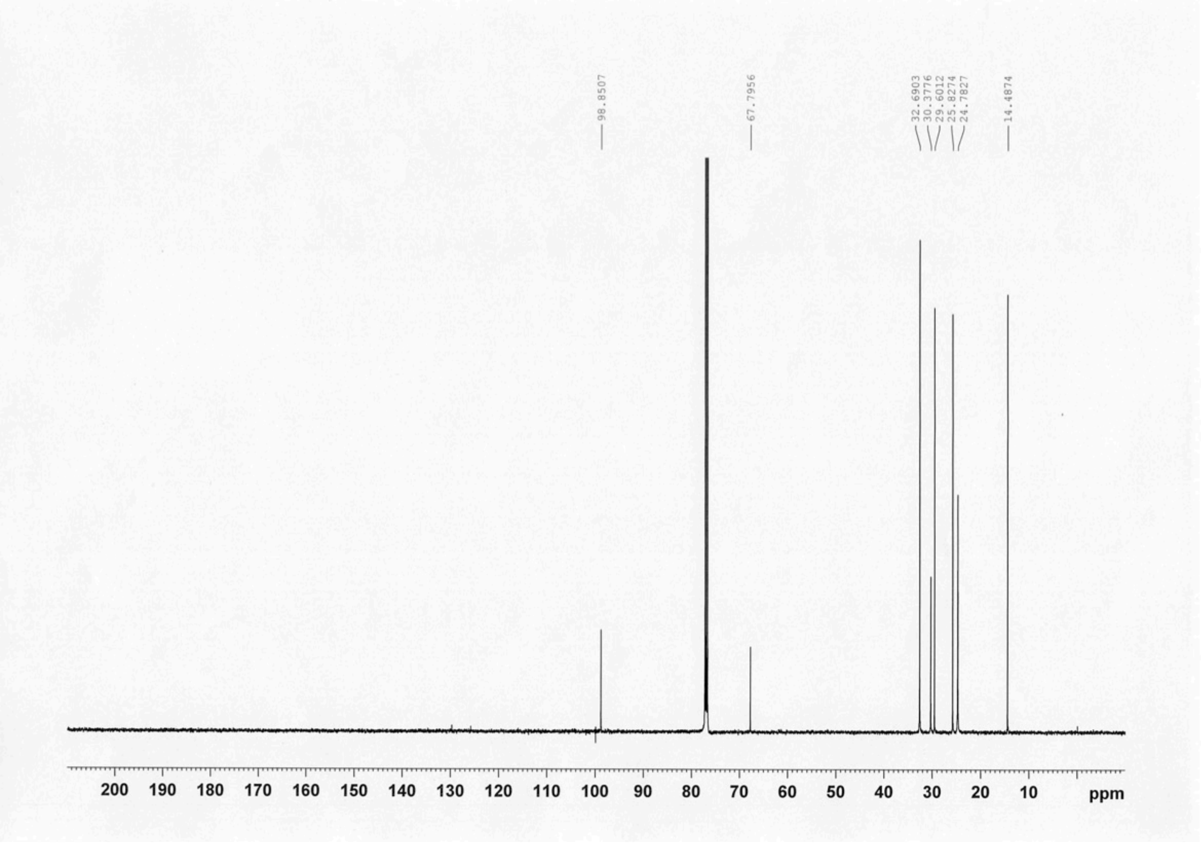

^{1}H NMR (500 MHz) and ^{13}C NMR (126 MHz) spectra of 3,17-di-*O*-methyl-17-(ethylthioethynyl)estradiol (**2k**) ($CDCl_3$)

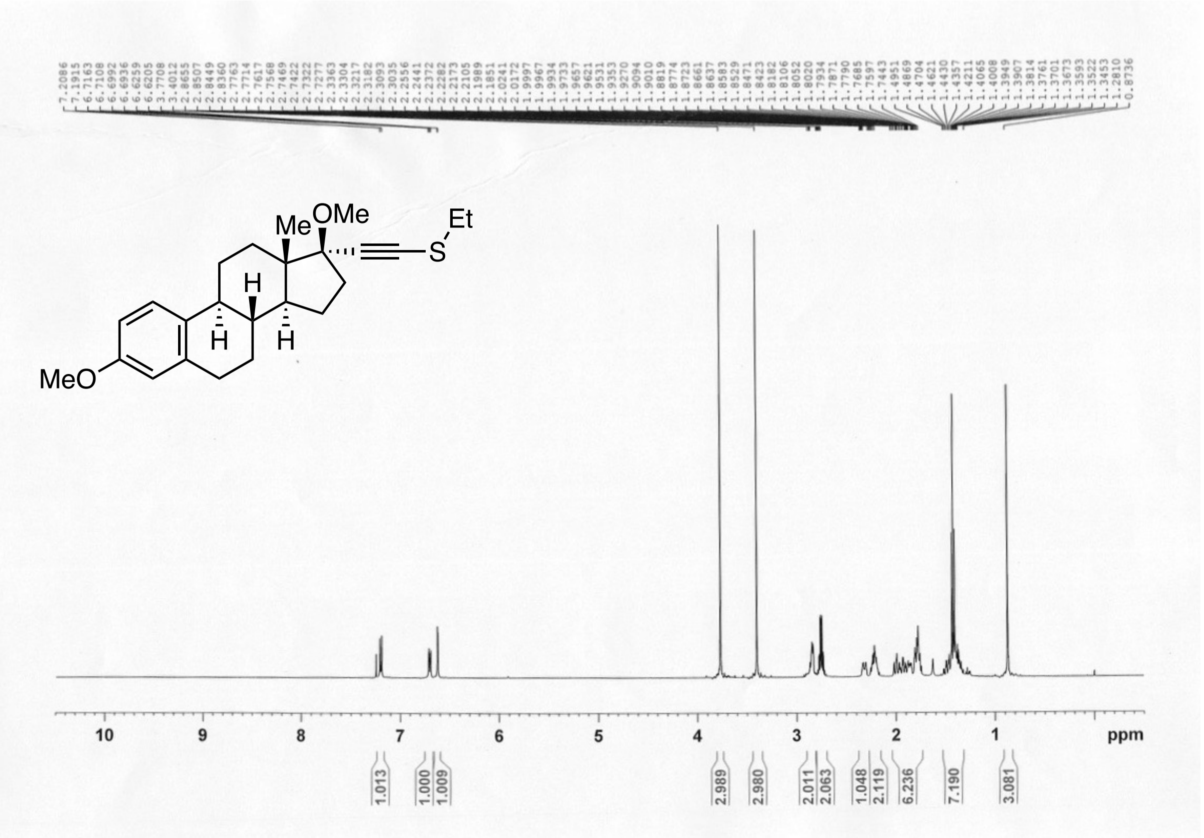

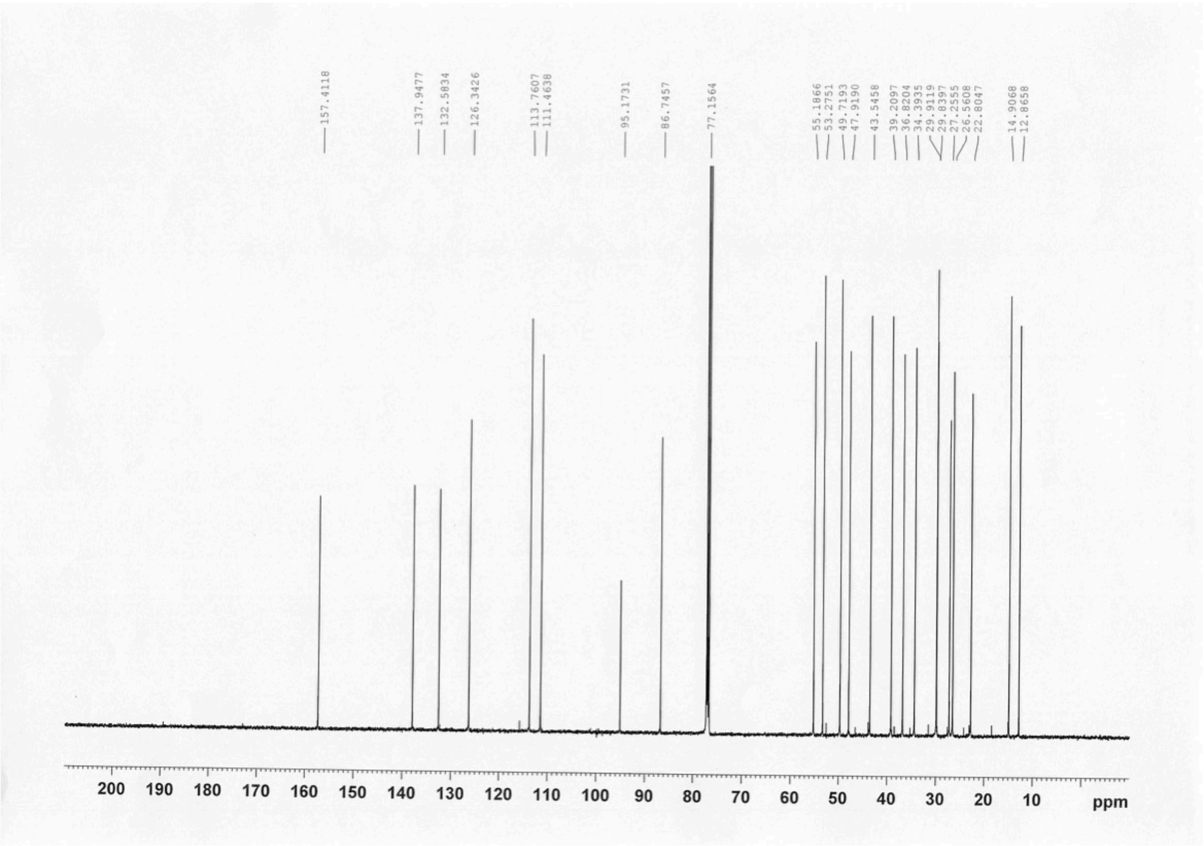

^{1}H NMR (500 MHz) and ^{13}C NMR (126 MHz) spectra of 2-((3-(ethylthio)prop-2-yn-1-yl)oxy)tetrahydro-2*H*-pyran (**2l**) ($CDCl_3$)

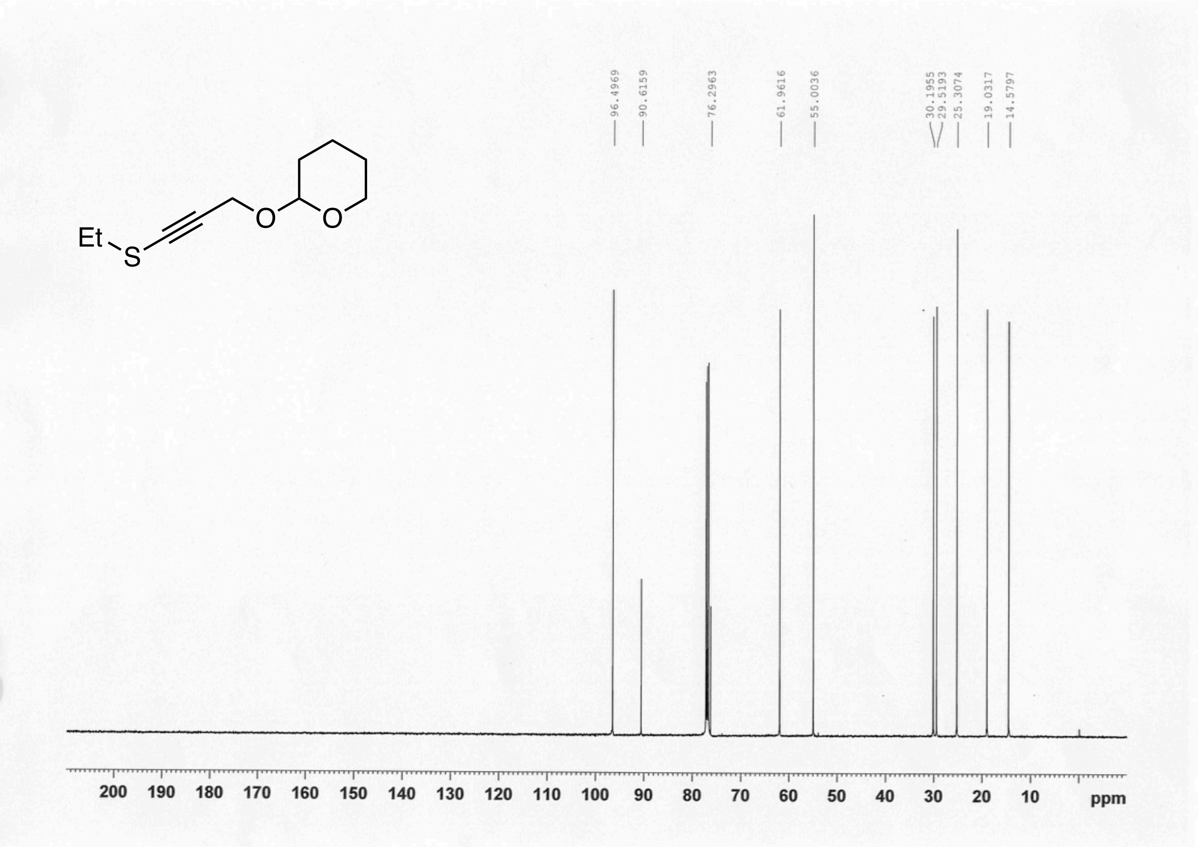

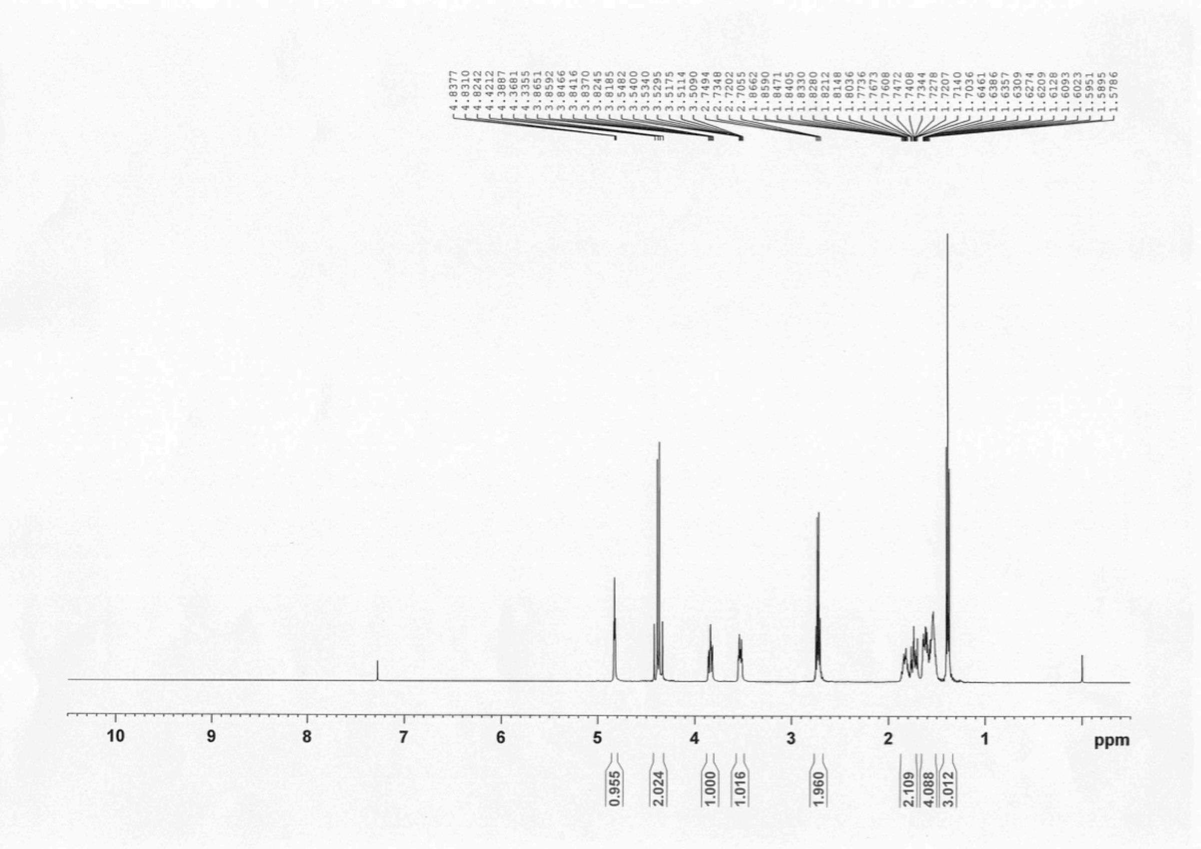

^{1}H NMR (500 MHz, DMSO-d_6) and ^{13}C NMR (126 MHz, $CDCl_3$) spectra of 4-chloro-3-(4-tolyl)benzo[*b*]thiophene (**3a**)

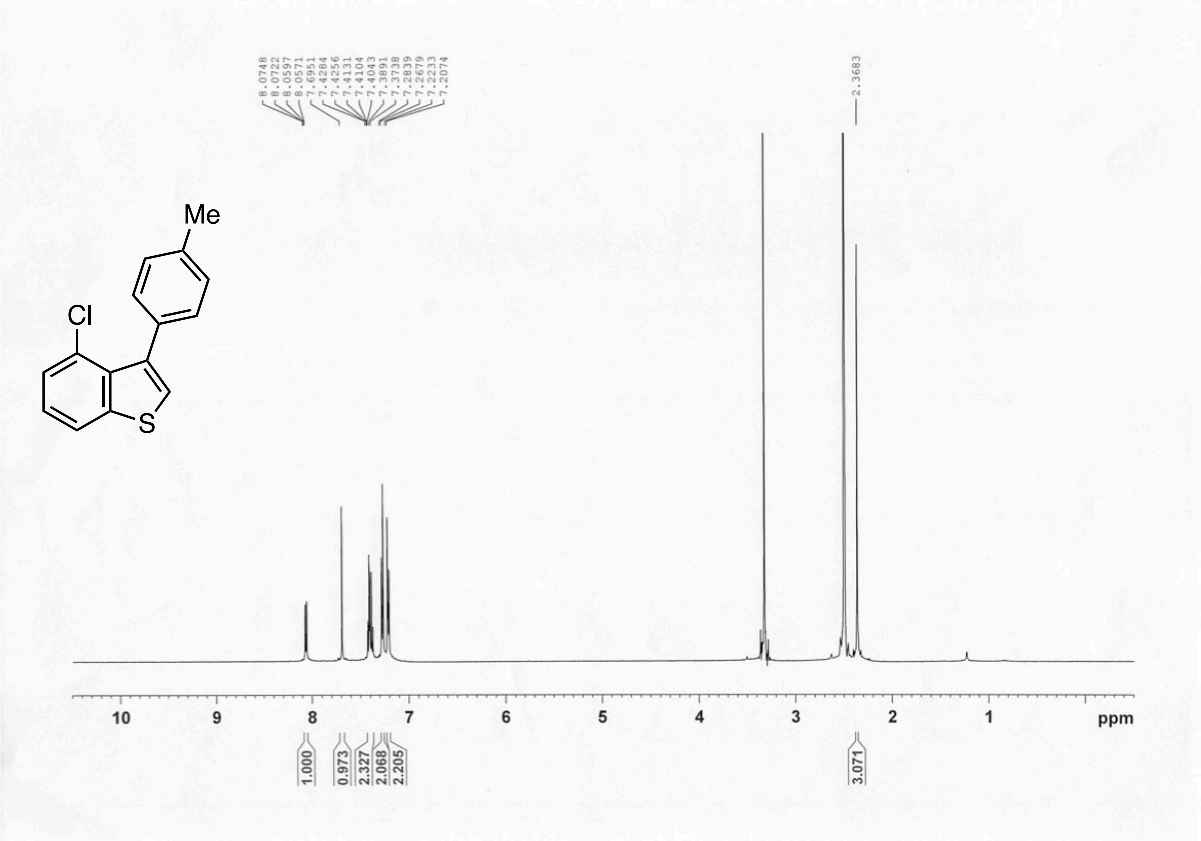

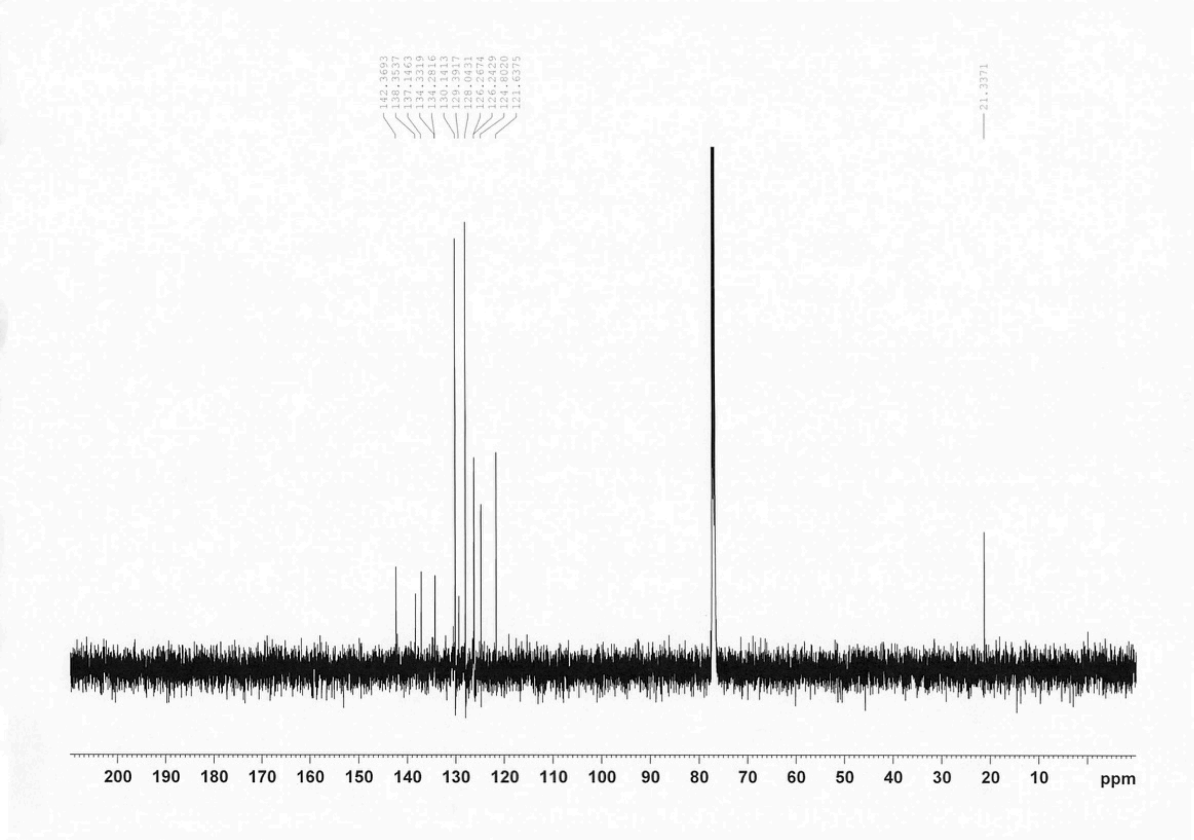

^{1}H NMR (500 MHz) and ^{13}C NMR (126 MHz) spectra of 4-chloro-3-(4-methoxyphenyl)benzo[*b*]thiophene (**3b**) ($CDCl_3$)

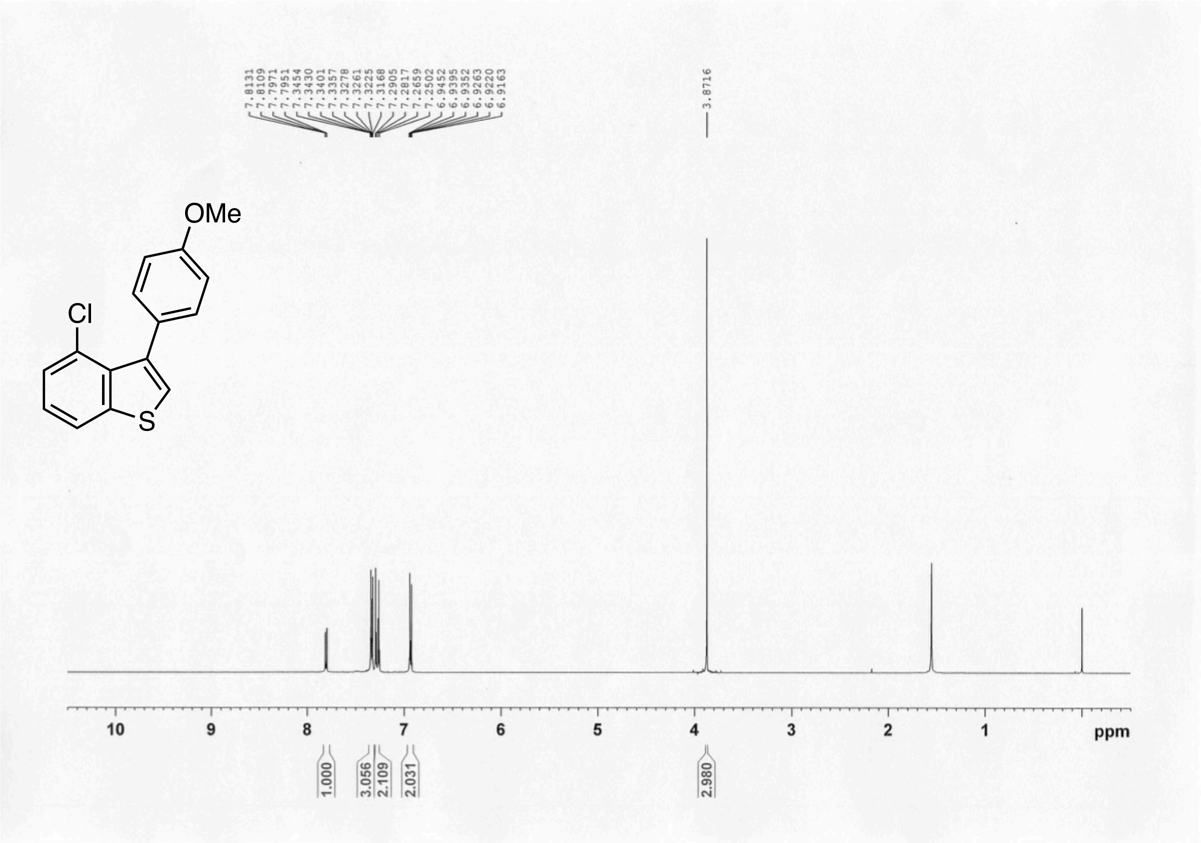

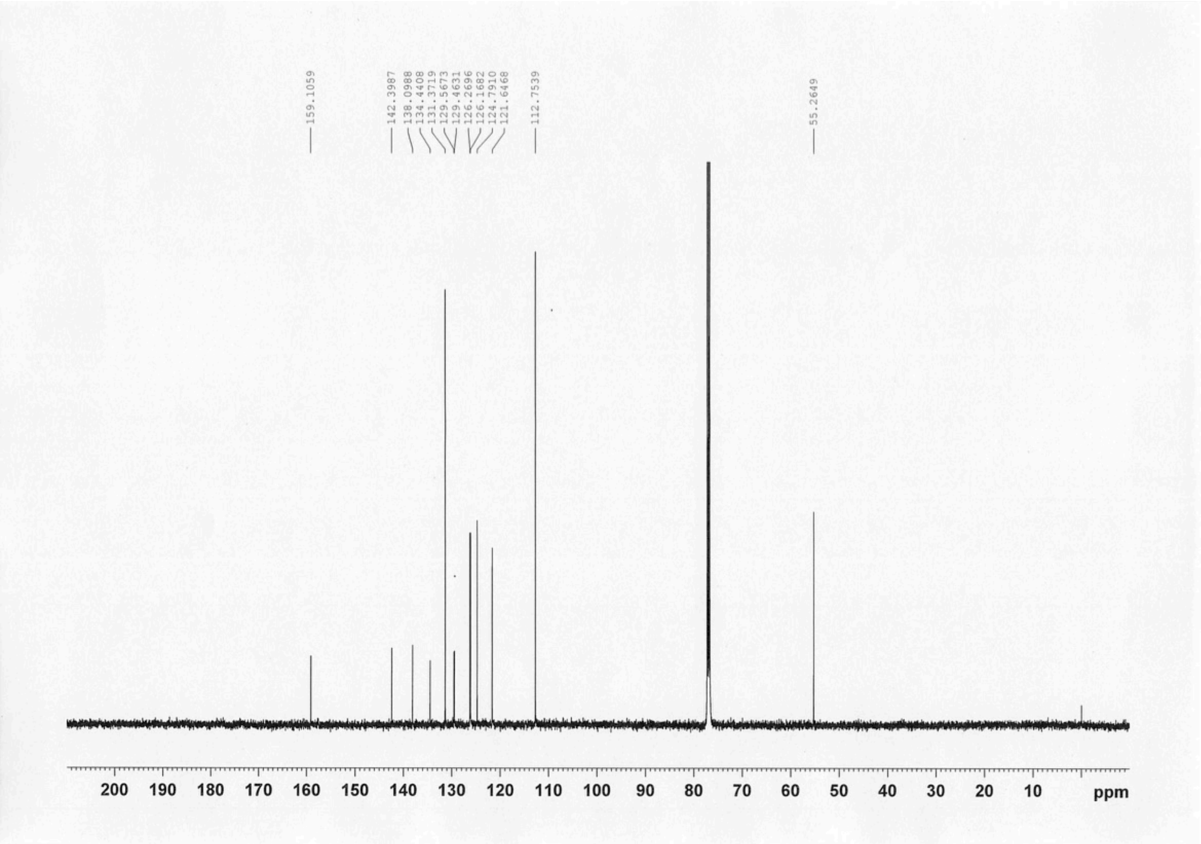

^{1}H NMR (500 MHz) and ^{13}C NMR (126 MHz) spectra of 4-chloro-3-(4-chlorophenyl)benzo[*b*]thiophene (**3c**) ($CDCl_3$)

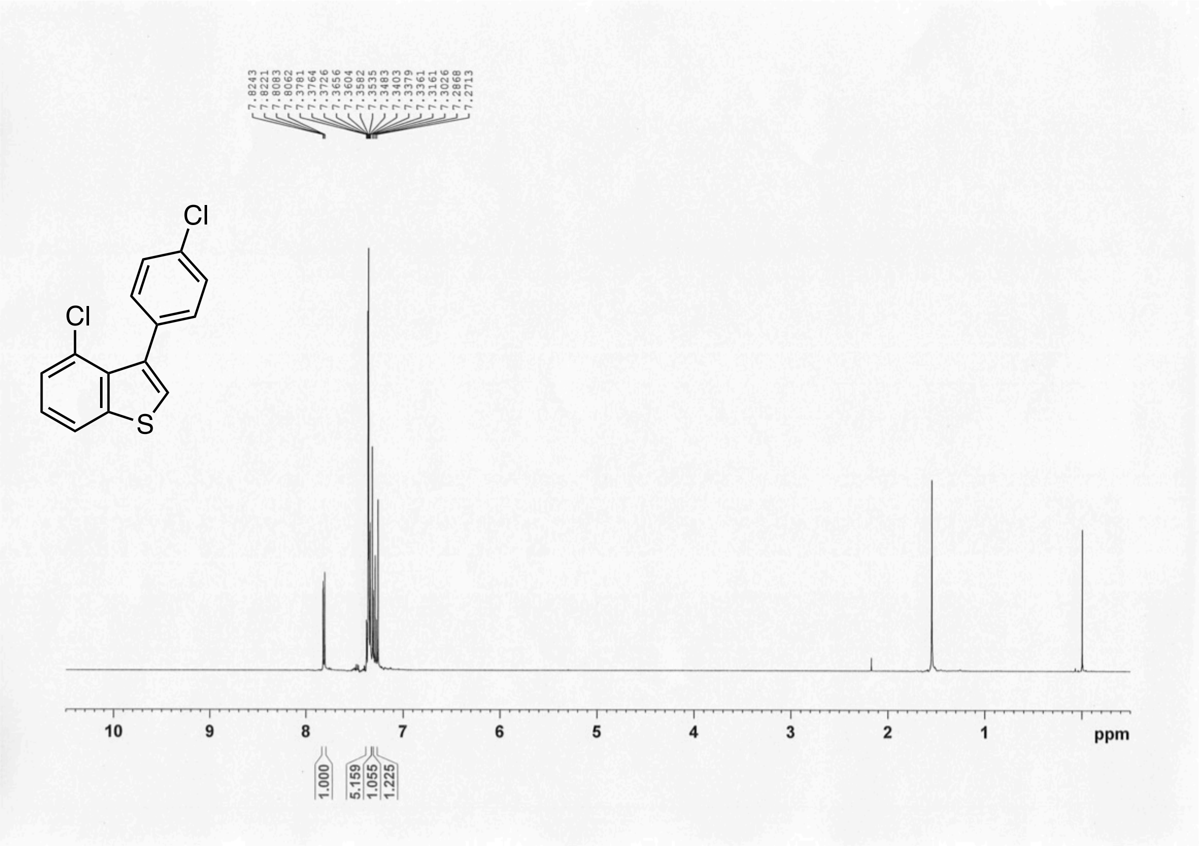

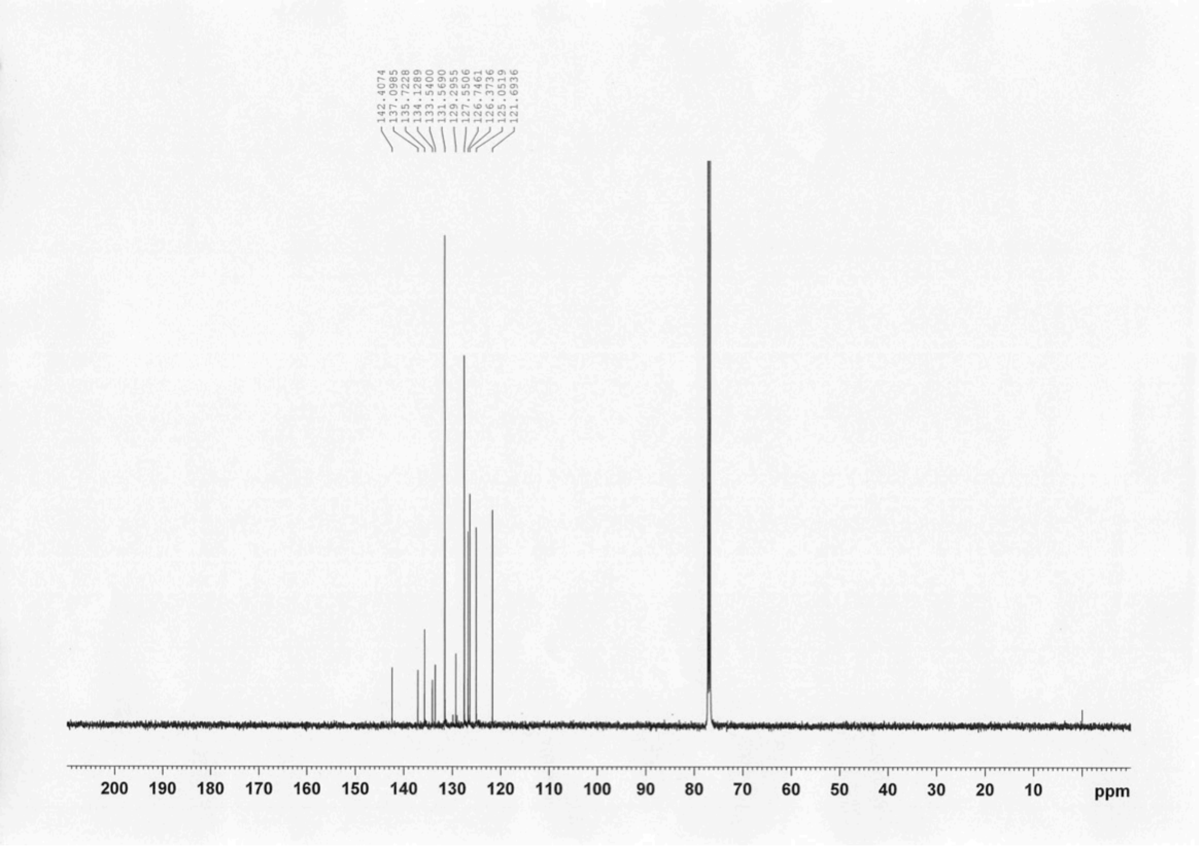

^{1}H NMR (500 MHz) and ^{13}C NMR (126 MHz) spectra of 4-chloro-3-(4-methoxycarbonylphenyl)benzo[*b*]thiophene (**3d**) ($CDCl_3$)

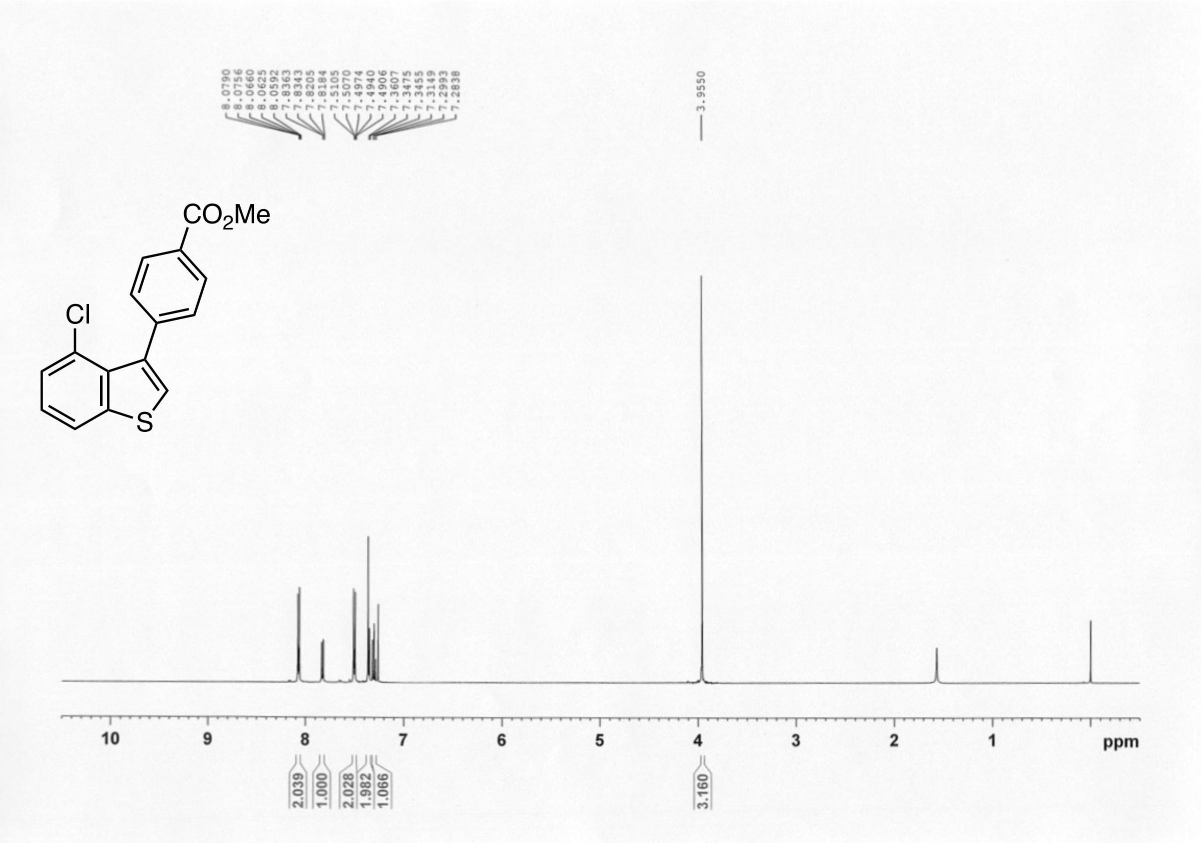

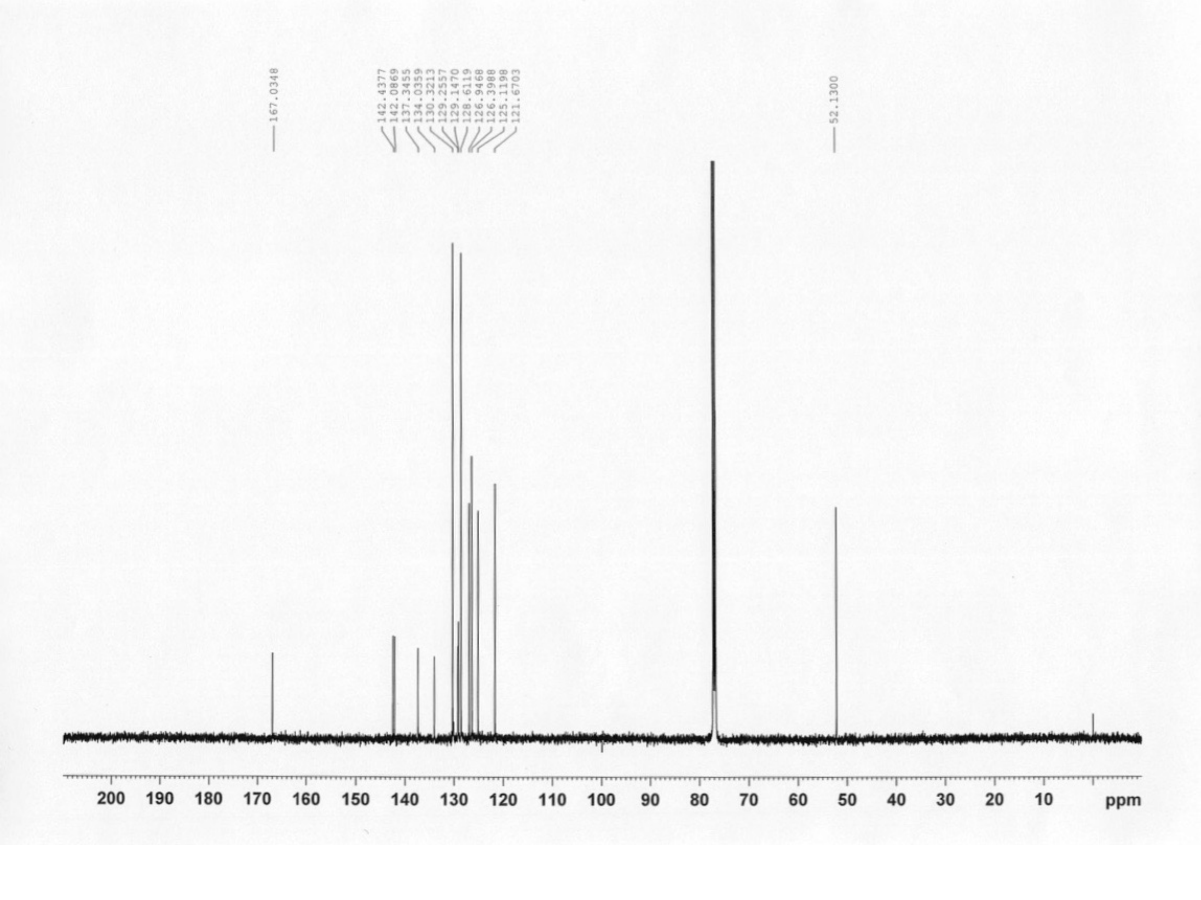

^{1}H NMR (500 MHz) and ^{13}C NMR (126 MHz) spectra of 3-(2-bromophenyl)-4-chlorobenzo[*b*]thiophene (**3e**) ($CDCl_3$)

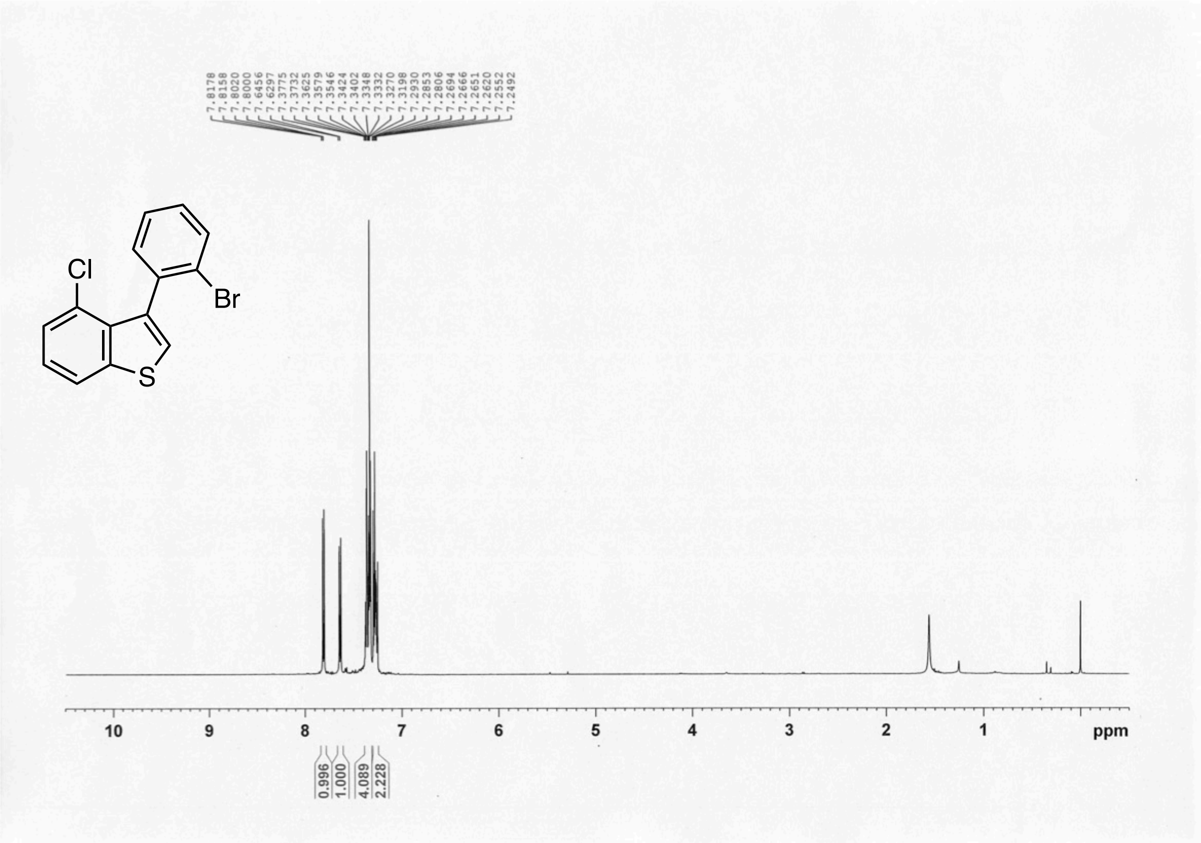

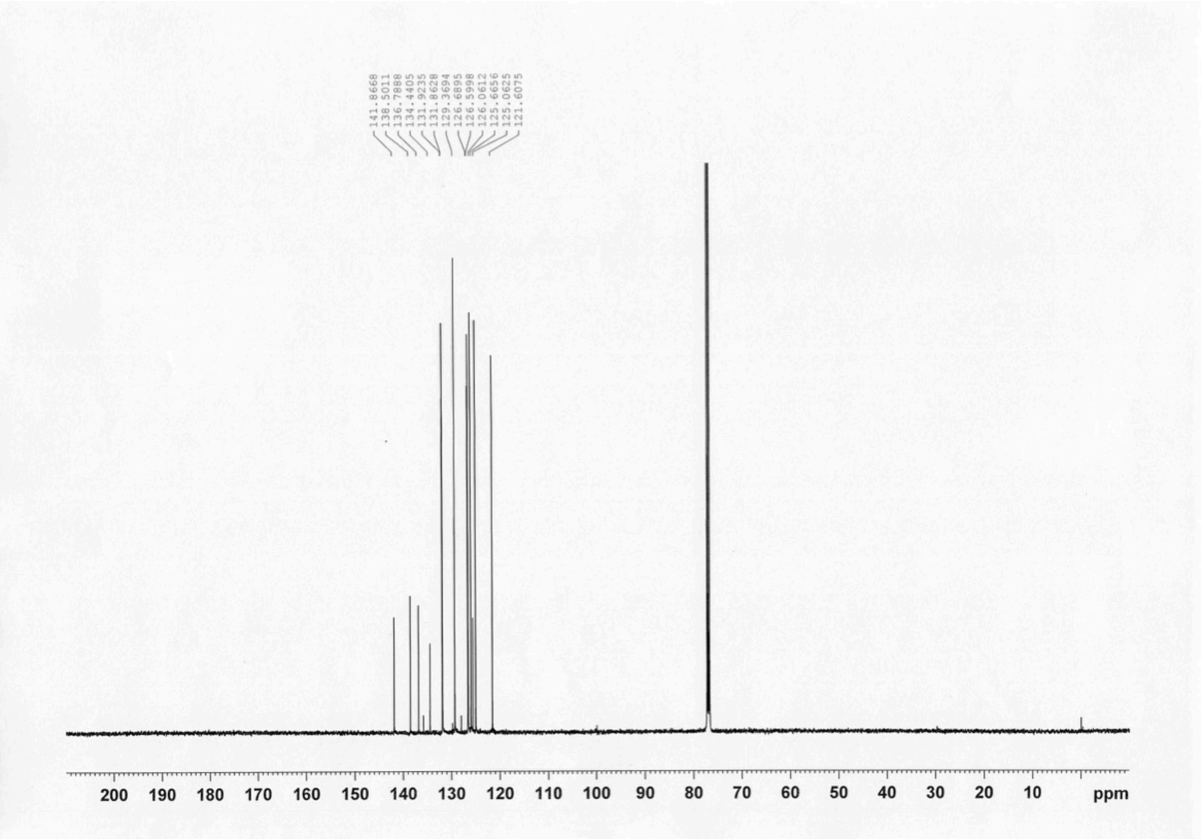

^{1}H NMR (500 MHz) and ^{13}C NMR (126 MHz) spectra of 4-chloro-3-(2-naphthyl)benzo[*b*]thiophene (**3f**) ($CDCl_3$)

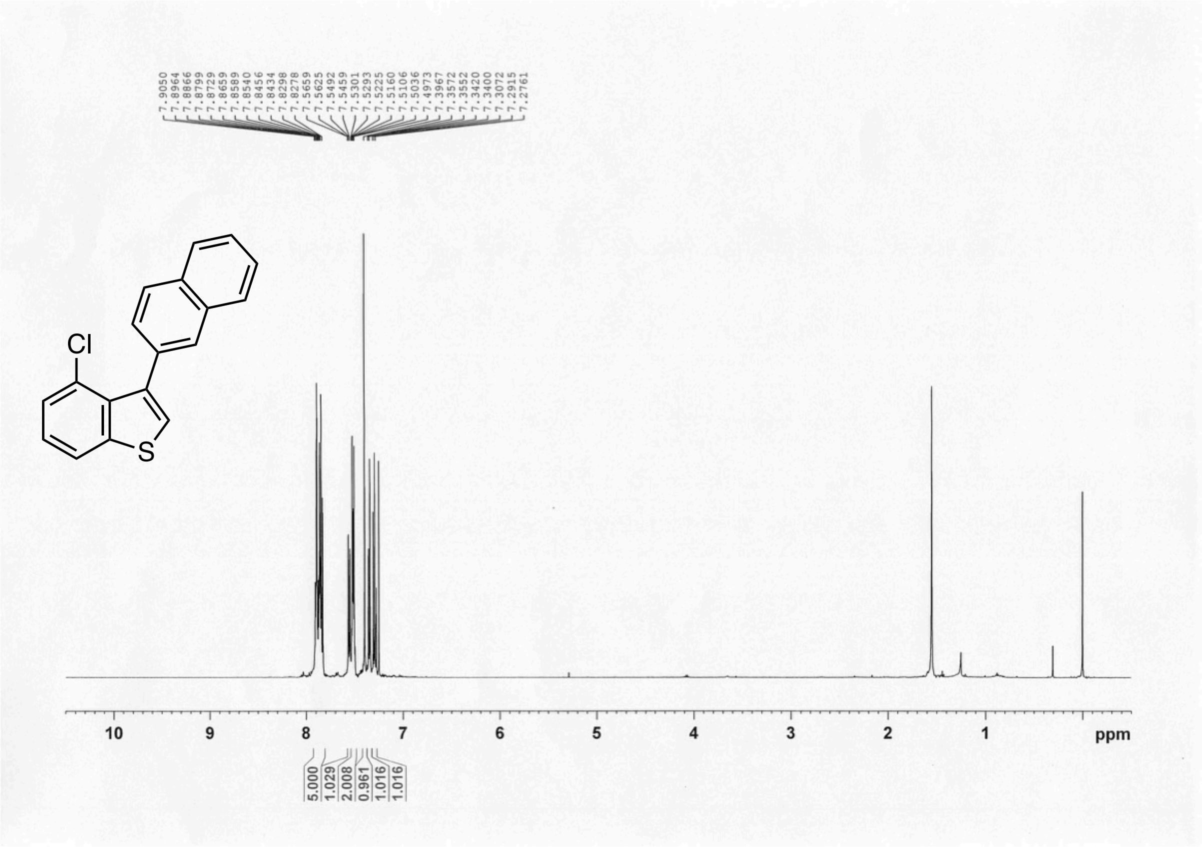

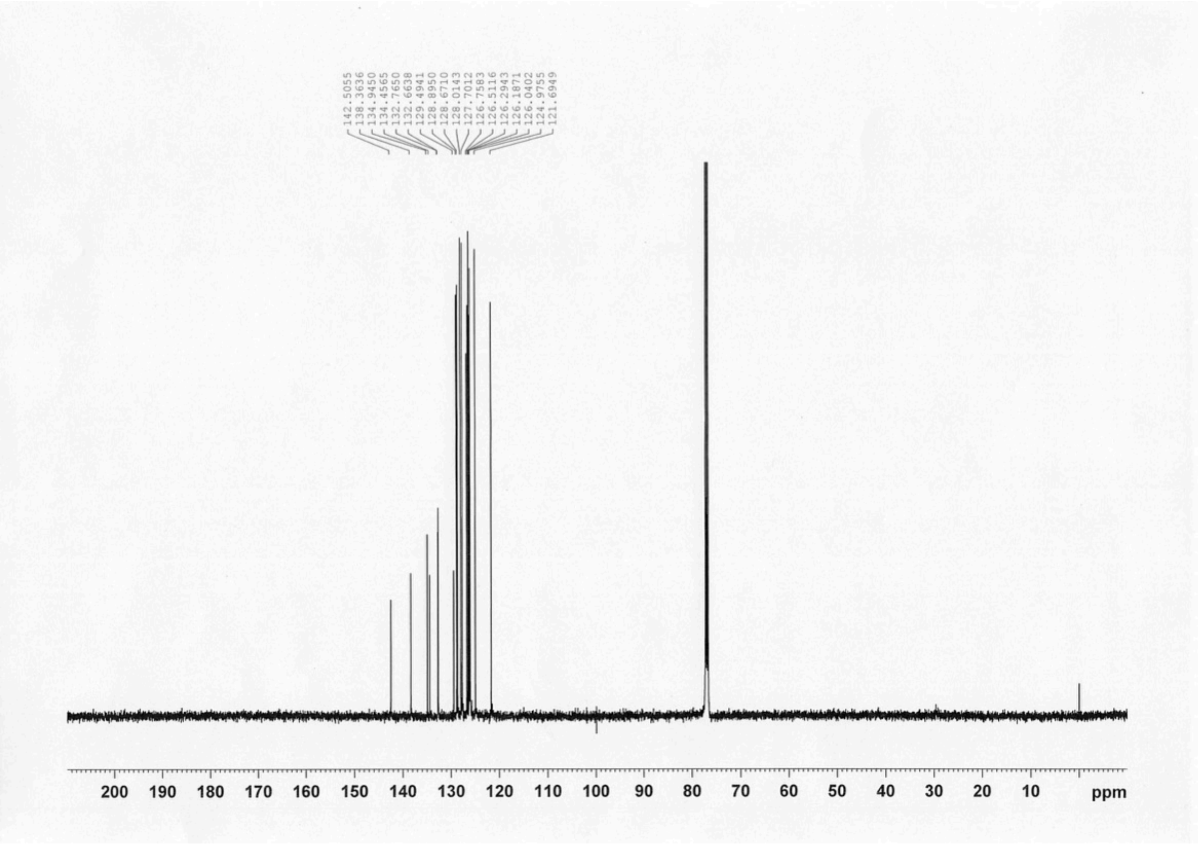

^{1}H NMR (500 MHz) and ^{13}C NMR (126 MHz) spectra of 4-chloro-3-(9-phenanthrenyl)benzo[*b*]thiophene (**3g**) ($CDCl_3$)

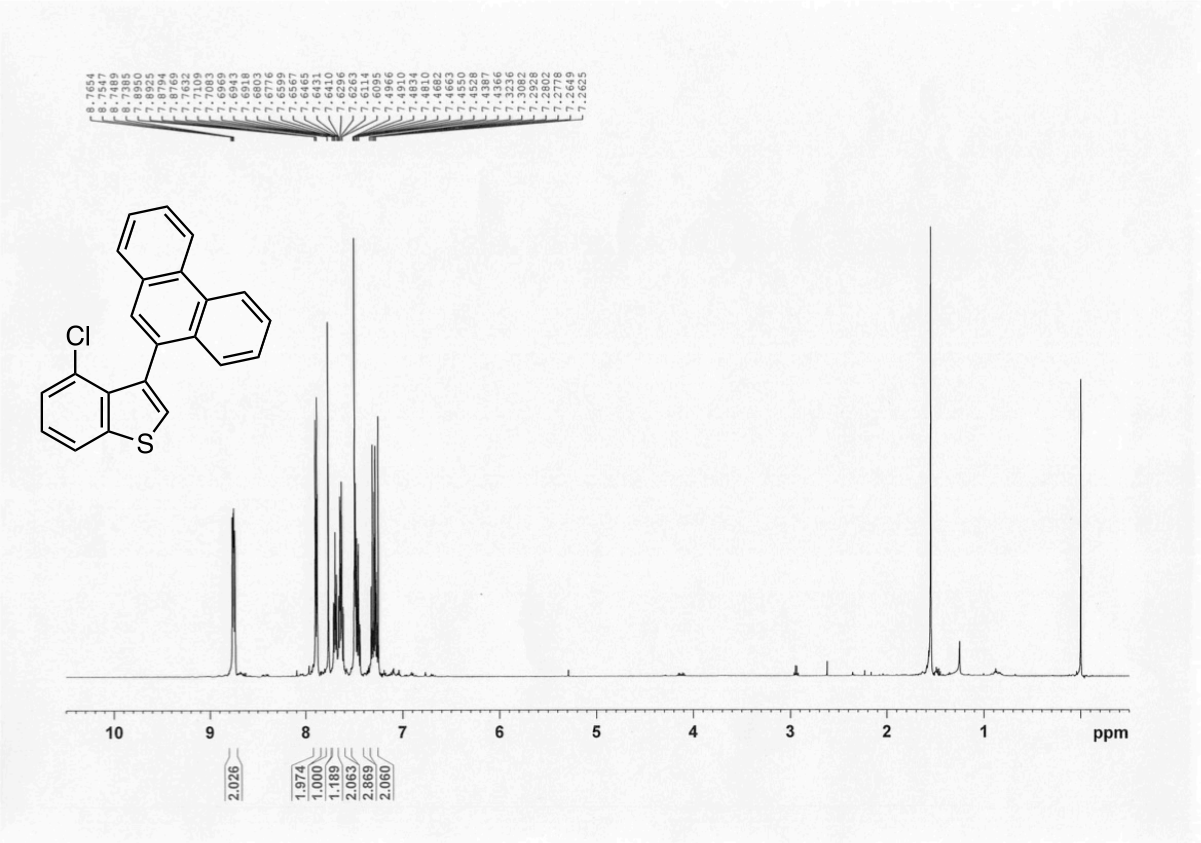

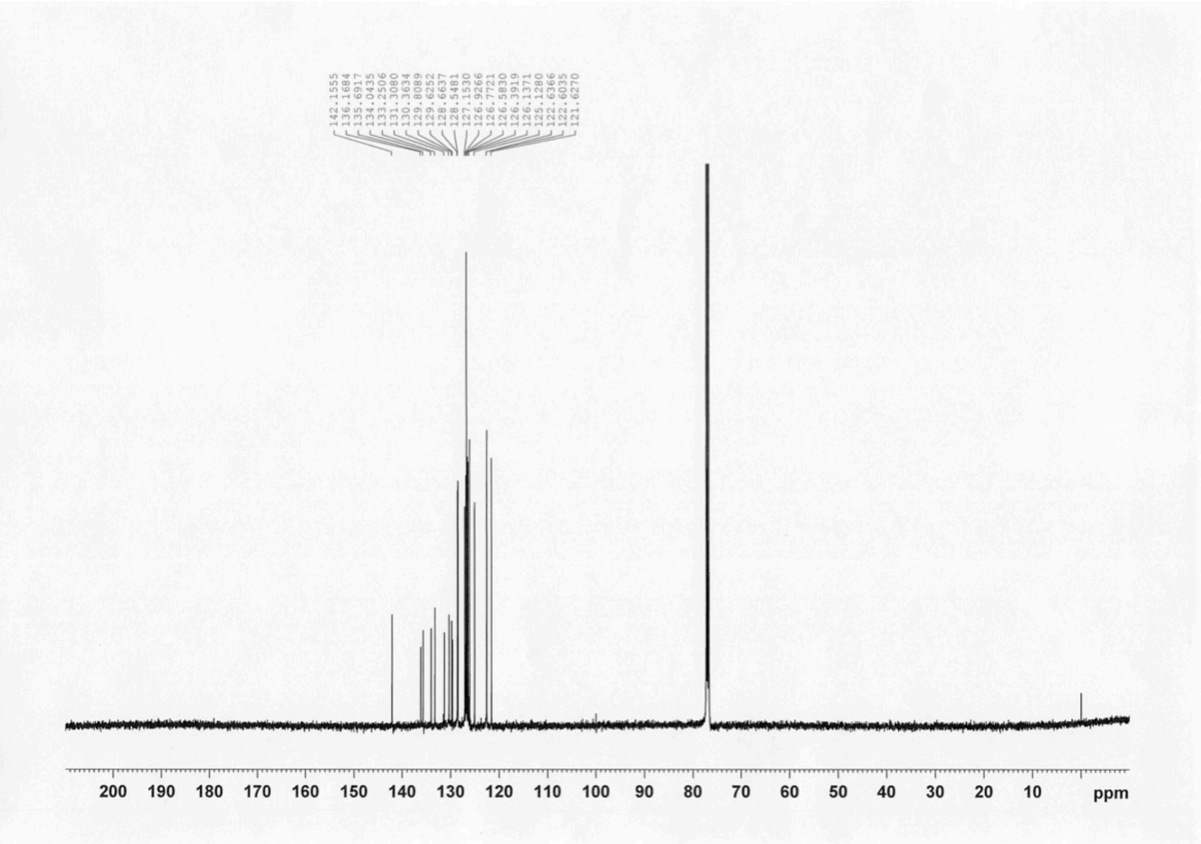

^{1}H NMR (500 MHz) and ^{13}C NMR (126 MHz) spectra of 4-chloro-3-(3-thienyl)benzo[*b*]thiophene (**3h**) ($CDCl_3$)

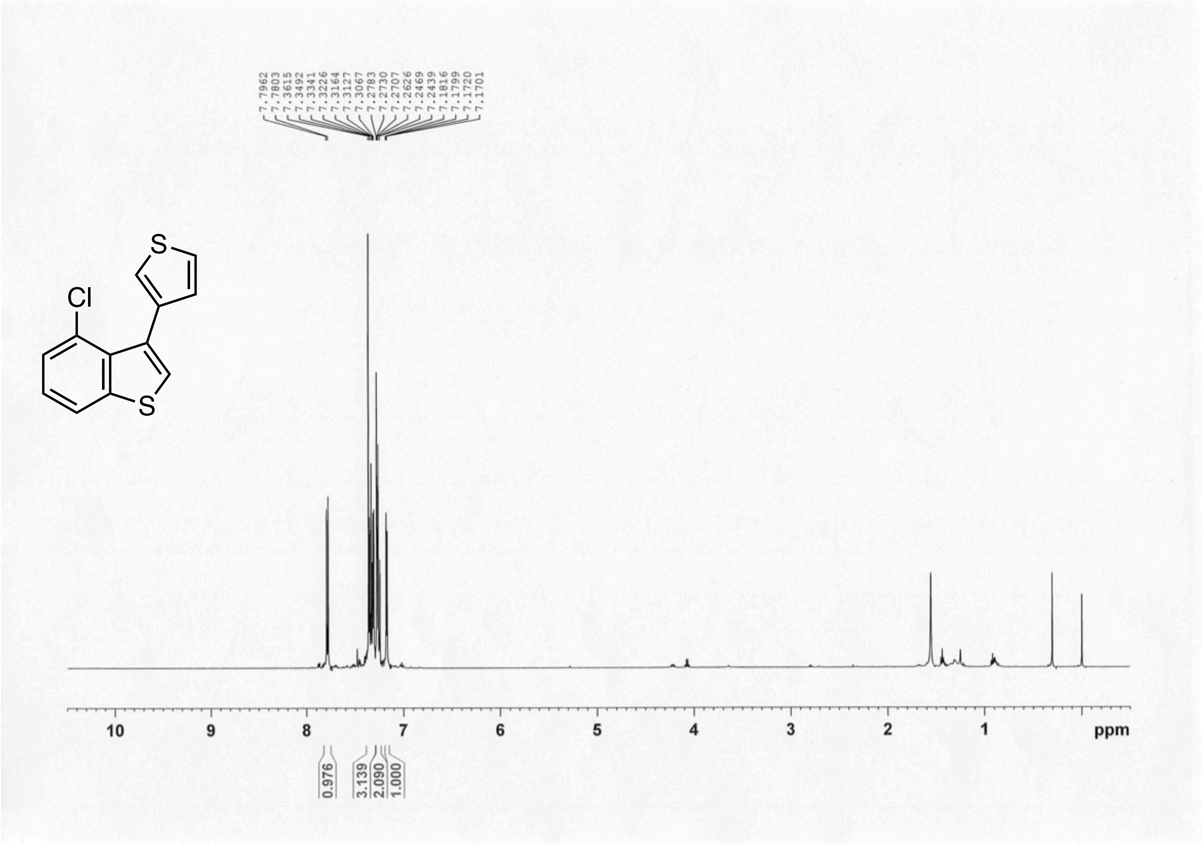

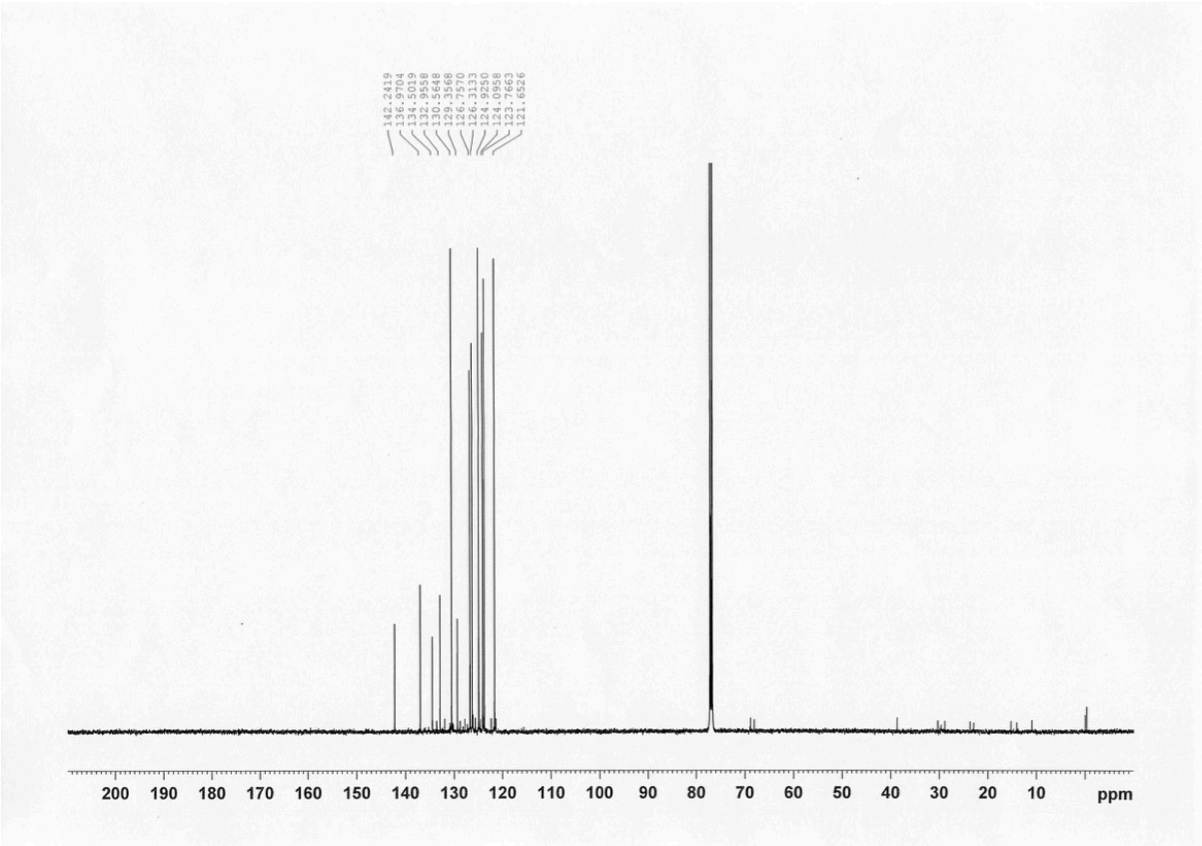

^{1}H NMR (500 MHz) and ^{13}C NMR (126 MHz) spectra of 4-chloro-3-(2-phenylethyl)benzo[*b*]thiophene (**3i**) ($CDCl_3$)

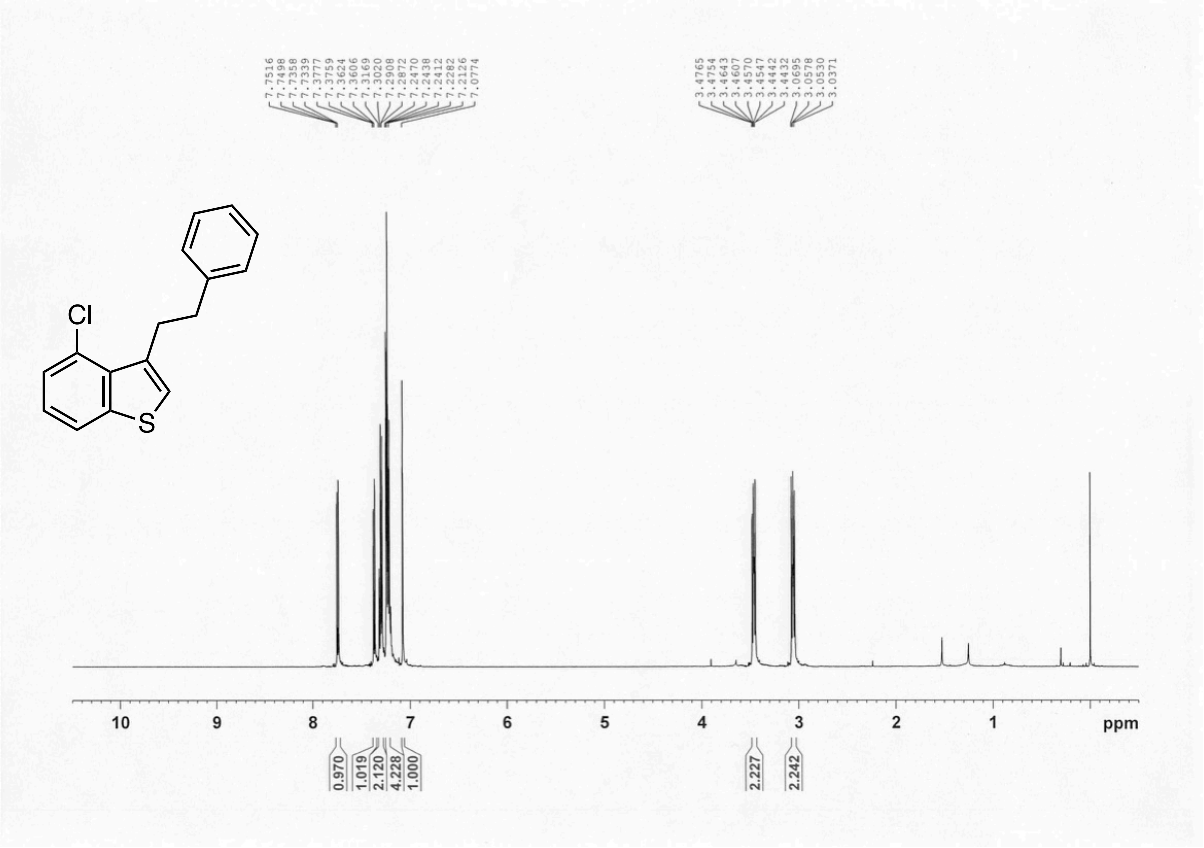

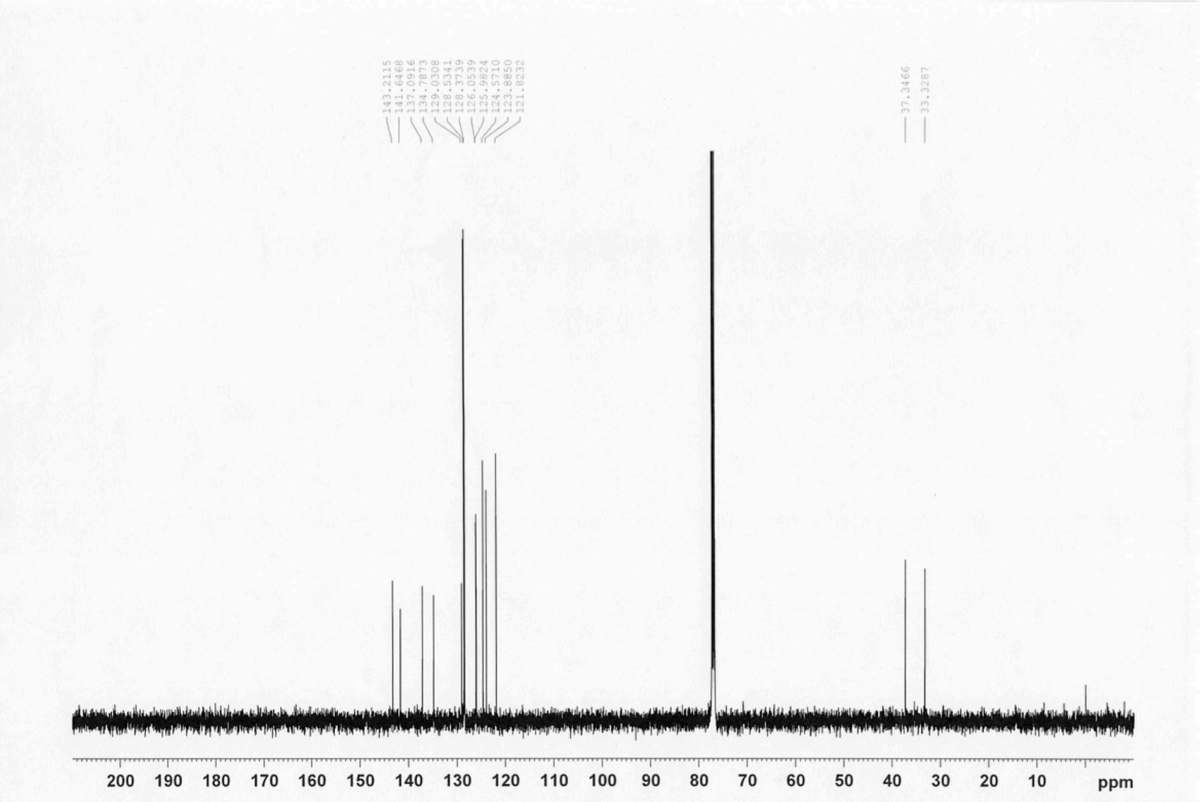

^{1}H NMR (500 MHz) and ^{13}C NMR (126 MHz) spectra of 4-chloro-3-cyclohexylbenzo[*b*]thiophene (**3j**) ($CDCl_3$)

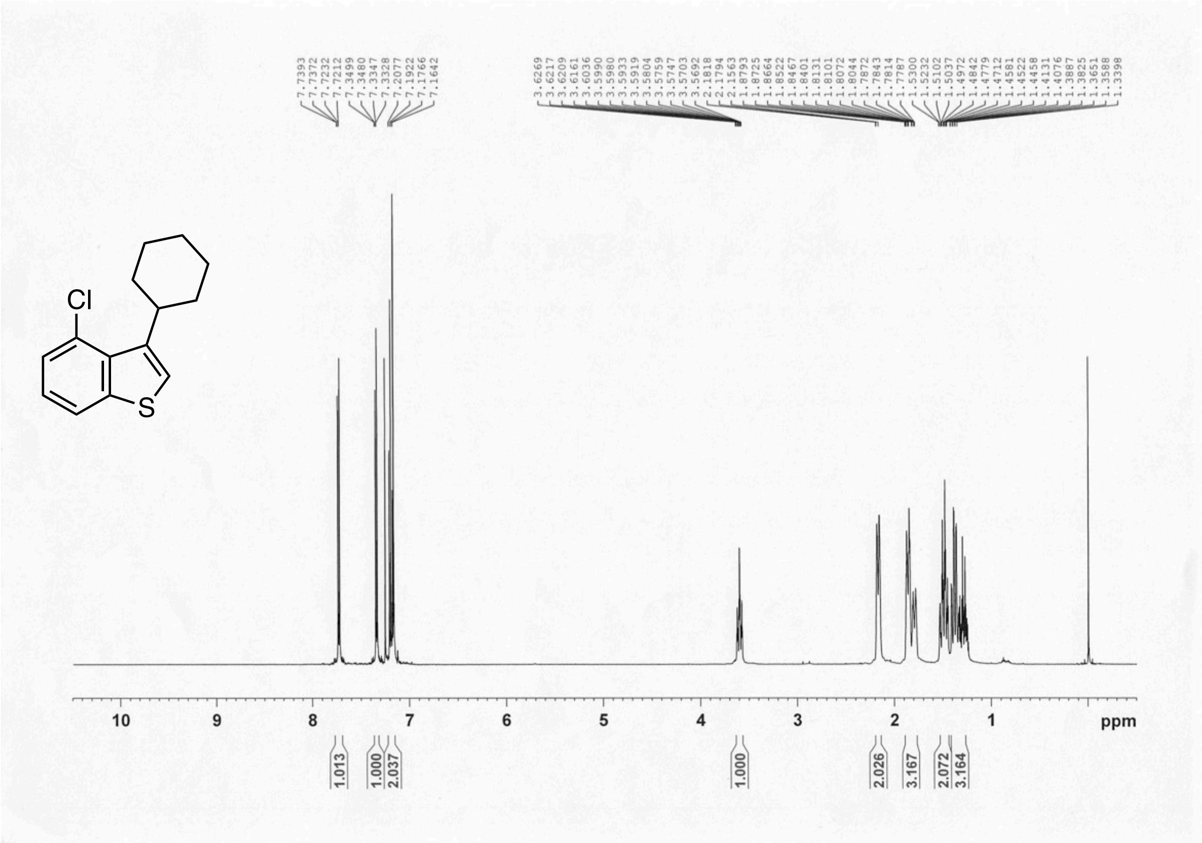

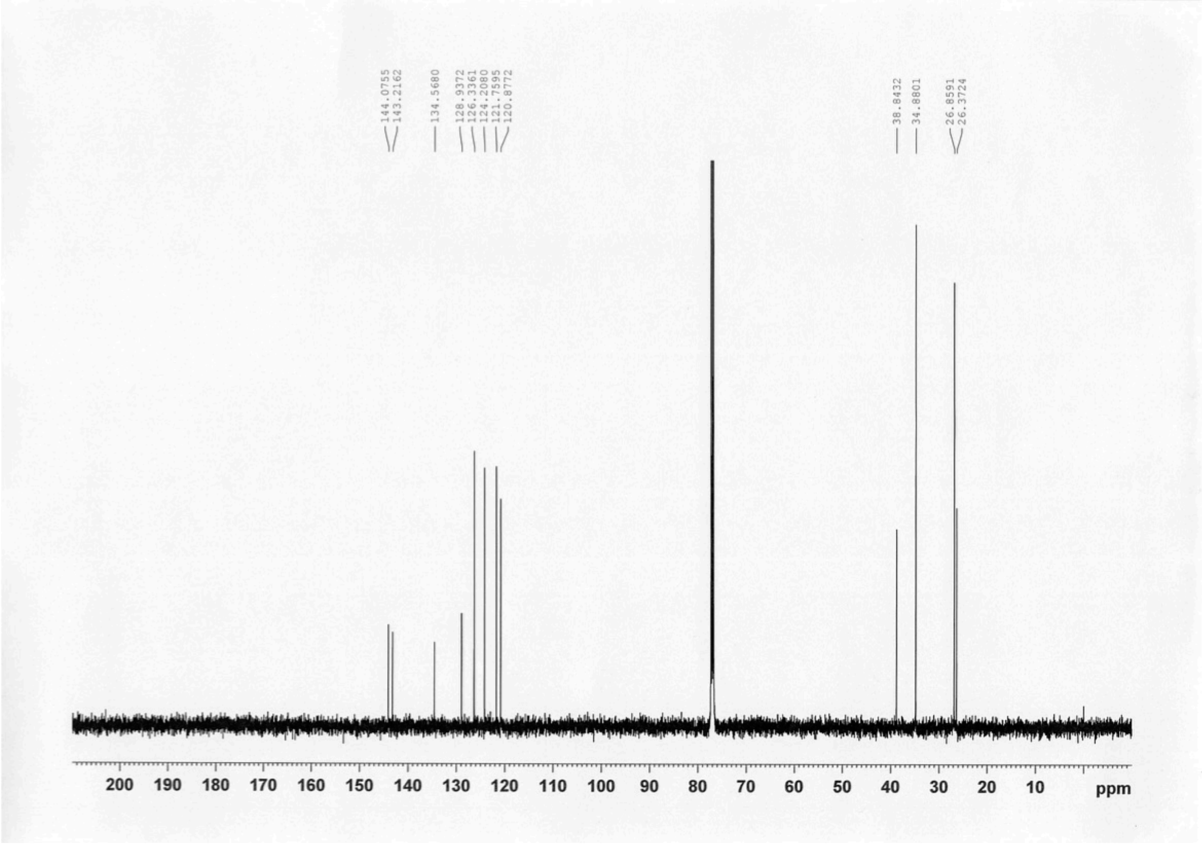

^{1}H NMR (500 MHz) and ^{13}C NMR (126 MHz) spectra of 4-chloro-3-(3,17-di-*O*-methylestradiol-17-yl)benzo[*b*]thiophene (**3k**) ($CDCl_3$)

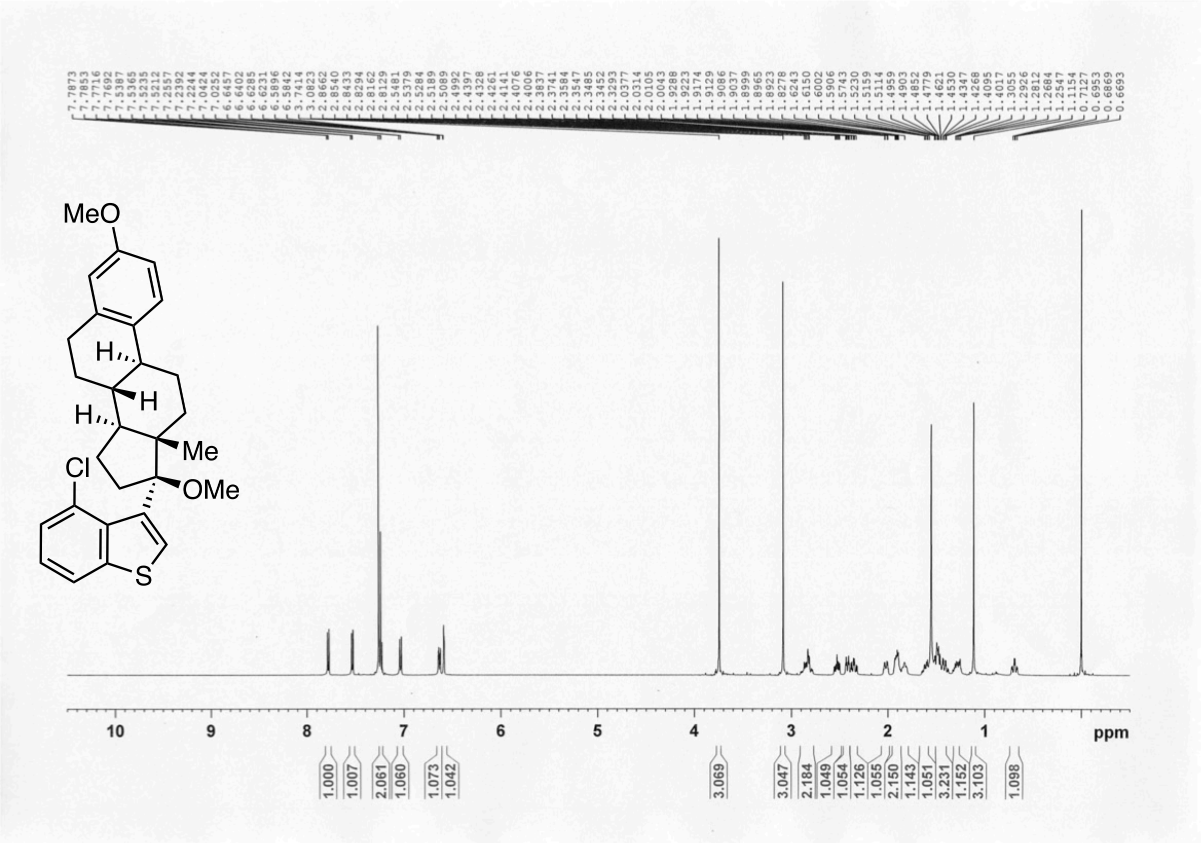

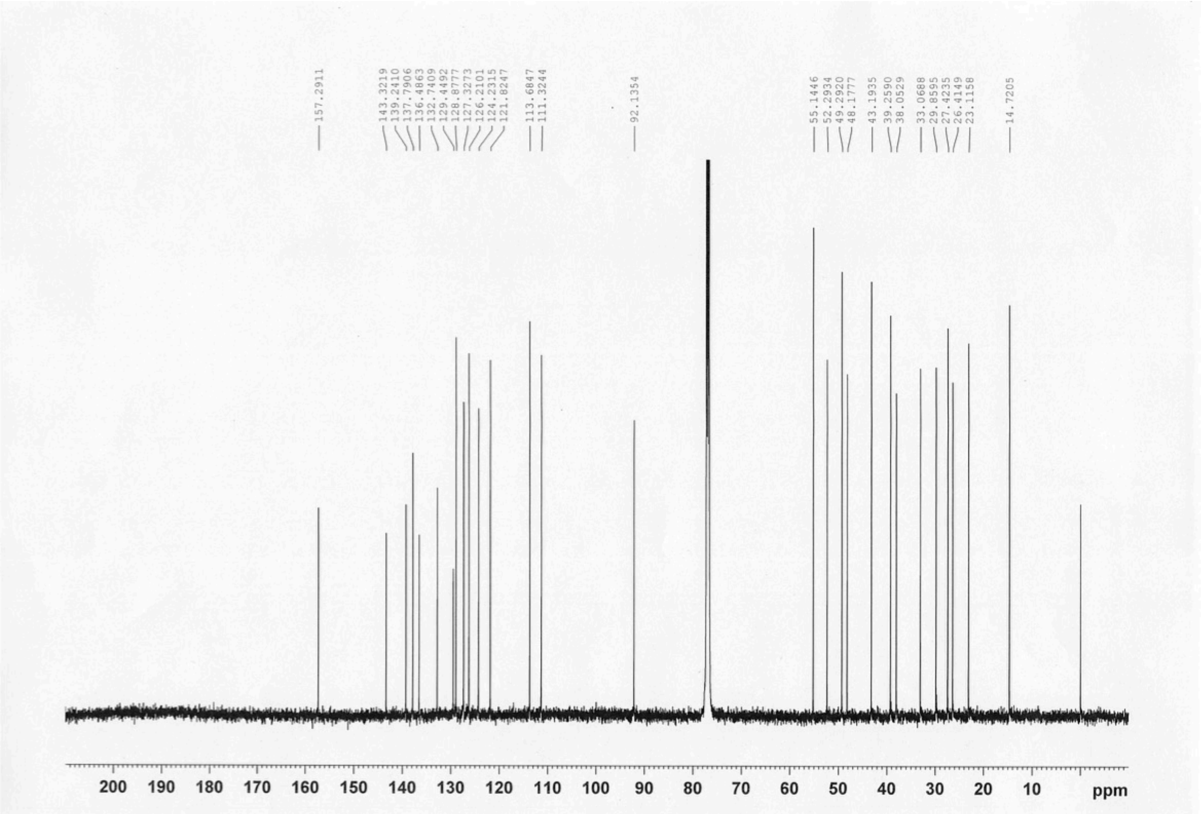

^{1}H NMR (500 MHz) and ^{13}C NMR (126 MHz) spectra of 4-fluoro-3-(4-tolyl)benzo[*b*]thiophene (**3m**) ($CDCl_3$)

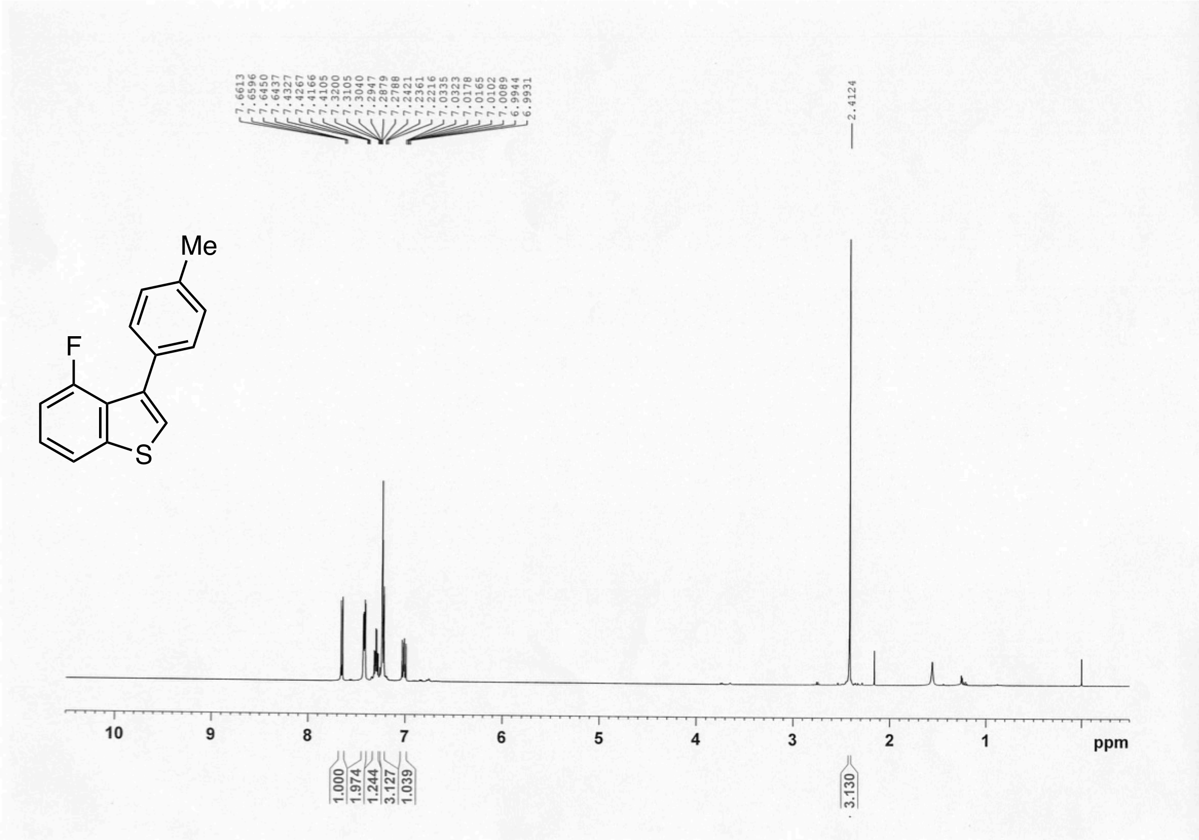

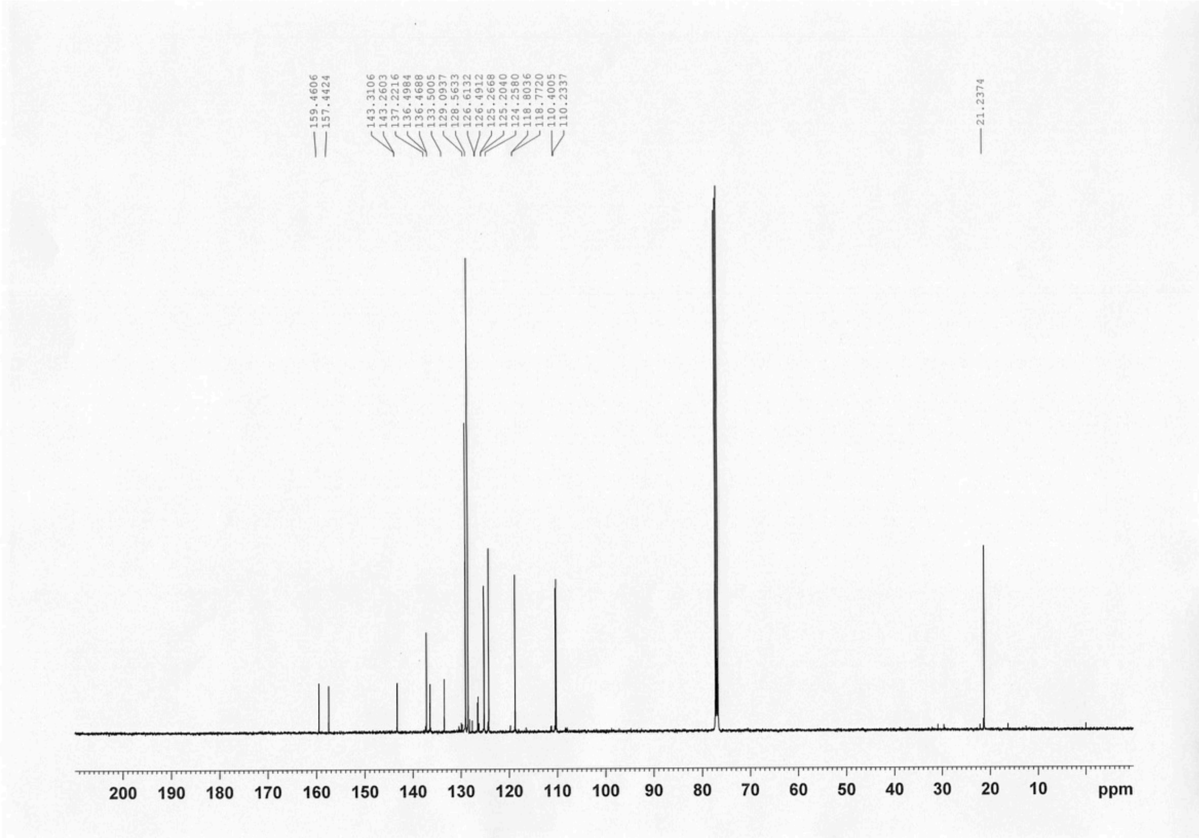

^{1}H NMR (500 MHz) and ^{13}C NMR (126 MHz) spectra of 4-bromo-3-(4-tolyl)benzo[*b*]thiophene (**3n**) ($CDCl_3$)

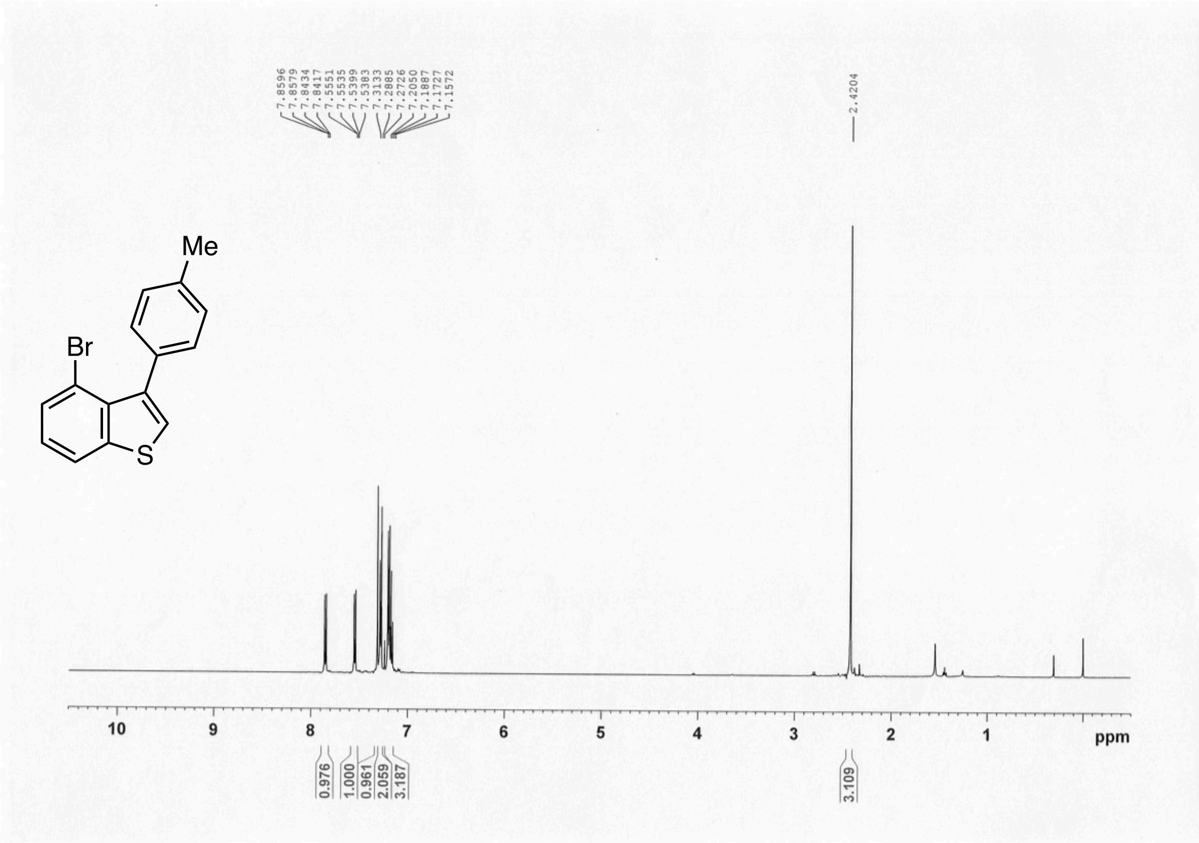

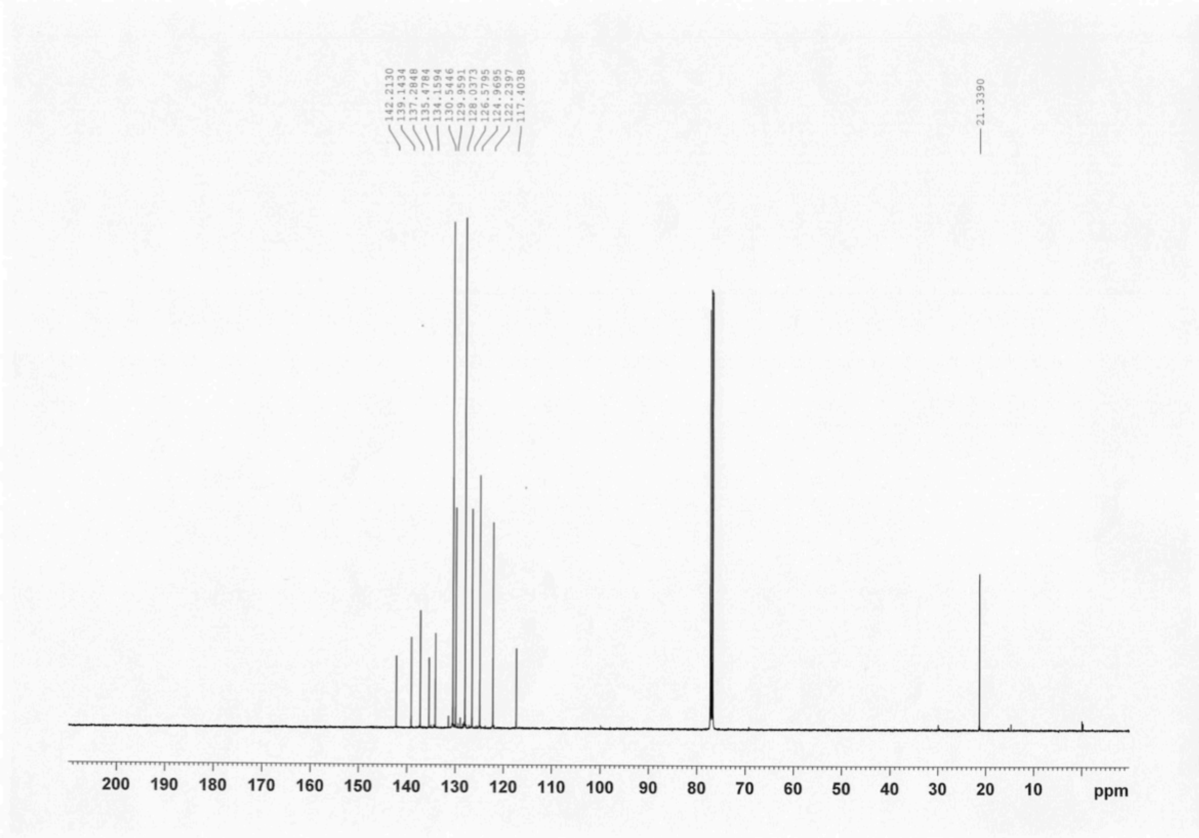

^{1}H NMR (500 MHz) and ^{13}C NMR (126 MHz) spectra of 4-methoxy-3-(4-tolyl)benzo[*b*]thiophene (**3o**) ($CDCl_3$)

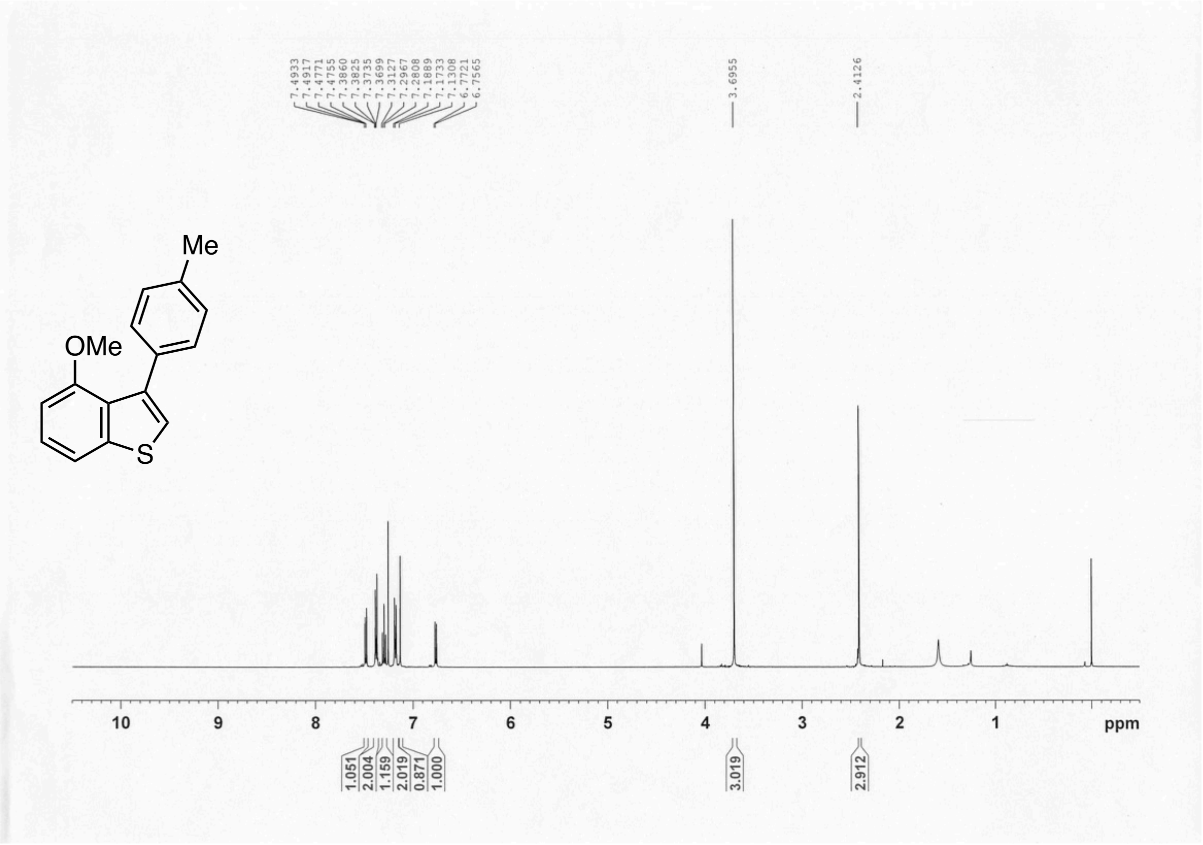

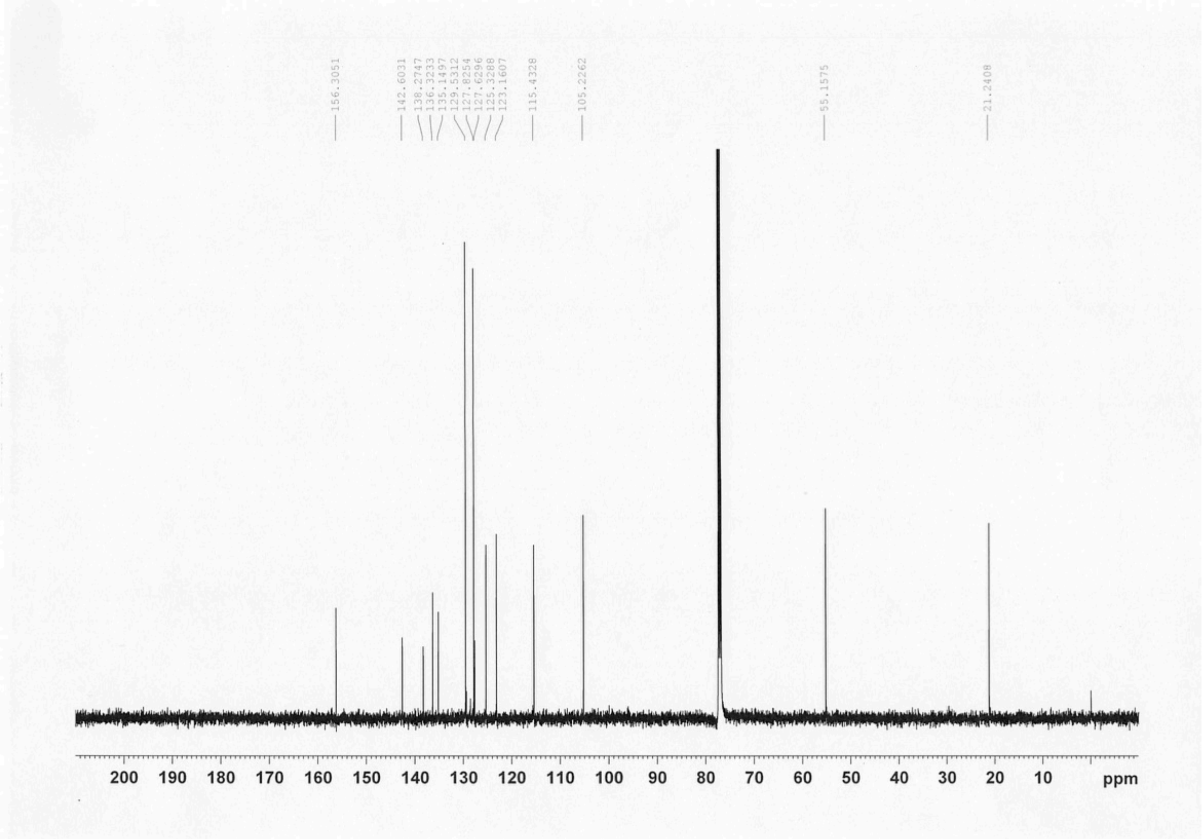

^{1}H NMR (500 MHz) and ^{13}C NMR (126 MHz) spectra of 4-diethylamino-3-(4-tolyl)benzo[*b*]thiophene (**3p**) ($CDCl_3$)

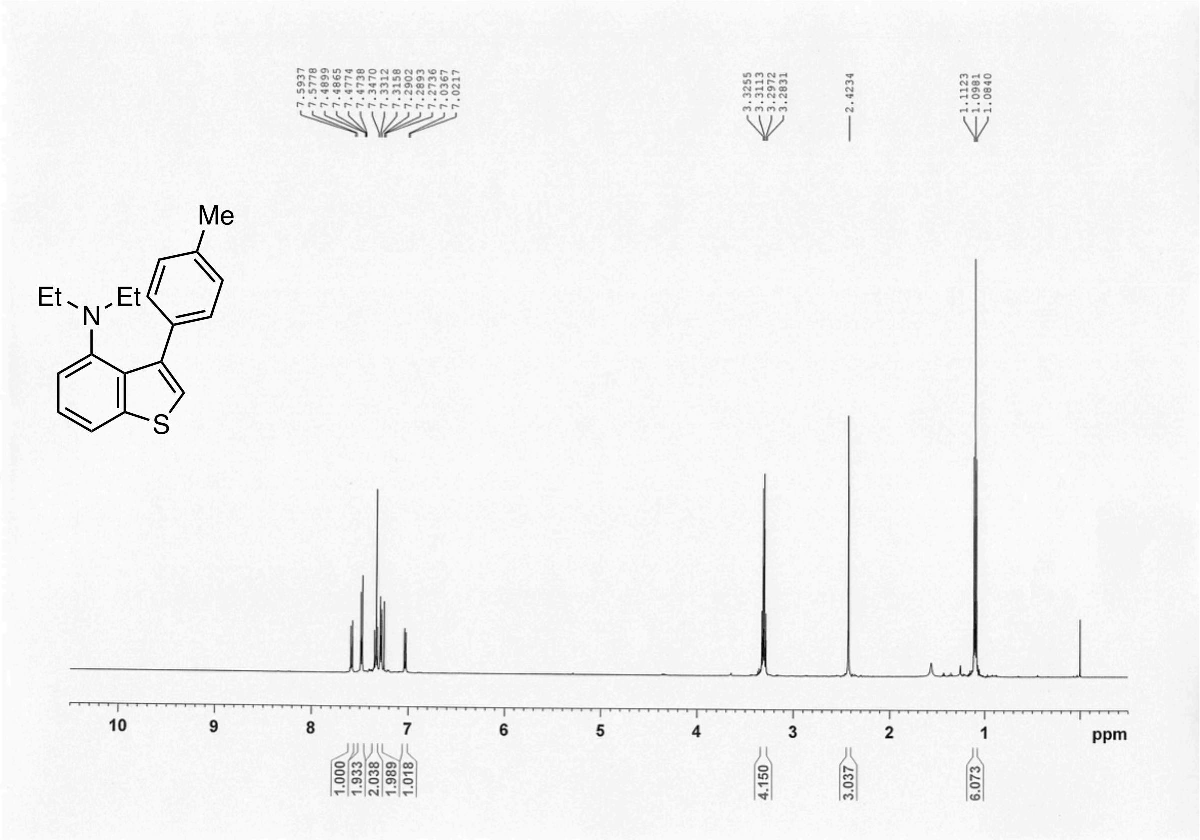

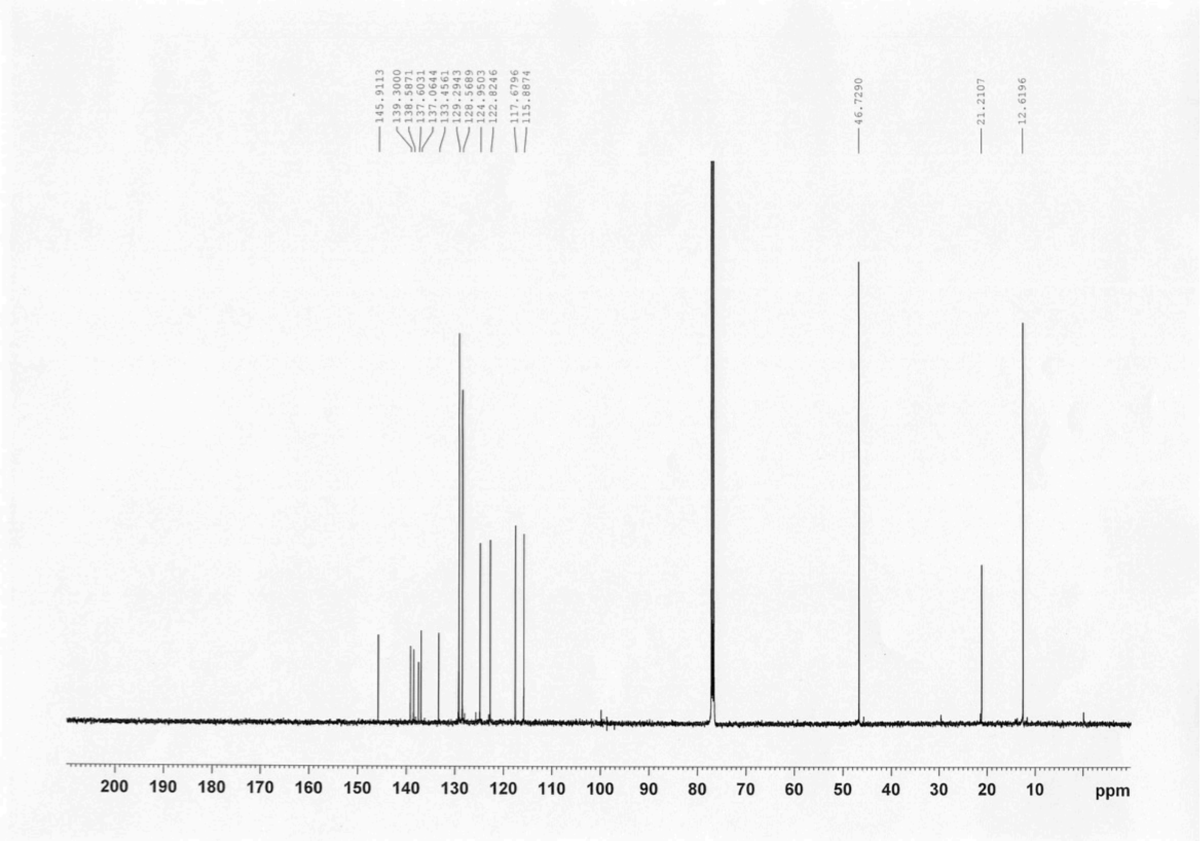

^{1}H NMR (500 MHz) and ^{13}C NMR (126 MHz) spectra of 5,6-(methylenedioxy)-3-(4-tolyl)benzo[*b*]thiophene (**3s**) ($CDCl_3$)

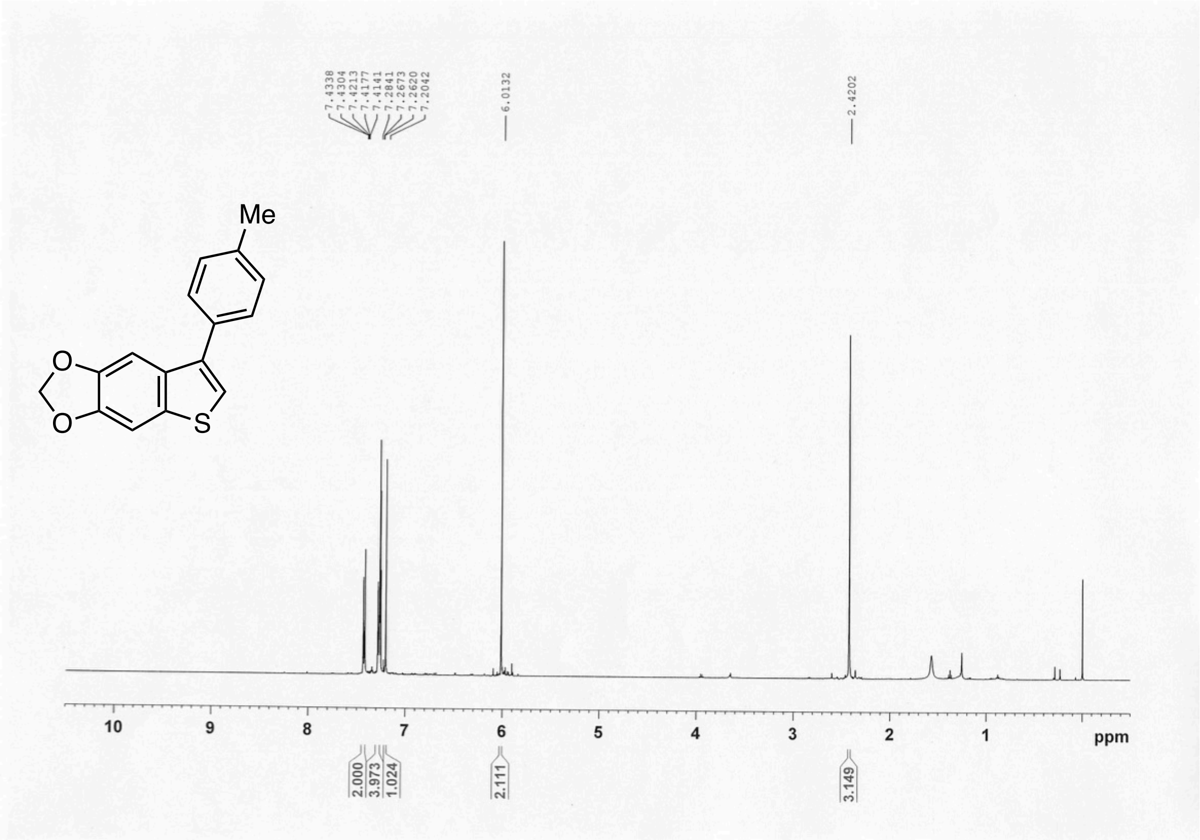

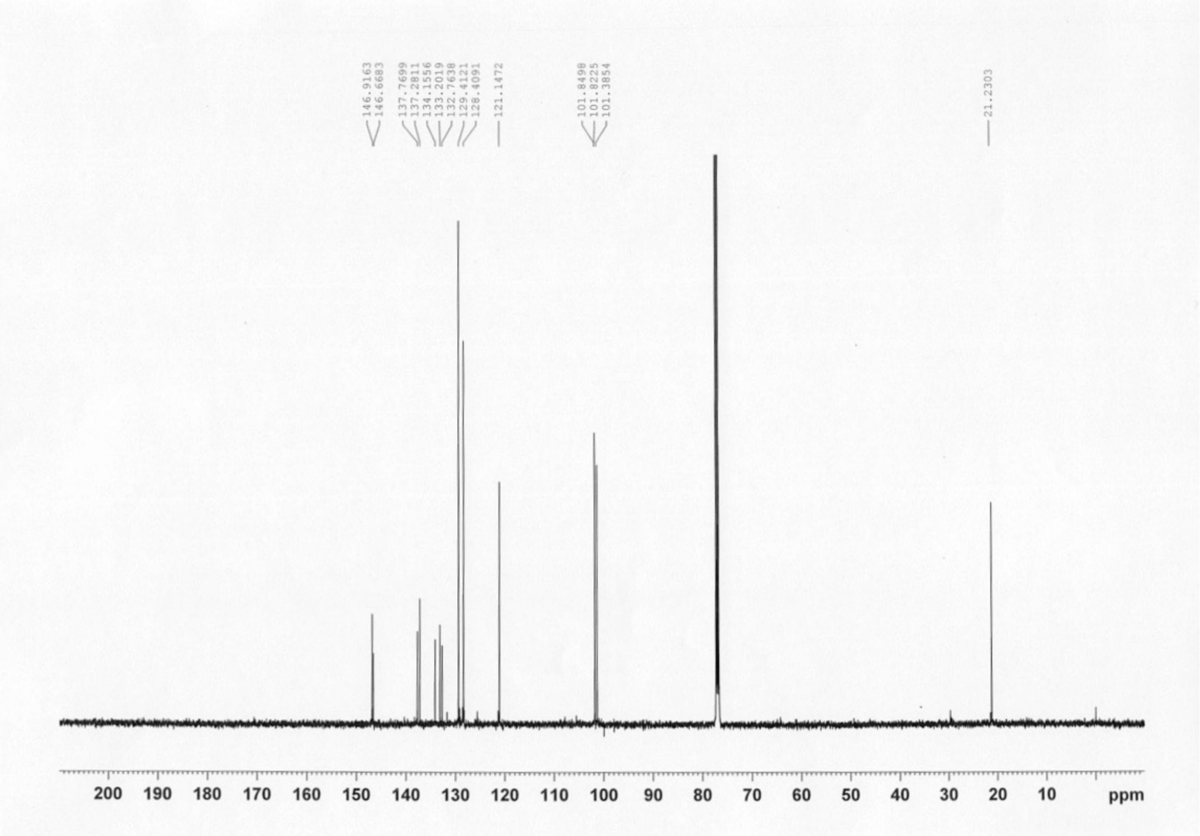

^{1}H NMR (500 MHz) and ^{13}C NMR (126 MHz) spectra of 6-bromo-4-chloro-3-(4-tolyl)benzo[*b*]thiophene (**3t**) ($CDCl_3$)

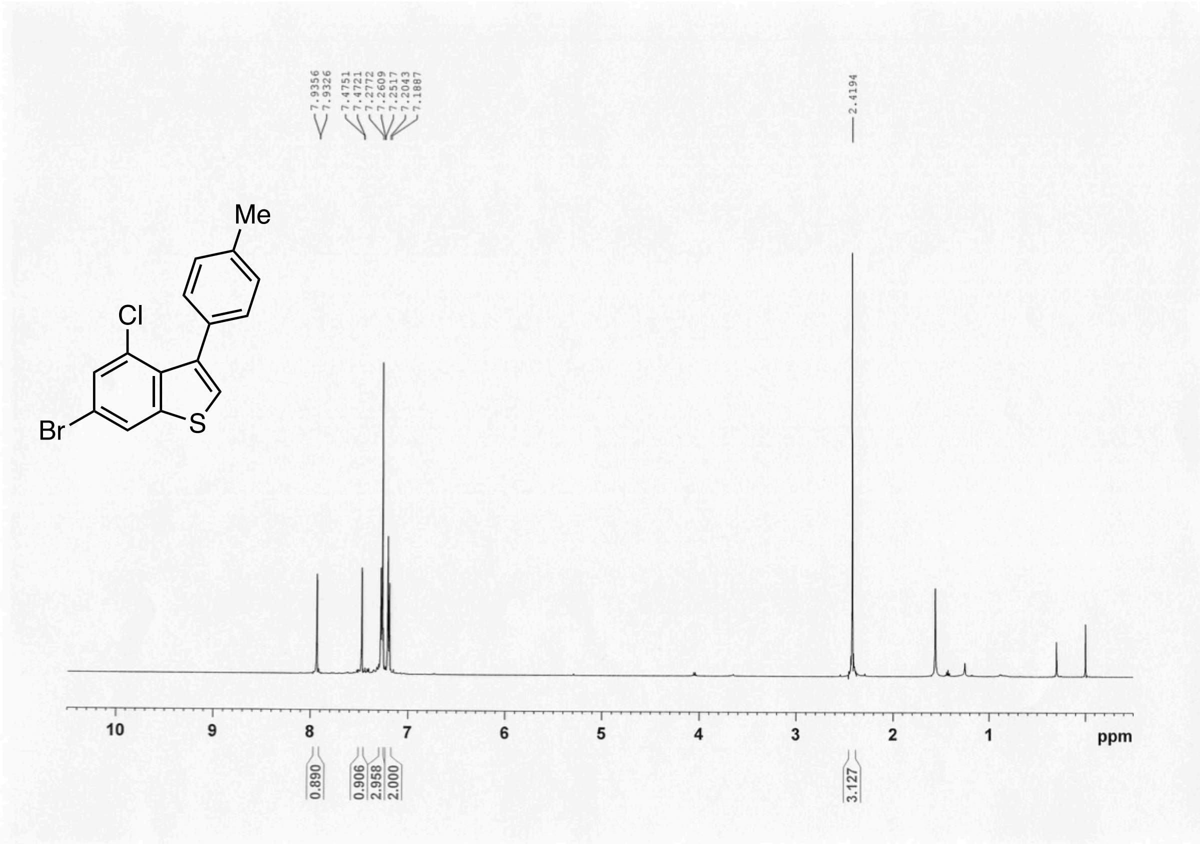

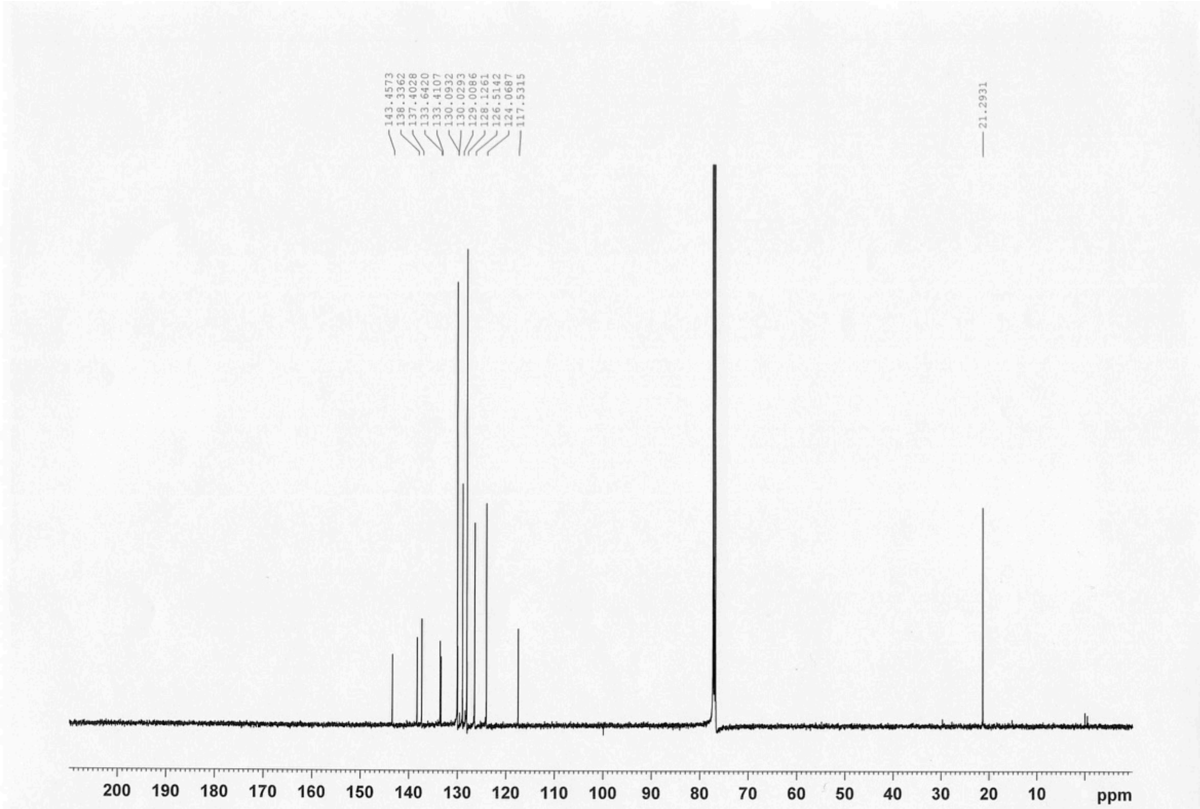

^{1}H NMR (500 MHz) and ^{13}C NMR (126 MHz) spectra of 2,3-dibutyl-8-(4-tolyl)benzo[1,2-*b*:3,4-*b'*]dithiophene (**3u**) ($CDCl_3$)

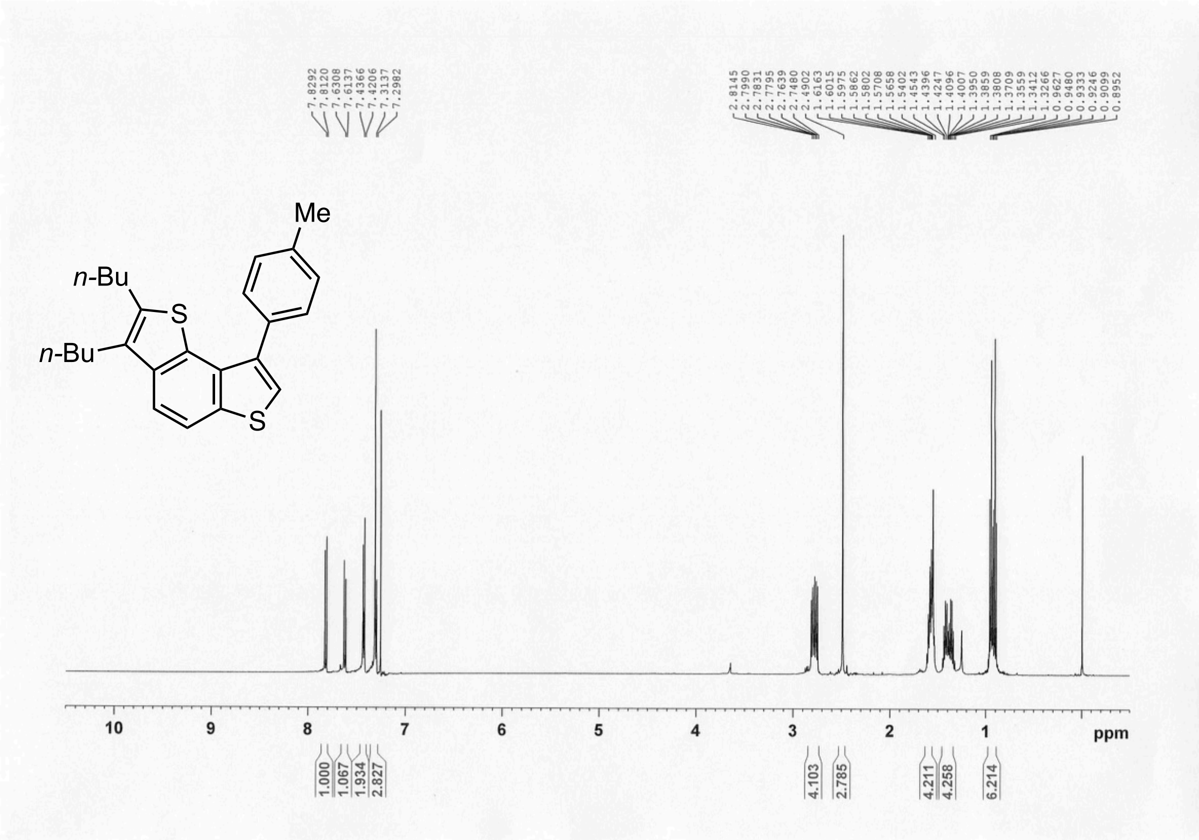

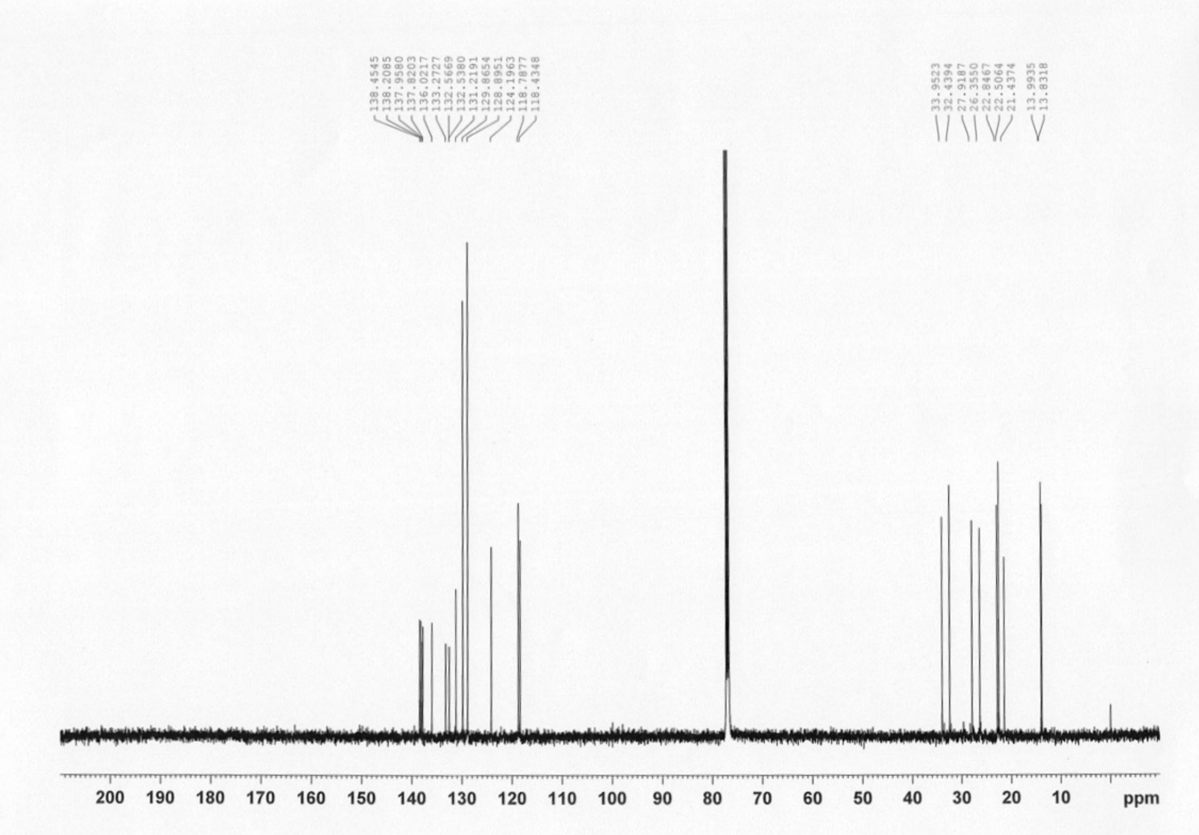

^{1}H NMR (500 MHz) and ^{13}C NMR (126 MHz) spectra of 1-(4-tolyl)phenanthro[3,4-*b*]thiophene (**3v**) ($CDCl_3$)

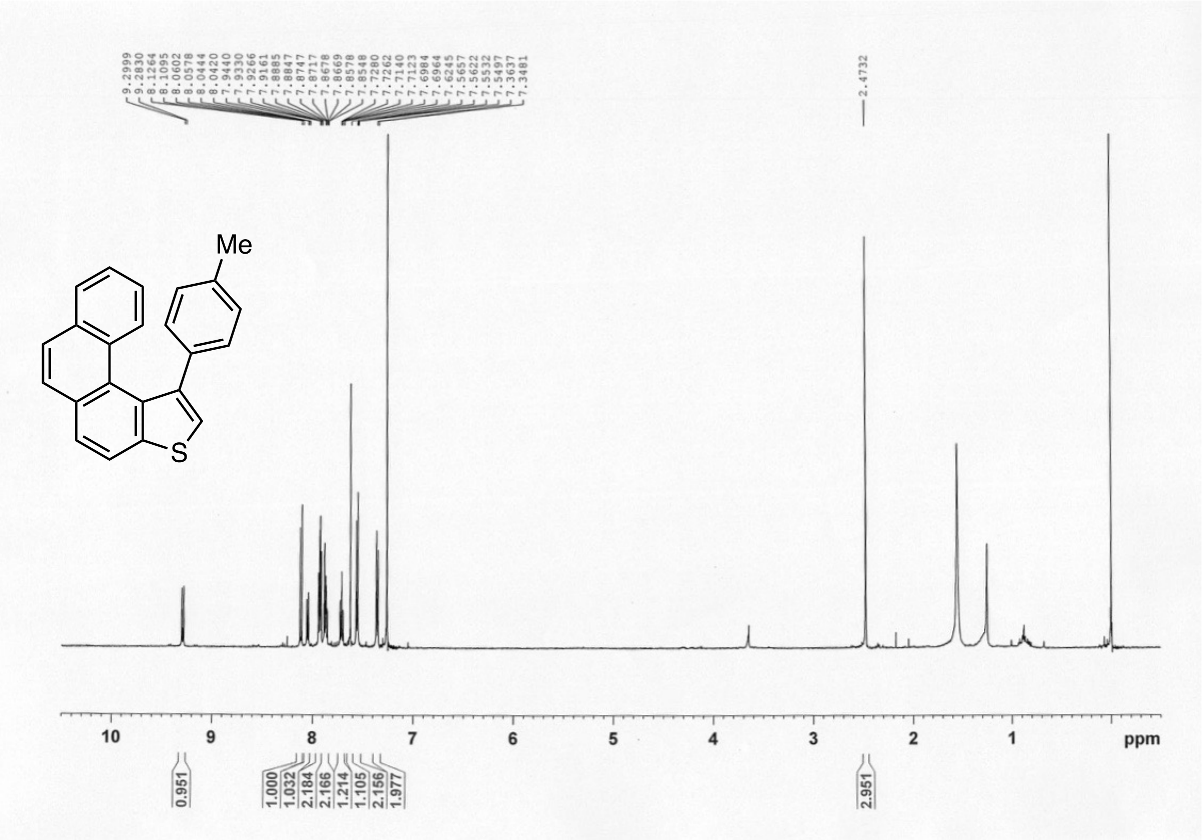

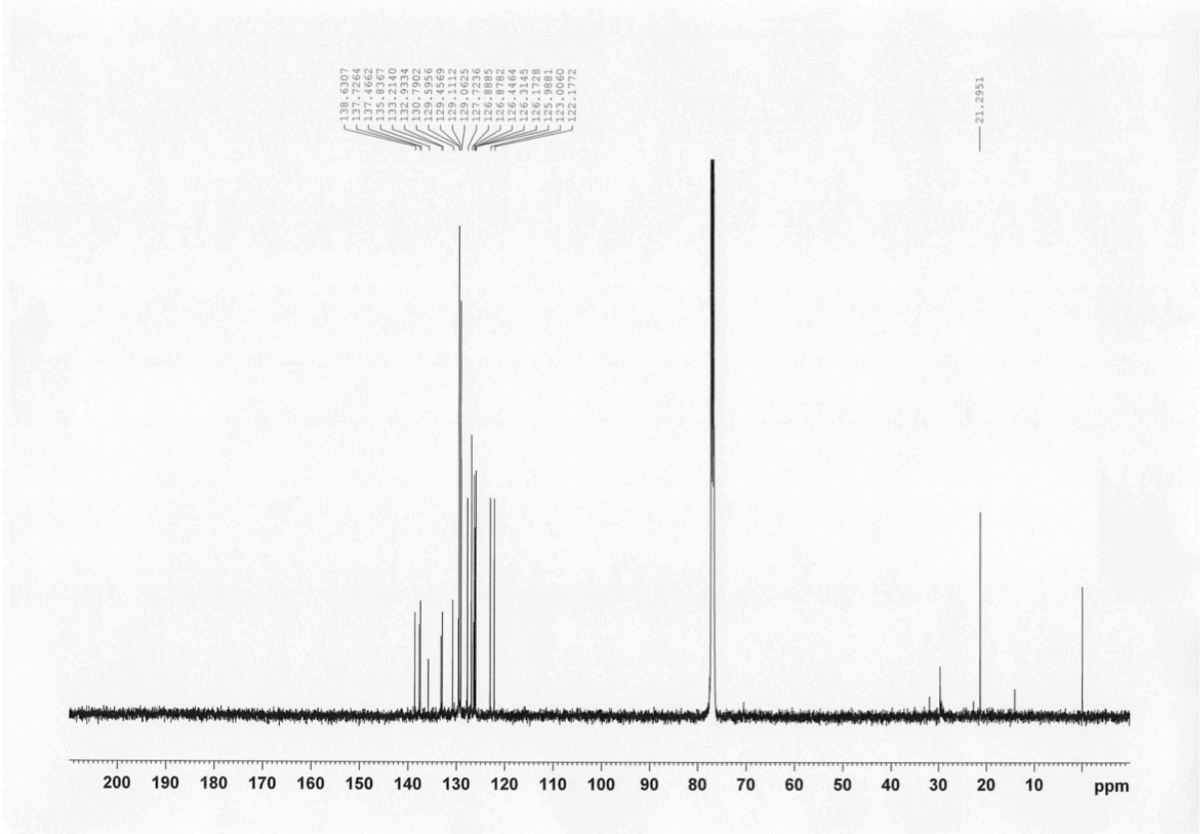

^{1}H NMR (500 MHz) and ^{13}C NMR (126 MHz) spectra of 4-chloro-3-(hydroxymethyl)benzo[*b*]thiophene (**3w**) ($CDCl_3$)

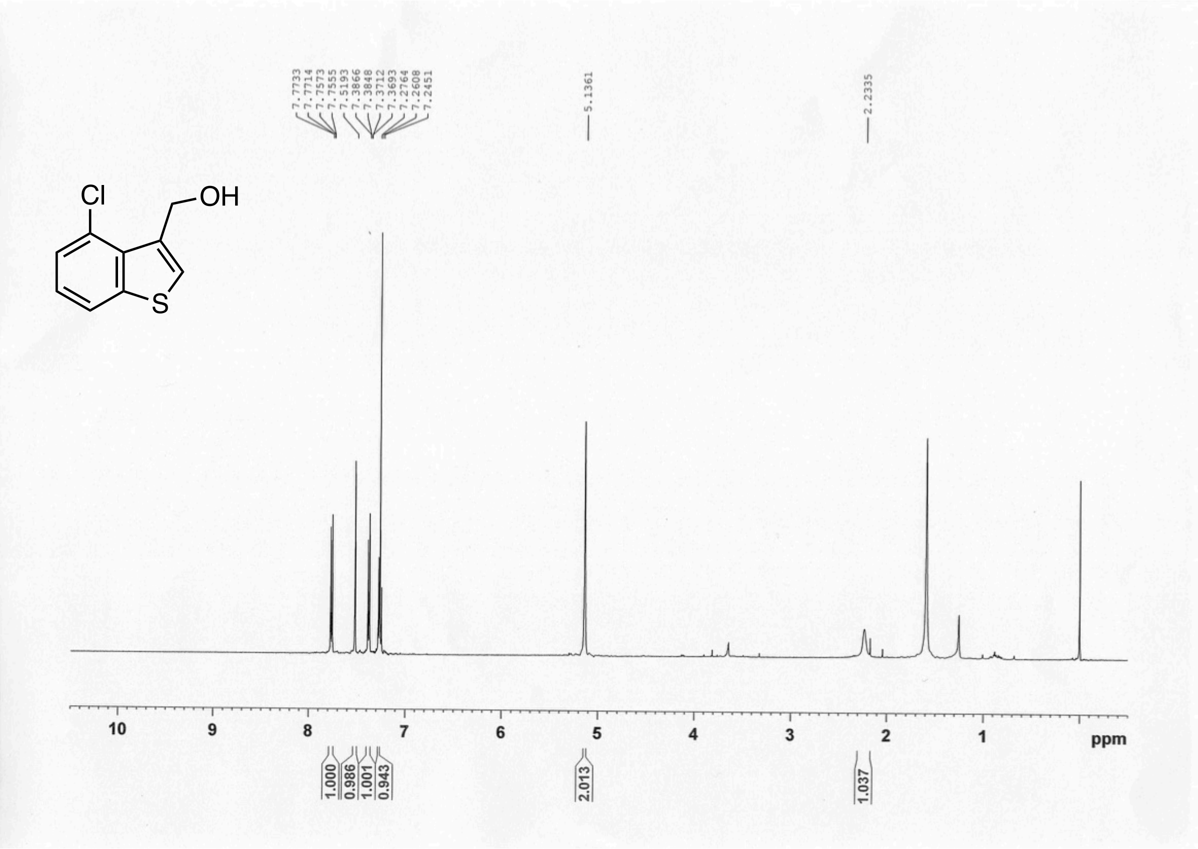

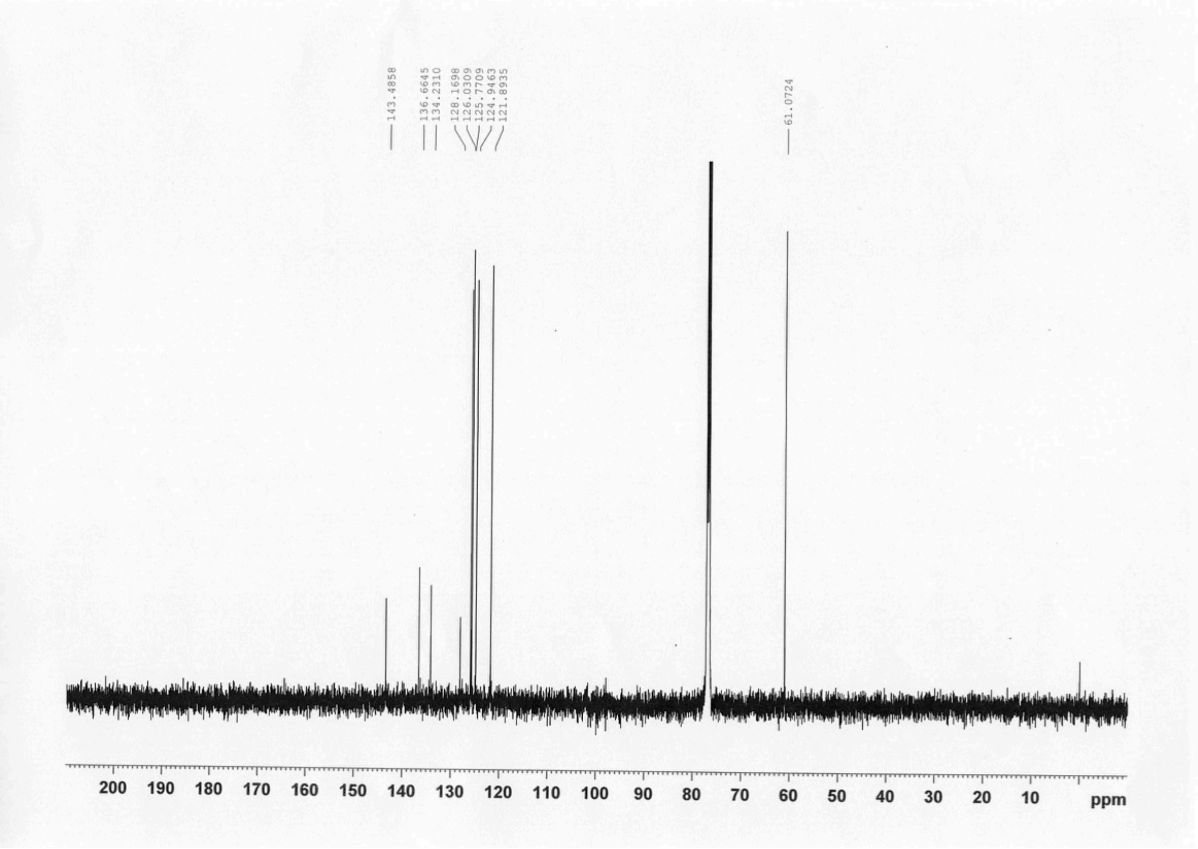

^{1}H NMR (500 MHz) and ^{13}C NMR (126 MHz) spectra of 8-(2-bromophenyl)-2,3-(dibutyl)benzo[1,2-*b*:3,4-*b'*]dithiophene (**3x**) ($CDCl_3$)

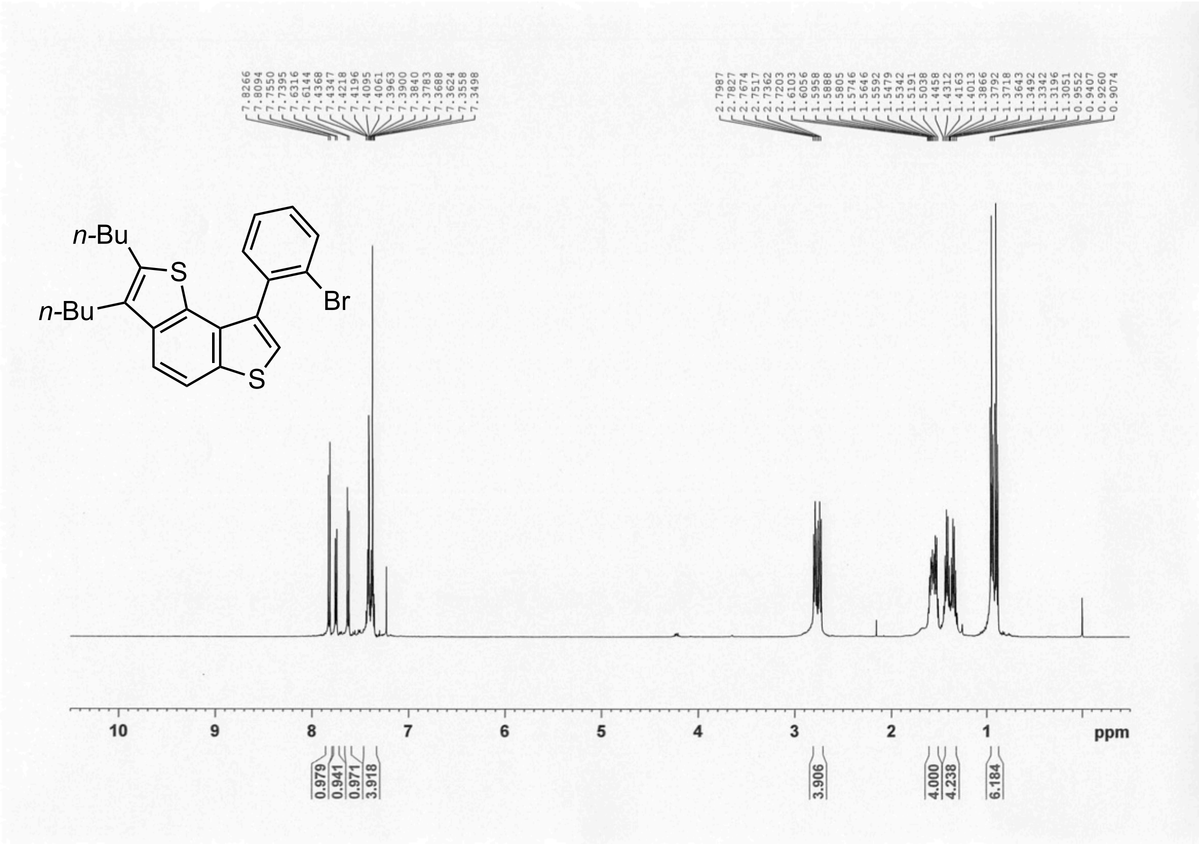

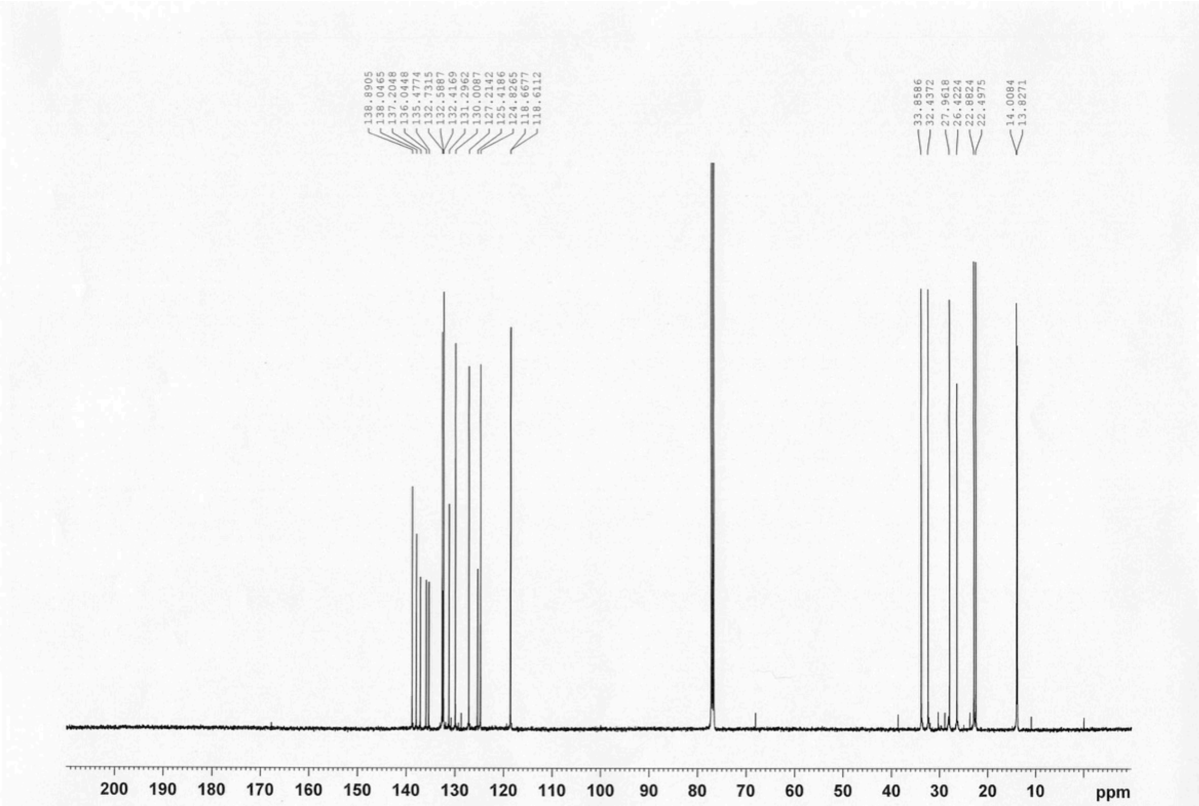

^{1}H NMR (500 MHz) and ^{13}C NMR (126 MHz) spectra of 3-chloro-2-ethoxy-1-((trifluoromethyl)sulfonyl)benzene (**4**) ($CDCl_3$)

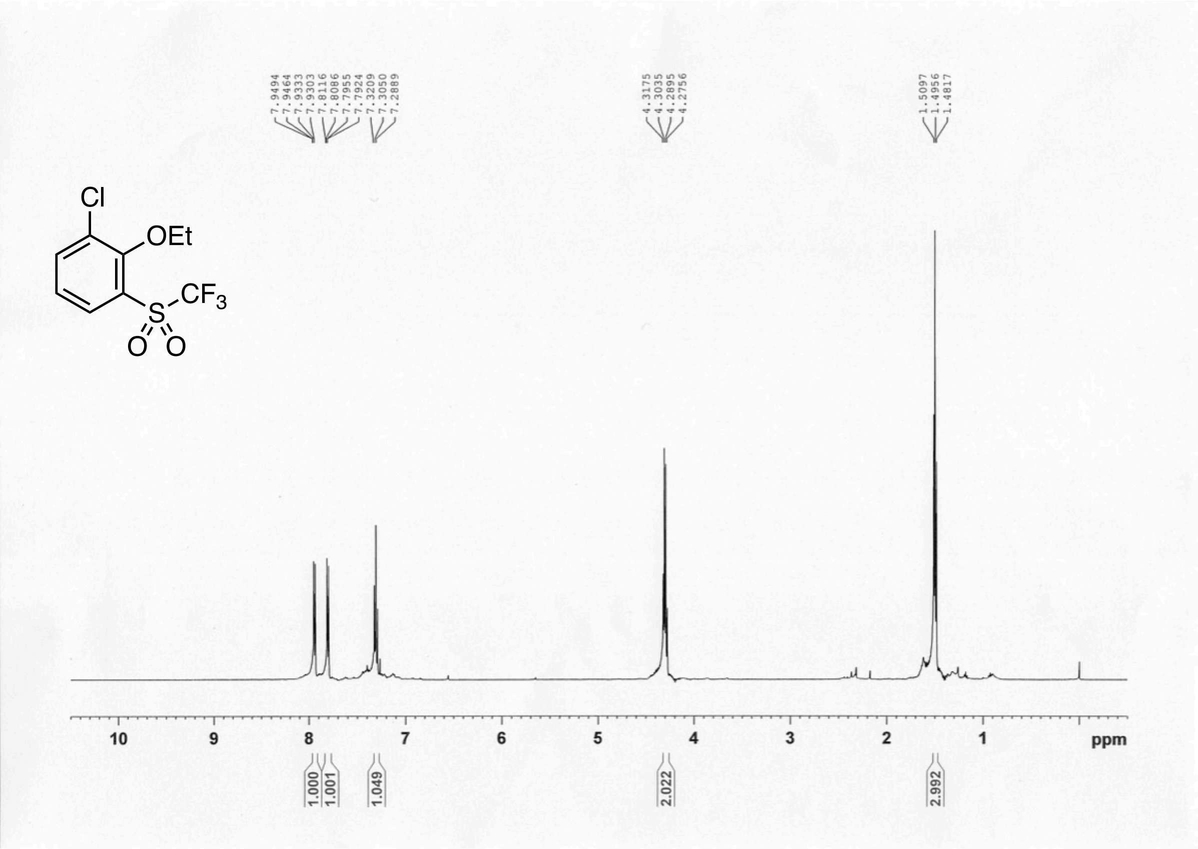

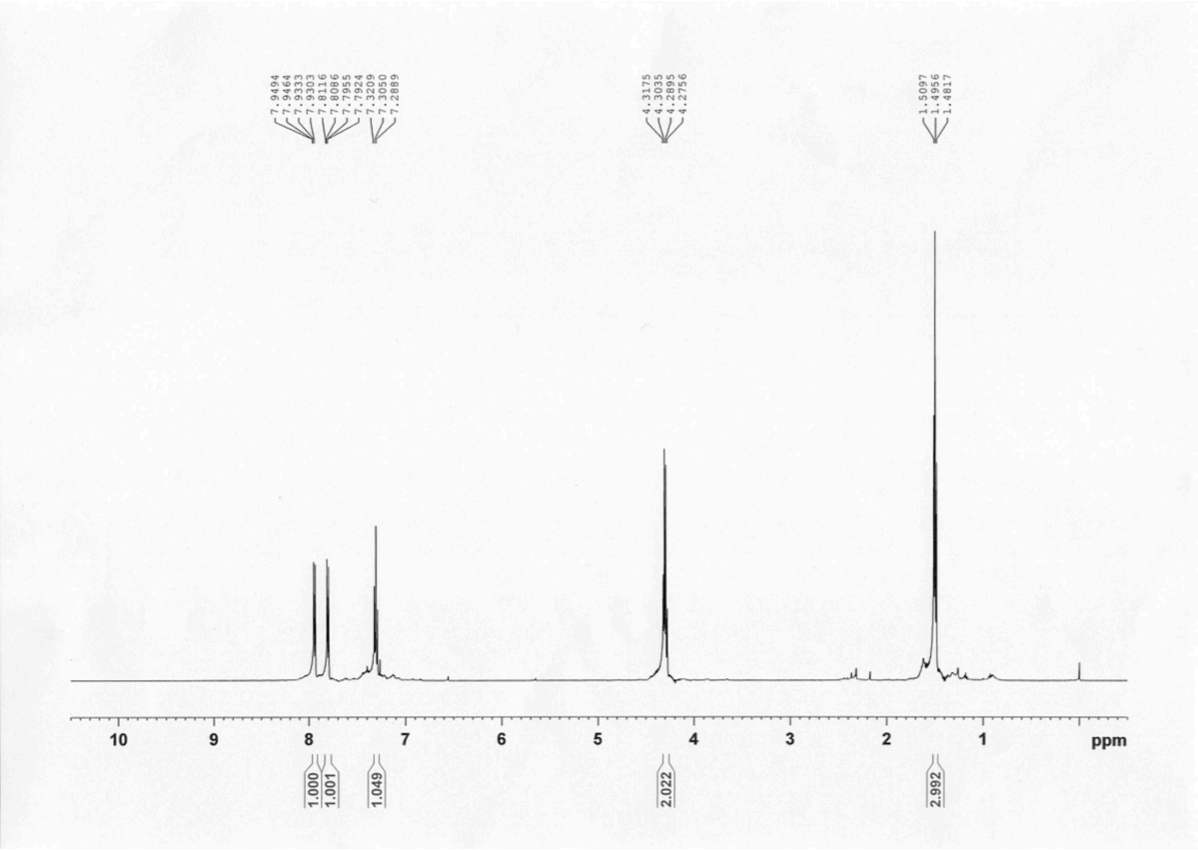

^{1}H NMR (400 MHz) and ^{13}C NMR (101 MHz) spectra of 4-chloro-3-(4-tolyl)-2-(4-tolylthio)benzo[*b*]thiophene (**5a**) ($CDCl_3$)

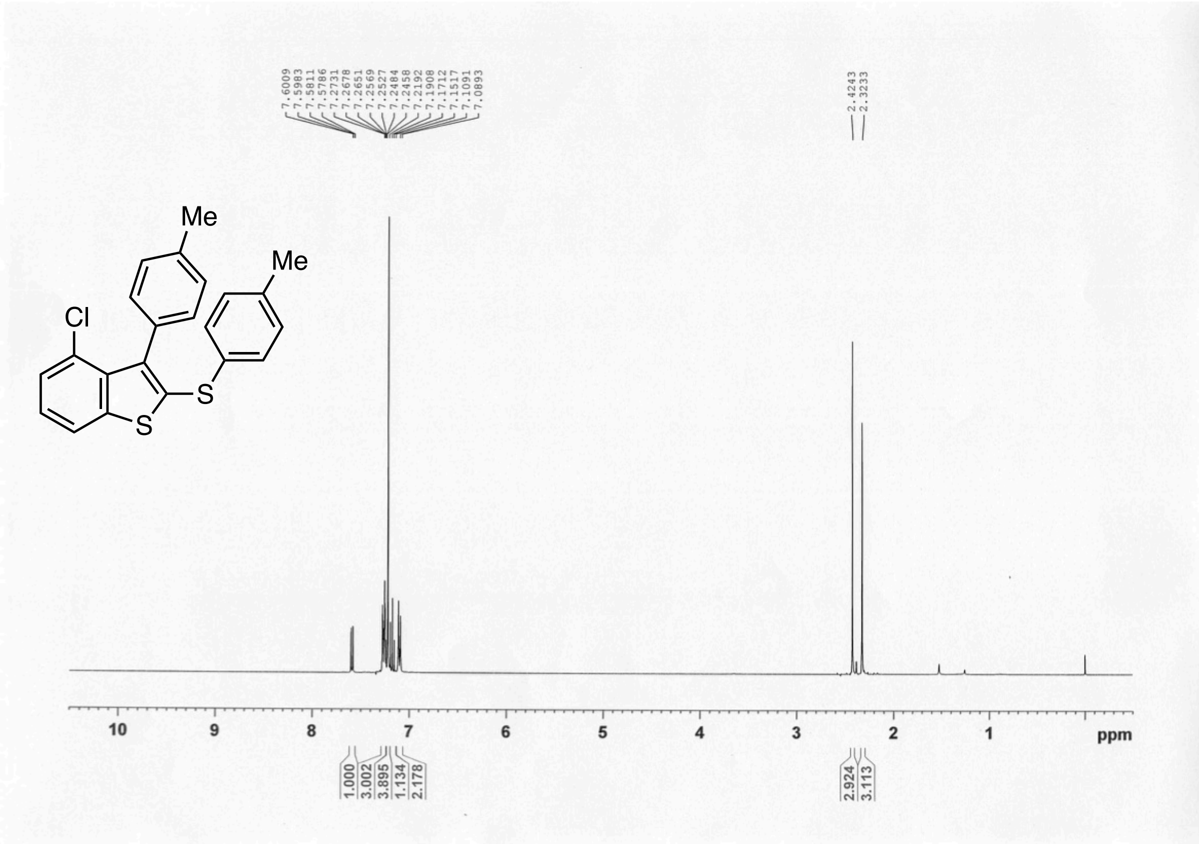

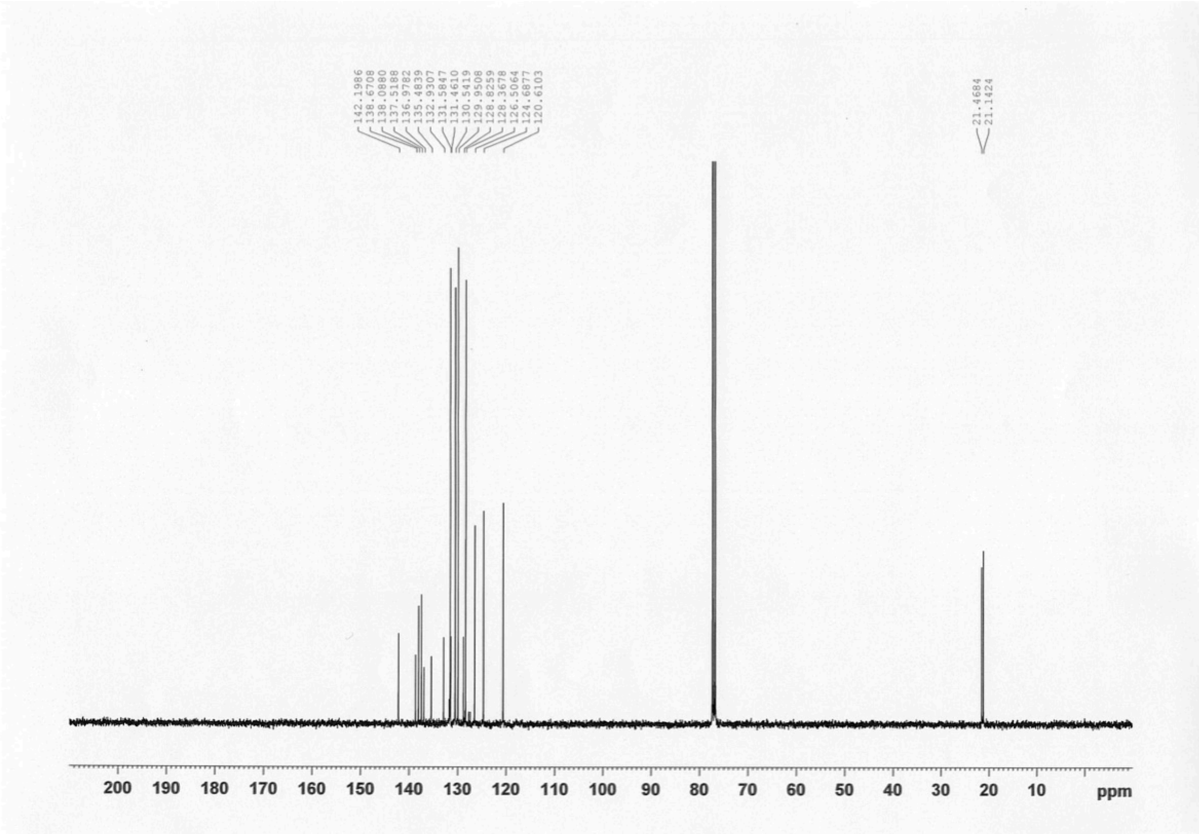

^{1}H NMR (500 MHz) and ^{13}C NMR (126 MHz) spectra of 4-chloro-2-iodo-3-(4-tolyl)benzo[*b*]thiophene (**5b**) ($CDCl_3$)

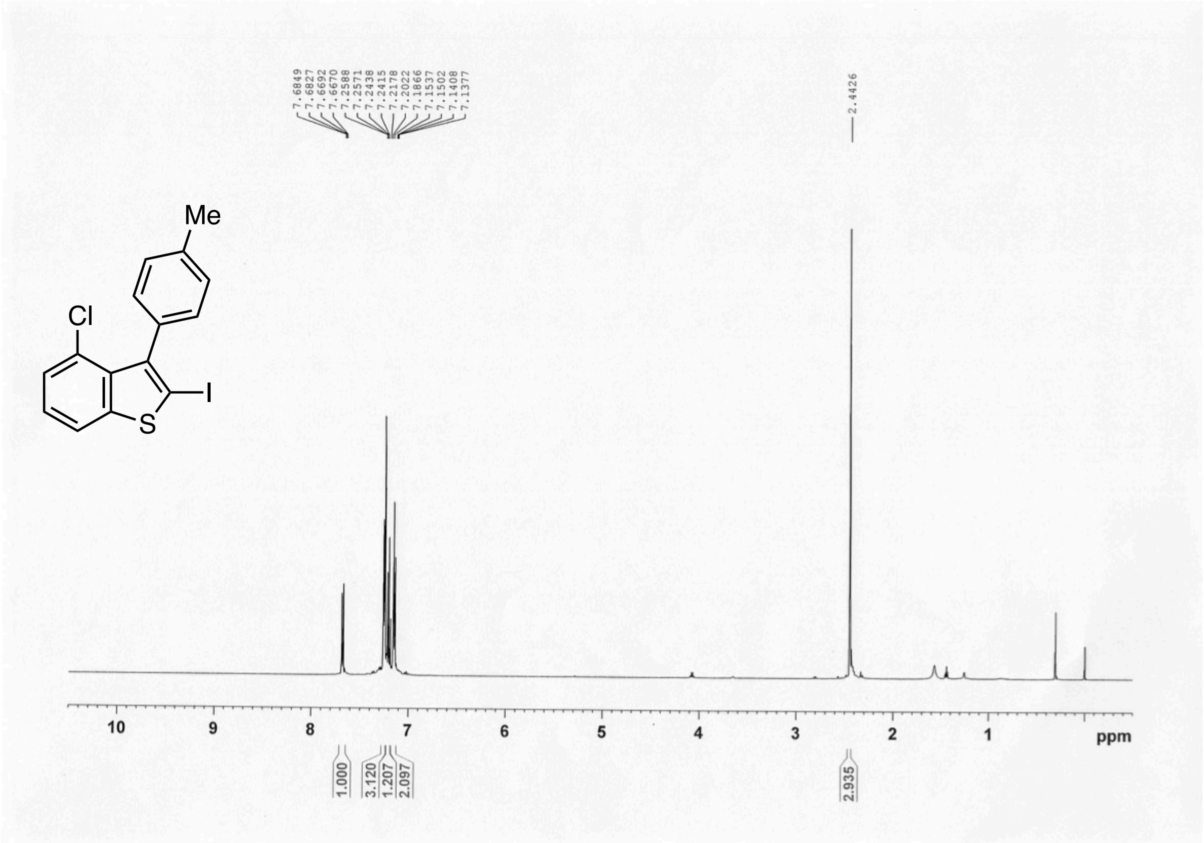

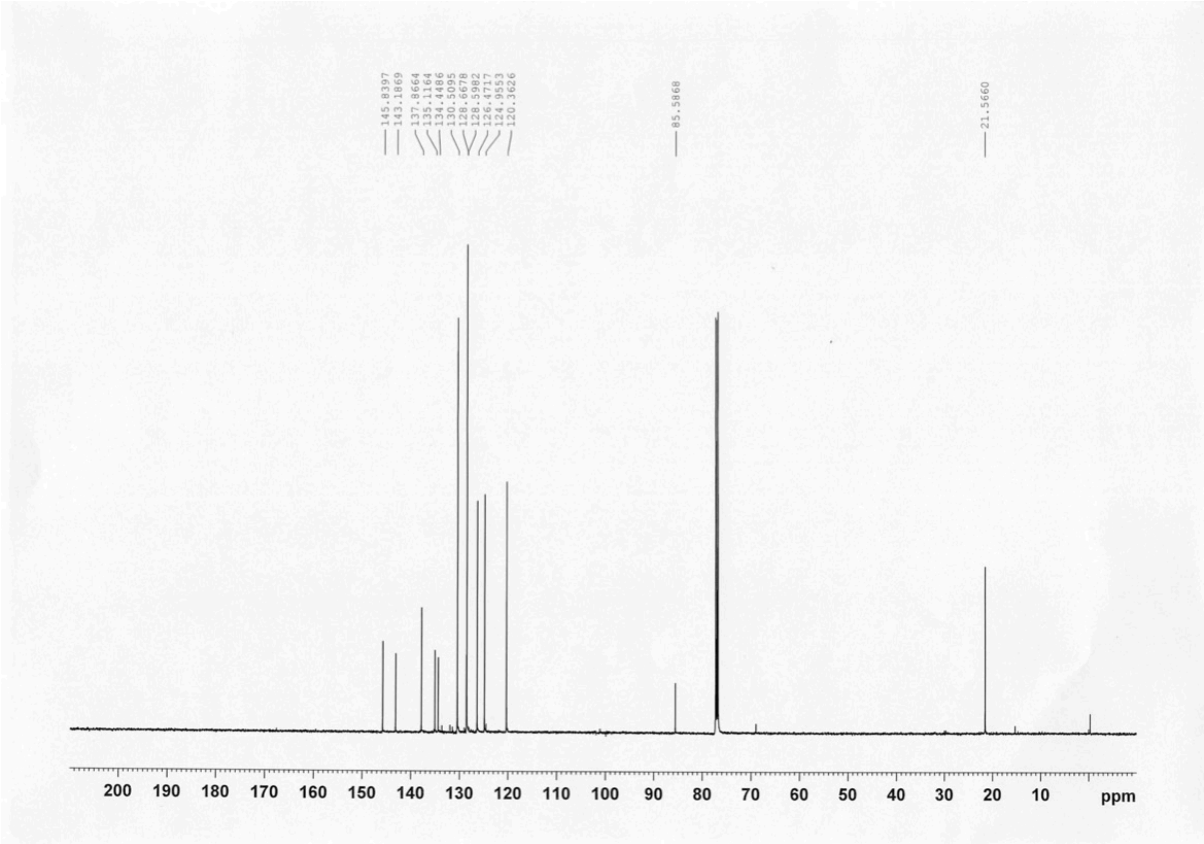

^{1}H NMR (400 MHz) and ^{13}C NMR (101 MHz) spectra of 4-chloro-2-ethoxycarbonyl-3-(4-tolyl)benzo[*b*]thiophene (**5c**) ($CDCl_3$)

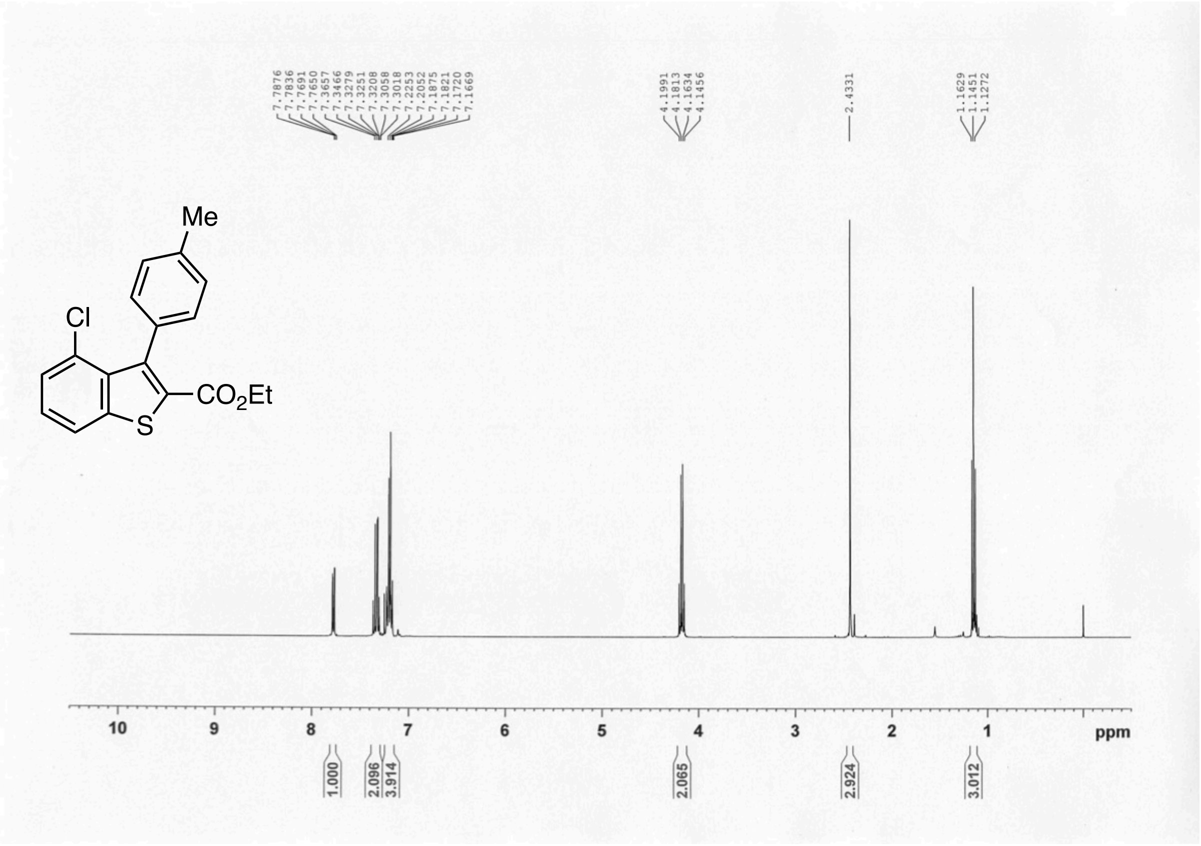

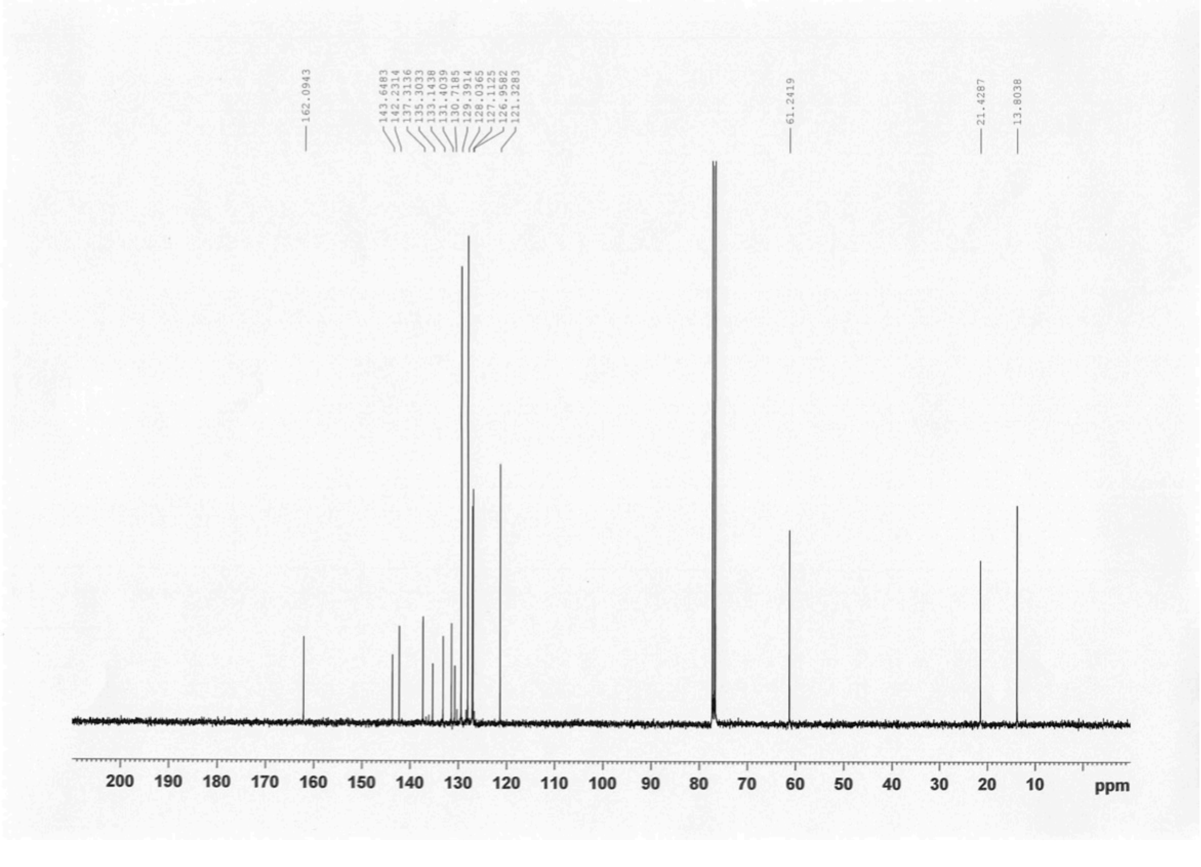

^{1}H NMR (500 MHz) and ^{13}C NMR (126 MHz) spectra of 4-chloro-3-(4-tolyl)benzo[*b*]thiophene *S*-oxide (**6**) ($CDCl_3$)

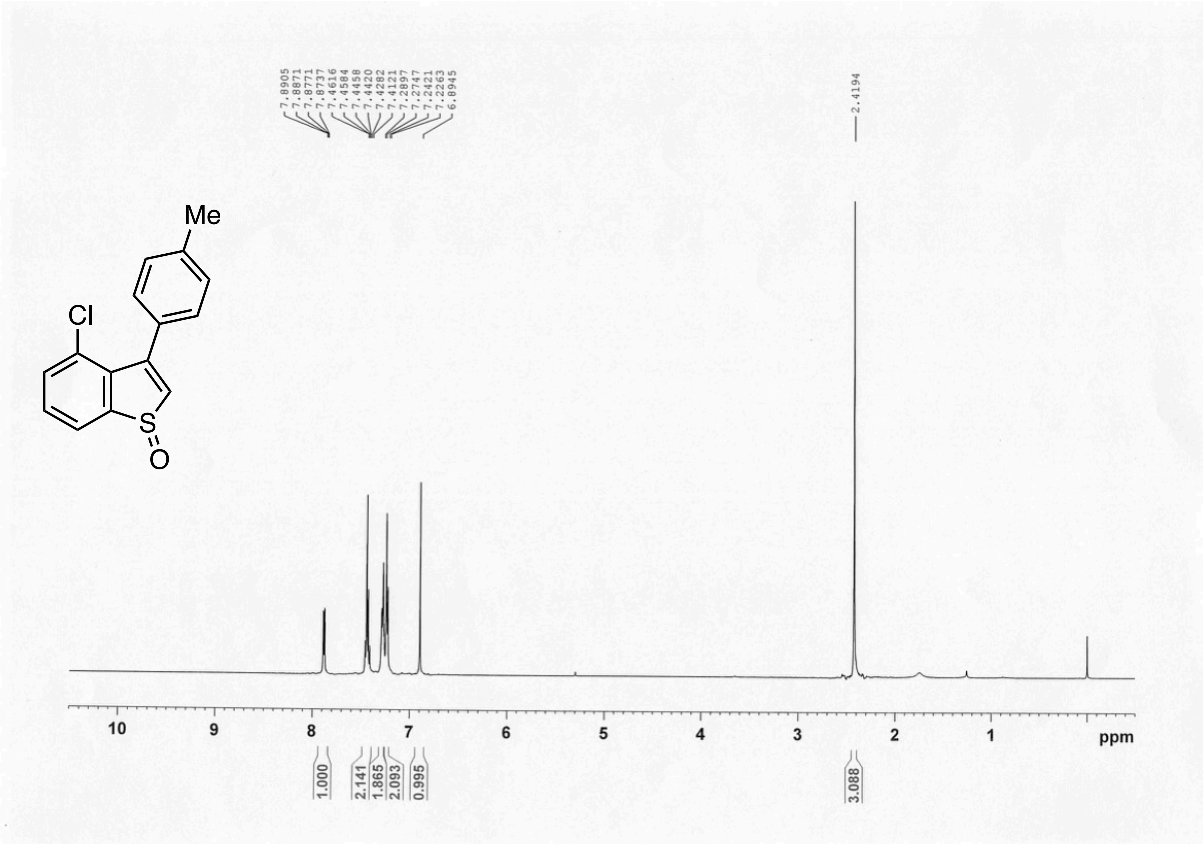

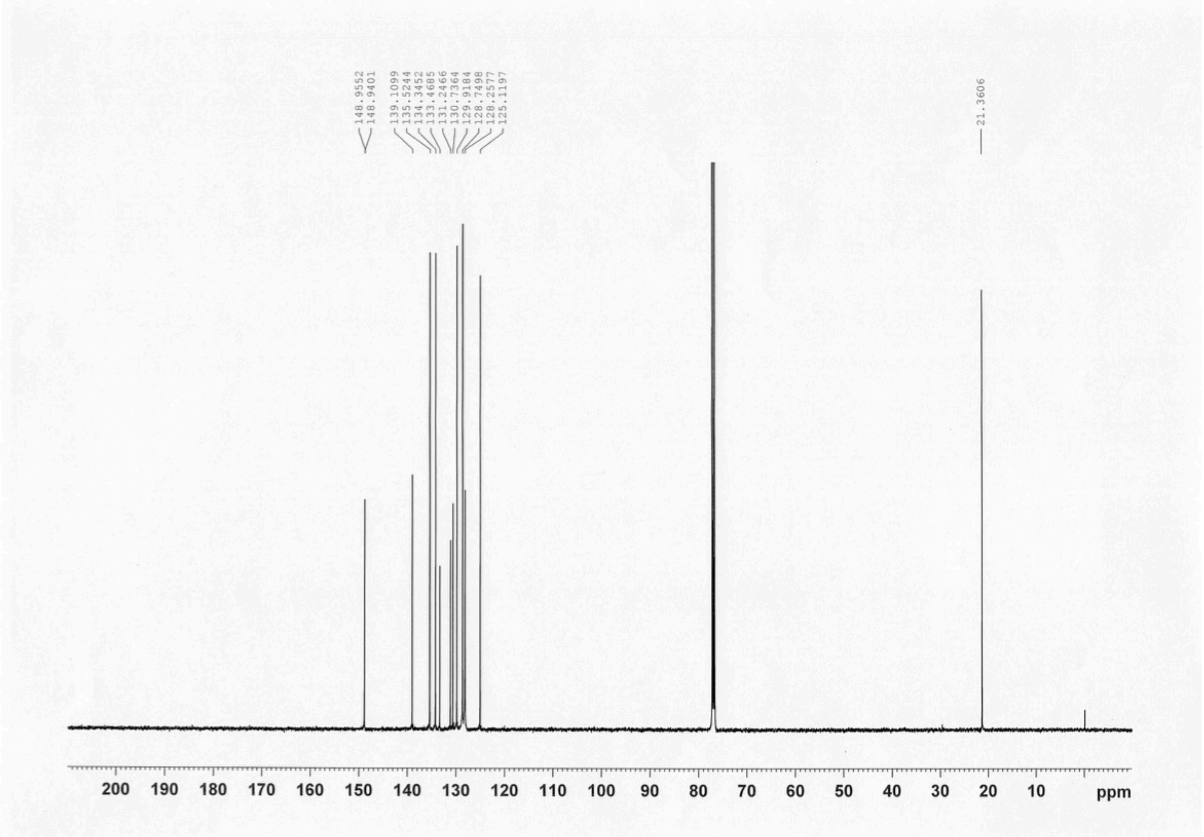

^{1}H NMR (500 MHz) and ^{13}C NMR (126 MHz) spectra of 4-chloro-2-(2-hydroxyphenyl)-3-(4-tolyl)benzo[*b*]thiophene (**7**) ($CDCl_3$)

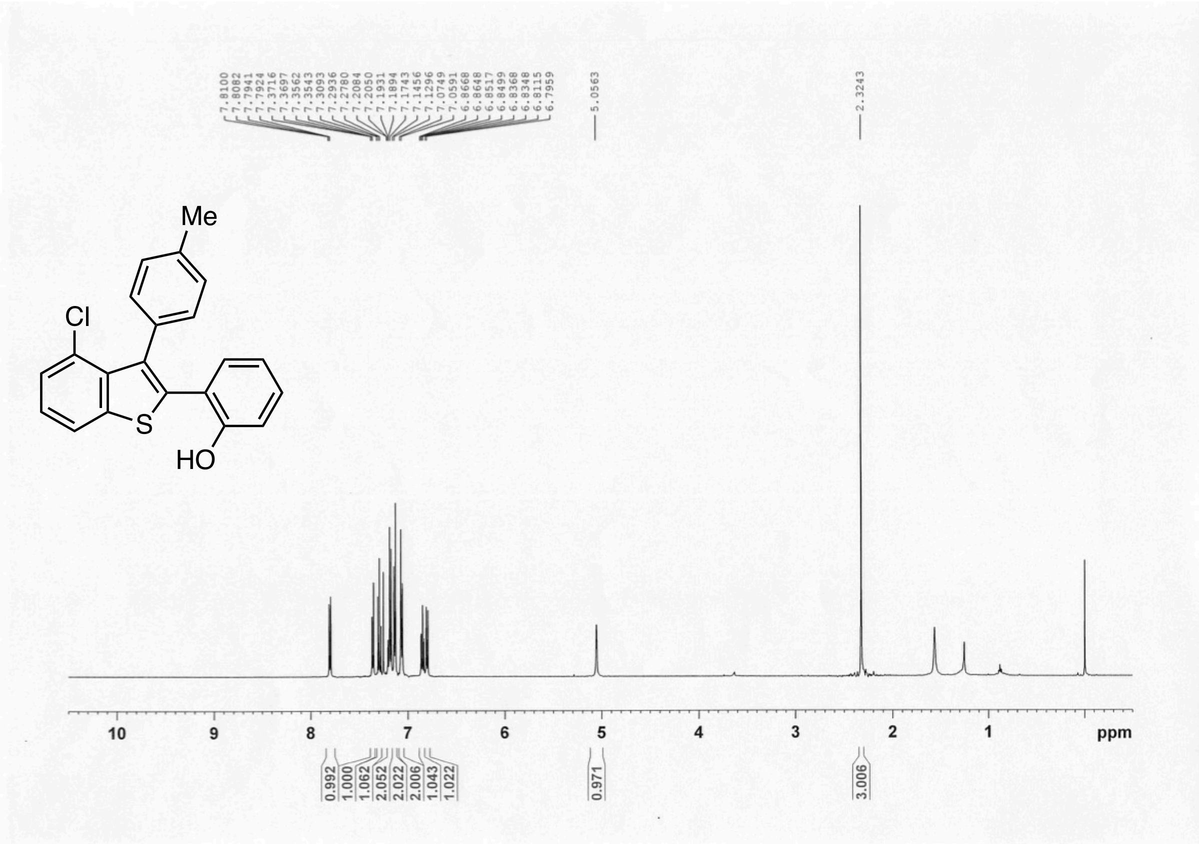

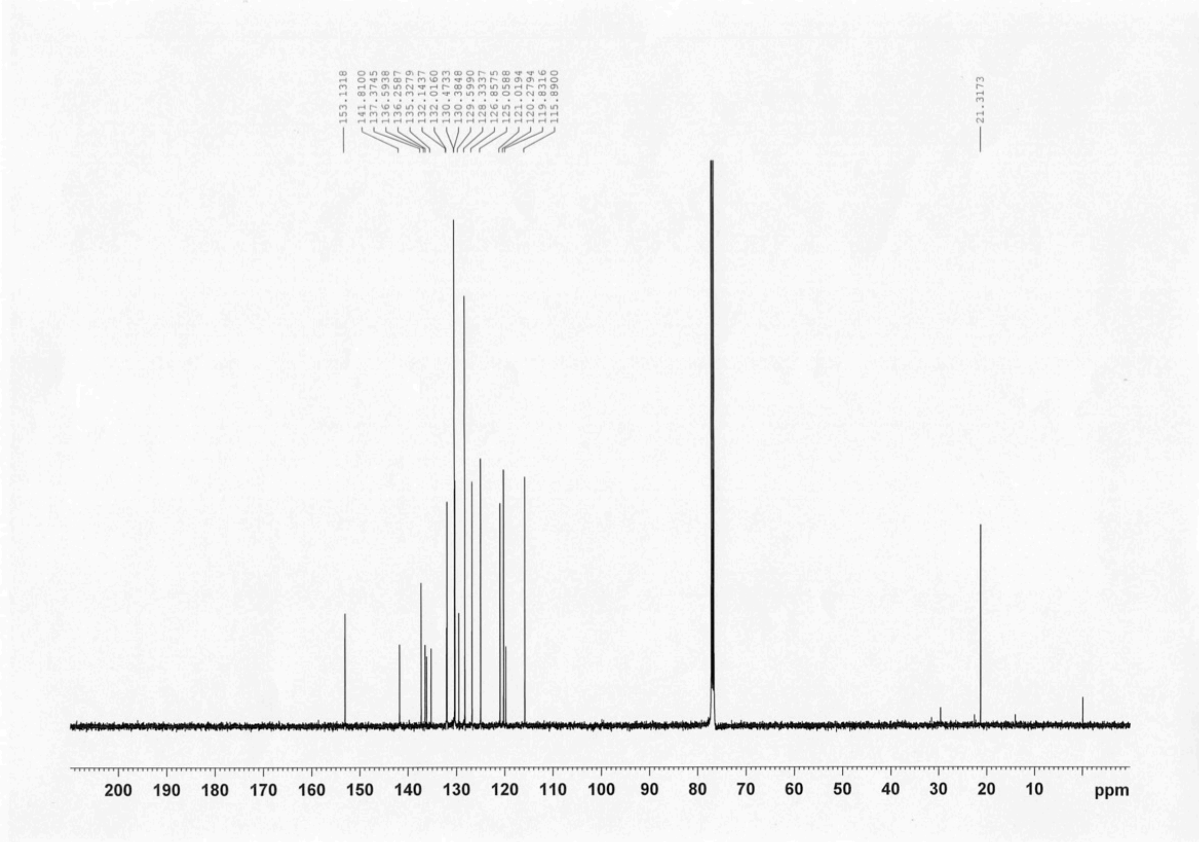

^{1}H NMR (500 MHz) and ^{13}C NMR (126 MHz) spectra of 4-chlorobenzo[*b*]thiophene-3-carboxylic acid (**9**) ($CDCl_3$)

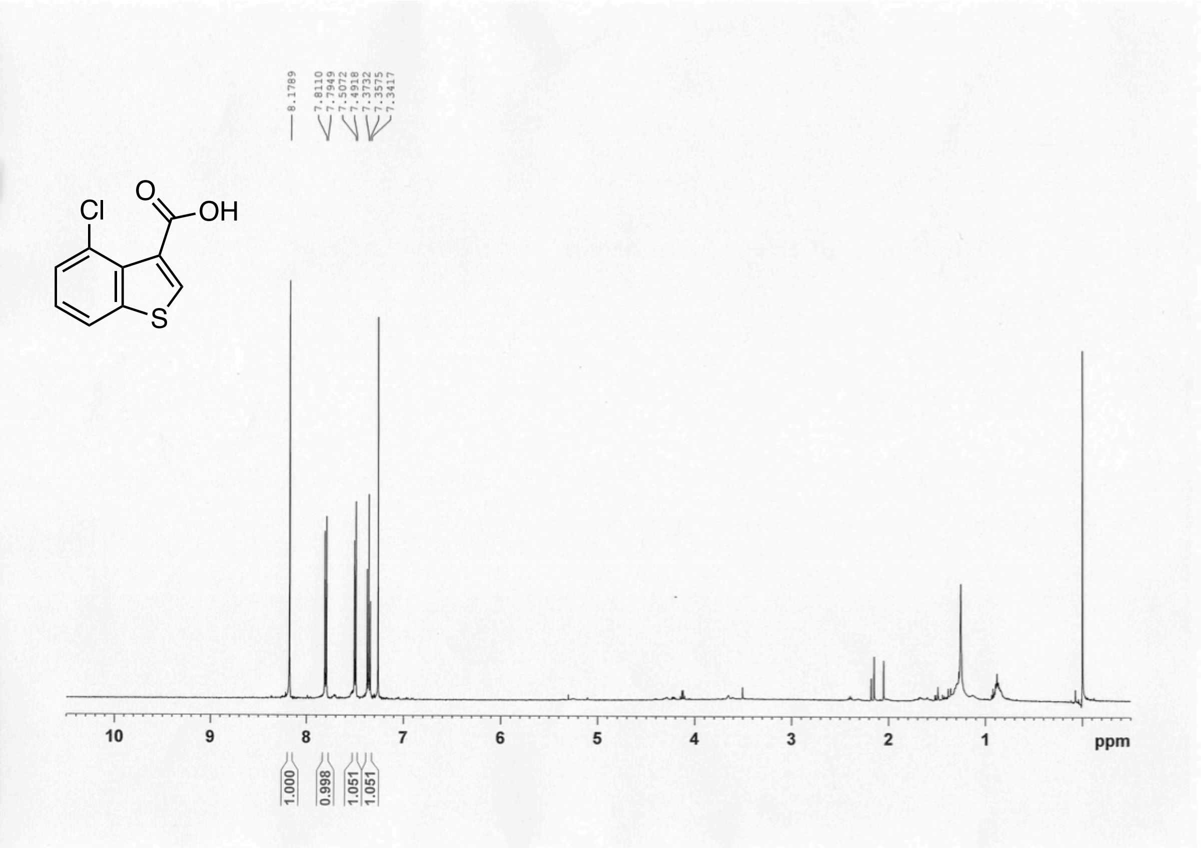

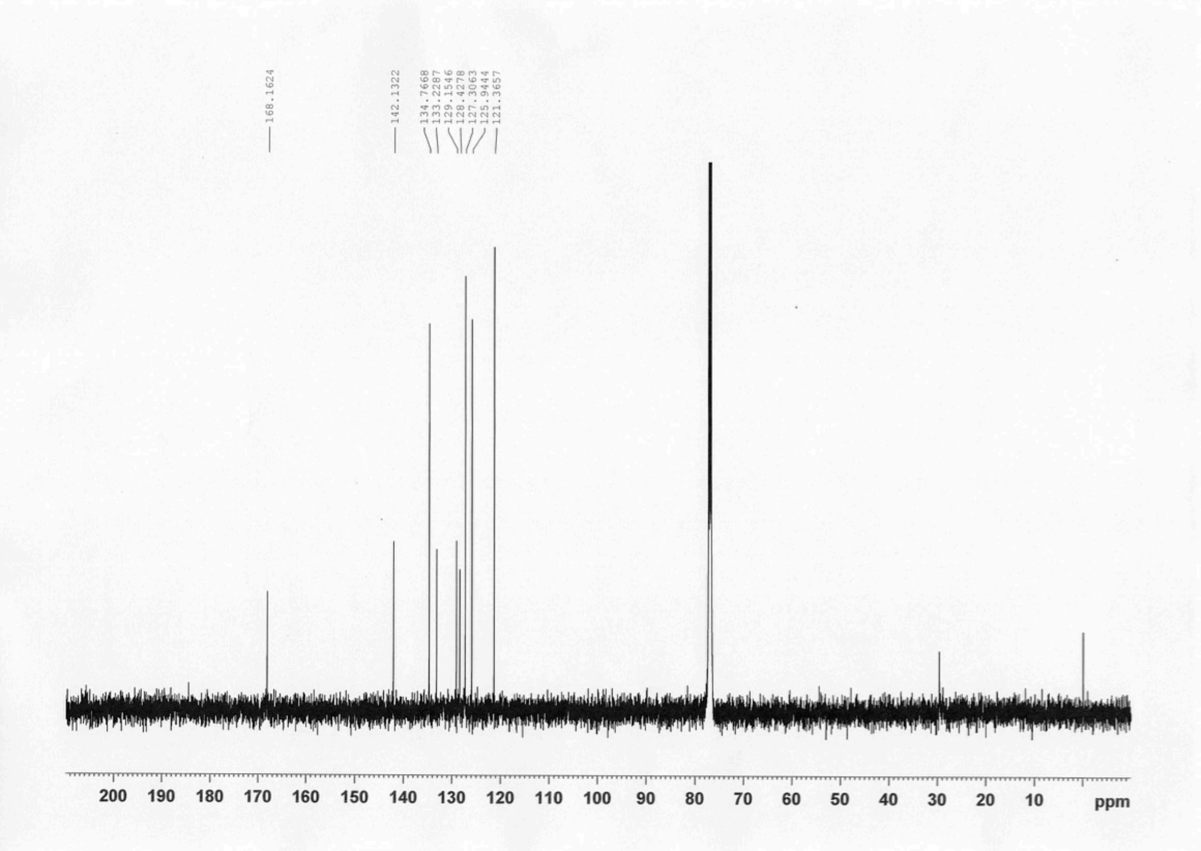

^{1}H NMR (500 MHz) and ^{13}C NMR (126 MHz) spectra of 4-chloro-3-iodobenzo[*b*]thiophene (**10**) ($CDCl_3$)

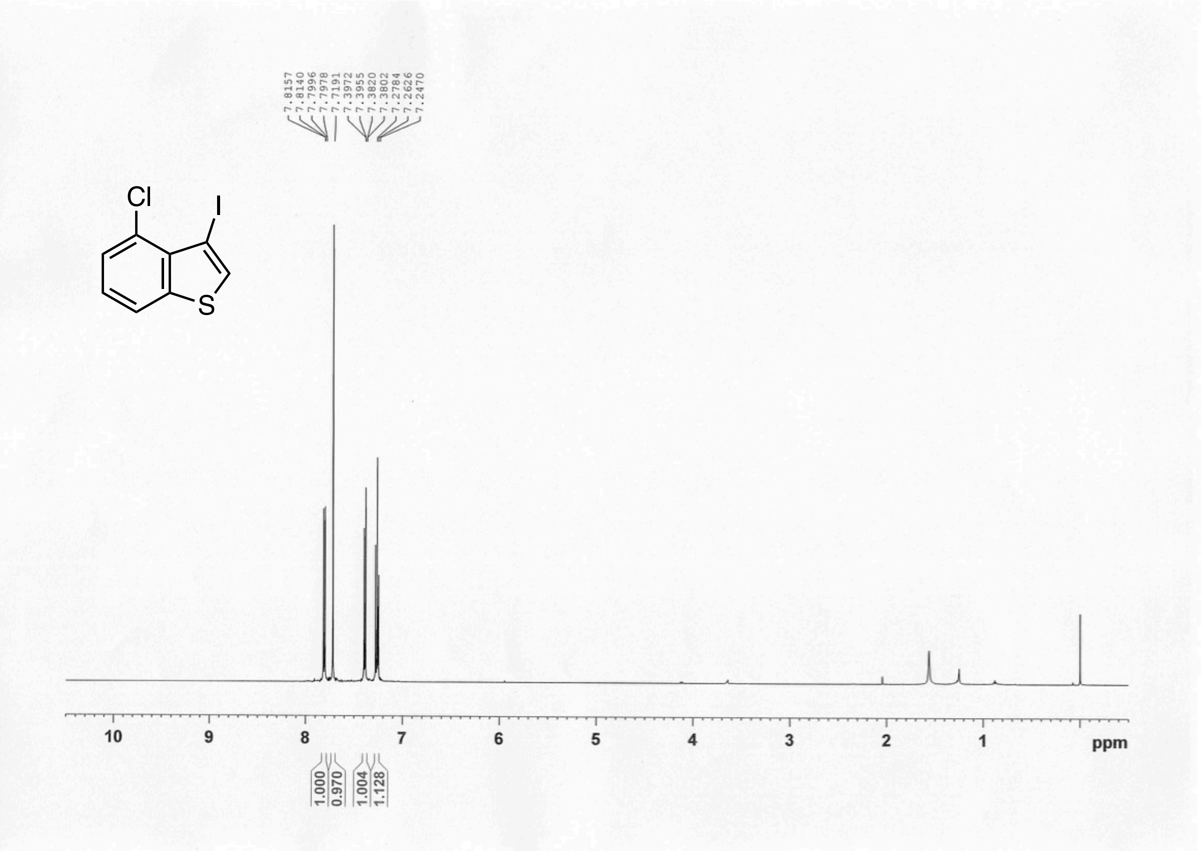

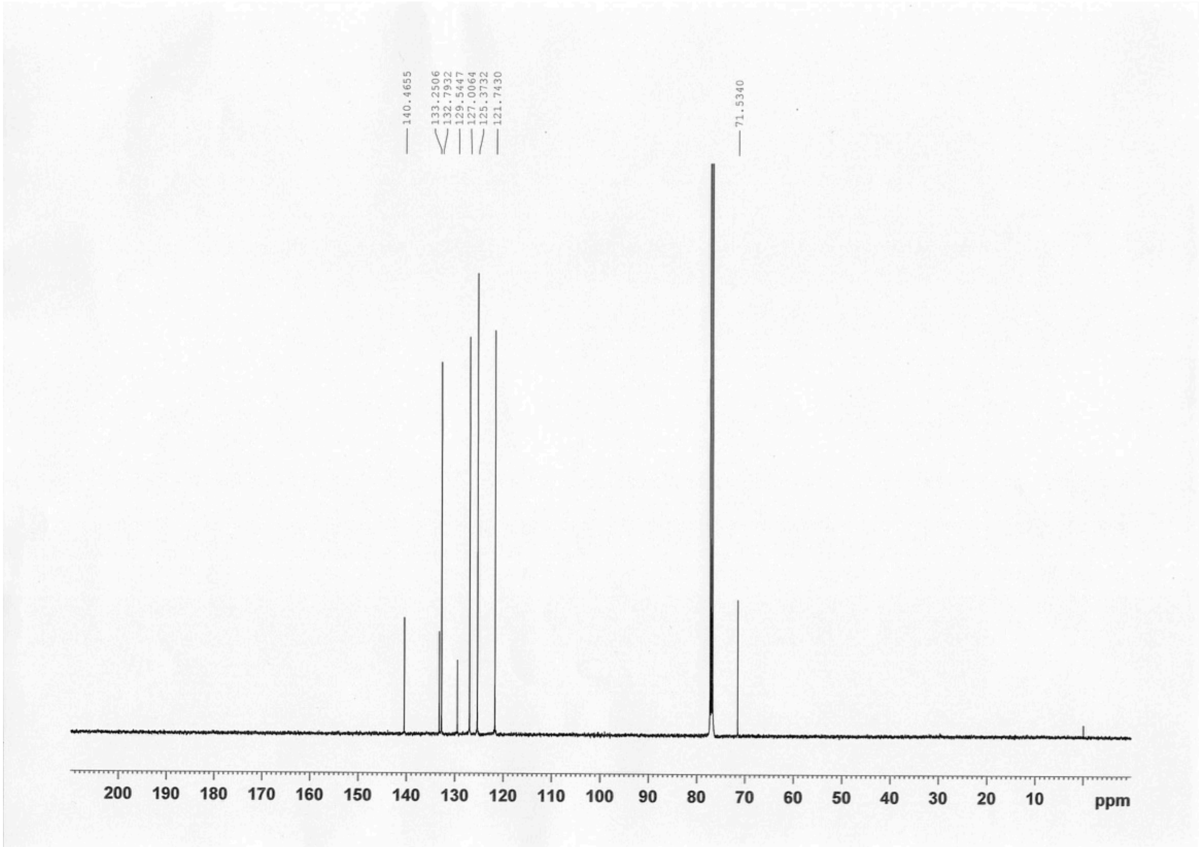

^{1}H NMR (500 MHz) and ^{13}C NMR (126 MHz) spectra of 6-bromo-4-chloro-2-(4-methoxyphenyl)-3-(4-tolyl)benzo[*b*]thiophene (**11**) ($CDCl_3$)

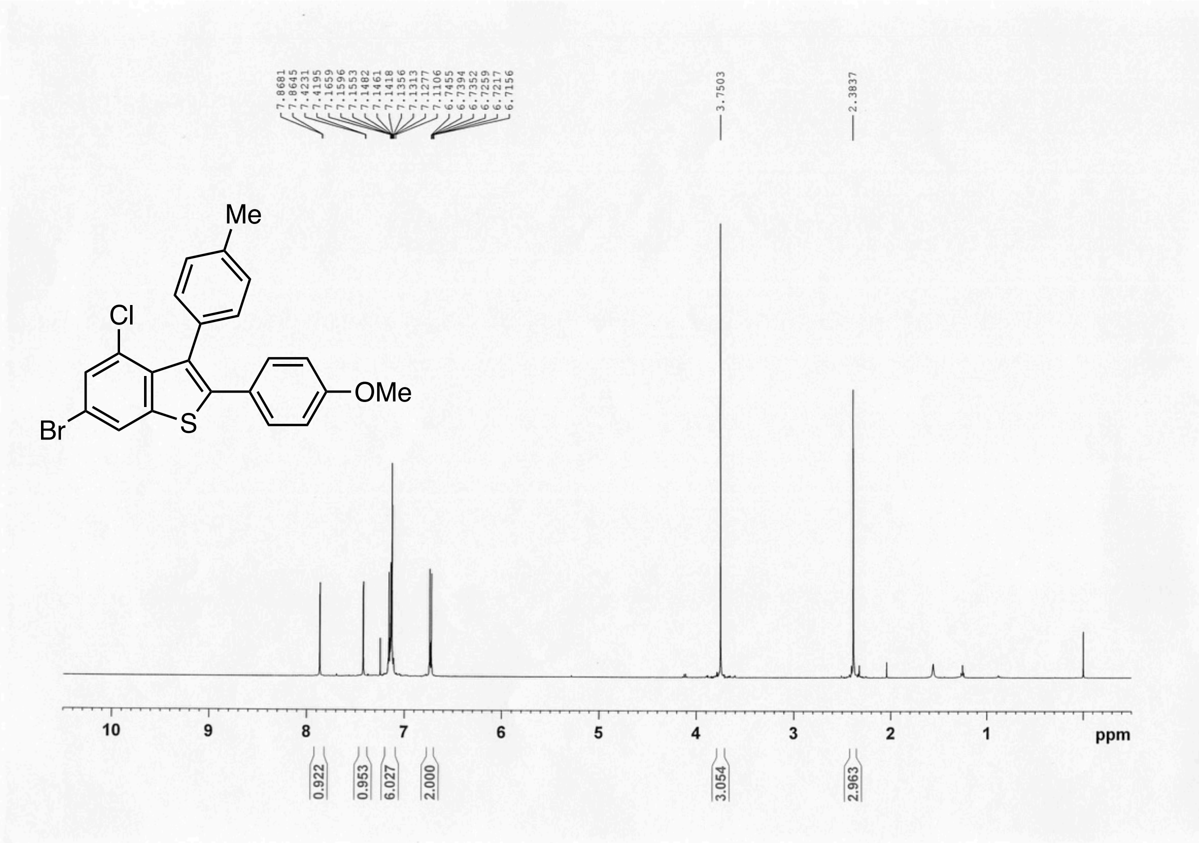

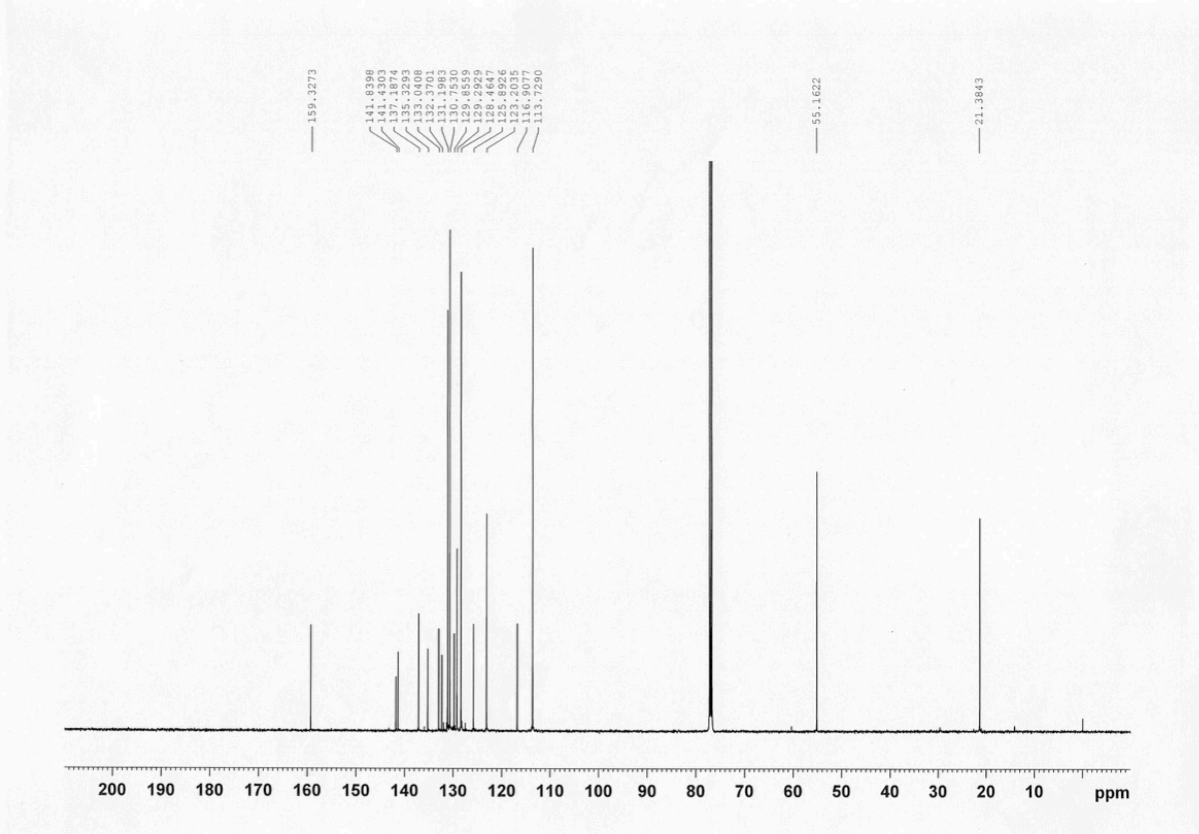

^{1}H NMR (500 MHz) and ^{13}C NMR (126 MHz) spectra of 2-(4-methoxyphenyl)-4-phenyl-3-(4-tolyl)-6-(4-(trifluoromethyl)phenyl)benzo[*b*]thiophene (**12**) ($CDCl_3$)

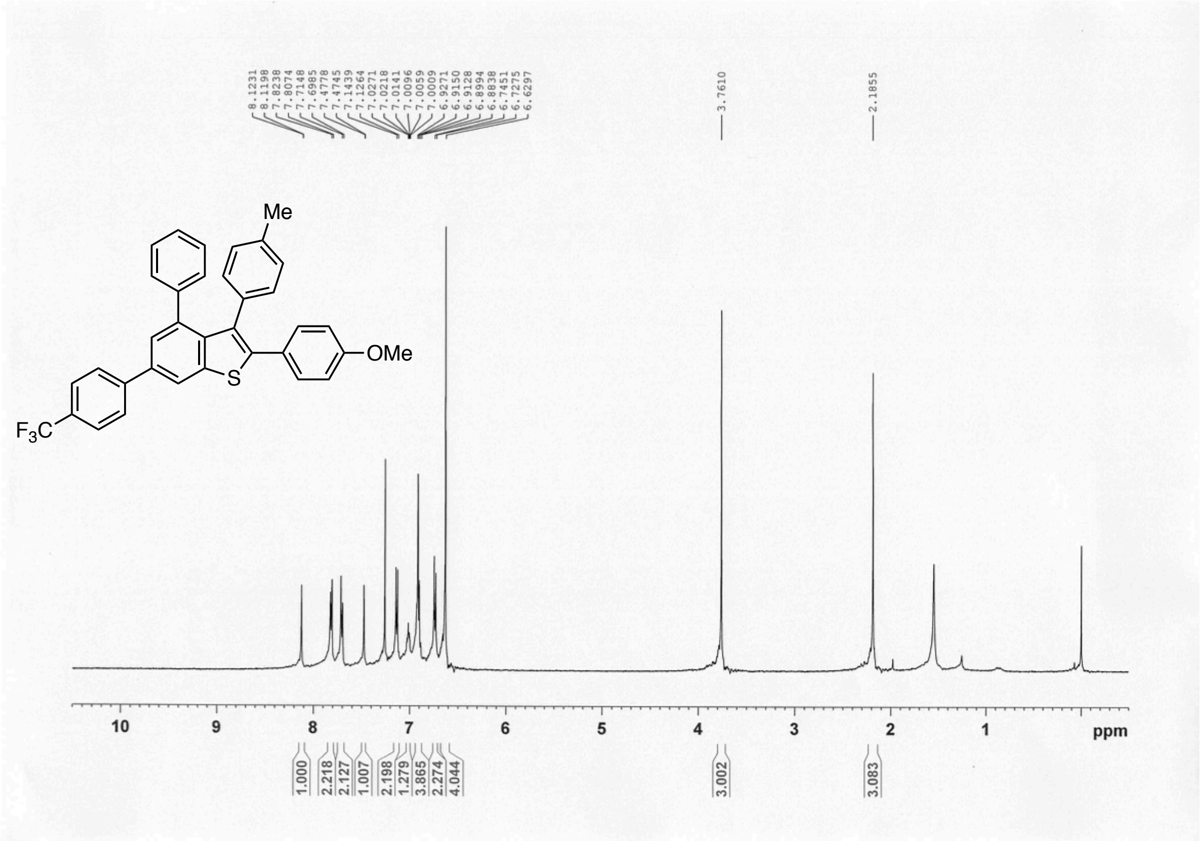

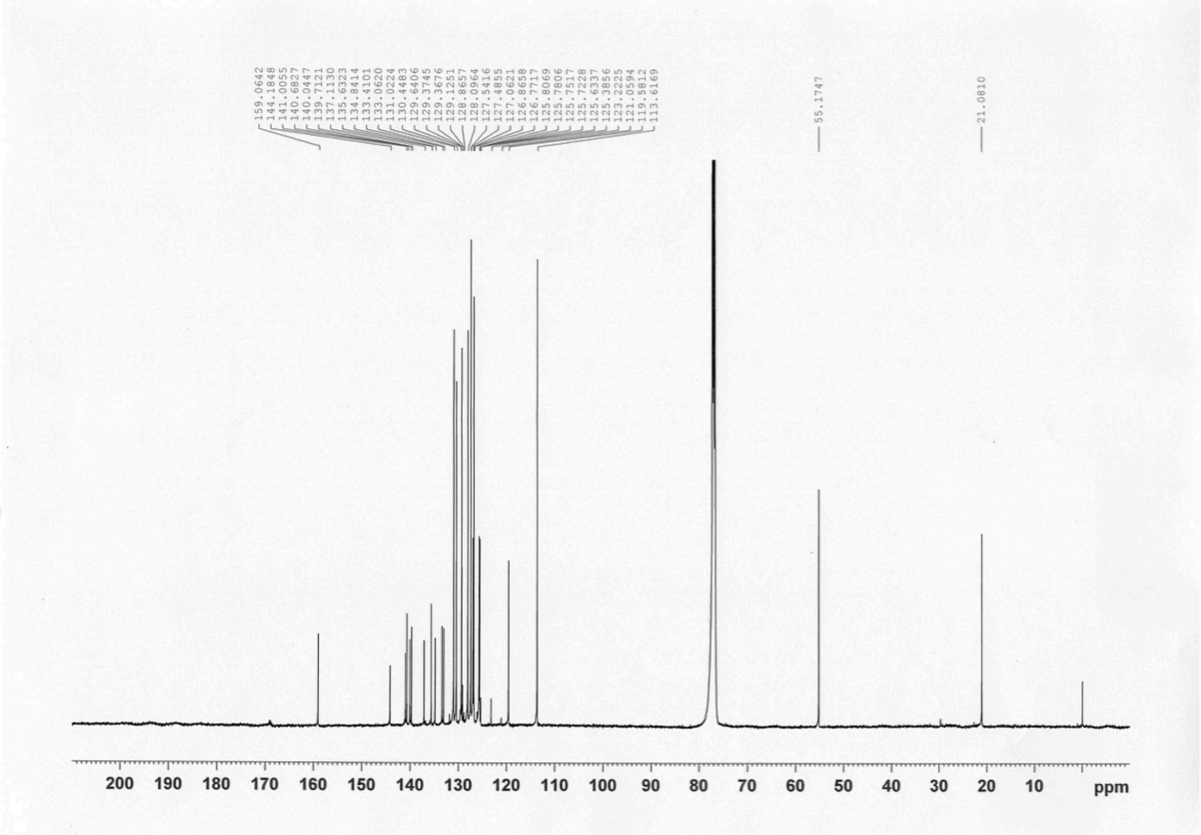

^{1}H NMR (400 MHz) and ^{13}C NMR (101 MHz) spectra of 8-(2-bromophenyl)-2,3-dibutyl-7-iodobenzo[1,2-*b*:3,4-*b′*]dithiophene (**13**) ($CDCl_3$)

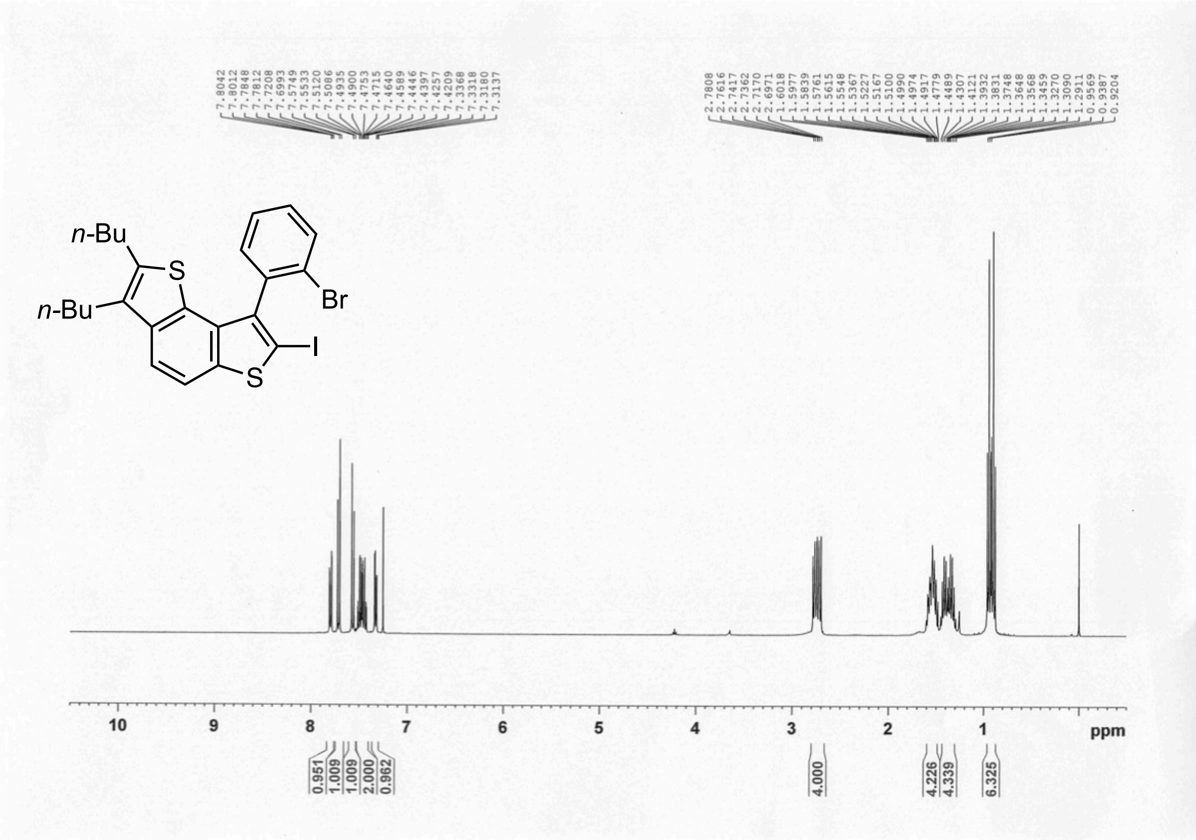

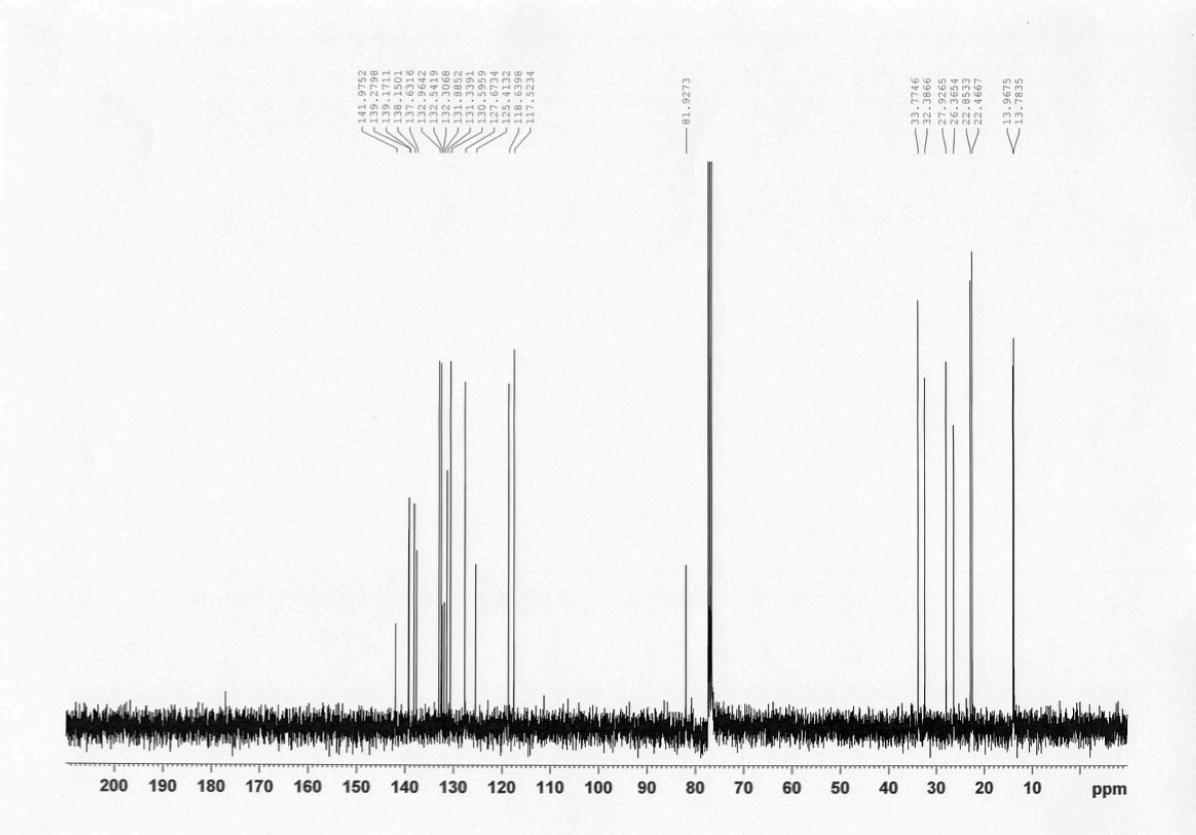

^{1}H NMR (500 MHz) and ^{13}C NMR (126 MHz) spectra of 2,3-dibutyl-7-(4-tolyl)-7*H*-thieno[3'',2'':5',6']benzo[1',2':4,5]thieno[2,3-*b*]indole (**14**) ($CDCl_3$)

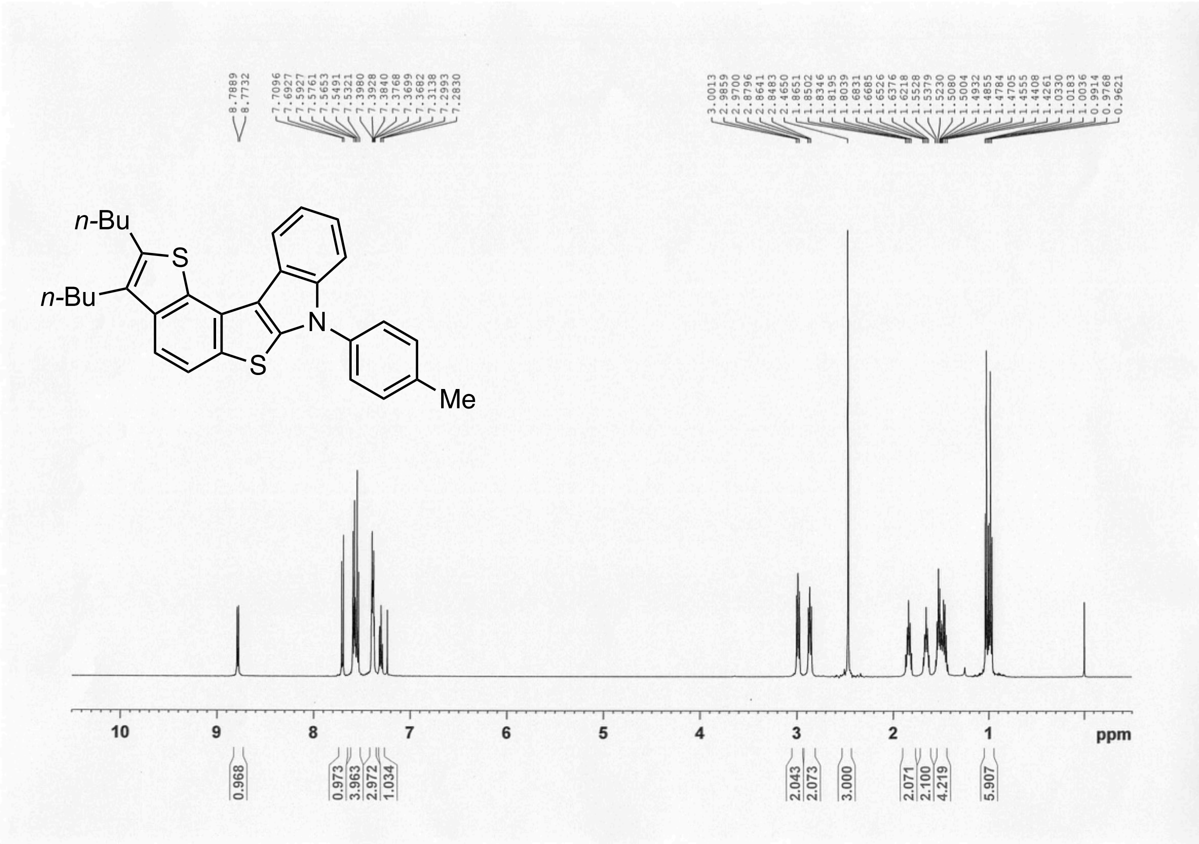

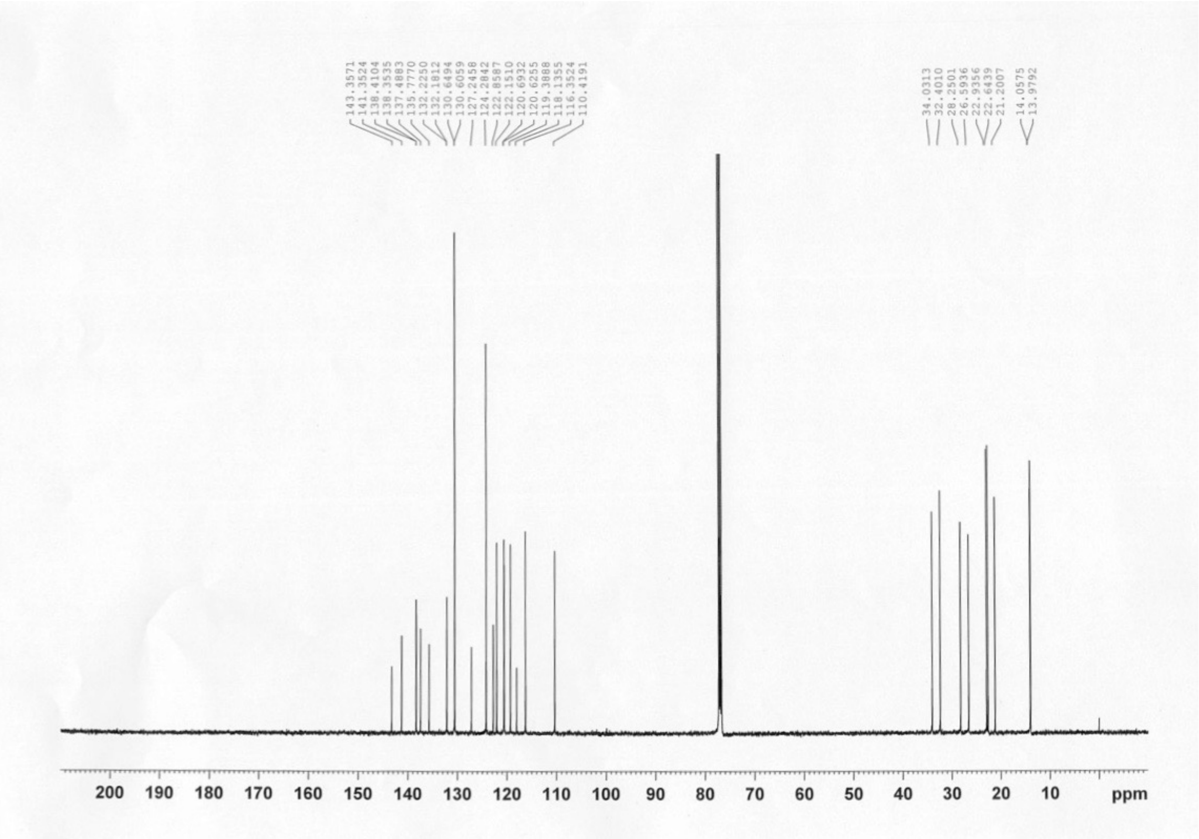

Supplement: SC-011-D0SC04450D-s001 [file SC-011-D0SC04450D-s001.pdf]
